# Intramolecular CH‐Hydrogen Bonding During the Dissociation of the Oxaphosphetane Intermediate Facilitates *Z/E*‐Selectivity in Wittig Olefination

**DOI:** 10.1002/open.202300171

**Published:** 2023-12-07

**Authors:** Kukkamudi Sreenivas, Chintada Nageswara Rao, Faiz Ahmed Khan

**Affiliations:** ^1^ Department of Chemistry Indian Institute of Technology Hyderabad Kandi Sangareddy Telangana 502 285 India; ^2^ Department of Drug Discovery and Biomedical Sciences, College of Pharmacy University of South Carolina Columbia SC-29208 USA; ^3^ Department of Chemistry Indian Institute of Technology Hyderabad, Kandi Sangareddy Telangana 502 285 India

**Keywords:** benzylphosphonium salts, Wittig olefination, hydrogen bonding, nitrostilbenes, 2-arylindoles

## Abstract

Herein, DFT studies corroborating experimental results revealed that the shortest intramolecular hydrogen bonding distance of *cis/trans*‐oxaphosphetane (OPA) oxygen with the CH‐hydrogen of a triphenylphosphine phenyl ring provides good evidence for the attained olefin *Z/E*‐selectivity in Wittig olefination of the studied examples. 2‐Nitrobenzaldehyde, 3‐nitrobenzaldehyde, 2‐nitro‐3‐bromobenzaldehyde, 2‐nitro‐5‐bromobenzaldehyde and 2‐nitro‐5‐arylbenzaldehydes provided *Z*‐nitrostilbenes with (2‐chloro‐4‐hydroxy‐3‐methoxy‐5‐(methoxycarbonyl)benzyl) triphenylphosphonium chloride as the major products. However, 4‐nitrobenzaldehyde and 2‐nitro‐6‐bromobenzaldehydes furnished *E*‐nitrostilbenes as the major products in high yields. Furthermore, the DFT computed intramolecular CH1/CH2‐hydrogen bond distances with Cl/NO_2_ of selected stilbene derivatives were in good agreement with intramolecular hydrogen bond distances measured from single‐crystal X‐ray diffraction measurements.

## Graphical abstract

The *Z*‐selective synthesis of novel nitrostilbenes has been reported. An intrinsic role of intramolecular hydrogen bonding during the dissociation of OPA‐intermediate is critical for the stereoselective Wittig olefination. DFT calculations and X‐ray measured intramolecular CH hydrogen bonding distances strongly support the observed phenomenon. Further, the current methodology has been utilized to synthesize medicinally potential salicylate‐methyl‐ester‐based 2‐arylindole derivatives.

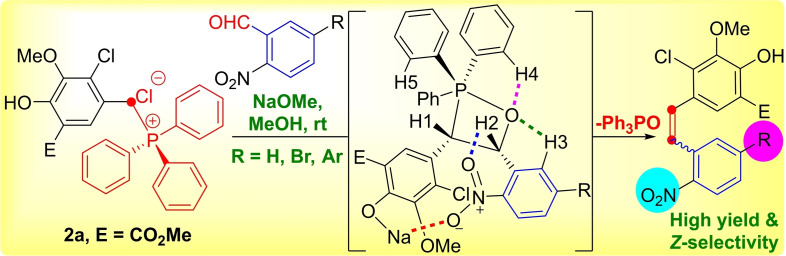

## Introduction

**Figure 1:**
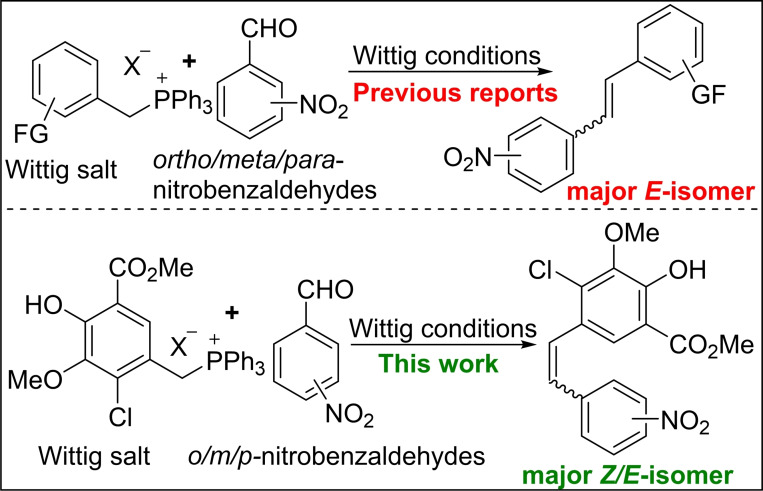
Wittig olefination of nitrobenzaldehydes.

Nitro group containing stilbenes and their derivatives possess numerous applications in organic synthesis. Owing to the conversion of the nitro group to other functional groups (azo, amino, *N*‐oxides, etc.) and heterocyclic compounds, nitrostilbenes have gained tremendous importance in medicinal chemistry.[^1^, ^2^] Apart from the metal‐catalysed couplings[^3^, ^4^, ^5^, ^6^, ^7^] and other reactions,^[8]^ the classical Wittig olefination method has usually been employed to synthesize these stilbenes.[^9^, ^10^] In the majority of cases, the *trans or E*‐nitrostilbene was formed as a major isomer over *cis or Z*‐nitrostilbene from all the three positional isomers of nitrobenzaldehyde, *ortho*‐nitrobenzaldehyde, *meta*‐nitrobenzaldehyde, and *para*‐nitrobenzaldehyde (Figure 1).[^1^, ^2^, ^3^, ^4^, ^5^, ^6^, ^7^, ^8^, ^9^, ^10^]

Unlike *meta/para*‐substituted‐benzaldehydes, the general olefin stereoselectivity in the case of *ortho*‐substituted benzaldehydes possessing β‐hetero‐atom or group usually provides *cis or Z*‐selective stilbenes due to the ancillary phosphorus coordination with hetero‐atom or group.[^11^, ^12^] Rebutting the ancillary phosphorus coordination hypothesis, a qualitative analysis conducted by Marcin Stępień by utilizing density functional theory (DFT) calculations coupled with a distortion or interaction energies show that the *Z*‐selectivity observed in Wittig olefinations of *ortho*‐substituted benzaldehydes is mainly due to the steric reasons.^[13]^

Due to the strong electron‐withdrawing nature of the aldehyde group, the adjacent C−H hydrogen atom on benzaldehyde is more acidic than the C−H hydrogen on benzene. A suitable example of this milieu is a regiospecific functionalization of substituted benzaldehydes via metal‐catalysed C−H activation.^[14]^ However, without metal catalysts, any electron withdrawing group or atom proximal to the acidic C−H hydrogen could accept the hydrogen atom to engage an inter or intramolecular hydrogen bonding. This inter or intramolecular hydrogen bonding is purely a non‐covalent interaction. Hence, the hydrogen bond distance (2.4–3.5 Å) between the hydrogen bond donor atom and the hydrogen bond acceptor atom is longer than the covalent bond distance (1–2 Å). Unlike OH, NH, and SH hydrogen bond donors, the C−H hydrogen bonds are considered to be weak hydrogen bond donors. Therefore, chemical transformations involving such C−H hydrogen bonds are barely studied. Yet, these weak C−H hydrogen bond interactions were considered extensively in drug discovery and medicinal chemistry to understand the structural and conformational geometries of proteins and enzymes.[^15^, ^16^]

With these insights, we hypothesized that a weak C−H hydrogen atom is surrounded by an electron‐withdrawing nitro and aldehyde groups; the ability of such a C−H hydrogen bond donor could increase dramatically. Thus, the role of such intramolecular C−H hydrogen bonding during the dissociation of oxaphosphetane intermediate (OPA‐INT) could influence the Wittig olefination (WO) stereoselectivity.

**Scheme 1:**
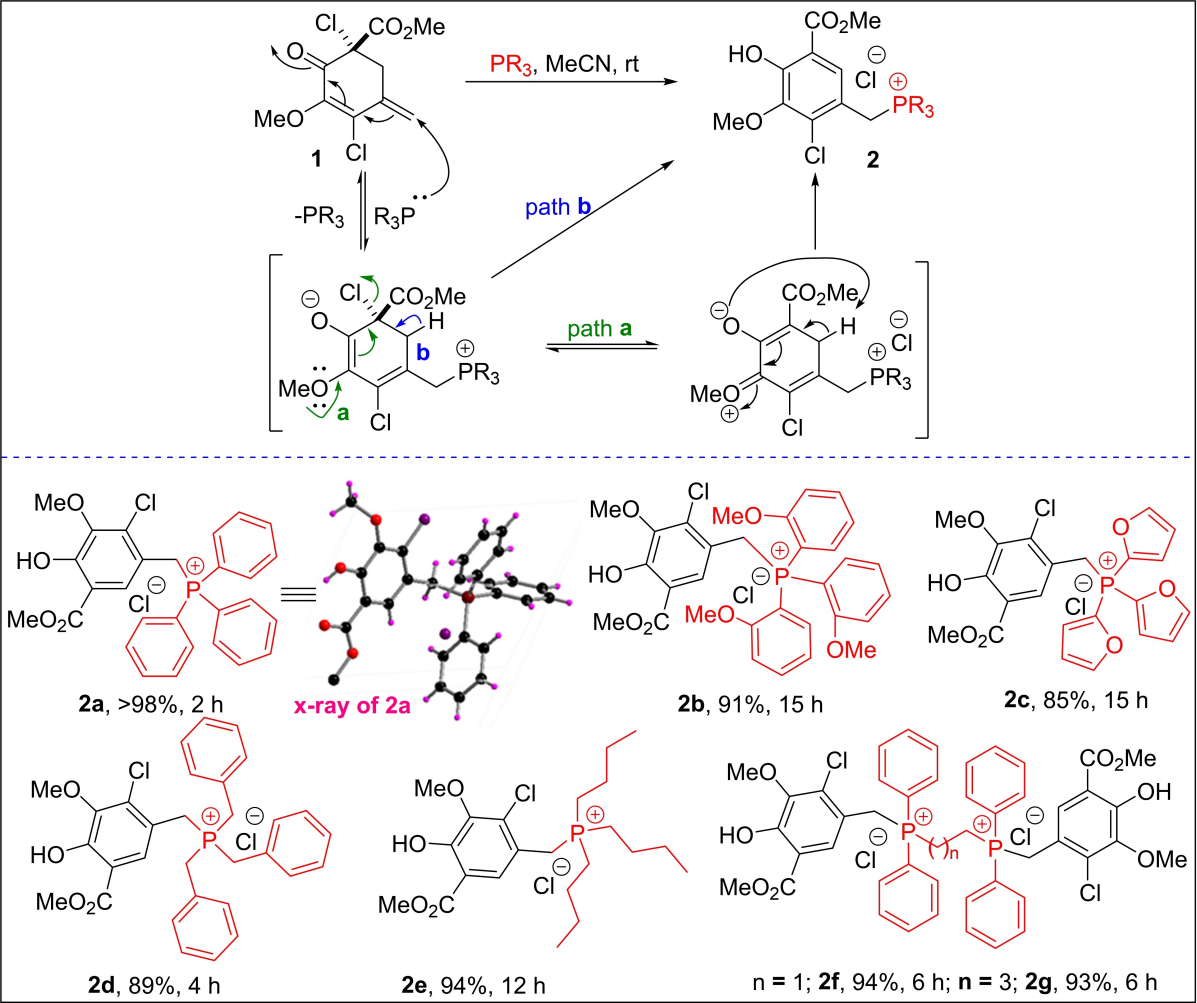
*De novo* synthesis of benzylphosphonium chloride salts **2 a**–**g**.

Therefore, we have developed a series of novel semi‐stabilized types of benzylic ylides that are generated in situ from Wittig reagents **2** under simple reaction conditions. The Wittig salts **2** were accessed in gram scale through a *de novo* 1,6‐conjugate addition of phosphorus nucleophiles to a 5‐methylidenecyclohexenone synthon **1**, as depicted in Scheme 1. Unlike traditional semi‐stabilized benzylic ylides that are recognized to result in stereoselectivity issues in Wittig olefination, the current benzylic ylides are unique. Subsequently, the reactivity and stereoselectivity of these Wittig ylides were studied with the three positional isomers of nitrobenzaldehyde. To account for the current Wittig olefination *Z* or *E*‐selectivity of *ortho/meta/para*‐nitrobenzaldehydes, the DFT studies in support of X‐ray crystal structure data of olefin products were considered. A careful computational data analysis suggested that the intramolecular CH‐hydrogen bonding with the four‐membered ring OPA‐Oxygen appears vital in obtaining the *Z/E*‐selectivity in the present WO.

## Results and Discussion

We utilized 5‐methylidenecyclohexenone **1** ^[17]^ to demonstrate the conjugate addition of phosphorus nucleophiles to generate the salicylate‐methyl‐ester‐based benzyltriphenylphosphonium chloride salts **2 a**–**g**, as described in Scheme 1.^[18]^ The representative structure of Wittig salt **2 a** was established based on the single‐crystal X‐ray analysis (See SI). Having synthesized a series of Wittig salts in multi‐gram scale quantities in high yields, we initially screened conditions for the WO of 4‐nitrobenzaldehyde with benzyltriphenylphosphonium chloride salt **2 a** under various bases, solvents, and temperatures (Table 1). Table 1 should be placed after Results and Discussion as text flows the discussion in table 1. Among the tested bases, *n*‐butyllithium (*n*‐BuLi), potassium carbonate (K_2_CO_3_), sodium hydride (NaH), potassium tert‐butoxide (*t*‐BuOK), 1,8‐diazabicyclo[5.4.0]undec‐7‐ene (DBU) and sodium methoxide (NaOMe), NaOMe in methanol appears to be the best reaction conditions in terms of yield, selectivity, and for the complete conversion of aldehyde to provide the 4’‐nitrostilbenes **3 a**, **b** in 89 % yield at room temperature for 6 h (Table 1, entry 11). Interestingly, the olefin stereoselectivity of differently designed Wittig salts **2 d**, **2 e**, and **2 f** were also consistent with Wittig salt **2 a** (Table 1). Further, the regio and diastereoselective WO of **2 d** is evidenced by the preferential reactivity of β‐hydroxy ester containing benzyl moiety in the presence of other benzyl groups on the ylidic‐phosphorus.

**Table 1:** Wittig reaction optimization.

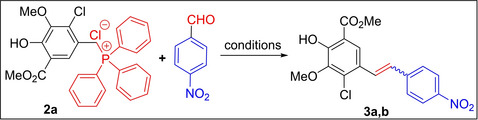						
Entry	Base/**2**	Equivalents.	Solvent	Temperature	Time	Yield of **3 a**, **b** [%] (*E/Z*)
1	K_2_CO_3_/**2 a**	3–6	THF	0 °C‐rt	24 h	no reaction
2	NaH/**2 a**	3–6	THF	0 °C‐rt	24 h	no reaction
3	DBU/**2 a**	3–6	THF	0 °C‐rt	24 h	no reaction
4	KO^*t*^Bu/**2 a**	3–6	THF	−30 °C‐rt	24 h	no reaction
5	NaHMDS/**2 a**	2.5	THF	−30 °C‐rt	8 h	30 (64 : 36)
6	NaHMDS/**2 a**	2.5	toluene	−30 °C‐rt	15 h	50 (66 : 34)
7	KHMDS/**2 a**	2.5	THF	−30 °C	10 h	45 (69 : 31)
8	LDA/**2 a**	2.5	THF	−78 °C	8 h	60 (71 : 29)
9	*n*‐BuLi/**2 a**	2.5	THF	−78 °C	5 h	78 (75 : 25)
10	LiHMDS/**2 a**	2.5	THF	−30 °C	3 h	83 (75 : 25)
**11**	**NaOMe/2 a**	**6**	**MeOH**	**30 °C**	**6 h**	**89 (79 : 21)**
12	NaOMe/**2 a**	6	THF	30 °C	6 h	72 (64 : 36)
13	NaOMe/**2 a**	6	DCM	30 °C	6 h	61 (66 : 34)
14	NaOMe/**2 a**	6	toluene	30 °C	6 h	50 (65 : 35)
15	NaOMe/**2 d**	6	MeOH	30 °C	6 h	84 (77 : 23)
16	NaOMe/**2 e**	6	MeOH	30 °C	7 d	65 (82 : 18)
17	NaOMe/**2 f**	10	MeOH	30 °C	6 h	76 (75 : 25)

**Scheme 2:**
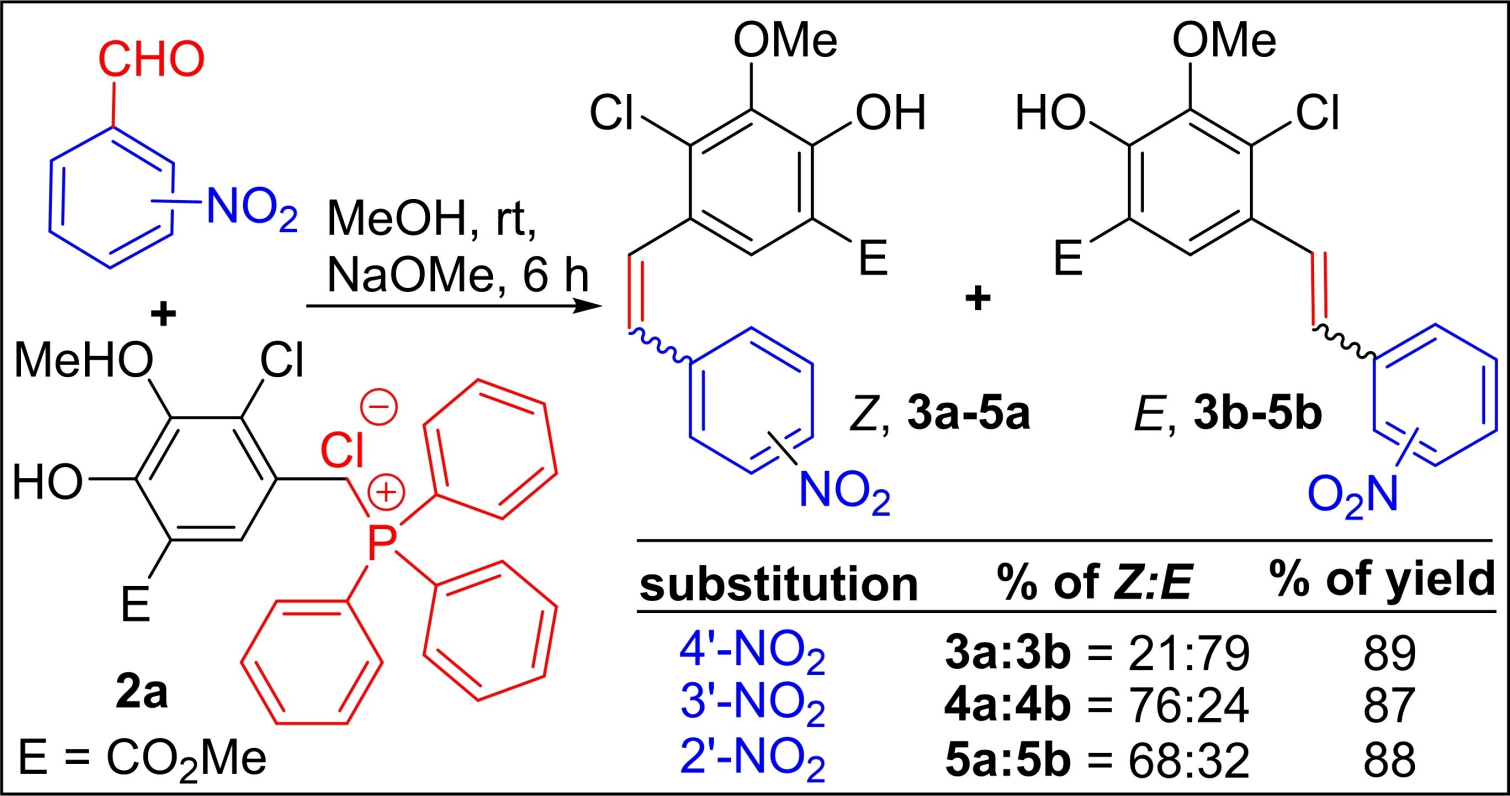
Synthesis of 2’/3’/4’‐nitro‐stilbene derivatives from **2 a**.

In contrast, the reaction of 3‐nitrobenzaldehyde with Wittig salt **2 a** under standard reaction conditions provided a mixture of stilbene products **4 a** and **4 b** with a predominate *Z*‐isomer (**4 a**) in very high yield (87 %, *Z/E*=76 : 24), as depicted in the Scheme 2.

Similarly, when **2 a** was reacted with 2‐nitrobenzaldehyde, again, the mixture of stilbene derivatives **5 a** and **5 b** was obtained in favour of *Z*‐isomer (**5 a**, *Z/E*=68 : 32) in 88 % yield. From these experiments, it is evident that the current Wittig ylides stereoselectivity with nitrobenzaldehydes is quite unique when compared with literature reports.

**Scheme 3:**
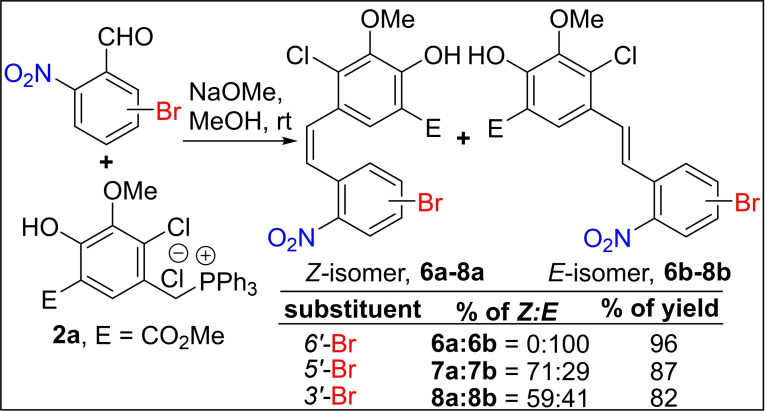
Synthesis of 3’/5’/6’‐bromo‐2’‐nitro‐stilbene derivatives.

To discern the substitution effect at C‐3, C‐5, and C‐6 positions of 2‐nitrobenzaldehyde on Wittig olefination stereoselectivity, we tested first the disubstituted nitrobenzaldehyde, 2‐nitro‐6‐bromobenzaldehyde with Wittig salt **2 a** under standard Wittig reaction conditions. Surprisingly, the 2‘‐nitro‐6‘‐bromostilbene derivative **6 b** was observed with exclusive *E*‐selectivity (*Z/E*=0 : 100) in 96 % yield, as depicted in Scheme 3. Whereas, in the case of 2‐nitro‐5‐bromobenzaldehyde and 2‐nitro‐3‐bromobenzaldehydes, we isolated a mixture of stilbene derivatives **7 a**, **b** (87 %, *Z/E*=71 : 29) and **8 a**, **b** (82 %, *Z/E*=59 : 41) with major *Z*‐isomer in high yields (Scheme 3).

These results show a dramatic shift in the stereoselectivity toward *E*‐isomer concerning the 2‐nitro‐6‐bromobenzaldehyde compared with 2‐nitrobenzaldehyde. On the other hand, the 2‐nitro‐3‐bromobenzaldehyde and 2‐nitro‐5‐bromobenzaldehydes followed the *Z*‐selectivity of 2‐nitrobenzaldehyde.

**Scheme 4:**
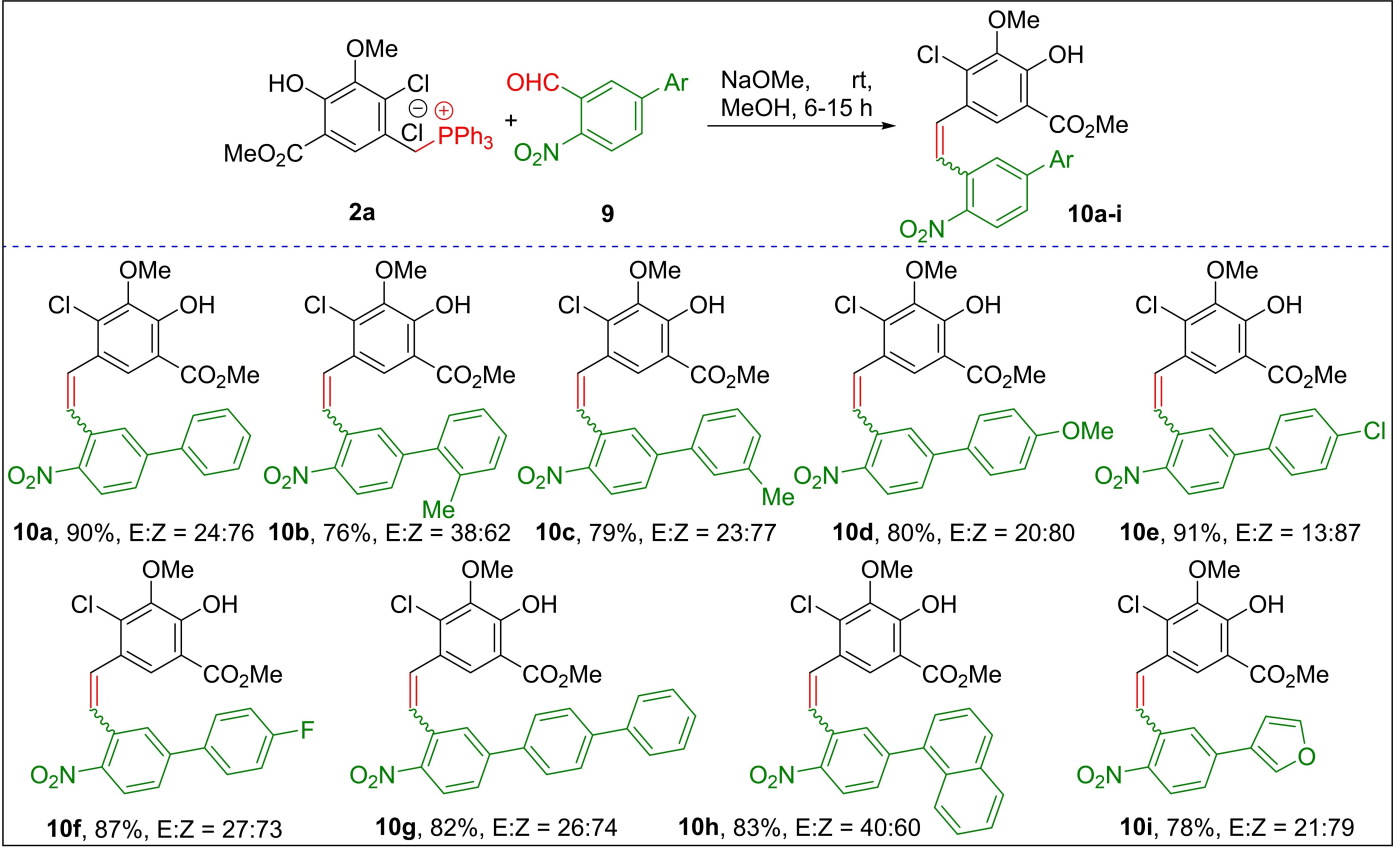
Synthesis of 5’‐aryl‐2’‐nitro‐stilbene derivatives.

To further understand the influence of sterically bulkier substituent groups at the C5‐position of the 2‐nitrobenzaldehyde on olefin *Z*‐selectivity, the 2‐nitro‐5‐bromobenzaldehyde was functionalized with various aryl‐boronic acids to obtain a series of 5‐aryl‐2‐nitrobenzaldehydes **9 a**–**i** (see SI) via palladium‐catalyzed Suzuki coupling.^[19]^ Gratifyingly, all the 5‐aryl‐2‐nitrobenzaldehydes **9 a**–**i** reacted smoothly with Wittig salt **2 a** to furnish the corresponding *Z*‐selective stilbenoids **10 a**–**i** (76–91 %, *Z/E*=60 : 40 to 87 : 13) in high yields under standard reaction conditions (Scheme 4). Among the 5‐aryl‐2‐nitrobenzaldehydes, the best *Z‐*selectivity was noticed when the 4‐chlorophenyl group (91 %, *Z/E*=87 : 13) was placed at the C‐5 position. These results indicated that the aryl substitution at the C5‐position of the 2‐nitrobenzaldehyde did not alter the olefin *Z*‐selectivity, as shown in Scheme 4. The above experimental results show that the current WO neither follows the ancillary phosphorous coordination[^11^, ^12^] nor the steric influence on the olefin stereoselectivity.^[13]^

**Scheme 5:**
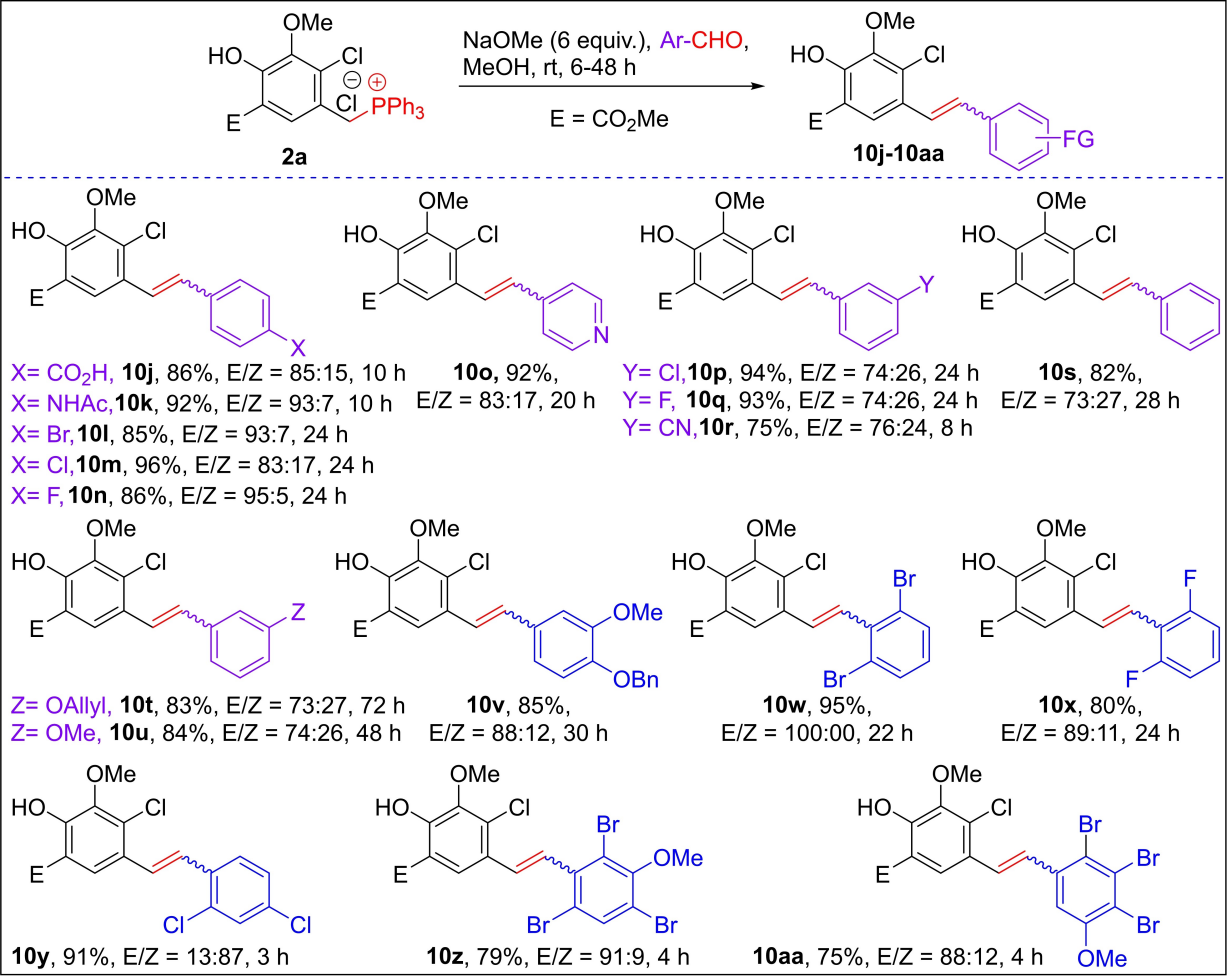
Reactivity of multi‐substituted aromatic aldehydes with Wittig salt **2 a**.

Further, the treatment of current Wittig ylides with mono, di, and tetra‐substituted aromatic aldehydes and hetero‐aromatic aldehydes without nitro group on the aromatic ring provided the corresponding stilbene derivatives **10 j**–**10 aa** in high yields (75–96 %) with high *E*‐selectivity (*E/Z*=73 : 27 to 100 : 0) as shown in Scheme 5. Interestingly, in the reaction of 2,4‐dichlorobenzaldehyde with Wittig salt **2 a**, we observed the *Z*‐selective stilbene derivative **10 y** (*E/Z*=13 : 87) in a 91 % yield.^[20]^

To gain evidence for the possible role of intramolecular C−H hydrogen bonding in OPA‐INT of WO, we have conducted the gas phase density functional theory (DFT) calculations at the B3LYP‐D3/6‐31G* level of theory using Spartan18.^[21]^ We performed the DFT calculations using equilibrium geometry, and our computations are based on ground state minima. The *Z/E*‐selectivity of Wittig olefination experimental results of all the studied nitrobenzaldehydes with current Wittig ylides correlate well with the DFT computed intramolecular CH‐hydrogen bond distances of OPA intermediates (Table S1, Supporting Information).

In addition, the vibrational frequencies of selected **OPA‐Int** of **5 a‐cis**, **5 b‐trans, 6 a‐cis, 6 b‐trans**, **10 e‐cis**, and **10 e‐trans** (SI) using B3LYP‐D3 with a basis set 6‐31G* at 298.15 K and 1.00 atm shown to be zero imaginary frequencies. Indeed, these results evidenced the electronic energy optimizations supporting the observed intramolecular hydrogen bonding and proved that the current equilibrium geometry optimizations are of OPA‐Intermediates with ground state minima but not the OPA‐transition states. Nevertheless, our extensive literature survey revealed that the characteristic features of all the reported ylide types leading to *Z*‐olefine products are traced to be of kinetic control.^[12a]^ However, the current ylides Wittig olefination stereoselectivity (Table S2, Supporting Information) appears to follow the thermodynamic control of OPA‐intermediate. Moreover, intramolecular hydrogen bonding (HB) distances play a significant role in the stability of the OPA‐intermediate. Based on the observed Wittig olefination stereoselectivity of the current ylides, we assume that the dissociation rate constants of *cis*‐OPA‐Int and *trans*‐OPA‐Int are approximately equal; hence, product outcomes follow the equilibrium constant of OPA‐Intermediates.

**Figure 2:**
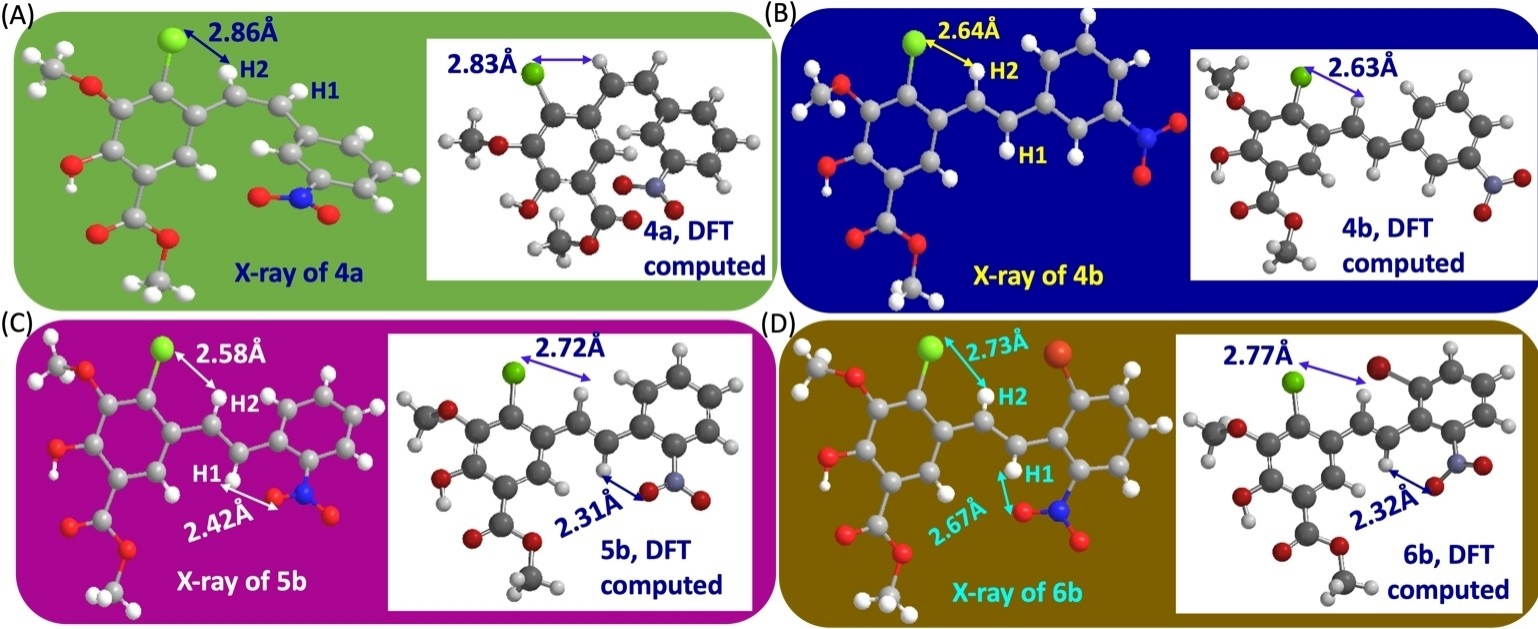
Intramolecular CH‐hydrogen bond distances of X‐ray and DFT computed stilbenes (A) **4 a‐cis**; (B) **4 b‐trans**; (C) **5 b‐trans**; (D) **6 b‐trans**.

Further, the DFT calculated intramolecular CH1 and CH2 hydrogen bonding distances with Cl or NO_2_ groups of stilbenes **4 a**, **4 b**, **5 b**, and **6 b** showed excellent correlation with the X‐ray measured intramolecular CH‐hydrogen bond distances as depicted in Figure 2A–D.

**Scheme 6:**
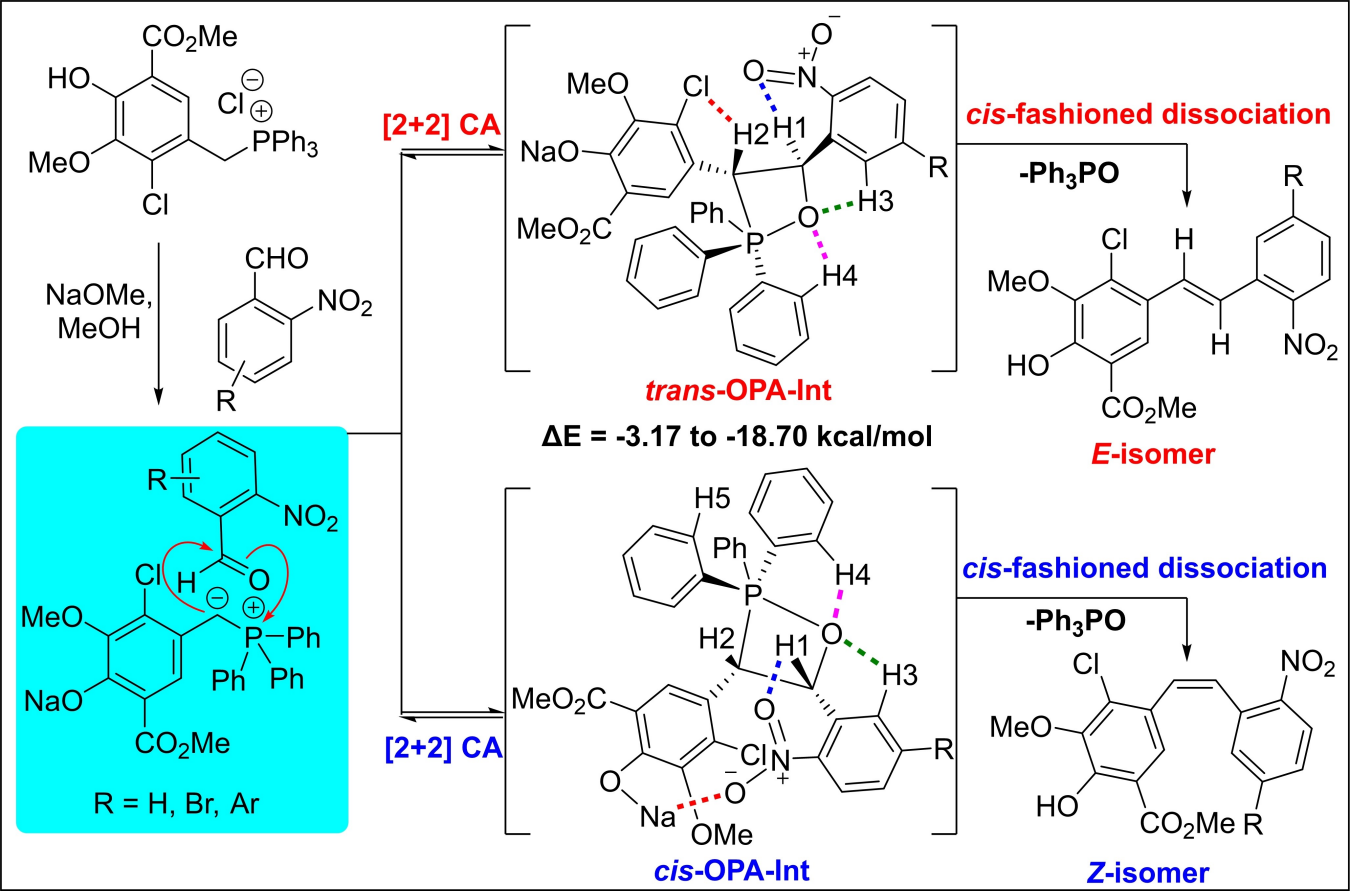
A plausible mechanism for the *Z*‐selective formation of 5‘‐Br(H/Ar)‐2‘‐nitrostilbenes.

Based on the observed experimental results and the supporting DFT calculations, we propose that the current Wittig olefination proceeds through a general [2+2] cycloaddition leading to the formation of a four‐membered ring oxaphosphetane intermediate (OPA‐Int), wherein both *cis* and *trans* conformational geometries are possible. An intramolecular CH‐hydrogen bonding between the CH hydrogen donors and the hydrogen acceptors such as oxygen, halogen, and sodium metal(Na) coordination with nitro group oxygen is critical for the stable OPA‐Int. However, the shortest intramolecular CH‐hydrogen bonding distance between the four‐membered ring OPA‐Oxygen and the CH4/CH5‐hydrogen of the triphenylphosphine phenyl ring provides good evidence for the obtained olefin *E/Z*‐stereoselectivity of the studied examples in the current Wittig olefination (Table S1, Supporting Information), as this CH‐hydrogen bond facilitates the *cis*‐fashioned dissociation of *cis* or *trans*‐OPA‐Int leading to the formation of triphenylphosphine oxide (Ph_3_P=O) and *Z* or *E*‐nitrostilbenes as depicted in the Scheme 6.

**Scheme 7:**
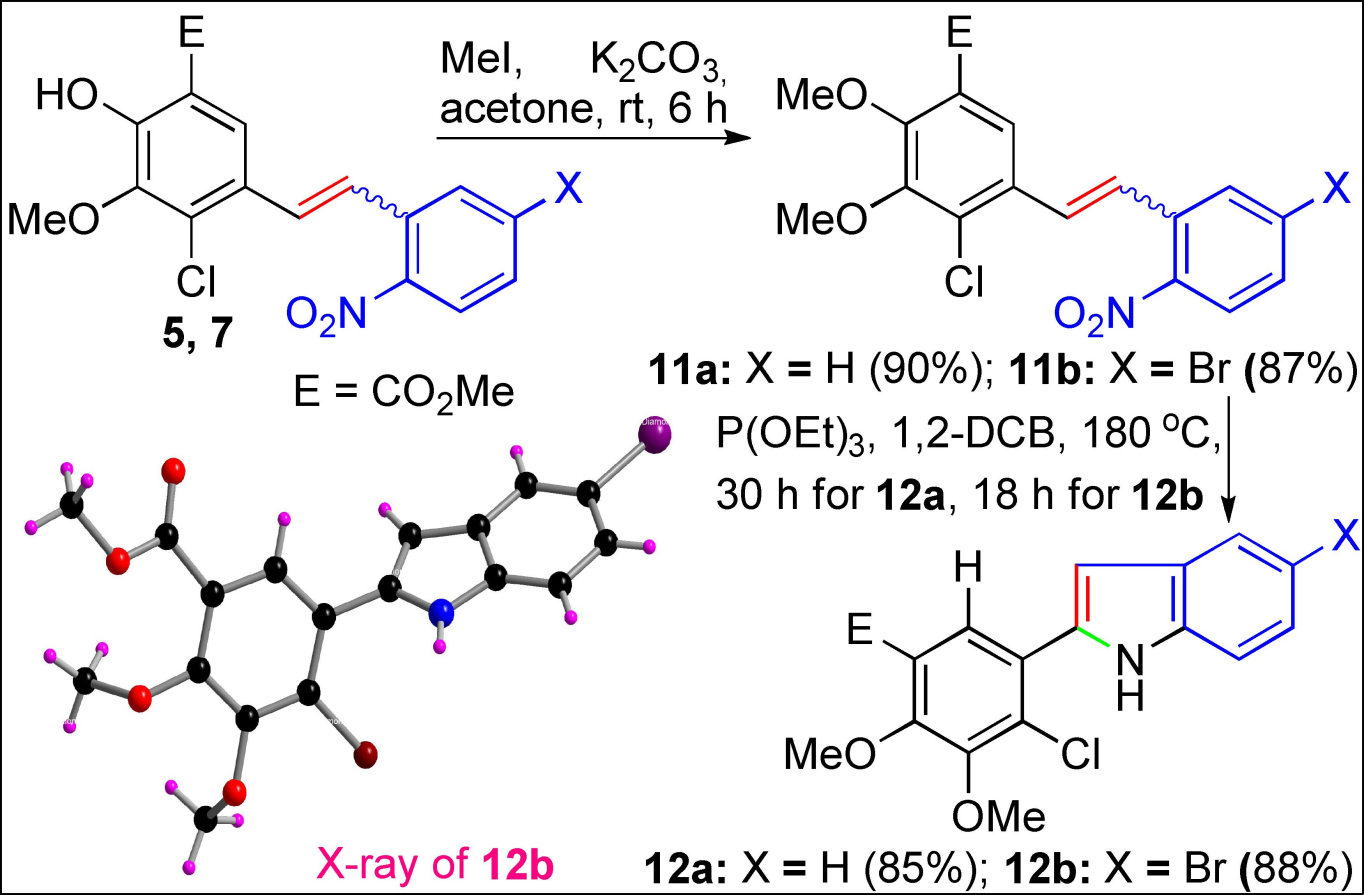
Synthesis of 2‐aryl indole derivatives **12 a** and **12 b**.

Finally, we utilized the present WO methodology to transform the 2’‐nitrostilbenoids **5** and **7** into 2‐arylindole derivatives **12 a** and **12 b** in 85–88 % yields by employing the Cadogan–Sundberg reductive cyclization (CSRC)^[22]^ as shown in Scheme 7. For the successful CSRC, methylation^[23]^ of the phenolic group in 2‘‐nitrostilbenoids **5** and **7** leading to the formation of **11 a** and **11 b** is essential. The X‐ray crystallographic data confirmed the molecular structure of 5‐bromo‐2‐arylindole derivative **12 b** (see SI).

## Conclusions

The current Wittig olefination (WO) proceeds through a reversible [2+2] cycloaddition of aldehyde and Wittig ylide, leading to the formation of an oxaphosphetane intermediate (OPA‐Int) in the first step. The subsequent *cis*‐fashioned dissociation of OPA‐Int would lead to triphenylphosphine oxide (Ph_3_P=O) and E/Z‐olefin formation. The formation of stable Ph_3_P=O is pivotal for the dissociation of OPA‐Int to deliver the C−C double bond in WO. However, the factors that governs the formation of Ph_3_P=O is the extreme stability of the P(V)=O bond has yet to be fully realized. In the current work, for the first time, we addressed an intrinsic role of intramolecular hydrogen bonding between OPA‐Oxygen, and the CH‐hydrogen of the triphenylphosphine phenyl rings that drives the dissociation step in Wittig olefination leading to the formation of olefin and the thermodynamically stable triphenylphosphine oxide. The DFT computed and X‐ray measured intramolecular CH hydrogen bonding distances strongly support the observed phenomenon. Further, the current strategy successfully transformed the 2’‐nitrostilbenoids into salicylate‐methyl‐ester‐based 2‐arylindole derivatives via Cadogan‐Sundberg reductive cyclization with 85–88 % yields.

## Supporting Information Summary

The authors have cited additional references within the Supporting Information.[^24^, ^25^, ^26^, ^27^, ^28^, ^29^]

Deposition Numbers 2015815 (for **2 a**), 2209207 (for **4 a**), 2209208 (for **4 b**), 2209205 (for **5 b**), 2209206 (for **6 b**), and 2015813 (for **12 b**) contain the supplementary crystallographic data for this paper. These data are provided free of charge by the joint Cambridge Crystallographic Data Centre and Fachinformationszentrum Karlsruhe Access Structures service. For general information, all experimental and analytical details see Supporting Information.

## Experimental Section

**Typical experimental procedure for the synthesis of benzylidenetriphenylphosphonium chloride salts 2 (GP‐1), (2‐chloro‐4‐hydroxy‐3‐methoxy‐5‐(methoxycarbonyl)benzyl)triphenylphosphonium chloride (2 a)**: To a stirred solution of cyclohexadienone **1** (13 g, 49.04 mmol), in acetonitrile (20 mL) was added triphenylphosphine (15.44 g, 58.86 mmol) at room temperature. The reaction mixture was allowed to stir for 2–15 h at the same temperature, and the reaction was monitored by TLC. After consumption of starting material, the white solids were filtered and washed with an excess of n‐hexanes to obtain the free powder **2 a** in excellent yield (25.5 g, >98 %), which upon **2 a** was placed in a desiccator and used for Wittig olefination; M. p. 158–161 °C; ^1^H NMR (400 MHz, CDCl_3_) *δ*: 11.02 (s, 1H), 7.79–7.67 (m, 10H), 7.64–7.58 (m, 6H), 5.72 (d, *J*=13.7 Hz, 2H), 3.77 (s, 3H), 3.69 (s, 3H); ^13^C NMR (100 MHz, CDCl_3_) *δ*: 169.64, 155.68, 155.25, 145.22, 136.33, 136.27, 135.10, 135.07, 134.30, 134.21, 130.30, 130.18, 128.19, 128.14, 118.03, 117.18, 116.87, 116.79, 112.43, 112.40, 60.45, 52.79, 27.83, 27.35.^31^P NMR (162 MHz, CDCl_3_) *δ*: 22.34; IR ν_max_ (Neat) 3125, 2953, 2876, 2108, 1604, 1540, 1397, 1208, 1165, 1024, 980 cm^−1^; HRMS‐ESI (*m/z*): [M]^+^ calcd for C_28_H_25_ClO_4_P^+^, 491.1173; found, 491.1171.

**(2‐chloro‐4‐hydroxy‐3‐methoxy‐5‐(methoxycarbonyl)benzyl)bis(2‐methoxyphenyl)(3‐methoxyphenyl)phosphoniumchloride (2 b)**: Compound **2 b** (112 mg, 91 %) has been prepared by following a similar experimental procedure of **GP‐1**, from cyclohexadienone **1** (53 mg, 0.2 mmol) and tris‐2‐methoxyphenyl phosphine (84.6 mg, 0.24 mmol); M. p. 190–195 °C; ^1^H NMR (400 MHz, DMSO‐*d*_6_) δ 10.52 (s, 1H), 7.83 (tt, *J*=7.2, 1.5 Hz, 3H), 7.36–7.27 (m, 7H), 7.22 (td, *J*=7.6, 2.9 Hz, 3H), 4.88 (d, *J*=16.0 Hz, 2H), 3.80 (s, 3H), 3.70 (s, 3H), 3.61 (s, 9H); ^13^C NMR (101 MHz, DMSO‐*d*_6_) δ 168.05, 161.62 (d, *J*=2.6 Hz), 153.44 (d, *J*=2.9 Hz), 145.04 (d, *J*=2.4 Hz), 137.69 (d, *J*=2.3 Hz), 135.48 (d, *J*=8.5 Hz), 134.55 (d, *J*=7.0 Hz), 133.33, 130.75, 127.22 (d, *J*=6.3 Hz), 122.31 (d, *J*=12.7 Hz), 121.28 (d, *J*=10.0, 113.46 (d, *J*=6.7 Hz), 113.20 (d, *J*=3.2 Hz), 105.34 (d, *J*=91.3 Hz), 60.66, 56.53, 27.54 (d, *J*=53.6 Hz); ^31^P NMR (162 MHz, DMSO‐*d*_6_) δ 24.77; IR ν_max_ (Neat) 3627, 2947, 2838, 1685, 1592, 1576, 1482, cm^−1^; HRMS‐ESI (*m/z*): [M]^+^ calcd for C_31_H_31_ClO_7_P^+^, 581.1490; found, 581.1485.

**(2‐chloro‐4‐hydroxy‐3‐methoxy‐5‐(methoxycarbonyl)benzyl)tri(furan‐2‐yl)phosphonium chloride (2 c)**: Compound **2 c** (85 mg, 85 %) has been prepared by following a similar experimental procedure of **GP‐1**, from cyclohexadienone **1** (53 mg, 0.2 mmol) and tris‐2‐furylphosphine (56 mg, 0.48 mmol); M. p. 105–106 °C; ^1^H NMR (400 MHz, Chloroform‐*d*) δ 11.08 (s, 1H), 8.04 (dd, *J*=3.8, 1.9 Hz, 3H), 7.91 (dd, *J*=2.9, 1.6 Hz, 3H), 7.71 (d, *J*=3.8 Hz, 1H), 6.74 (dt, *J*=3.8, 1.9 Hz, 3H), 5.57 (d, *J*=14.9 Hz, 2H), 3.85 (s, 3H), 3.75 (s, 3H); ^13^C NMR (101 MHz, Chloroform‐*d*) δ 169.75, 156.07 (d, *J*=3.7 Hz), 153.23 (d, *J*=8.6 Hz), 145.33, 135.45 (d, *J*=6.0 Hz), 132.27 (d, *J*=22.1 Hz), 130.01 (d, *J*=139.4 Hz), 128.46 (d, *J*=6.7 Hz), 115.39 (d, *J*=9.9 Hz), 113.62 (d, *J*=10.1 Hz), 112.47 (d, *J*=4.2 Hz), 52.99, 29.11 (d, *J*=54.9 Hz); ^31^P NMR (162 MHz, Chloroform‐*d*) δ −15.18; IR ν_max_ (Neat) 3679, 3045, 2985, 1685, 1610, 1551, 1446 cm^−1^; HRMS‐ESI (*m/z*): [M]^+^ calcd for C_22_H_19_ClO_7_P^+^, 461.0551; found, 461.0548.

**Tribenzyl(2‐chloro‐4‐hydroxy‐3‐methoxy‐5‐(methoxycarbonyl)benzyl)phosphonium chloride (2 d)**: Compound **2 d** (243 mg, 89 %) has been prepared by following a similar experimental procedure of **GP‐1**, from cyclohexadienone **1** (106 mg, 0.4 mmol) and tris‐benzylphosphine (146 mg, 0.48 mmol); M. p. 78–80 °C; ^1^H NMR (400 MHz, Chloroform‐*d*) δ 11.05 (s, 1H), 7.31–7.21 (m, 10H), 7.20‐7.11 (m, 6H), 4.09 (d, *J*=14.7 Hz, 6H), 4.05 (d, *J*=13.7 Hz, 2H), 3.88 (s, 3H), 3.82 (s, 3H); ^13^C NMR (101 MHz, Chloroform‐*d*) δ 169.40, 155.84 (d, *J*=2.7 Hz), 145.82, 134.59 (d, *J*=4.9 Hz), 130.63 (d, *J*=5.4 Hz), 129.49 (d, *J*=2.9 Hz), 128.61 (d, *J*=3.6 Hz), 127.46 (d, *J*=8.3 Hz), 126.70 (d, *J*=5.0 Hz), 117.64 (d, *J*=7.9 Hz), 112.60 (d, *J*=2.7 Hz), 60.66, 27.34 (d, *J*=42.0 Hz), 23.57 (d, *J*=44.7 Hz); ^31^P NMR (162 MHz, Chloroform‐*d*) δ 25.29; IR ν_max_ (Neat) 3657, 3037, 2961, 1688, 1603, 1499, 1458, 1348 cm^−1^; HRMS‐ESI (*m/z*): [M]^+^ calcd for C_31_H_31_ClO_4_P^+^, 533.1643; found, 533.1640.

**Tributyl(2‐chloro‐4‐hydroxy‐3‐methoxy‐5‐(methoxycarbonyl)benzyl)phosphonium chloride (2 e)**: Compound **2 e** (162 mg, 94 %) has been prepared by following a similar experimental procedure of **GP‐1**, from cyclohexadienone **1** (106 mg, 0.4 mmol) and tris‐*n*‐butylphosphine (97 mg, 0.48 mmol); M. p. 85–87 °C; ^1^H NMR (300 MHz, Chloroform‐*d*) δ 11.10 (s, 1H), 7.96 (s, 1H), 4.37 (d, *J*=14.9 Hz, 2H), 3.90 (d, *J*=13.6 Hz, 6H), 2.41 (dq, *J*=16.1, 6.5, 5.7 Hz, 6H), 1.41 (dq, *J*=10.7, 7.0, 5.3 Hz, 12H), 0.94‐0.80 (m, 9H); ^13^C NMR (75 MHz, Chloroform‐*d*) δ 169.64, 155.62 (d, *J*=2.8 Hz), 145.59 (d, *J*=2.7 Hz), 134.04 (d, *J*=4.8 Hz), 127.35 (d, *J*=4.8 Hz), 118.23 (d, *J*=8.8 Hz), 112.68 (d, *J*=3.0 Hz), 60.67, 53.06, 24.41 (d, *J*=45.9 Hz), 23.92 (d, *J*=15.4 Hz), 23.62 (d, *J*=4.9 Hz), 18.96 (d, *J*=45.9 Hz), 13.36; ^31^P NMR (121 MHz, Chloroform‐*d*) δ 33.28; IR ν_max_ (Neat) 3665, 3067, 2985, 1685, 1605, 1459, 1346 cm^−1^; HRMS‐ESI (*m/z*): [M]^+^ calcd for C_22_H_37_ClO_4_P^+^, 431.2113; found, 431.2109.

**Ethane‐1,2‐diylbis((2‐chloro‐4‐hydroxy‐3‐methoxy‐5‐(methoxycarbonyl)benzyl)diphenylphosphonium) chloride (2 f)**: Compound **2 f** (167 mg, 94 %) has been prepared by following a similar experimental procedure of **GP‐1**, from cyclohexadienone **1** (100 mg, 0.377 mmol) and 1,2‐bis(diphenylphosphino)ethane (76 mg, 0.188 mmol); M. p. 197–200 °C; ^1^H NMR (400 MHz, DMSO‐*d_6_*) *δ*: 10.66 (s, 2H), 7.93–7.88 (m, 3H), 7.77–7.67 (m, 18H), 7.37 (s, 1H), 4.88 (t, *J*=6.7 Hz, 4H), 3.77 (s, 6H), 3.61 (s, 6H), 3.48 (s, 4H); ^13^C NMR (100 MHz, CDCl_3_) *δ*: 169.52, 155.69, 145.39, 135.76, 135.30, 135.13, 134.78, 134.73, 134.68, 134.00, 133.91, 132.38, 131.51, 131.13, 131.03, 130.77, 130.26, 130.20, 130.14, 129.16, 129.04, 128.84, 127.44, 116.68, 115.55, 115.15, 115.11, 114.73, 112.21, 79.24, 60.06, 52.65, 26.32, 26.07, 25.83, 14.37, 14.12, 13.88; ^31^P NMR (162 MHz, CDCl_3_) *δ*: 28.50; IR ν_max_ (Neat) 3313, 3054, 2943, 2779, 1671, 1601, 1443, 1337, 1256, 1160, 1047, 919 cm^−1^; HRMS‐ESI (*m/z*): [M]^+^ calcd for C_23_H_22_ClO_4_P^2+^, 428.0939; found, 428.0939.

**Butane‐1,4‐diylbis((2‐chloro‐4‐hydroxy‐3‐methoxy‐5‐(methoxycarbonyl)benzyl)diphenylphosphonium) dichloride (2 g)**: Compound **2 g** (170 mg, 93 %) has been prepared by following a similar experimental procedure of **GP‐1**, from cyclohexadienone **1** (100 mg, 0.377 mmol) and 1,4‐bis(diphenylphosphino)butane (81 mg, 0.188 mmol); M. p. 193–196 °C; ^1^H NMR (400 MHz, CDCl_3_) *δ*: 10.03 (br, 2H), 7.84–7.66 (m, 12H), 7.62–7.59 (m, 6H), 7.49‐7.43 (m, 4H), 4.94 (d, *J*=13.8 Hz, 4H), 4.80 (br, 4H), 3.82 (s, 6H), 3.71 (s, 6H), 3.62‐3.52 (m, 4H); ^13^C NMR (100 MHz, DMSO‐*d_6_*) *δ*: 169.45, 155.50, 150.52, 145.30, 134.83, 134.10, 134.01, 130.18, 130.06, 128.84, 117.21, 116.77, 115.95, 112.18, 60.43, 52.79, 27.79, 22.48, 21.38, 20.90; ^31^P NMR (162 MHz, CDCl_3_) *δ*: 28.51; IR ν_max_ (Neat) 3365, 2943, 2934, 1669, 1605, 1441, 1342, 1249, 1154, 1113, 1052, 953 cm^−1^; HRMS‐ESI (*m/z*): [M]^+^ calcd for C_24_H_24_ClO_4_P^+^, 442.1095; found, 442.1095.

## Author Contributions

The manuscript was written through the contributions of all authors. All authors have approved the final version of the manuscript.

## Conflict of interests

The authors declare no conflict of interest.

1

## Supporting information

As a service to our authors and readers, this journal provides supporting information supplied by the authors. Such materials are peer reviewed and may be re‐organized for online delivery, but are not copy‐edited or typeset. Technical support issues arising from supporting information (other than missing files) should be addressed to the authors.

Supporting Information

## Data Availability

The data that support the findings of this study are available in the supplementary material of this article.
