# Supplementary material for: Intramolecular CH‐Hydrogen Bonding During the Dissociation of the Oxaphosphetane Intermediate Facilitates Z/E‐Selectivity in Wittig Olefination
Source: ChemistryOpen. 2023 Dec 7;13(3):e202300171. doi: 10.1002/open.202300171 (PMC10924042; doi:10.1002/open.202300171)
Supplement: Supplementary file 1 — Supporting Information [file OPEN-13-e202300171-s001.pdf]

ChemistryOpen

Supporting Information

# Intramolecular CH-Hydrogen Bonding During the Dissociation of the Oxaphosphetane Intermediate Facilitates *Z/E*-Selectivity in Wittig Olefination

Kukkamudi Sreenivas, Chintada Nageswara Rao,* and Faiz Ahmed Khan*

**Table of Contents**


| S. No. | Contents | Page. No |
|---|---|---|
| 1 | Table 1: DFT calculated intramolecular CH4/CH5 hydrogen bond distances with OPA-oxygen and experimentally observed olefin selectivity. | S2 |
| 2 | **Figure 1.** DFT calculated intramolecular hydrogen bond distances: (a) *cis*-OPA-Int of **3a**; (b) *trans*-OPA-Int of **3b;** (c) *cis*-OPA-Int of **4a**; (d) *trans*-OPA-Int of **4b;** (e) *cis*-OPA-Int of **5a**; (f) *trans*-OPA-Int of **5b;** (g) *cis*-OPA-Int of **6a**; (h) *trans*-OPA-Int of **6b**. | S3 |
| 3 | Table 2: Characteristic features of the Wittig ylides and the comparison with current ylides. | S4 |
| 4 | DFT calculated vibrational frequencies of **5a-*cis*, 5b-*trans*, 6a-*cis*, 6b-*trans*, 10e-*cis*, and 10e-*trans* OPA-Int**. | S4-S16 |
| 5 | General Methods. | S16 |
| 6 | Experimental procedures and spectral data of all new compounds. | S17-S49 |
| 7 | Crystal data for compounds **2a, 4a, 4b, 5b, 6b** and **12b**. | S50-S90 |
| 8 | Copies of $^{1}H$ and $^{13}C$ NMR spectra of new compounds. | S90-S152 |
| 9 | References. | S153 |

**Table 1:** ***DFT calculated intramolecular CH4/CH5 hydrogen bond distances with OPA-oxygen and experimentally observed olefin selectivity.***

| S. No. | DFT computed structure | ΔE = $E_{trans}$-$E_{cis}$ | CH4/CH5-Hydrogen bond distance (Å)[a] with OPA-Oxygen | | Experimentally observed olefin selectivity | S. No. | DFT computed structure | ΔE = $E_{trans}$-$E_{cis}$ | CH4/CH5-Hydrogen bond distance (Å)[a] with OPA-Oxygen | | Experimentally observed olefin selectivity |
|---|---|---|---|---|---|---|---|---|---|---|---|
| | OPA-INT | in kcal/mol | *cis* | *trans* | *Z/E* | | OPA-INT | in kcal/mol | *cis* | *trans* | *Z/E* |
| 1 | 3a/3b | -12.37 | 2.37 | 2.29 | 21/79 | 12 | 10g | 7.37 | 2.20 | 2.38 | 74/26 |
| 2 | 4a/4b | 11.84 | 2.13 | 2.25 | 76/24 | 13 | 10h[b] | 16.65 | 2.69 | 2.82 | 60/40 |
| 3 | 5a/5b | 3.17 | 2.21 | 2.26 | 68/32 | 14 | 10i | 16.32 | 2.18 | 2.26 | 79/21 |
| 4 | 6a/6b | -9.34 | 2.43 | 2.29 | 0/100 | 15 | 10j | -10.20 | 2.27 | 2.19 | 15/85 |
| 5 | 7a/7b[b] | 5.99 | 2.36 | 2.71 | 71/21 | 16 | 10m | -9.04 | 2.25 | 2.22 | 17/83 |
| 6 | 8a/8b[b] | 0.29 | 2.45 | 2.56 | 59/41 | 17 | 10q | -6.41 | 2.22 | 2.18 | 26/74 |
| 7 | 10a | 14.74 | 2.18 | 2.37 | 76/24 | 18 | 10s | -16.77 | 2.19 | 2.17 | 27/73 |
| 8 | 10b | 11.16 | 2.22 | 2.25 | 62/38 | 19 | 10w | -9.31 | 2.26 | 2.13 | 0/100 |
| 9 | 10d[b] | 18.70 | 2.31 | 2.63 | 80/20 | 20 | 10x[b] | -10.81 | 2.70 | 2.51 | 11/89 |
| 10 | 10e | 11.06 | 2.14 | 2.28 | 87/13 | 21 | 10y | 10.10 | 2.27 | 2.37 | 87/13 |
| 11 | 10f | 1.39 | 2.11 | 2.17 | 73/27 | 22 | 10z | -8.84 | 2.28 | 2.18 | 9/91 |

*[a]H-bond distance considered in this study was 2 to 3.4Å; [b]values represents avarage of CH4/CH5 H-bond distance*

**Figure 1.** *DFT calculated intramolecular hydrogen bond distances: (a) cis-OPA-Int of **3a**; (b) trans-OPA-Int of **3b;** (c) cis-OPA-Int of **4a**; (d) trans-OPA-Int of **4b;** (e) cis-OPA-Int of **5a**; (f) trans-OPA-Int of **5b;** (g) cis-OPA-Int of **6a;** (h) trans-OPA-Int of **6b.***

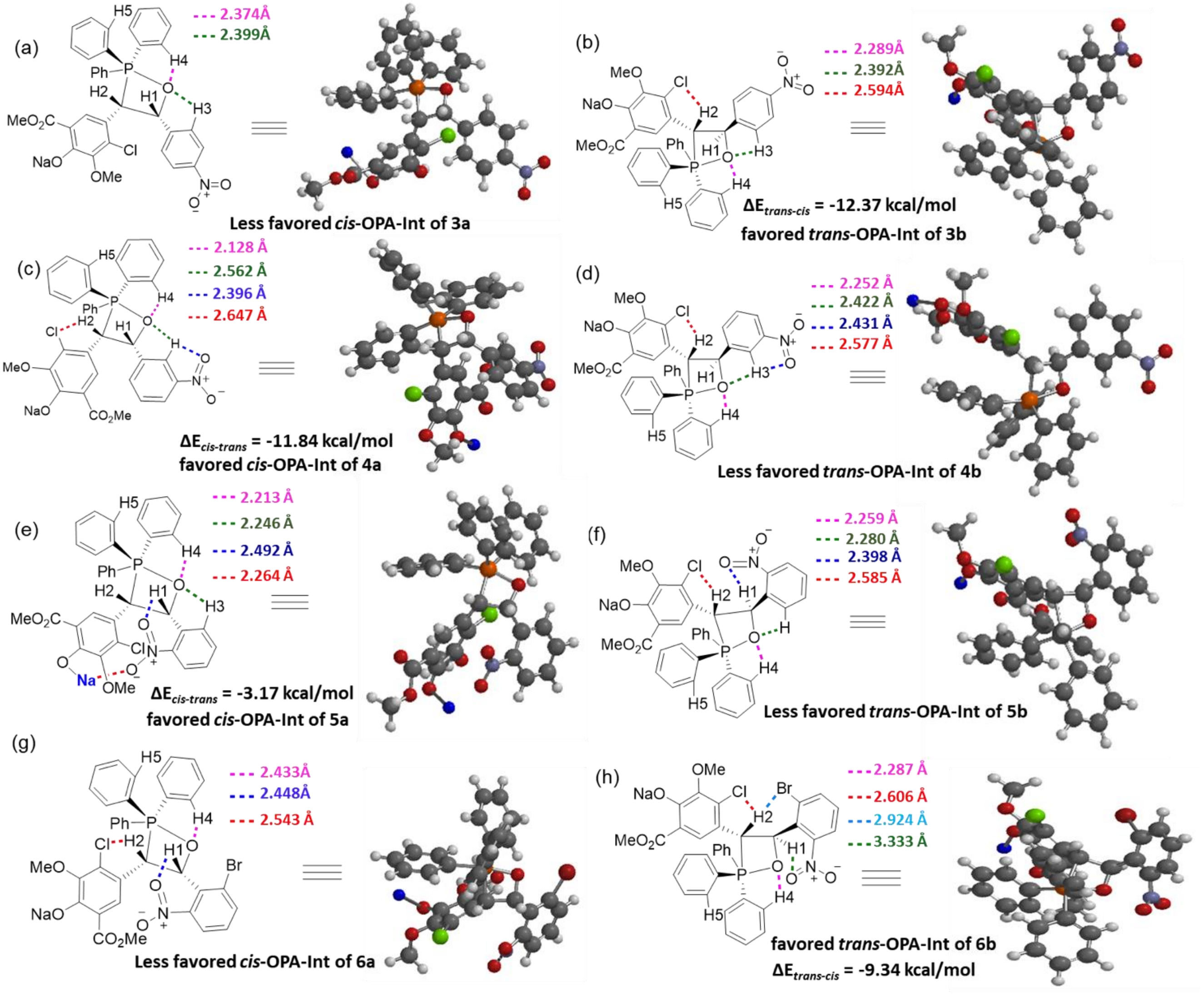

**Table 2: Characteristic features of the reported Wittig ylides and the comparison with current ylides.**

| Non-stabilized | Stabilized | Semi-stabilized | Semi-stabilized (This work) |
|---|---|---|---|
| Unstable in air | Stable in air | Stability in air depends on the P-substitution | Stable in air |
| Proceeds through the dissociation of OPA as a rate-determining step. | Proceeds through the formation of OPA as a rate-determining step. | Proceeds through the dissociation of OPA as a rate-determining step. | Proceeds through the dissociation of OPA as a rate-determining step. |
| The reaction profile is traced to kinetic control. | The reaction profile is traced to thermodynamic control. | The reaction profile is traced to kinetic control. | The reaction profile is traced to thermodynamic control. |
| The formed OPA is irreversible. | The formed OPA is reversible. | The formed OPA are irreversible. | The formed OPA is reversible. |
| *Z*-selective olefines were observed in general. | *E*-selective olefines were observed in general. | Un-selective, equal *E* and *Z* alkenes were observed. | High *E*/*Z*-selective olefines were observed. |
| The reactions are fast (second to minutes). | The reaction times are quite long (minutes to hours). | The reaction times were moderate (minute to hour). | The reaction times are quite long (hours). |
| Lower temperatures. | Harsh reaction conditions. | Mild reaction conditions. | Milder reaction conditions. |

The DFT calculations using **"equilibrium geometry"** based on "**ground state minima".** The vibrational frequencies of **OPA-Int** of **5a-cis** and **5b-trans, 6a-cis** and **6b-trans,** and **10e-cis** and **10e-trans** using B3LYP-D3 with a basis set 6-31G* at 298.15 K and 1.00 atm which has shown zero Imaginary Frequencies. And the calculated vibrational frequencies are uncorrected.

## DFT calculated vibrational frequencies.

### 5a-*cis*-OPA-Int:

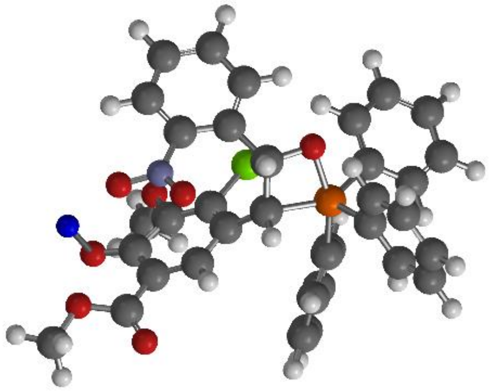

| | Uncorrected $cm^{-1}$ | Intensity |
|---|---|---|
| 61 | 538 | 67.01 |
| 62 | 541 | 71.88 |

| | | |
|---|---|---|
| 63 | 559 | 69.66 |
| 66 | 609 | 58.86 |
| 67 | 629 | 40.45 |
| 79 | 716 | 64.13 |
| 80 | 721 | 30.58 |
| 82 | 732 | 69.07 |
| 120 | 1062 | 48.08 |
| 121 | 1083 | 80.25 |
| 126 | 1120 | 103.28 |
| 130 | 1143 | 375.89 |
| 131 | 1163 | 46.31 |
| 139 | 1211 | 80.46 |
| 145 | 1254 | 92.84 |
| 148 | 1303 | 192.73 |
| 149 | 1312 | 67.46 |
| 153 | 1357 | 189.93 |
| 155 | 1368 | 52.51 |
| 159 | 1377 | 77.06 |
| 169 | 1510 | 54.89 |
| 176 | 1547 | 481.93 |
| 178 | 1593 | 141.41 |
| 183 | 1641 | 306.84 |
| 188 | 1785 | 295.24 |
| 190 | 3033 | 62.27 |

| | | |
|---|---|---|
| 191 | 3073 | 50.79 |
| 193 | 3119 | 41.22 |
| 203 | 3196 | 35.79 |
| 205 | 3204 | 47.29 |
| 206 | 3207 | 39.16 |

**5b-*trans*-OPA-Int:**

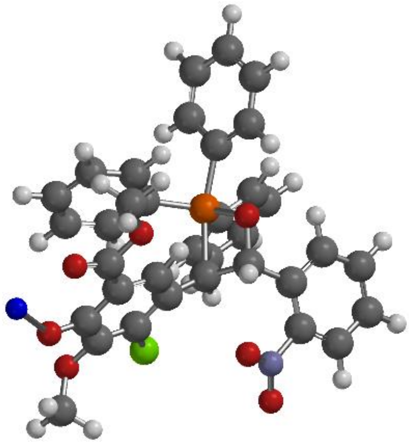

| | Uncorrected $cm^{-1}$ | Intensity |
|---|---|---|
| 59 | 508 | 32.96 |
| 60 | 519 | 38.66 |
| 61 | 537 | 56.77 |
| 62 | 554 | 55.25 |
| 70 | 654 | 41.75 |
| 73 | 687 | 31.30 |
| 79 | 718 | 59.17 |
| 80 | 725 | 55.46 |
| 81 | 731 | 61.95 |

| | | |
|---|---|---|
| 82 | 748 | 46.59 |
| 90 | 812 | 42.62 |
| 91 | 859 | 37.87 |
| 96 | 899 | 61.96 |
| 97 | 914 | 50.61 |
| 112 | 1013 | 33.12 |
| 115 | 1020 | 46.00 |
| 122 | 1103 | 40.59 |
| 125 | 1112 | 79.48 |
| 126 | 1114 | 58.92 |
| 130 | 1145 | 50.97 |
| 134 | 1184 | 52.18 |
| 140 | 1220 | 49.99 |
| 141 | 1225 | 176.33 |
| 145 | 1269 | 45.60 |
| 146 | 1286 | 366.13 |
| 147 | 1295 | 150.93 |
| 158 | 1383 | 140.69 |
| 159 | 1394 | 181.87 |
| 165 | 1486 | 78.00 |
| 167 | 1493 | 33.57 |
| 168 | 1508 | 54.38 |
| 172 | 1534 | 73.00 |
| 173 | 1534 | 151.36 |

| | | |
|---|---|---|
| 177 | 1556 | 97.86 |
| 178 | 1618 | 154.37 |
| 182 | 1637 | 48.33 |
| 187 | 1671 | 87.18 |
| 188 | 1693 | 868.94 |
| 189 | 3034 | 86.29 |
| 190 | 3059 | 51.04 |
| 191 | 3113 | 44.92 |
| 192 | 3129 | 32.02 |
| 202 | 3191 | 34.85 |
| 205 | 3203 | 44.12 |
| 207 | 3208 | 36.29 |

**6a-*cis*-OPA-Int:**

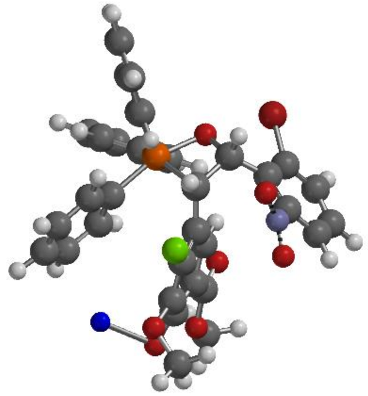

| | Uncorrected $cm^{-1}$ | Intensity |
|---|---|---|
| 65 | 545 | 150.60 |
| 66 | 556 | 99.43 |
| 68 | 606 | 46.30 |

| | | |
|---|---|---|
| 74 | 658 | 34.20 |
| 79 | 714 | 40.37 |
| 81 | 717 | 35.73 |
| 82 | 724 | 37.73 |
| 83 | 730 | 38.45 |
| 86 | 754 | 58.25 |
| 99 | 899 | 42.39 |
| 119 | 1053 | 37.08 |
| 122 | 1068 | 38.55 |
| 124 | 1090 | 60.41 |
| 127 | 1114 | 73.79 |
| 133 | 1180 | 92.49 |
| 139 | 1207 | 42.06 |
| 141 | 1213 | 82.14 |
| 142 | 1226 | 85.99 |
| 146 | 1243 | 43.33 |
| 147 | 1246 | 79.94 |
| 148 | 1269 | 241.24 |
| 149 | 1286 | 139.81 |
| 155 | 1361 | 84.95 |
| 156 | 1366 | 38.81 |
| 161 | 1395 | 190.27 |
| 167 | 1487 | 35.24 |
| 170 | 1507 | 89.92 |

| | | |
|---|---|---|
| 172 | 1523 | 50.60 |
| 173 | 1528 | 85.40 |
| 174 | 1529 | 76.79 |
| 178 | 1567 | 210.80 |
| 179 | 1607 | 91.31 |
| 181 | 1621 | 83.29 |
| 185 | 1644 | 170.32 |
| 189 | 1750 | 193.67 |
| 191 | 3055 | 62.85 |
| 192 | 3060 | 48.91 |
| 205 | 3205 | 35.00 |

**6b-*trans*-OPA-Int:**

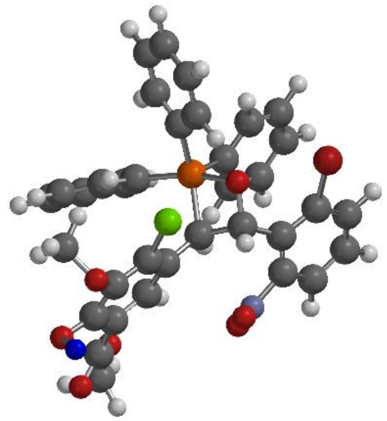

| | Uncorrected cm$^{-1}$ | Intensity |
|---|---|---|
| 63 | 526 | 55.82 |
| 65 | 545 | 197.94 |
| 66 | 566 | 19.75 |
| 69 | 608 | 34.56 |
| 79 | 707 | 42.77 |

| | | |
|---|---|---|
| 81 | 721 | 43.00 |
| 84 | 733 | 51.61 |
| 85 | 749 | 36.13 |
| 100 | 917 | 41.16 |
| 121 | 1062 | 61.13 |
| 122 | 1077 | 47.20 |
| 123 | 1085 | 71.05 |
| 124 | 1105 | 36.43 |
| 130 | 1127 | 71.98 |
| 132 | 1163 | 191.10 |
| 141 | 1219 | 59.37 |
| 142 | 1226 | 32.03 |
| 146 | 1243 | 31.10 |
| 147 | 1250 | 153.72 |
| 148 | 1270 | 66.49 |
| 149 | 1283 | 115.15 |
| 150 | 1309 | 109.93 |
| 155 | 1348 | 54.58 |
| 160 | 1394 | 32.73 |
| 161 | 1407 | 167.01 |
| 171 | 1516 | 275.98 |
| 178 | 1564 | 156.70 |
| 180 | 1625 | 102.80 |
| 184 | 1643 | 158.35 |

| | | |
|---|---|---|
| 189 | 1797 | 441.07 |
| 190 | 3046 | 45.74 |
| 191 | 3059 | 54.05 |
| 196 | 3137 | 39.65 |
| 205 | 3202 | 39.58 |
| 206 | 3205 | 60.46 |

**10e-*cis*-OPA-Int:**

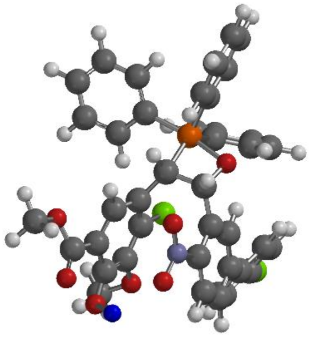

| | Uncorrected $cm^{-1}$ | Intensity |
|---|---|---|
| 64 | 464 | 48.84 |
| 65 | 471 | 45.81 |
| 70 | 522 | 39.79 |
| 72 | 538 | 43.59 |
| 85 | 691 | 38.76 |
| 88 | 707 | 43.04 |
| 104 | 834 | 36.51 |
| 112 | 900 | 55.26 |
| 128 | 1006 | 39.16 |
| 133 | 1032 | 57.47 |

| | | |
|---|---|---|
| 139 | 1097 | 55.69 |
| 140 | 1100 | 88.39 |
| 142 | 1107 | 38.85 |
| 143 | 1113 | 108.13 |
| 147 | 1133 | 47.17 |
| 148 | 1136 | 110.46 |
| 150 | 1171 | 43.78 |
| 151 | 1181 | 58.70 |
| 160 | 1229 | 120.55 |
| 165 | 1269 | 86.33 |
| 166 | 1272 | 188.41 |
| 167 | 1292 | 135.55 |
| 168 | 1304 | 320.48 |
| 169 | 1315 | 126.77 |
| 174 | 1336 | 58.03 |
| 175 | 1342 | 280.44 |
| 176 | 1345 | 117.37 |
| 177 | 1348 | 65.14 |
| 181 | 1380 | 118.50 |
| 182 | 1384 | 89.23 |
| 191 | 1491 | 35.25 |
| 194 | 1513 | 36.72 |
| 201 | 1546 | 107.57 |
| 202 | 1548 | 175.09 |

| | | |
|---|---|---|
| 203 | 1573 | 146.16 |
| 205 | 1628 | 42.69 |
| 206 | 1630 | 77.38 |
| 207 | 1631 | 33.08 |
| 212 | 1652 | 117.27 |
| 214 | 1659 | 65.74 |
| 215 | 1715 | 639.13 |
| 216 | 3036 | 58.56 |
| 218 | 3068 | 49.04 |
| 231 | 3201 | 43.37 |
| 232 | 3206 | 38.96 |

**10e-*trans*-OPA-Int:**

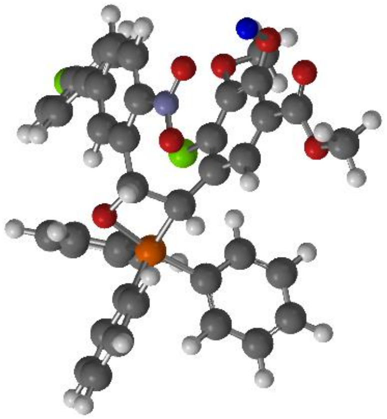

| | Uncorrected $cm^{-1}$ | Intensity |
|---|---|---|
| 72 | 548 | 194.48 |
| 73 | 560 | 45.69 |
| 84 | 672 | 42.27 |
| 88 | 707 | 32.71 |

| | | |
|---|---|---|
| 90 | 713 | 33.01 |
| 93 | 725 | 45.50 |
| 104 | 838 | 40.68 |
| 112 | 907 | 44.00 |
| 133 | 1031 | 42.12 |
| 137 | 1058 | 63.77 |
| 139 | 1087 | 34.55 |
| 141 | 1107 | 46.96 |
| 142 | 1110 | 76.46 |
| 147 | 1125 | 149.77 |
| 149 | 1137 | 339.36 |
| 165 | 1246 | 97.65 |
| 167 | 1300 | 287.74 |
| 169 | 1324 | 46.76 |
| 179 | 1371 | 63.03 |
| 182 | 1387 | 319.48 |
| 183 | 1399 | 53.72 |
| 190 | 1485 | 40.18 |
| 192 | 1502 | 54.47 |
| 194 | 1522 | 50.57 |
| 198 | 1537 | 465.63 |
| 200 | 1548 | 67.91 |
| 203 | 1599 | 93.47 |
| 205 | 1623 | 40.84 |

| | | |
|---|---|---|
| 208 | 1639 | 140.31 |
| 209 | 1640 | 124.26 |
| 210 | 1644 | 143.52 |
| 214 | 1667 | 123.37 |
| 215 | 1790 | 296.22 |
| 216 | 3016 | 54.56 |
| 217 | 3071 | 49.45 |
| 219 | 3113 | 77.48 |
| 232 | 3203 | 38.02 |

**General Methods.**

All reactions were performed in oven dried apparatus. Commercial grade solvents were distilled before use. Melting points were obtained in open capillary tubes and are uncorrected. Infrared spectra were recorded as neat. The samples for NMR were made by dissolving in $CDCl_3$ and TMS is used as an internal standard, the $\delta$ value for the peaks in $^1$H NMR were reported in terms of ppm with reference to TMS (0 ppm) peak and the coupling constants were reported in Hz. The multiplicities are reported as follows br = broad, s = singlet, d = doublet, dd = doublet of doublet, dtd = doublet of triplet of doublet, t = triplet, q = quartet, quin = quintet, sxt = sextet, m = multiplet. The chemical shift in $^{13}$C NMR is assigned by fixing the middle peak of $CDCl_3$ at 77.00 ppm. The NMR data analysis was carried out by using Mnova software. HRMS was recorded using electron spray ionization (ESI) or atmospheric chemical ionization (APCI) mode. The reactions were monitored by thin layer chromatography (tlc) on microscopic slides coated with silica gel and visualization of spots was accomplished by exposure to iodine vapor or spraying with 4% ethanolic $H_2SO_4$ solution or by UV radiation. The silica gel (100 x 200) column chromatography was carried for purification of compounds with various combinations of hexanes and EtOAc solvent system as eluent. Commercial solvents, hexane, ethyl acetate, acetone, acetonitrile, dichloromethane, methanol and toluene were distilled under appropriate drying agents.[1] Tetrahydrofuran was distilled immediately prior to use from sodium/benzophenone ketyl under argon. Distilled water was used for all quenching of the reactions. Commercially available aldehydes were used as

received based on their analytical purities. Sodium methoxide was procured from TCI as granules and used as it is in the reactions. All Wittig olefination reaction were conducted at room temperature in open air conditions, unless otherwise mentioned.

**General procedure for Wittig olefination (GP-2)**

To a stirred solution of Wittig salt **2a** (63 mg, 0.12 mmol) in methanol (1.5 mL) was added NaOMe (38 mg, 0.70 mmol) at room temperature. After 10 minutes, aromatic aldehyde (0.1 mmol) was added, the reaction mixture allowed to stir at room temperature. Based on consumption of starting material (monitored by TLC), the reaction mixture was quenched with distilled water (3 mL) and was diluted with ethyl acetate (10 mL). The organic compound was extracted with ethyl acetate (5 mL x 3) from the aqueous layer. The combined organic layers were washed with brine (5 mL) and dried over $Na_2SO_4$. The solvent was removed under reduced pressure at water bath temperature 45 $^oC$. *E/Z* ratios of all Wittig reactions were calculated based on $^1$H-NMR of crude reaction mixtures, which up on a quick filtration through a short $SiO_2$ bed and washed with 15% ethyl acetate in hexanes. In the case of E or Z isomers separation, we purified the mixtures further with the elution of 10-15% ethyl acetate in hexanes on $SiO_2$ column.

**Methyl 4-chloro-2-hydroxy-3-methoxy-5-(4-nitrostyryl)benzoate (3)**

Compound **3a,b** (32 mg, 89%, *E/Z* = 79:21) has been prepared by following experimental procedure similar to **GP-2**, from Wittig salt **2a** (63 mg, 0.12 mmol) and 4-nitrobenzaldehyde (15.1 mg, 0.1 mmol).

**Data for *Z*-isomer of 3a:**

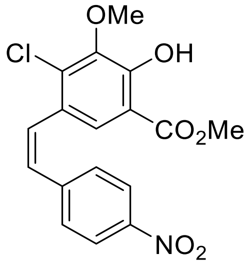


Mp = 117-118 $^oC$ (yellow crystalline solid).

$^1$H NMR (400 MHz, $CDCl_3$) *δ:* 11.02 (s, 1H), 8.04 - 8.10 (m, 2H), 7.35 (s, 1H), 7.27 - 7.33 (m, *J*=8.8 Hz, 2H), 6.69-6.81 (m, 2H), 3.97 (s, 3H), 3.81 (s, 3H).

$^{13}$C NMR (100 MHz, $CDCl_3$) *δ:* 169.8, 155.3, 146.7, 145.4, 143.2, 134.2, 130.0, 129.6, 129.5, 127.1, 126.3, 125.4, 124.2, 123.6, 112.0, 60.7, 52.7.

IR $\nu_{max}$ (Neat) 3144, 2976, 2870, 1664, 1543, 1424, 1169, 1026, 959 $cm^{-1}$.

HRMS-ESI (*m/z*): $[M+H]^+$ calcd for $C_{17}H_{15}ClNO_6$, 364.0588; found, 364.0589.

**Data for *E*-isomer of 3b:**

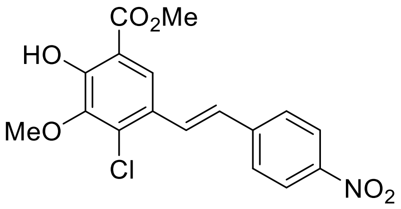


Mp = 105-107 °C (light yellow crystalline solid).

$^{1}$H NMR (400 MHz, $CDCl_3$) *δ:* 11.08 (s, 1H), 8.21-8.26 (m, *J* = 8.8 Hz, 2H), 7.98 (s, 1H), 7.64 - 7.69 (m, *J* = 8.8 Hz, 2H), 7.57 (d, *J* = 16.6 Hz, 1H), 7.05 (d, *J* = 16.1 Hz, 1H), 4.02 (s, 3H), 3.96 (s, 3H).

$^{13}$C NMR (100 MHz, $CDCl_3$) *δ:* 170.0, 155.7, 147.0, 145.2, 143.4, 134.4, 128.4, 127.9, 127.1, 126.4, 124.2, 122.1, 112.2, 60.6, 52.8.

IR $\nu_{max}$ (Neat) 3128, 2986, 2859, 1608, 1594, 1432, 1189, 1065, 980 $cm^{-1}$.

HRMS-ESI (*m/z*): $[M+H]^+$ calcd for $C_{17}H_{15}ClNO_6$, 364.0588; found, 364.0589.

**Methyl 4-chloro-2-hydroxy-3-methoxy-5-(3-nitrostyryl)benzoate (4a,b)**

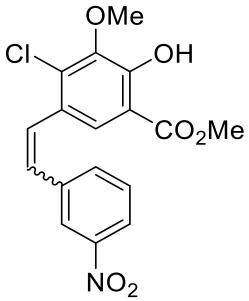


Compound **4a,b** (31.6 mg, 87%, *E/Z* = 24:76) has been prepared by following experimental procedure similar to **GP-2** from Wittig salt **2a** (63 mg, 0.12 mmol) and 3-nitrobenzaldehyde (15.1 mg, 0.1 mmol).

Mp = 114-116 °C (yellow solid)

$^{1}$H NMR (400 MHz, $CDCl_3$) *δ:* 10.99 (s, 1H), 8.00-8.08 (m, 2H), 7.49-7.60 (m, 1H), 7.40-7.47 (m, 1H), 7.31-7.40 (m, 2H), 7.27 (s, 1H), 6.69-6.79 (m, 2H), 4.03 (s, 1H), 3.97 (s, 4H), 3.81 (s, 3H).

$^{13}$C NMR (100 MHz, $CDCl_3$) *δ:* 169.9, 155.2, 148.7, 148.3, 145.4, 138.8, 138.1, 134.6, 134.3, 132.1, 129.7, 129.4, 129.2, 129.0, 127.9, 127.0, 126.3, 125.2, 123.7, 122.4, 122.1, 122.0, 121.4, 111.9, 60.7, 60.6, 52.8, 52.6.

IR $\nu_{max}$ (Neat) 3100, 2980, 2890, 1678, 1543, 1436, 1432, 1389, 1288, 1029, 982 $cm^{-1}$.

HRMS-ESI (*m/z*): $[M+H]^+$ calcd for $C_{17}H_{15}ClNO_6$, 364.0588; found, 364.0596.

**Methyl 4-chloro-2-hydroxy-3-methoxy-5-(2-nitrostyryl)benzoate (5a,b)**

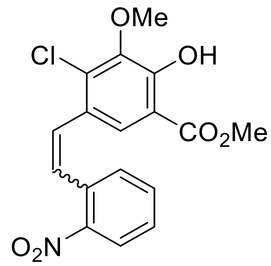


Compound **5a,b** (32 mg, 88%, *E/Z* = 32:68) has been prepared by following experimental procedure similar to **GP-2** from Wittig salt **2a** (63 mg, 0.12 mmol) and 2-nitrobenzaldehyde (15.1 mg, 0.1 mmol).

Mp = 122-125 °C (yellow solid).

$^1$H NMR (400 MHz, $CDCl_3$) $\delta$: 11.11 (s, 1H), 10.91 (s, 1H), 8.08 (dd, *J* = 8.1, 1.9 Hz, 1H), 7.99-7.97 (m, 1H), 7.78 (d, *J* = 7.6 Hz, 1H), 7.64 (t, *J* = 7.6 Hz, 1H), 7.49 (d, *J* = 16.1 Hz, 1H), 7.42 (d, *J* = 16.1 Hz, 1H), 7.41-7.34 (m, 2H), 7.11-7.09 (m, 2H), 7.03 (d, *J* = 11.8 Hz, 1H), 6.81 (d, *J* = 11.8 Hz, 1H), 4.02 (s, 3H), 3.96 (s, 3H). 3.93 (s, 3H), 3.76 (s, 3H).

$^{13}$C NMR (100 MHz, $CDCl_3$) $\delta$: 170.11, 169.85, 155.56, 154.88, 148.20, 147.91, 145.02, 134.38, 134.21, 133.33, 133.23, 132.97, 132.80, 132.00, 128.96, 128.57, 128.53, 128.33, 128.19, 128.02, 126.64, 126.17, 125.95, 125.26, 124.82, 124.60, 122.35, 112.25, 111.62, 60.57, 52.82, 52.50.

IR $\nu_{max}$ (Neat) 3075, 2922, 2853, 1681, 1521, 1455, 1345, 1243, 1148, 960 $cm^{-1}$.

HRMS-ESI (*m/z*): $[M+H]^+$ calcd for $C_{17}H_{15}ClNO_6$, 364.0588; found, 364.0586.

**Methyl (E)-5-(2-bromo-6-nitrostyryl)-4-chloro-2-hydroxy-3-methoxybenzoate (6b)**

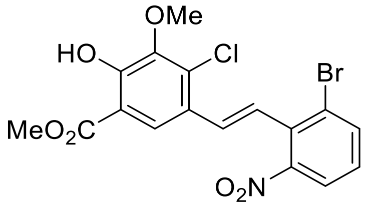


Compound **6b** (42.5 mg, 96%, only *E*-isomer) has been prepared by following experimental procedure similar to **GP-2**, from Wittig salt **2a** (63 mg, 0.12 mmol) and 2-bromo-6-nitrobenzaldehyde (23 mg, 0.1 mmol).

Mp = 160-161 °C (yellow crystalline solid).

$^{1}$H NMR (400 MHz, Chloroform-*d*) δ 11.03 (s, 1H), 7.89 (s, 1H), 7.79 (d, *J* = 8.0 Hz, 1H), 7.69 (d, *J* = 8.1 Hz, 1H), 7.23 (t, *J* = 8.1 Hz, 1H), 6.95 (d, *J* = 16.8 Hz, 1H), 6.95 (d, *J* = 16.8 Hz, 1H), 3.95 (s, 3H), 3.87 (s, 3H).

$^{13}$C NMR (101 MHz, Chloroform-*d*) δ 170.12, 155.78, 150.48, 145.11, 136.68, 134.33, 132.71, 131.83, 128.84, 126.41, 125.45, 124.34, 123.04, 122.42, 112.16, 60.57, 52.83.

IR $\nu_{max}$ (Neat) 3690, 3053, 2959, 1683, 1602, 1536, 1448, 1350, 1059, 967 cm$^{-1}$.

HRMS-EI (*m/z*): [M]$^{+}$ calcd for $C_{17}H_{13}ClBrNO_6$, 440.9615; found, 440.9610.

**Methyl 5-(5-bromo-2-nitrostyryl)-4-chloro-2-hydroxy-3-methoxybenzoate (7a,b)**

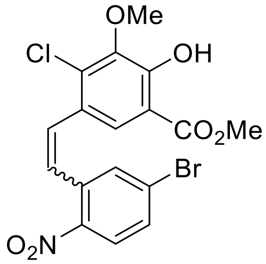


Compound **7a,b** (38.5 mg, 87%, *E/Z* = 29:71) has been prepared by following experimental procedure similar to **GP-2**, from Wittig salt **2a** (63 mg, 0.12 mmol) and 5-bromo-2-nitrobenzaldehyde (23 mg, 0.1 mmol).

Mp = 134-137 °C (light yellow solid).

$^{1}$H NMR (400 MHz, $CDCl_3$) *δ:* 11.13 (s, 1H), 10.93 (s, 1H), 7.96 (s, 1H), 7.95 (d, *J* = 3.5 Hz, 1H), 7.89-7.87 (m, 1H), 7.57-7.56 (dd, *J* = 8.7, 2.0 Hz, 1H), 7.51-7.48 (dd, *J* = 8.8, 2.0 Hz, 1H), 7.45 (d, *J* = 16.1 Hz, 1H), 7.37 (d, *J* = 16.0 Hz, 1H), 7.28 (d, *J* = 2.0 Hz, 1H), 7.12 (s, 1H), 6.96 (d, *J* = 12.2 Hz, 1H), 6.85 (d, *J* = 11.7 Hz, 1H), 4.02 (s, 3H), 3.96 (s, 3H), 3.94 (s, 3H), 3.81 (s, 3H).

$^{13}$C NMR (100 MHz, $CDCl_3$) *δ:* 170.03, 169.74, 155.85, 155.17, 147.96, 146.50, 145.24, 145.16, 134.80, 134.73, 134.59, 134.37, 131.38, 131.32, 131.27, 130.20, 128.97, 128.26, 127.96, 127.16, 126.40, 126.20, 126.09, 125.66, 125.52, 124.09, 122.50, 112.27, 111.67, 60.61, 60.57, 52.86, 52.59.

IR $\nu_{max}$ (Neat) 3100, 2981, 2852, 1742, 1694, 1648, 1517, 1463, 1255, 1009 cm$^{-1}$.

HRMS-ESI (*m/z*): [M+K]$^{+}$ calcd for $C_{17}H_{13}KBrClNO_6$, 479.9252; found, 479.9255.

**Methyl 5-(3-bromo-2-nitrostyryl)-4-chloro-2-hydroxy-3-methoxybenzoate (8a,b)**

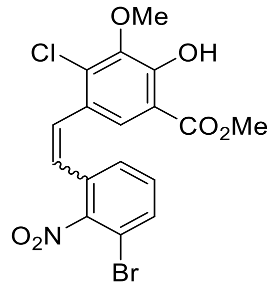


Compound **8a,b** (36 mg, 82%, *E/Z* = 41:59) has been prepared by following experimental procedure similar to **GP-2**, from Wittig salt **2a** (63 mg, 0.12 mmol) and 3-bromo-2-nitrobenzaldehyde (23 mg, 0.1 mmol).

Mp = 136-138 $^oC$ (pale yellow solid).

$^1H$ NMR (400 MHz, $CDCl_3$) *δ:* 11.16 (s, 1H), 7.83 (s, 1H), 7.74 (d, *J* = 7.8 Hz, 1H), 7.59 (d, *J* = 7.8 Hz, 1H), 7.48 (d, *J* = 16.1 Hz, 1H), 7.36-7.41 (m, 1H), 6.72 (d, *J* = 16.1 Hz, 1H), 4.03 (s, 3H), 3.92-3.95 (m, 3H).

$^{13}C$ NMR (100 MHz, $CDCl_3$) *δ:* 170.1, 155.6, 146.3, 134.4, 133.3, 132.5, 132.4, 131.3, 131.1, 130.8, 130.1, 129.4, 127.5, 126.7, 125.9, 125.7, 123.6, 122.5, 121.4, 113.3, 112.9, 112.5, 60.5, 60.4, 52.9, 52.7.

IR $\nu_{max}$ (Neat) 3100, 2925, 2853, 1680, 1537, 1454, 1342, 1243, 1054, 935 $cm^{-1}$.

HRMS-ESI (*m/z*): $[M+H]^+$ calcd for $C_{17}H_{14}BrClNO_6$, 441.9693; found, 441.9705.

**Scheme 1:** Preparation of 5-aryl-2-nitrobenzaldehyde (**9**).

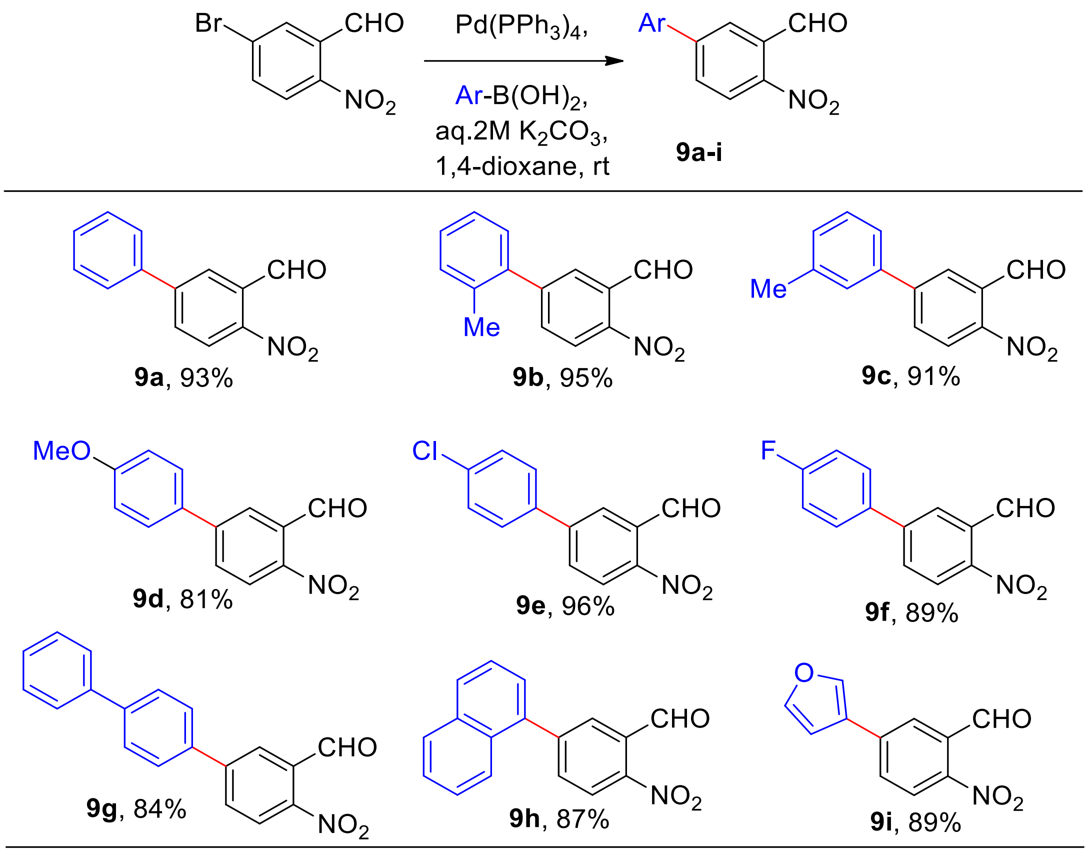


**General procedure for the synthesis of 5-aryl-2-nitrobenzaldehyde 9a-i (GP-3).**[3]

To a mixture of aryl halide (1 mmol) and arylboronic acid (1.5 mmol) in 1,4-dioxane (4 mL) was added aqueous $K_2CO_3$ (2M, 1 mL) solution and purged with argon. To the reaction mixture, 5 mol% of $Pd(PPh_3)_4$ was added and stirred at room temperature for 4 h (monitored by TLC). Based on starting material consumption the reaction mixture was diluted with water and EtOAc. Aqueous layer extracted with EtOAc (3X6 mL), the EtOAc layer was separated, dried and concentrated. The residue was purified by column chromatography (hexane-EtOAc) to afford the desired products **9a-i**.

**4-nitro-[1,1'-biphenyl]-3-carbaldehyde (9a).**

Compound **9a** (211 mg, 93%) has been prepared by following **GP-3** from 5-bromo-2-nitrobenzaldehyde (230 mg, 1.0 mmol) and phenylboronic acid (183 mg, 1.5 mmol). $R_f$ (1% ethyl acetate in hexanes) = 0.5.

Light yellow color solid, Mp = 90-93 $^{o}$C.

$^{1}$H NMR (400 MHz, $CDCl_3$) *δ:* 10.48 (s, 1H), 8.19 (d, *J* = 8.4 Hz, 1H), 8.11 (d, *J* = 2.1 Hz, 1H), 7.92 (dd, *J* = 8.4, 2.0 Hz, 1H), 7.66-7.61 (m, 2H), 7.53-7.44 (m, 3H).

$^{13}$C NMR (100 MHz, $CDCl_3$) *δ:* 188.43, 148.07, 147.29, 137.54, 132.08, 131.54, 129.50, 129.35, 127.87, 127.37, 125.36.

IR $\nu_{max}$ (Neat) 3115, 2876, 1694, 1604, 1545, 1398, 1210, 1019, 970 $cm^{-1}$.

**2'-methyl-4-nitro-[1,1'-biphenyl]-3-carbaldehyde (9b).**

Compound **9b** (230 mg, 95%) has been prepared by following **GP-3** from 5-bromo-2-nitrobenzaldehyde (230 mg, 1.0 mmol) and *o*-tolylboronic acid (204 mg, 1.5 mmol). $R_f$ (1% ethyl acetate in hexanes) = 0.4.

Light yellow color solid, Mp = 107-110 $^{o}$C.

$^{1}$H NMR (400 MHz, $CDCl_3$) δ 10.43 (s, 1H), 8.13 (d, *J* = 8.3 Hz, 1H), 7.83 (d, *J* = 2.1 Hz, 1H), 7.63 (dd, *J* = 8.4, 2.0 Hz, 1H), 7.26 (ttd, *J* = 13.8, 7.4, 1.8 Hz, 3H), 7.15 (dd, *J* = 7.5, 1.5 Hz, 1H), 2.21 (s, 3H).

$^{13}$C NMR (101 MHz, $CDCl_3$) δ 188.34, 148.60, 148.00, 138.45, 135.06, 134.14, 131.44, 130.92, 130.27, 129.45, 128.98, 126.36, 124.64, 20.33.

IR $\nu_{max}$ (Neat) 3110, 2856, 1698, 1610, 1547, 1389, 1215, 1014, 973 $cm^{-1}$.

HRMS-ESI (*m/z*): $[M]^+$ calcd for $C_{14}H_{11}NO_3$, 341.0739; found, 341.0743.

**3'-methyl-4-nitro-[1,1'-biphenyl]-3-carbaldehyde (9c).**

Compound **9c** (220 mg, 91%) has been prepared by following **GP-3** from 5-bromo-2-nitrobenzaldehyde (230 mg, 1.0 mmol) and *m*-tolylboronic acid (204 mg, 1.5 mmol). $R_f$ (2% ethyl acetate in hexanes) = 0.6.

Light yellow color solid, Mp = 116-118 $^{o}$C.

$^{1}$H NMR (400 MHz, $CDCl_3$) *δ:* 10.49 (s, 1H), 8.19 (d, *J* = 8.5 Hz, 1H), 8.11 (d, *J* = 2.0 Hz, 1H), 7.92 (dd, *J* = 8.5, 2.1 Hz, 1H), 7.45-7.37 (m, 3H), 7.31-7.26 (m, 1H), 2.44 (s, 3H).

$^{13}$C NMR (100 MHz, $CDCl_3$) *δ:* 188.49, 147.99, 147.48, 139.16, 137.52, 132.05, 131.51, 130.25, 129.24, 128.08, 127.85, 125.30, 124.49, 21.48.

IR $\nu_{max}$ (Neat) 3100, 2858, 1689, 1614, 1550, 1391, 1213, 1006, 975 $cm^{-1}$.

**4'-methoxy-4-nitro-[1,1'-biphenyl]-3-carbaldehyde (9d).**

Compound **9d** (210 mg, 81%) has been prepared by following **GP-3** from 5-bromo-2-nitrobenzaldehyde (230 mg, 1.0 mmol) and (4-methoxyphenyl)boronic acid (228 mg, 1.5 mmol). $R_f$ (3% ethyl acetate in hexanes) = 0.4.

Light yellow color solid, Mp = 110-112 $^oC$.

$^1H$ NMR (400 MHz, $CDCl_3$) *δ:* 10.47 (s, 1H), 8.16 (d, *J* = 8.5 Hz, 1H), 8.04 (d, *J* = 2.2 Hz, 1H), 7.86 (dd, *J* = 8.5, 2.2 Hz, 1H), 7.63-7.55 (m, 2H), 7.05-6.97 (m, 2H), 3.87 (s, 3H).

$^{13}C$ NMR (100 MHz, $CDCl_3$) *δ:* 188.65, 160.89, 147.36, 146.85, 132.21, 130.62, 129.74, 128.61, 127.00, 125.40, 114.78, 55.45.

IR $\nu_{max}$ (Neat) 3106, 2865, 1686, 1618, 1553, 1384, 1201, 1010, 970 $cm^{-1}$.

**4'-chloro-4-nitro-[1,1'-biphenyl]-3-carbaldehyde (9e).**

Compound **9e** (250 mg, 96%) has been prepared by following **GP-3** from 5-bromo-2-nitrobenzaldehyde (230 mg, 1.0 mmol) and (4-chlorophenyl)boronic acid (235 mg, 1.5 mmol). $R_f$ (3% ethyl acetate in hexanes) = 0.6. Light yellow color solid, Mp = 121-122 $^oC$.

$^1H$ NMR (400 MHz, $CDCl_3$) *δ:* 10.47 (s, 1H), 8.20 (d, *J* = 8.4 Hz, 1H), 8.07 (d, *J* = 2.2 Hz, 1H), 7.89 (dd, *J* = 8.5, 2.1 Hz, 1H), 7.60-7.55 (m, 2H), 7.50-7.44 (m, 2H).

$^{13}C$ NMR (100 MHz, $CDCl_3$) *δ:* 188.21, 148.24, 145.98, 135.98, 135.89, 132.12, 131.39, 129.58, 128.63, 127.73, 125.48.

IR $\nu_{max}$ (Neat) 3097, 2860, 1696, 1611, 1555, 1390, 1208, 1012, 963 $cm^{-1}$.

**4'-fluoro-4-nitro-[1,1'-biphenyl]-3-carbaldehyde (9f).**

Compound **9f** (219 mg, 89%) has been prepared by following **GP-3** from 5-bromo-2-nitrobenzaldehyde (230 mg, 1.0 mmol) and (4-fluorophenyl)boronic acid (210 mg, 1.5 mmol). $R_f$ (2% ethyl acetate in hexanes) = 0.4. Light yellow color solid, Mp = 105-107 $^oC$.

$^1H$ NMR (400 MHz, $CDCl_3$) *δ:* $^1H$ NMR (300 MHz, $CDCl_3$) δ 10.52 (s, 1H), 8.22 (d, *J* = 8.4 Hz, 1H), 8.10 (d, *J* = 2.0 Hz, 1H), 7.90 (dd, *J* = 8.4, 2.1 Hz, 1H), 7.64 (dd, *J* = 8.7, 5.2 Hz, 2H), 7.21 (t, *J* = 8.5 Hz, 2H).

$^{13}C$ NMR (100 MHz, $CDCl_3$) *δ:* 188.32, 146.85, 146.26, 132.17, 131.37, 129.31. 129.19, 127.75, 125.46, 116.61, 116.33.

IR $\nu_{max}$ (Neat) 3094, 2869, 1691, 1616, 1552, 1387, 1200, 1009, 960 $cm^{-1}$.

**4-nitro-[1,1':4',1''-terphenyl]-3-carbaldehyde (9g).**

Compound **9g** (254 mg, 84%) has been prepared by following **GP-3** from 5-bromo-2-nitrobenzaldehyde (230 mg, 1.0 mmol) and [1,1'-biphenyl]-4-ylboronic acid (297 mg, 1.5 mmol). $R_f$ (4% ethyl acetate in hexanes) = 0.6. Light yellow color solid, Mp = 126-128 $^oC$.

$^1H$ NMR (400 MHz, $CDCl_3$) *δ:* 10.50 (s, 1H), 8.18 (d, *J* = 18.4 Hz, 2H), 7.95 (s, 1H), 7.55 (m, 9H).

$^{13}C$ NMR (100 MHz, $CDCl_3$) *δ:* 188.44, 148.05, 146.82, 142.40, 139.94, 136.31, 132.22, 131.31, 129.02, 128.01, 127.80, 127.12, 125.46.

IR $\nu_{max}$ (Neat) 3107, 2850, 1684, 1620, 1557, 1380, 1213, 1015, 969 $cm^{-1}$.

**5-(naphthalen-1-yl)-2-nitrobenzaldehyde (9h).**

Compound **9h** (240 mg, 87%) has been prepared by following **GP-3** from 5-bromo-2-nitrobenzaldehyde (230 mg, 1.0 mmol) and naphthalen-1-ylboronic acid (258 mg, 1.5 mmol). $R_f$ (3% ethyl acetate in hexanes) = 0.4.

Light yellow color solid, Mp = 129-130 $^oC$.

$^1H$ NMR (400 MHz, $CDCl_3$) δ 10.45 (s, 1H), 8.18 (d, *J* = 8.4 Hz, 1H), 7.98 (d, *J* = 2.0 Hz, 1H), 7.91-7.83 (m, 2H), 7.79 (dd, *J* = 8.3, 2.0 Hz, 1H), 7.65 (dd, *J* = 8.5, 1.3 Hz, 1H), 7.52-7.38 (m, 3H), 7.35 (dd, *J* = 7.1, 1.2 Hz, 1H).

$^{13}C$ NMR (101 MHz, $CDCl_3$) δ 188.29, 148.29, 147.50, 136.59, 134.93, 133.81, 131.67, 131.02, 130.69, 129.52, 128.74, 127.41, 127.12, 126.44, 125.37, 124.79, 124.72.

IR $\nu_{max}$ (Neat) 3117, 2859, 1686, 1625, 1566, 1389, 1217, 1014, 974 $cm^{-1}$.

HRMS-ESI (*m/z*): $[M]^+$ calcd for $C_{17}H_{11}NO_3$, 377.0739; found, 377.0747.

**5-(naphthalen-1-yl)-2-nitrobenzaldehyde (9i).**

Compound **9i** (193 mg, 89%) has been prepared by following **GP-3** from 5-bromo-2-nitrobenzaldehyde (230 mg, 1.0 mmol) and furan-3-ylboronic acid (168 mg, 1.5 mmol). $R_f$ (2% ethyl acetate in hexanes) = 0.6.

Light yellow color solid, Mp = 100-102 $^oC$.

$^1H$ NMR (400 MHz, $CDCl_3$) *δ*: 10.49 (s, 1H), 8.16 (d, *J* = 8.5 Hz, 1H), 8.02–7.89 (m, 2H), 7.80 (dd, *J* = 8.5, 2.0 Hz, 1H), 7.56 (t, J = 1.4 Hz, 1H), 6.79 (d, *J* = 0.7 Hz, 1H).

$^{13}C$ NMR (100 MHz, $CDCl_3$) *δ:* 188.45, 147.40, 144.87, 140.84, 139.09, 132.40, 129.79, 126.19, 125.55, 124.09, 108.37.

IR $\nu_{max}$ (Neat) 3112, 2869, 1691, 1615, 1562, 1394, 1211, 1004, 977 $cm^{-1}$.

**Scheme 2: Synthesis of methyl salicylate derived *Z*-selective 5'aryl-2'-nitro-stilbenoids (10a-i).**

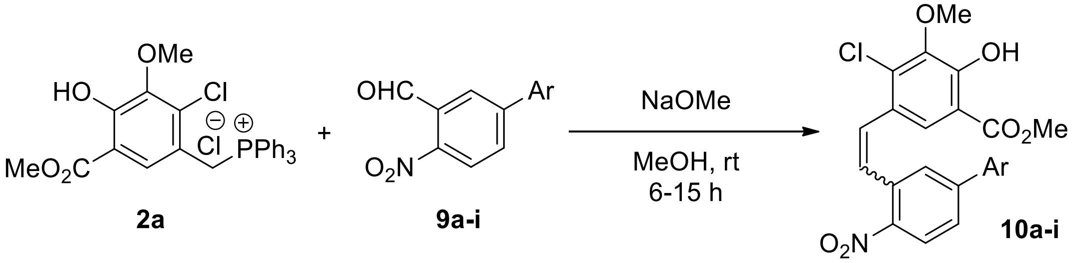


**Methyl 4-chloro-2-hydroxy-3-methoxy-5-(2-(4-nitro-[1,1'-biphenyl]-3-yl)vinyl)benzoate (10a)**

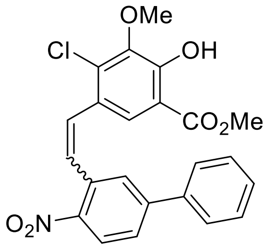


Compound **10a** (40 mg, 90%, *E/Z* = 24:76) has been prepared by following **GP-2** from Wittig salt **2a** (63 mg, 0.119 mmol) and 4-nitro-[1,1'-biphenyl]-3-carbaldehyde **9a** (22.7 mg, 0.100 mmol). $R_f$ (8% ethyl acetate in hexanes) = 0.5.

Yellow color solid, Mp = 108-110 °C.

$^1$H NMR (400 MHz, $CDCl_3$) $\delta$: 11.07 (s, 1H), 10.86 (s, 1H), 8.15 (d, *J* = 8.3 Hz, 1H), 8.09 (d, *J* = 8.5 Hz, 1H), 7.99 (s, 1H), 7.91 (d, *J* = 1.8 Hz, 1H), 7.66-7.63 (m, 1H), 7.58-7.52 (m, 3H),7.51 (d, *J* = 16.1 Hz, 1H), 7.41 (d, *J* = 16.1 Hz, 1H) 7.39-7.36 (m, 3H), 7.33-7.31 (m, 2H), 7.28-7.23 (m, 2H), 7.21 (s, 1H), 7.12 (d, *J* = 11.7 Hz, 1H), 6.86 (d, *J* = 11.7 Hz, 1H), 4.01 (s, 3H), 3.96 (s, 3H), 3.92 (s, 3H), 3.74 (s, 3H).

$^{13}$C NMR (100 MHz, $CDCl_3$) $\delta$: 170.09, 169.76, 155.61, 154.93, 146.95, 146.69, 146.51, 146.03, 145.24, 138.79, 138.36, 134.44, 134.20, 133.59, 133.48, 130.52, 129.16, 129.06, 128.94, 128.89, 128.82, 128.27, 127.36, 127.20, 127.01, 126.85, 126.74, 126.52, 126.45, 125.82, 125.56, 125.29, 122.43, 112.32, 111.71, 60.51 (2C), 52.73, 52.41.

IR $\nu_{max}$ (Neat) 3071, 2926, 2854, 1679, 1515, 1448, 1341, 1243, 1021, 944 cm$^{-1}$.

HRMS-ESI (*m/z*): $[M+H]^+$ calcd for $C_{23}H_{18}ClNO_6$, 440.0895; found, 440.0894.

**Methyl 4-chloro-2-hydroxy-3-methoxy-5-(2-(2'-methyl-4-nitro-[1,1'-biphenyl]-3-yl)vinyl)benzoate (10b)**

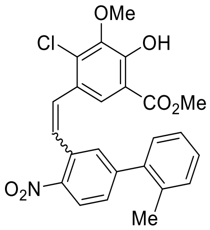


Compound **10b** (35 mg, 76%, *E/Z* = 38:62) has been prepared by following **GP-2** from Wittig salt **2a** (63 mg, 0.119 mmol) and 2'-methyl-4-nitro-[1,1'-biphenyl]-3-carbaldehyde **9b** (24.1 mg, 0.100 mmol). $R_f$ (10% ethyl acetate in hexanes) = 0.5.

Yellow color solid, Mp = 135-138 $^oC$.

$^1H$ NMR (400 MHz, $CDCl_3$) *δ:* 11.10 (s, 1H), 10.91 (s, 1H), 8.17 (dd, *J* = 8.3, 1.3 Hz, 1H), 8.07 (dd, *J* = 8.3, 1.5 Hz, 1H), 7.99 (d, *J* = 1.32 Hz, 1H), 7.71 (s, 1H), 7.57 (d, *J* = 16.0 Hz, 1H), 7.42-7.39 (m, 6H), 7.37 (d, *J* = 16.0 Hz, 1H), 7.18-7.15 (m, 3H), 7.10 (d, *J* = 11.7 1H), 7.07 (s, 1H), 6.92 (d, *J* = 7.8 Hz, 1H), 6.84 (d, *J* = 11.7 Hz, 1H), 4.02 (s, 3H), 3.94 (s, 3H), 3.91 (s, 3H), 3.78 (s, 3H), 2.32 (s, 3H), 1.85 (s, 3H).

$^{13}C$ NMR (100 MHz, $CDCl_3$) *δ:* 170.12, 169.82, 155.60, 154.91, 147.56, 147.44, 146.73, 146.47, 145.16, 145.10, 139.52, 139.16, 135.16, 134.76, 134.56, 134.24, 133.00, 132.89, 132.83, 130.78, 130.59, 129.41, 129.28, 129.16, 129.10, 129.04, 128.93, 128.54, 128.45, 128.08, 126.66, 126.30, 126.20, 126.08, 125.51, 124.90, 124.74, 122.42, 112.27, 111.60, 60.53, 52.82, 52.55, 20.41, 19.60.

IR $\nu_{max}$ (Neat) 3068, 2926, 2854, 1679, 1515, 1449, 1341, 1243, 1089, 944 $cm^{-1}$.

HRMS-ESI (*m/z*): $[M+H]^+$ calcd for $C_{24}H_{20}ClNO_6$, 454.1052; found, 454.1058.

**Methyl 4-chloro-2-hydroxy-3-methoxy-5-(2-(3'-methyl-4-nitro-[1,1'-biphenyl]-3-yl)vinyl)benzoate (10c)**

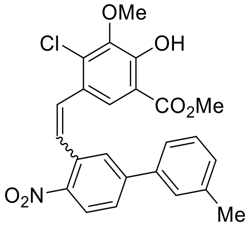

Compound **10c** (36 mg, 79%, *E/Z* = 23:77) has been prepared by following **GP-2** from Wittig salt **2a** (63 mg, 0.119 mmol) and 3'-methyl-4-nitro-[1,1'-biphenyl]-3-carbaldehyde **9c** (24.1 mg, 0.100 mmol). $R_f$ (10% ethyl acetate in hexanes) = 0.5.

Yellow color solid, Mp = 136-137 °C.

$^1$H NMR (400 MHz, $CDCl_3$) $\delta$: 11.11, (s, 1H), 10.92 (s, 1H), 8.15 (d, *J* = 8.3 Hz, 1H), 8.09 (d, *J* = 8.5 Hz, 1H), 8.00 (s, 1H), 7.89 (d, *J* = 2.0 Hz, 1H), 7.62-7.57 (m, 1H), 7.59 (d, *J* = 16.1 Hz, 1H), 7.53 (dd, *J* = 8.8, 2.0 Hz, 1H), 7.46-7.42 (m, 3H), 7.41 (d, *J* = 16.1 Hz, 1H), 7.31 (d, *J* = 2.0 Hz, 1H), 7.25-7.23 (m, 2H), 7.18-7.16 (m, 2H), 7.12 (d, *J* = 11.7 Hz, 1H), 7.07 (d, *J* = 7.3 Hz, 1H), 6.98 (s, 1H), 6.86 (d, *J* = 11.2 Hz, 1H), 4.01 (s, 3H), 3.95 (s, 3H), 3.92 (s, 3H), 3.74 (s, 3H), 2.45 (s, 3H), 2.34 (s, 3H).

$^{13}$C NMR (100 MHz, $CDCl_3$) $\delta$: 170.13, 169.83, 155.58, 154.92, 146.73, 146.53, 146.19, 145.22, 145.10, 138.94, 138.84, 138.76, 138.33, 134.48, 134.25, 133.59, 133.36, 130.57, 129.69, 129.60, 129.10, 129.03, 128.97, 128.20, 128.09, 127.90, 127.25, 126.93, 126.74, 126.59, 126.51, 125.94, 125.88, 125.58, 125.33, 124.53, 124.10, 122.45, 112.28, 111.72, 60.58, 52.82, 52.52, 21.54, 21.42.

IR $\nu_{max}$ (Neat) 3200, 2925, 2854, 1679, 1515, 1449, 1341, 1240, 1029, 910 cm$^{-1}$.

HRMS-ESI (*m/z*): $[M+H]^+$ calcd for $C_{24}H_{20}ClNO_6$, 454.1052; found, 454.1047.

**Methyl 4-chloro-2-hydroxy-3-methoxy-5-(2-(4'-methoxy-4-nitro-[1,1'-biphenyl]-3-yl)vinyl)benzoate (10d)**

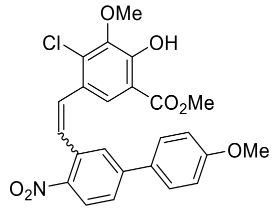


Compound **10d** (38 mg, 80%, *E/Z* = 20:80) has been prepared by following **GP-2** from Wittig salt **2a** (63 mg, 0.119 mmol) and 4'-methoxy-4-nitro-[1,1'-biphenyl]-3-carbaldehyde **9d** (25.7 mg, 0.100 mmol). $R_f$ (12% ethyl acetate in hexanes) = 0.5.

Yellow color solid, Mp = 165-167 °C.

$^1$H NMR (400 MHz, $CDCl_3$) $\delta$: 11.11 (s, 1H), 10.90 (s, 1H), 8.15 (d, *J* = 8.3 Hz, 1H), 8.09 (d, *J* = 8.6 Hz, 1H), 8.00 (s, 1H), 7.86 (d, *J* = 2.0 Hz, 1H), 7.62-7.57 (m, 2H), 7.51 (dd, *J* = 8.3, 2.0 Hz, 1H), 7.40 (d, *J* = 16.0 Hz, 1H), 7.29-7.26 (m, 3H), 7.23-7.19 (m, 3H), 7.12 (d, *J* =

11.7 Hz, 1H), 7.05-7.02 (m, 1H), 6.91-6.87 (m, 3H), 6.85 (d, $J$ = 11.7 Hz, 1H), 4.01 (s, 3H), 3.95 (s, 3H), 3.93 (s, 3H), 3.88 (s, 3H), 3.82 (s, 3H), 3.74 (s, 3H).

$^{13}$C NMR (100 MHz, $CDCl_3$) $\delta$: 170.14, 169.82, 160.48, 160.40, 155.56, 154.86, 146.21, 146.15, 145.71, 145.11, 134.46, 133.74, 133.61, 131.01, 130.58, 129.83, 129.30, 128.88, 128.57, 128.23, 127.94, 126.75, 126.59, 126.49, 126.27, 126.13, 125.91, 125.72, 125.45, 122.44, 114.66, 114.57, 111.68, 60.59, 55.45, 55.41, 52.82, 52.51.

IR $\nu_{max}$ (Neat) 3213, 2932, 2849, 1676, 1598, 1512, 1441, 1336, 1244, 1185, 991 cm$^{-1}$.

HRMS-ESI (*m/z*): $[M+H]^+$ calcd for $C_{24}H_{20}ClNO_7$, 470.1001; found, 470.1000.

**Methyl 4-chloro-5-(2-(4'-chloro-4-nitro-[1,1'-biphenyl]-3-yl)vinyl)-2-hydroxy-3-methoxybenzoate (10e)**

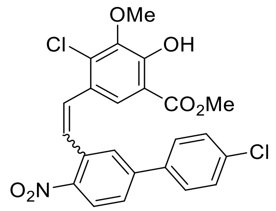


Compound **10e** (43 mg, 91%, *E/Z* = 13:87) has been prepared by following **GP-2** from Wittig salt **2a** (63 mg, 0.119 mmol) and 4'-chloro-4-nitro-[1,1'-biphenyl]-3-carbaldehyde **9e** (26.1 mg, 0.100 mmol). $R_f$ (10% ethyl acetate in hexanes) = 0.5.

Light yellow color solid, Mp = 147-149 $^{o}$C.

$^{1}$H NMR (400 MHz, $CDCl_3$) $\delta$: 11.12, (s, 1H), 10.90 (s, 1H), 8.17 (d, $J$ = 8.3 Hz, 1H), 8.11 (d, $J$ = 8.6 Hz, 1H), 8.00 (s, 1H), 7.87 (d, $J$ = 1.9 Hz, 1H), 7.60-7.59 (m, 1H), 7.57 (d, $J$ = 16.1 Hz, 1H), 7.51 (dd, $J$ = 8.8, 1.9 3H), 7.41 (d, $J$ = 16.1 Hz, 1H), 7.37-7.33 (m, 3H), 7.28 (m, 2H), 7.21-7.16 (m, 3H), 7.12 (d, $J$ = 11.7 Hz, 1H), 6.87 (d, $J$ = 11.7 Hz, 1H), 4.02 (s, 1H), 3.96 (s, 3H), 3.92 (s, 3H), 3.75 (s, 3H).

$^{13}$C NMR (100 MHz, $CDCl_3$) $\delta$: 170.10, 169.75, 154.95, 147.00, 145.18, 144.77, 136.71, 135.23, 134.45, 134.20, 133.75, 133.68, 130.40, 129.42, 129.33, 129.10, 128.89, 128.65, 128.41, 128.26, 127.08, 126.70, 126.57, 126.39, 125.81, 125.74, 125.60, 125.49, 122.45, 111.69, 60.61 (2C), 52.84, 52.55.

IR $\nu_{max}$ (Neat) 3128, 3004, 2943, 2845, 1678, 1601, 1513, 1445, 1338, 1247, 1187, 986 cm$^{-1}$.

HRMS-ESI (*m/z*): $[M+Na]^+$ calcd for $C_{23}H_{17}Cl_2NO_6$, 496.0325; found, 496.0329.

**Methyl 4-chloro-5-(2-(4'-fluoro-4-nitro-[1,1'-biphenyl]-3-yl)vinyl)-2-hydroxy-3-methoxybenzoate (10f)**

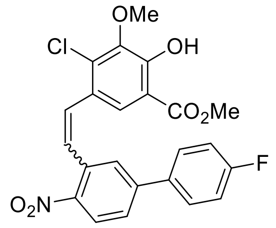


Compound **10f** (40 mg, 87%, *E/Z* = 27:73) has been prepared by following **GP-2** from Wittig salt **2a** (63 mg, 0.119 mmol) and 4'-fluoro-4-nitro-[1,1'-biphenyl]-3-carbaldehyde **9f** (24.5 mg, 0.100 mmol). $R_f$ (10% ethyl acetate in hexanes) = 0.5.

Yellow color solid, Mp = 90-93 $^oC$.

$^1H$ NMR (400 MHz, $CDCl_3$) *δ:* 11.11 (s, 1H), 10.90 (s, 1H), 8.16 (d, *J* = 8.8 Hz, 1H), 8.10 (d, *J* = 8.5 Hz, 1H), 7.99 (s, 1H), 7.86 (d, *J* = 2.0 Hz, 1H), 7.65-7.58 (m, 2H), 7.57 (d, *J* = 16.0 Hz, 1H), 7.51 (dd, *J* = 8.8, 2.0 Hz, 1H), 7.41 (d, *J* = 16.0 Hz, 1H), 7.27-7.19 (m, 5H), 7.12 (d, *J* = 11.8 Hz, 1H), 7.10-7.04 (m, 4H), 6.86 (d, *J* = 11.7 Hz, 1H), 4.01 (s, 3H), 3.96 (s, 3H), 3.93 (s, 3H), 3.75 (s, 3H).

$^{13}C$ NMR (100 MHz, $CDCl_3$) *δ:* 170.09, 169.76, 164.50, 162.02, 155.63, 154.92, 146.80, 146.57, 145.46, 145.16, 145.11, 145.00, 134.88, 134.45, 134.41, 134.22, 133.71, 133.64, 130.36, 129.19, 129.11, 128.98, 128.82, 128.74, 128.30, 127.05, 126.70, 126.60, 126.41, 126.39, 125.83, 125.70, 125.44, 122.44, 116.33, 116.25, 116.11, 116.04, 112.29, 111.68, 60.58, 52.82, 52.54.

IR $\nu_{max}$ (Neat) 3181, 2922, 2853, 1679, 1453, 1341, 1238, 1151, 1054, 960 $cm^{-1}$.

HRMS-ESI (*m/z*): $[M+H]^+$ calcd for $C_{23}H_{17}ClFNO_6$, 458.0801; found, 458.0799.

**Methyl 4-chloro-2-hydroxy-3-methoxy-5-(2-(4-nitro-[1,1':4',1''-terphenyl]-3-yl)vinyl)benzoate (10g)**

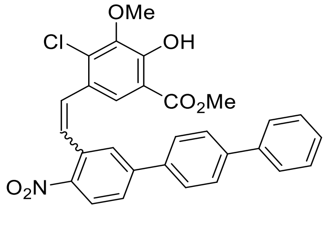


Compound **10g** (43 mg, 82%, *E/Z* = 26:74) has been prepared by following **GP-2** from Wittig salt **2a** (63 mg, 0.119 mmol) and 4-nitro-[1,1':4',1"-terphenyl]-3-carbaldehyde **9g** (30.3 mg, 0.100 mmol). $R_f$ (8% ethyl acetate in hexanes) = 0.5.

Light yellow color solid, Mp = 131-134 °C.

$^1$H NMR (400 MHz, $CDCl_3$) *δ:* 11.11 (s, 1H), 10.92 (s, 1H), 8.19 (d, *J* = 8.8 Hz, 1H), 8.12 (d, *J* = 8.5 Hz, 1H), 8.01 (s, 1H), 7.96 (d, *J* = 2.0 Hz, 1H), 7.74 (s, 1H), 7.69-7.56 (m, 8H), 7.50-7.47 (m, 4H), 7.46 (d, *J* = 16.0 Hz, 1H), 7.44-7.32 (m, 5H), 7.23 (s, 1H), 7.14 (d, *J* = 11.7 Hz, 1H), 6.88 (d, *J* = 11.7 Hz, 1H), 4.02 (s, 3H), 3.96 (s, 3H), 3.94 (s, 3H), 3.75 (s, 3H).

$^{13}$C NMR (100 MHz, $CDCl_3$) *δ:* 170.14, 169.81, 155.63, 154.95, 146.82, 146.04, 146.6, 145.58, 145.19, 141.85, 140.14, 140.01, 137.05, 137.1, 134.50, 134.3, 133.74, 133.64, 130.38, 129.14, 129.08, 129.0, 128.93, 128.24, 127.89, 127.80, 127.44, 127.11, 127.06, 126.71, 126.46, 126.40, 125.91, 125.73, 125.46, 122.47, 112.30, 111.73, 60.62, 52.83, 52.54.

IR $\nu_{max}$ (Neat) 3112, 2923, 2853, 1682, 1515, 1459, 1340, 1244, 1054 cm$^{-1}$.

HRMS-ESI (*m/z*): $[M+H]^+$ calcd for $C_{29}H_{22}ClNO_6$, 516.1208; found, 516.1209.

**Methyl 4-chloro-2-hydroxy-3-methoxy-5-(5-(naphthalen-1-yl)-2-nitrostyryl)benzoate (10h)**

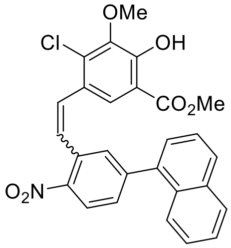


Compound **10h** (41 mg, 83%, *E/Z* = 40:60) has been prepared by following **GP-2** from Wittig salt **2a** (63 mg, 0.119 mmol) and 5-(naphthalen-1-yl)-2-nitrobenzaldehyde **9h** (27.7 mg, 0.100 mmol). $R_f$ (8% ethyl acetate in hexanes) = 0.5.

Yellow color solid, Mp = 160-163 °C.

$^1$H NMR (400 MHz, $CDCl_3$) *δ:* 11.10 (s, 1H), 11.00 (s, 1H), 8.25 (d, *J* = 8.3 Hz, 1H), 8.14 (d, *J* = 8.3 Hz, 1H), 8.00 (s, 1H), 7.95 (d, *J* = 7.8 Hz, 2H), 7.88 (d, *J* = 1.9 Hz, 1H), 7.86-7.82 (m, 3H), 7.61 (d, *J* = 16.0 Hz, 1H), 7.59-7.41 (m, 7H), 7.39 (d, *J* = 16.0 Hz, 1H), 7.29 (s, 1H), 7.25-7.24 (m, 1H), 7.23-7.18 (m, 1H), 7.17-7.11 (m, 2H), 6.85 (d, *J*=11.7 Hz, 1H), 4.01 (s, 3H), 3.92 (s, 3H), 3.83 (s, 3H), 3.79 (s, 3H).

$^{13}$C NMR (100 MHz, $CDCl_3$) *δ:* 170.12, 169.93, 155.62, 155.01, 147.96, 147.03, 146.80, 146.44, 145.24, 145.09, 137.68, 137.33, 134.70, 134.29, 133.83, 133.66, 133.57, 133.11, 133.08, 131.02, 130.70, 130.03, 129.95, 129.79, 129.31, 129.01, 128.95, 128.87, 128.63,

128.54, 128.16, 127.08, 126.94, 126.82, 126.63, 126.48, 126.34, 126.30, 126.23, 126.10, 125.38, 125.22, 125.18, 125.05 124.47, 122.44, 112.26, 111.63, 60.55, 60.50, 52.84, 52.66.

IR $\nu_{max}$ (Neat) 3742, 3061, 2937, 2854, 1678, 1515, 1445, 1341, 1243, 1009 $cm^{-1}$.

HRMS-ESI (*m/z*): $[M+H]^+$ calcd for $C_{27}H_{20}ClNO_6$, 490.1052; found, 490.1042.

**Methyl 4-chloro-5-(5-(furan-3-yl)-2-nitrostyryl)-2-hydroxy-3-methoxybenzoate (10i)**

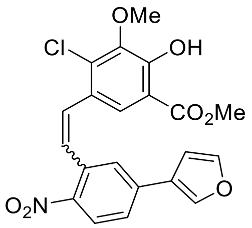


Compound **10i** (34 mg, 78%, *E/Z* = 21:79) has been prepared by following **GP-2** from Wittig salt **2a** (63 mg, 0.119 mmol) and 5-(furan-3-yl)-2-nitrobenzaldehyde **9i** (21.7 mg, 0.100 mmol). $R_f$ (12% ethyl acetate in hexanes) = 0.5.

Yellow color solid, Mp = 122-124 °C.

$^1$H NMR (400 MHz, $CDCl_3$) $\delta$: 11.11 (s, 1H), 10.89 (s, 1H), 8.12 (d, *J* = 8.8 Hz, 1H), 8.06 (d, *J* = 8.5 Hz, 1H), 7.99 (s, 1H), 7.90 (t, *J* = 1.3 1H), 7.78 (d, *J* = 1.9 Hz, 1H), 7.60-7.55 (m, 2H), 7.53-7.50 (m, 2H), 7.44-7.43 (m, 1H), 7.42 (d, *J* = 1.9 Hz, 1H), 7.37 (d, *J* = 16.0 Hz, 1H), 7.20 (d, *J* = 1.8 Hz, 1H), 7.18 (d, *J* = 1.0 Hz, 1H), 7.10 (d, *J* = 11.7 Hz, 1H), 6.84 (d, *J* = 11.7 Hz, 1H), 6,80-6.79 (m, 1H), 6.45- 6.44 (m, 1H), 4.01 (s, 3H), 3.96 (s, 3H), 3.92 (s, 3H), 3.75 (s, 3H).

$^{13}$C NMR (100 MHz, $CDCl_3$) $\delta$: 170.11, 169.80, 155.60, 154.87, 146.22, 145.99, 145.05, 144.55, 144.49, 140.28, 140.01, 138.08, 137.75, 134.30, 134.21, 134.02, 133.90, 129.14, 129.01, 128.80, 128.08, 126.65, 126.40, 125.95, 125.86, 125.81, 125.64,125.57, 125.34, 125.01, 124.72, 124.40, 122.45, 112.28, 111.70, 108.61, 108.22, 60.58, 52.82, 52.51.

IR $\nu_{max}$ (Neat) 3742, 3131, 2946, 2854, 1678, 1601, 1514, 1446, 1339, 1244, 967 $cm^{-1}$.

HRMS-ESI (*m/z*): $[M+H]^+$ calcd for $C_{21}H_{16}ClNO_7$, 430.0688; found, 430.0696.

## Scheme 3:

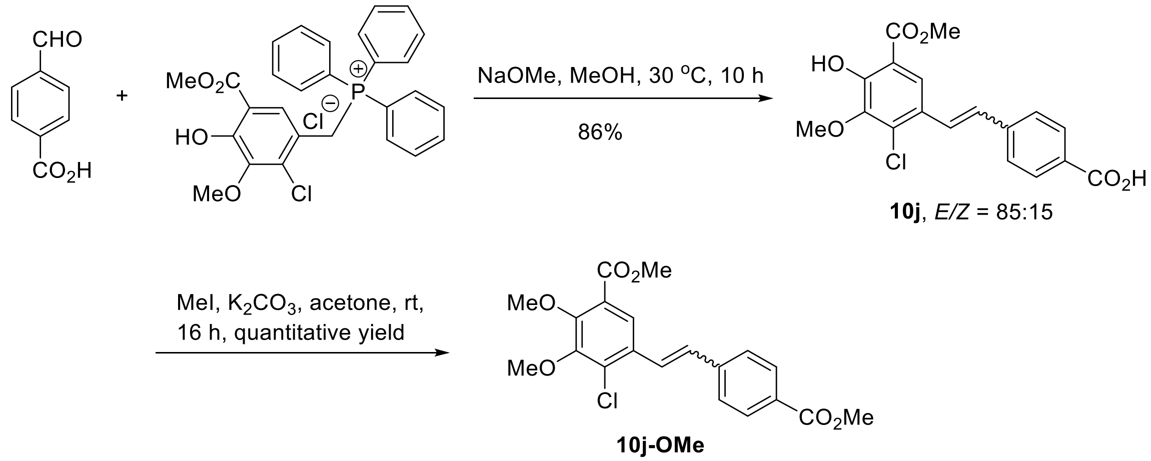


**4-(2-chloro-4-hydroxy-3-methoxy-5-(methoxycarbonyl)styryl)benzoic acid (10j)**

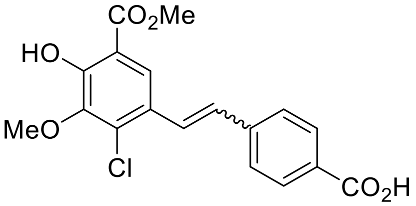


Compound **10j** (31 mg, 86%, *E/Z* = 85:15) has been prepared by following experimental procedure similar to **GP-2** from Wittig salt **2a** (63 mg, 0.12 mmol) and 4-carboxybenzaldehyde (15 mg, 0.1 mmol).

$^{1}$H NMR (400 MHz, Chloroform-*d*) δ 10.98 (s, 6H), 10.04 (s, 1H), 8.15 (d, *J* = 6.6 Hz, 2H), 8.09-7.69 (m, 20H), 7.28 (t, *J* = 5.6 Hz, 4H), 7.15 (q, *J* = 6.6, 5.5 Hz, 2H), 6.98 (dt, *J* = 17.0, 5.5 Hz, 5H), 6.64 (tq, *J* = 15.3, 10.0, 7.8 Hz, 2H), 3.93 (m, 39H), 3.73 (t, *J* = 5.8 Hz, 5H).

**Methyl 4-chloro-2-hydroxy-3-methoxy-5-(4-(methoxycarbonyl)styryl)benzoate (10j-OMe)**

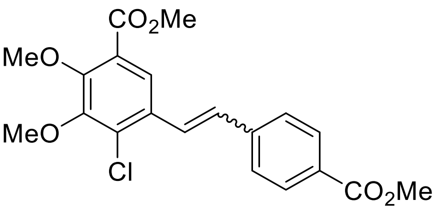


To a stirred solution of compound (0.1 mmol), in acetone (1 mL), was added $K_2CO_3$ (0.15 mmol), and methyl iodide (0.2 mmol) at room temperature. The reaction mixture was allowed to stir at the same temperature for 4 h. After consumption of starting material (monitored by TLC), diluted with water and ethyl acetate and aqueous layer was extracted with ethyl acetate

(3 X 5 mL) all organic layers were dried with $Na_2SO_4$ and concentrated on rotary evaporator. The crude material was purified by silica gel column chromatography with ethyl acetate and hexane as eluents.

Mp = 60-62 °C (colorless solid)

1H NMR (400 MHz, Chloroform-*d*) δ 7.96 (d, *J* = 8.4 Hz, 8H), 7.83 (s, 4H), 7.80 (d, *J* = 8.4 Hz, 2H), 7.52 (d, *J* = 8.4 Hz, 8H), 7.45 (d, *J* = 16.3 Hz, 4H), 7.28 (s, 1H), 7.13 (d, *J* = 8.3 Hz, 2H), 7.02 (d, *J* = 16.3 Hz, 4H), 6.67 (d, *J* = 12.3 Hz, 1H), 6.60 (d, *J* = 12.3 Hz, 1H), 3.92-3.83 (m, 54H), 3.81 (s, 3H), 3.70 (s, 3H).

13C NMR (101 MHz, Chloroform-*d*) δ 166.78 (2C), 165.73, 165.34, 153.52, 150.85, 150.80, 141.17, 140.78, 132.41, 131.92, 131.62, 131.42, 130.67, 130.09, 129.84, 129.62, 129.49, 128.96, 128.74, 128.22, 127.89, 127.00, 126.70, 126.04, 124.46, 124.19, 123.35, 61.96 (2C), 60.96, 60.90, 52.53, 52.31, 52.17, 52.10.

IR $\nu_{max}$ (Neat) 3068, 2961, 1688, 1608, 1590, 1488, 1437, 1317 $cm^{-1}$.

HRMS-EI (*m/z*): $[M]^+$ calcd for $C_{20}H_{19}ClO_6$, 390.0870; found, 390.0881.

**Scheme 4:**

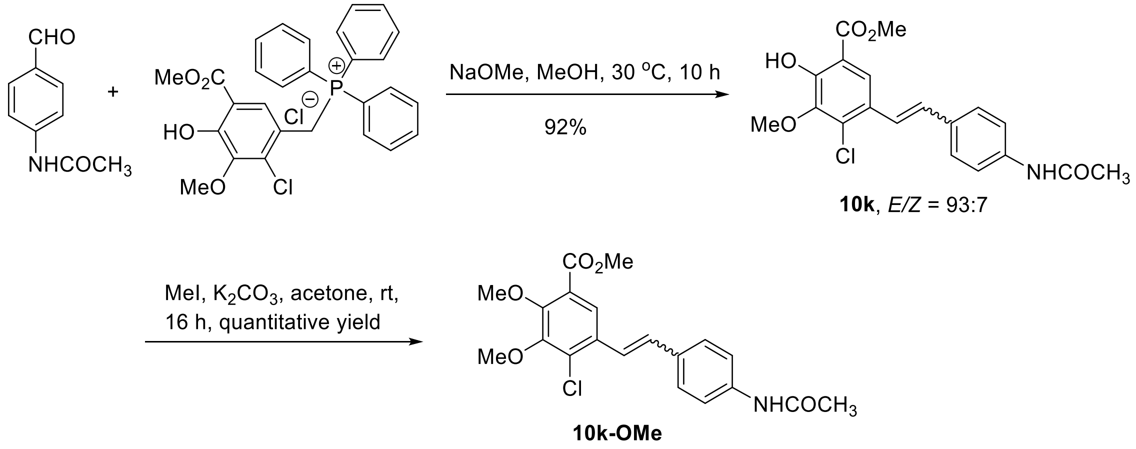


**Methyl 5-(4-acetamidostyryl)-4-chloro-2-hydroxy-3-methoxybenzoate (10k)**

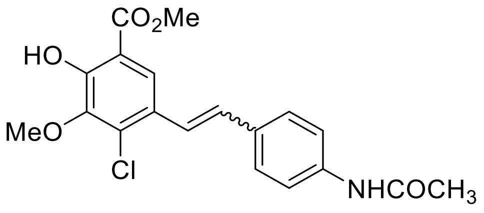

Compound **10k** (35 mg, 92%, *E/Z* = 93:7) has been prepared by following experimental procedure similar to **GP-2** from Wittig salt **2a** (63 mg, 0.12 mmol) and N-(4-formylphenyl)acetamide (16.3 mg, 0.1 mmol).

$^1$H NMR (400 MHz, Chloroform-*d*) δ 10.89 (s, 11H), 9.80 (s, 8H), 9.78 (s, 5H), 9.20 (s, 12H), 9.09 (s, 1H), 7.86 (s, 13H), 7.73 (q, *J* = 8.7 Hz, 35H), 7.21 (d, *J* = 16.2 Hz, 12H), 6.98 (d, *J* = 8.2 Hz, 2H), 6.87 (d, *J* = 16.2 Hz, 12H), 6.53 (d, *J* = 12.0 Hz, 1H), 6.37 (d, *J* = 12.0 Hz, 1H), 3.93 (s, 36H), 3.86 (s, 38H), 3.74 (d, *J* = 3.1 Hz, 7H), 3.69 (s, 5H), 2.11 (s, 21H), 2.09 (s, 34H), 2.06 (s, 2H), 2.05 (s, 3H).

**Methyl (*E*)-5-(4-acetamidostyryl)-4-chloro-2,3-dimethoxybenzoate (10k-OMe)**

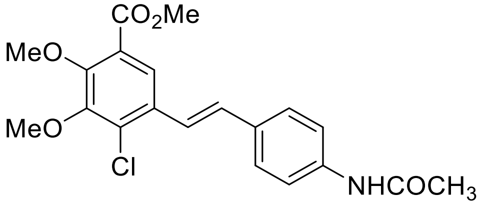


Mp = 115-117 $^{o}$C (colorless solid)

$^1$H NMR (400 MHz, Chloroform-*d*) δ 7.81 (s, 1H), 7.50-7.40 (m, 4H), 7.28 (d, *J* = 16.2 Hz, 2H), 7.28 (bs, 1H, NH), 6.96 (d, *J* = 16.2 Hz, 1H), 3.89 (s, 3H), 3.88 (s, 3H), 3.85 (s, 3H), 2.13 (s, 3H).

$^{13}$C NMR (101 MHz, Chloroform-*d*) δ 168.29, 165.90, 152.95, 150.71, 137.88, 132.88, 132.27, 131.12, 127.57, 127.37, 124.32, 123.01, 122.76, 119.89, 61.95, 60.88, 52.50, 24.72.

IR $\nu_{max}$ (Neat) 3694, 3431, 3065, 2938, 1729, 1695, 1607, 1592, 1519, 1468, 1315 cm$^{-1}$.

HRMS-ESI (*m/z*): [M]$^+$ calcd for $C_{20}H_{20}ClNO_5$, 389.1030; found, 389.1037.

**Methyl 5-(4-bromostyryl)-4-chloro-2-hydroxy-3-methoxybenzoate (10l)**

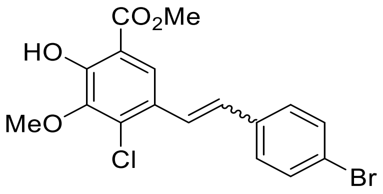


Compound **10l** (34 mg, 85%, *E/Z* = 93:7) has been prepared by following experimental procedure similar to **GP-2** from Wittig salt **2a** (63 mg, 0.12 mmol) and 4-bromobenzaldehyde (18.5 mg, 0.1 mmol).

Mp = 146-148 $^{o}$C (colorless crystalline solid).

$^1$H NMR (400 MHz, Chloroform-*d*) δ 10.92 (s, 1H), 10.90 (s, 1H), 7.85 (s, 1H), 7.41 (d, *J* = 8.1 Hz, 2H), 7.35-7.27 (m, 3H), 7.30 (d, *J* = 16.2 Hz, 1H), 7.25 (d, J = 8.5 Hz, 2H), 6.94 (d, *J* = 8.3 Hz, 2H), 6.84 (d, *J* = 16.2 Hz, 1H), 6.54 (d, *J* = 12.2Hz, 1H), 6.50 (d, *J* = 12.2 Hz, 1H), 3.93 (s, 3H), 3.87 (s, 3H), 3.86 (s, 3H), 3.76 (s, 3H).

$^{13}$C NMR (101 MHz, Chloroform-*d*) δ 170.13, 155.03, 145.05, 135.94, 134.08, 131.87, 129.13, 128.16, 127.22, 124.62, 121.77, 121.69, 112.11, 60.56, 52.76.

IR $\nu_{max}$ (Neat) 3684, 3060, 2980, 1685, 1605, 1488, 1444, 1350, 1055, 969 cm$^{-1}$.

HRMS-EI (*m/z*): $[M]^+$ calcd for $C_{17}H_{14}ClBrO_4$, 395.9764; found, 395.9768.

**Methyl 4-chloro-5-(4-chlorostyryl)-2-hydroxy-3-methoxybenzoate (10m)**

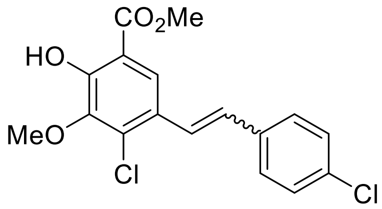


Compound **10m** (34 mg, 96%, *E/Z* = 83:17) has been prepared by following experimental procedure similar to **GP-2** from Wittig salt **2a** (63 mg, 0.12 mmol) and 4-chlorobenzaldehyde (14 mg, 0.1 mmol).

Mp = 134-136 °C (white crystalline solid)

$^1$H NMR (400 MHz, Chloroform-*d*) δ 10.91 (s, 5H), 10.90 (s, 1H), 7.84 (s, 5H), 7.44-7.34 (m, 10H), 7.31 (d, *J* = 12.3 Hz, 3H), 7.28-7.23 (m, 13H), 7.10 (d, *J* = 8.6 Hz, 2H), 7.00 (d, *J* = 8.6 Hz, 2H), 6.85 (d, *J* = 16.2 Hz, 5H), 6.55 (d, *J* = 12.0 Hz, 1H), 6.49 (d, *J* = 12.0 Hz, 1H), 3.93 (s, 15H), 3.87 (d, *J* = 2.1 Hz, 18H), 3.75 (s, 3H).

$^{13}$C NMR (101 MHz, Chloroform-*d*) δ 170.14, 170.06, 155.01, 154.79, 145.03, 135.50, 134.81, 134.07, 133.85, 133.65, 133.59, 133.05, 130.52, 130.08, 129.09, 128.92, 128.50, 127.86, 127.24, 127.05, 126.74, 125.53, 124.50, 121.67, 112.10, 111.85, 60.62, 60.55, 52.76, 52.62.

IR $\nu_{max}$ (Neat) 3684, 3050, 2940, 1685, 1603, 1513, 1469, 1326 cm$^{-1}$.

HRMS-EI (*m/z*): $[M]^+$ calcd for $C_{17}H_{14}Cl_2O_4$, 352.0269; found, 352.0282.

**Methyl 4-chloro-5-(4-fluorostyryl)-2-hydroxy-3-methoxybenzoate (10n**

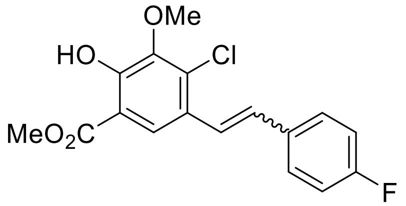


Compound **10n** (29 mg, 86%, *E/Z* = 95:5) has been prepared by following experimental procedure similar to **GP-2** from Wittig salt **2a** (63 mg, 0.12 mmol) and 4-fluorobenzaldehyde (12.4 mg, 0.1 mmol).

Mp = 95-97 $^o$C (light yellow solid)

$^1$H NMR (400 MHz, Chloroform-*d*) δ 11.02 (s, 1H), 10.90 (s, 19H), 7.84 (s, 19H), 7.60 (ddd, *J* = 12.0, 7.7, 1.6 Hz, 18H), 7.43 (dddd, *J* = 13.3, 7.8, 6.0, 2.4 Hz, 68H), 7.22 (d, *J* = 16.2 Hz, 23H), 7.09-7.02 (m, 3H), 6.98 (t, *J* = 8.7 Hz, 40H), 6.87 (d, *J* = 16.2 Hz, 21H), 6.82 (dt, *J* = 8.9, 3.3 Hz, 5H), 6.57 (d, *J* = 12.0 Hz, 1H), 6.46 (d, *J* = 12.0 Hz, 1H), 3.93 (s, 58H), 3.87 (s, 61H), 3.75 (s, 3H), 3.73 (s, 3H).

$^{13}$C NMR (101 MHz, Chloroform-*d*) δ 170.17, 162.54 (d, *J* = 247.9 Hz), 154.88, 145.0, 134.01, 133.20 (d, *J* = 3.3 Hz), 132.12 (d, *J* = 9.9 Hz), 131.97 (d, *J* = 2.7 Hz), 129.24, 128.53 (d, *J* = 12.2 Hz), 128.24 (d, *J* = 8.0 Hz), 127.45, 123.72 (d, *J* = 2.5 Hz), 121.59, 115.73 (d, *J* = 21.7 Hz), 112.08, 60.54, 52.74.

IR $\nu_{max}$ (Neat) 3690, 3060, 2957, 1681, 1601, 1510, 1445, 1345 cm$^{-1}$.

HRMS-EI (*m/z*): [M]$^+$ calcd for $C_{17}H_{14}ClFO_4$, 336.0565; found, 336.0576.

**Methyl 4-chloro-2-hydroxy-3-methoxy-5-(2-(pyridin-4-yl)vinyl)benzoate (10o)**

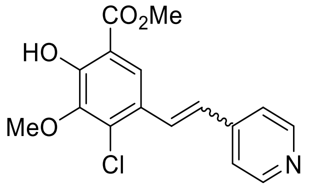


Compound **10o** (29 mg, 92%, *E/Z* = 83:17) has been prepared by following experimental procedure similar to **GP-2** from pyridine-4-carboxaldehyde (10.7 mg, 0.1 mmol). $R_f$ (80% ethyl acetate in hexanes) = 0.5.

Mp = 140-142 $^o$C (white solid).

$^1$H NMR (400 MHz, Chloroform-*d*) δ 11.00 (s, 5H), 10.96 (s, 1H), 8.53 (s, 10H), 8.38 (s, 2H), 7.90 (s, 5H), 7.65-7.57 (m, 5H), 7.53 (d, *J* = 16.2 Hz, 5H), 7.48 (s, 2H), 7.43-7.37 (m, 4H),

7.32 (d, $J$ = 5.3 Hz, 10H), 6.97 (d, $J$ = 5.1 Hz, 2H), 6.85 (d, $J$ = 16.2 Hz, 5H), 6.69 (d, $J$ = 12.0 Hz, 1H), 6.53 (d, $J$ = 12.0 Hz, 1H), 3.95 (s, 15H), 3.89 (s, 18H), 3.74 (s, 3H).

$^{13}$C NMR (101 MHz, Chloroform-*d*) δ 170.02, 169.92, 155.65, 155.24, 150.26, 149.92, 145.18, 144.25, 134.40, 132.16, 132.06, 131.94, 130.18, 129.15, 128.58, 128.46, 128.40, 127.63, 126.37, 125.42, 122.15, 121.00, 112.19, 111.89, 60.66, 60.60, 52.84, 52.70.

IR $\nu_{max}$ (Neat) 3670, 3055, 2950, 1685, 1616, 1450, 1357 cm$^{-1}$.

HRMS-EI (*m/z*): $[M]^+$ calcd for $C_{16}H_{14}ClNO_4$, 319.0611; found, 319.0609.

**Methyl 4-chloro-5-(3-chlorostyryl)-2-hydroxy-3-methoxybenzoate (10p)**

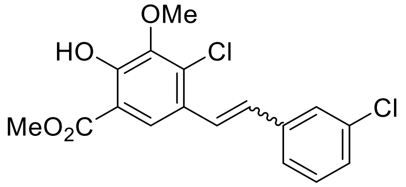


Compound **10p** (33 mg, 94%, *E/Z* = 74:26) has been prepared by following experimental procedure similar to **GP-2** from Wittig salt **2a** (63 mg, 0.12 mmol) and 3-chlorobenzaldehyde (14 mg, 0.1 mmol).

Mp = 108-110 $^{o}$C (colorless crystalline solid).

$^{1}$H NMR (400 MHz, Chloroform-*d*) δ 10.93 (s, 1H), 10.89 (s, 1H), 7.85 (s, 1H), 7.43 (s, 1H), 7.37-7.28 (m, 2H), 7.31 (d, $J$ = 16.2 Hz, 1H), 7.21 (m, 4H), 7.12-7.00 (m, 1H), 6.93 (d, $J$ = 7.4 Hz, 1H), 6.84 (d, $J$ = 16.2 Hz, 1H), 6.54 (s, 2H), 3.93 (s, 8H), 3.88 (s, 12H), 3.75 (s, 3H).

$^{13}$C NMR (101 MHz, Chloroform-*d*) δ 170.12, 170.05, 155.14, 154.87, 145.16, 145.06, 138.86, 138.22, 134.71, 134.16, 133.84, 133.65, 130.33, 129.96, 129.53, 128.94, 128.84, 128.54, 128.47, 127.87, 127.38, 127.07, 126.79, 126.58, 125.56, 125.32, 124.84, 121.78, 112.11, 111.78, 60.62, 60.56, 52.77, 52.57.

IR $\nu_{max}$ (Neat) 3690, 3087, 2990, 1685, 1600, 447, 1342, 1059, 965 cm$^{-1}$.

HRMS-EI (*m/z*): $[M]^+$ calcd for $C_{17}H_{14}Cl_2O_4$, 352.0269; found, 352.0276.

**Methyl 4-chloro-5-(3-fluorostyryl)-2-hydroxy-3-methoxybenzoate (10q)**

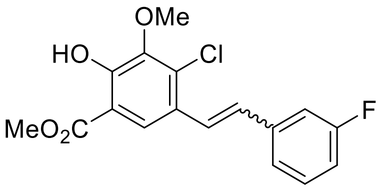

Compound **10q** (31 mg, 93%, *E/Z* = 74:26) has been prepared by following experimental procedure similar to **GP-2** from Wittig salt **2a** (63 mg, 0.12 mmol) and 3-fluorobenzaldehyde (12.4 mg, 0.1 mmol).

Mp = 75-76-148 °C (colorless crystalline solid).

$^1$H NMR (400 MHz, Chloroform-*d*) δ 10.93 (s, 1H), 10.89 (s, 1H), 7.86 (s, 1H), 7.36 (s, 1H), 7.32 (d, *J* = 16.2 Hz, 1H), 7.25 (dd, *J* = 5.3, 2.7 Hz, 2H), 7.23-7.19 (m, 2H), 7.15 (m, 1H), 7.09 (q, *J* = 7.7 Hz, 1H), 6.94-6.75 (m, 2H), 6.87 (d, *J* = 16.2 Hz, 1H), 6.57 (d, *J* = 12.1 Hz, 1H), 6.52 (d, *J* = 12.1 Hz, 1H), 3.93 (s, 3H), 3.88 (s, 6H), 3.74 (s, 3H).

$^{13}$C NMR (101 MHz, Chloroform-*d*) δ 170.14, 170.06, 163.18 (d, *J* = 245.6 Hz), 162.68 (d, *J* = 245.3 Hz), 155.12, 154.87, 145.15, 145.06, 139.34 (d, *J* = 7.7 Hz), 138.56 (d, *J* = 7.7 Hz), 134.16, 133.75 (d, *J* = 19.5 Hz), 130.56 (d, *J* = 2.3 Hz), 130.20 (d, *J* = 8.4 Hz), 129.78 (d, *J* = 8.4 Hz), 129.20 (d, *J* = 2.7 Hz), 128.64 (d, *J* = 18.8 Hz), 128.47, 127.17 (d, *J* = 16.3 Hz), 125.59, 125.26, 124.60 (d, *J* = 2.8 Hz), 122.58 (d, *J* = 2.7 Hz), 121.78, 115.41 (d, *J* = 22.0 Hz), 114.77 (d, *J* = 21.3 Hz), 114.25 (d, *J* = 21.2 Hz), 113.07 (d, *J* = 21.8 Hz), 112.11, 111.82, 60.62, 60.55, 52.76, 52.56.

IR $\nu_{max}$ (Neat) 3690, 3057, 2957, 1683, 1610, 1581, 1450, 1350, 1057, 965 cm$^{-1}$.

HRMS-EI (*m/z*): $[M]^+$ calcd for $C_{17}H_{14}ClFO_4$, 336.0565; found, 336.0564.

**Methyl 4-chloro-5-(3-cyanostyryl)-2-hydroxy-3-methoxybenzoate (10r)**

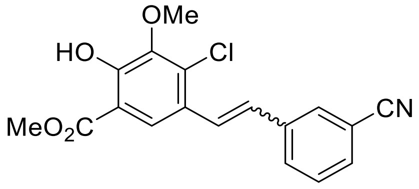

Compound **10r** (26 mg, 75%, *E/Z* = 76:24) has been prepared by following similar experimental procedure of **GP-2**, from Wittig salt **2a** (63 mg, 0.119 mmol) and 3-cyanobenzaldehyde (13.1 mg, 0.1 mmol).

Mp = 141-143 °C (colorless solid).

$^{1}$H NMR (400 MHz, $CDCl_3$) $\delta$: 11.04 (s, 1H), 10.99 (s, 1H), 7.94 (s, 1H), 7.76 (m, 3H), 7.67 (m, 1H), 7.56-7.54 (dt, $J$ = 7.64, 1.28 Hz, 1H), 7.50-7.48 (m, 1H), 7.47-7.46 (m, 2H), 7.45 (d, $J$ = 16.20 Hz, 1H), 7.36-7.33 (m, 1H), 6.95 (d, $J$ = 16.20 Hz, 1H), 6.70 (d, $J$ = 12.05 Hz, 1H), 6.65 (d, $J$ = 12.0 Hz, 1H), 4.02 (s, 3H), 3.96 (s, 3H), 3.95 (s, 3H), 3.82 (s, 3H).

$^{13}$C NMR (100 MHz, $CDCl_3$) $\delta$: 170.03, 169.86, 155.42, 155.12, 145.16, 138.26, 137.71, 134.24, 132.96, 132.28, 132.16, 131.04, 130.77, 130.54, 130.20, 129.57, 129.35, 129.13, 128.62, 128.46, 127.89, 126.61, 126.57, 126.24, 125.31, 121.88, 118.68, 118.41, 113.03, 112.56, 112.21, 60.64, 60.57, 52.80, 52.63.

IR $\nu_{max}$ (Neat) 3078, 3006, 2230, 1678, 1591, 1444, 1343, 1057, 961 cm$^{-1}$.

HRMS-EI (*m/z*): $[M]^+$ calcd for $C_{18}H_{14}ClNO_4$, 343.0611; found, 343.0610.

**Methyl 4-chloro-2-hydroxy-3-methoxy-5-styrylbenzoate (10s)**

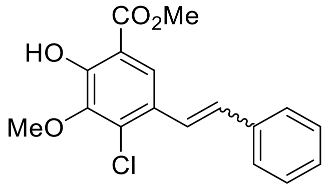


Compound **10s** (26 mg, 82%, *E/Z* = 73:27) has been prepared by following experimental procedure similar to **GP-2**, from Wittig salt **2a** (63 mg, 0.12 mmol) and benzaldehyde (10.6 mg, 0.1 mmol).

Colorless thick liquid.

$^{1}$H NMR (400 MHz, Chloroform-*d*) $\delta$ 10.90 (s, 2H), 10.87 (s, 1H), 7.87 (s, 1H), 7.46 (d, $J$ = 7.5 Hz, 2H), 7.36 (d, $J$ = 16.2 Hz, 1H), 7.33-7.29 (m, 2H), 7.27 (m, 2H), 7.24-7.20 (m, 3H), 7.12-7.06 (m, 3H), 6.92 (d, $J$ = 16.2 Hz, 1H), 6.61 (d, $J$ = 12.0 Hz, 1H), 6.48 (d, $J$ = 12.0 Hz, 1H), 3.93 (s, 3H), 3.88 (s, 6H), 3.72 (s, 3H).

$^{13}$C NMR (101 MHz, Chloroform-*d*) $\delta$ 170.22, 170.17, 154.86, 154.63, 145.04, 145.00, 137.13, 137.00, 134.09, 133.85, 131.83, 130.47, 128.76, 128.55, 128.48, 128.29, 128.02, 127.62, 127.50, 126.72, 125.99, 125.71, 123.95, 121.68, 112.08, 111.78, 60.60, 60.54, 52.73, 52.49.

IR $\nu_{max}$ (Neat) 3690, 3057, 2957, 1683, 1605, 1450, 1344, 1059, 965 cm$^{-1}$.

HRMS-EI (*m/z*): $[M]^+$ calcd for $C_{17}H_{15}ClO_4$, 318.0659; found, 318.0663.

**Methyl 5-(2-(allyloxy)styryl)-4-chloro-2-hydroxy-3-methoxybenzoate (10t)**

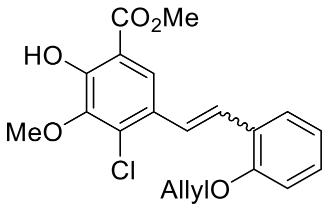


Compound **10t** (16.3 mg, 83%, *E/Z* = 73:27, 53% conversion) has been prepared by following experimental procedure similar to **GP-2**, from Wittig salt **2a** (63 mg, 0.12 mmol) and 2-(allyloxy)benzaldehyde (16.2 mg, 0.1 mmol).

Mp = 104-106 $^{o}$C (colorless crystalline solid).

$^{1}$H NMR (400 MHz, Chloroform-*d*) δ 10.92 (s, 1H), 10.89 (s, 3H), 7.90 (s, 3H), 7.77 (dd, *J* = 7.7, 1.8 Hz, 2H), 7.62 (s, 1H), 7.52 (dd, *J* = 7.7, 1.7 Hz, 3H), 7.45 (ddd, *J* = 8.8, 7.3, 1.8 Hz, 2H), 7.37 (d, *J* = 16.2 Hz, 4H), 7.28 (d, *J* = 16.3 Hz, 3H), 7.18-7.13 (m, 2H), 7.11-7.04 (m, 1H), 6.83 (d, *J* = 8.2 Hz, 3H), 6.80 (d, *J* = 11.8 Hz, 2H), 6.64 (t, *J* = 7.5 Hz, 1H), 6.55 (d, *J* = 12.1 Hz, 1H), 6.11-5.90 (m, 5H), 5.44-5.33 (m, 5H), 5.32-5.17 (m, 5H), 4.59 (dt, *J* = 5.2, 1.6 Hz, 4H), 4.56 (dt, *J* = 5.1, 1.6 Hz, 4H), 4.48 (dt, *J* = 5.1, 1.6 Hz, 2H), 3.92 (s, 8H), 3.88 (s, 3H), 3.87 (s, 8H), 3.69 (s, 3H), 3.38 (s, 3H).

$^{13}$C NMR (101 MHz, Chloroform-*d*) δ 170.30, 160.95, 156.07, 154.67, 135.87, 134.07, 133.38, 133.28, 132.40, 129.80, 128.97, 128.62, 128.46, 128.33, 127.23, 127.20, 126.43, 125.93, 125.69, 125.57, 124.64, 124.27, 121.86, 121.09, 120.88, 120.48, 118.10, 117.40, 117.30, 112.84, 112.49, 112.22, 112.06, 69.27, 69.17, 60.52, 58.58, 52.69, 52.59.

IR $\nu_{max}$ (Neat) 3690, 3055, 2942, 1681, 1601, 1486, 1453, 1350, 1095, 965 cm$^{-1}$.

HRMS-EI (*m/z*): [M]$^{+}$ calcd for $C_{20}H_{19}ClO_5$, 374.0921; found, 374.0932.

**Methyl 4-chloro-2-hydroxy-3-methoxy-5-(3-methoxystyryl)benzoate (10u)**

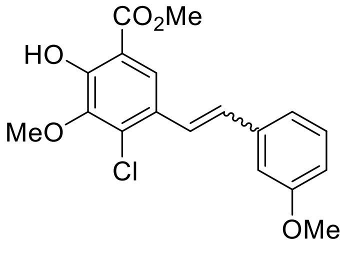


Compound **10u** (29 mg, 84%, *E/Z* = 74:26) has been prepared by following experimental procedure similar to **GP-2**, from Wittig salt **2a** (63 mg, 0.12 mmol) and 3-methoxybenzaldehyde (13.6 mg, 0.1 mmol).

Mp = 86-90 °C (colorless crystalline solid).

$^1$H NMR (400 MHz, Chloroform-*d*) δ 10.91 (s, 3H), 10.87 (s, 1H), 7.86 (s, 3H), 7.44-7.36 (m, 3H), 7.31 (d, *J* = 16.2 Hz, 3H), 7.25-7.17 (m, 3H), 7.06 (d, *J* = 7.7 Hz, 3H), 6.99 (t, *J* = 2.0 Hz, 3H), 6.89 (d, *J* = 16.2 Hz, 3H), 6.77 (dd, *J* = 8.2, 2.5 Hz, 3H), 6.65 (ddd, *J* = 13.6, 4.7, 2.2 Hz, 3H), 6.58 (d, *J* = 12.1 Hz, 1H), 6.48 (d, *J* = 12.1 Hz, 1H), 3.93 (s, 8H), 3.87 (s, 8H), 3.79 (s, 3H), 3.78 (s, 8H), 3.74 (s, 3H), 3.59 (s, 3H).

$^{13}$C NMR (101 MHz, Chloroform-*d*) δ 170.20, 170.15, 159.92, 159.41, 154.89, 154.64, 145.01, 144.99, 138.44, 137.64, 134.09, 131.72, 130.33, 130.06, 129.73, 129.29, 127.50, 126.21, 125.73, 124.26, 123.61, 121.71, 121.58, 121.37, 119.41, 113.57, 113.31, 112.08, 112.03, 60.59, 60.54, 55.30, 55.08, 52.73, 52.52.

IR $\nu_{max}$ (Neat) 3690, 3054, 2958, 1683, 1600, 1510, 1445, 1345, 1057, 964 cm$^{-1}$.

HRMS-EI (*m/z*): $[M]^+$ calcd for $C_{18}H_{17}ClO_5$, 348.0765; found, 348.0759.

**Scheme 5:**

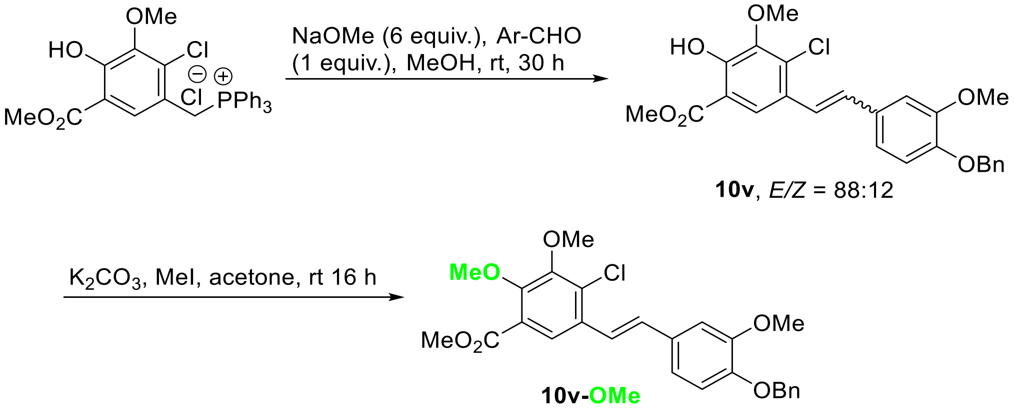


**Methyl (E)-5-(4-(benzyloxy)-3-methoxystyryl)-4-chloro-2-hydroxy-3-methoxybenzoate (10v)**

Compound **10v** (39 mg, 85%, *E/Z* = 88:12) has been prepared by following experimental procedure similar to **GP-2**, from Wittig salt **2a** (63 mg, 0.12 mmol) and 4-(benzyloxy)-3-methoxybenzaldehyde (24.2 mg, 0.1 mmol).

Mp = 125-127 °C (colorless solid).

$^1$H NMR (400 MHz, Chloroform-*d*) δ 10.87 (s, 8H), 9.75 (s, 6H), 7.82 (s, 7H), 7.47 (s, 1H), 7.41–7.19 (m, 78H), 7.15 (d, *J* = 16.1 Hz, 7H), 7.04-6.88 (m, 20H), 6.83 (d, *J* = 16.1 Hz, 7H), 6.79 (d, *J* = 8.3 Hz, 7H), 6.50 (d, *J* = 12.0 Hz, 1H), 6.36 (d, *J* = 12.0 Hz, 1H), 5.16 (s, 11H), 5.10 (s, 14H), 5.03 (s, 2H), 3.91 (s, 22H), 3.86 (d, *J* = 2.9 Hz, 61H), 3.74 (s, 3H), 3.55 (s, 3H).

$^{13}$C NMR (101 MHz, Chloroform-*d*) δ 170.23, 170.15, 154.62, 154.53, 149.79, 149.02, 148.34, 147.61, 144.96, 143.77, 136.97, 136.01, 134.48, 133.89, 132.27, 131.49, 130.60, 130.26, 129.51, 128.76, 128.61, 128.57, 127.92, 127.90, 127.82, 127.26, 127.23, 125.66, 124.50, 122.20, 121.93, 121.48, 119.95, 113.91, 113.41, 112.06, 111.75, 109.61, 70.99, 70.84, 60.61, 60.54, 56.08, 55.65, 52.72, 52.62.

**10v-OMe data:**

$^{1}$H NMR (400 MHz, Chloroform-*d*) δ 7.80 (s, 1H), 7.38 (d, *J* = 7.0 Hz, 2H), 7.30 (t, *J* = 7.4 Hz, 2H), 7.25 (d, *J* = 7.2 Hz, 1H), 7.19 (d, *J* = 16.1 Hz, 1H), 7.00 (m, 2H), 6.93 (d, *J* = 16.1 Hz, 1H), 6.81 (d, *J* = 8.3 Hz, 1H), 5.12 (s, 2H), 3.89 (s, 6H), 3.88 (s, 3H), 3.85 (s, 3H).

$^{13}$C NMR (101 MHz, Chloroform-*d*) δ 165.93, 152.79, 150.69, 149.79, 148.55, 136.94, 132.43, 131.67, 130.36, 128.62, 127.93, 127.25, 124.31, 122.95, 121.80, 120.19, 113.88, 109.75, 70.98, 61.95, 60.88, 56.09, 52.50.

IR $\nu_{max}$ (Neat) 3687, 3057, 2989, 1685, 1605, 1514, 1443, 1346 cm$^{-1}$.

HRMS-ESI (*m/z*): [M+H]$^{+}$ calcd for $C_{25}H_{24}ClO_6$, 455.1256; found, 455.1256.

**Methyl (E)-4-chloro-5-(2,6-dibromostyryl)-2-hydroxy-3-methoxybenzoate (10w)**

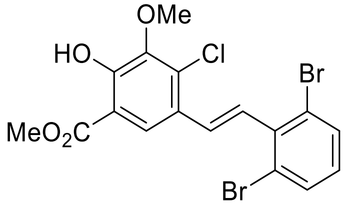


Compound **10w** (45 mg, 95%, only *E*-isomer) has been prepared by following experimental procedure similar to **GP-2**, from Wittig salt **2a** (63 mg, 0.12 mmol) and 2,6-dibromobenzaldehyde (26.4 mg, 0.1 mmol).

Mp = 145-147 $^{o}$C (white solid).

$^{1}$H NMR (500 MHz, Chloroform-*d*) δ 10.99 (s, 1H), 7.92 (s, 1H), 7.52 (d, *J* = 8.0 Hz, 2H), 7.21 (d, *J* = 16.5 Hz, 1H), 6.91 (t, *J* = 8.0 Hz, 1H), 6.82 (d, *J* = 16.5 Hz, 1H), 3.94 (s, 3H), 3.88 (s, 3H).

$^{13}$C NMR (126 MHz, Chloroform-*d*) δ 170.19, 155.46, 145.09, 137.72, 133.65, 132.42, 129.30, 128.48, 126.87, 124.80, 124.11, 122.14, 112.15, 60.55, 52.80.

IR $\nu_{max}$ (Neat) 3690, 3057, 2961, 1681, 1605, 1545, 1448, 1350, 1057, 969 cm$^{-1}$.

HRMS-EI (*m/z*): [M]$^{+}$ calcd for $C_{17}H_{13}ClBr_2O_4$, 473.8869; found, 473.8862.

**Methyl 4-chloro-5-(2,6-difluorostyryl)-2-hydroxy-3-methoxybenzoate (10x)**

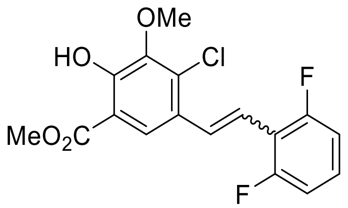


Compound **10x** (28 mg, 80%, *E/Z* = 89:11) has been prepared by following experimental procedure similar to **GP-2**, from Wittig salt **2a** (63 mg, 0.12 mmol) and 2-6-difluorobenzaldehyde (14.2 mg, 0.1 mmol).

Mp = 125-127 $^oC$ (colorless crystalline solid).

$^1H$ NMR (400 MHz, Chloroform-*d*) δ 10.96 (s, 8H), 10.84 (s, 1H), 7.88 (s, 8H), 7.65 (d, *J* = 16.6 Hz, 8H), 7.11 (m, 10H), 6.92 (d, *J* = 16.6 Hz, 8H), 6.87 (d, *J* = 11.9 Hz, 1H), 6.85 (t, *J* = 8.5 Hz, 16H), 6.73 (t, *J* = 7.9 Hz, 2H), 6.42 (d, *J* = 11.9 Hz, 1H), 3.93 (s, 24H), 3.87 (s, 24H), 3.86 (s, 3H), 3.68 (s, 3H).

$^{13}C$ NMR (101 MHz, Chloroform-*d*) δ 170.21, 161.02 (dd, $J_{C-F}$ = 251.9, 7.5 Hz), 155.30, 145.03, 130.50 (t, *J* = 8.7 Hz), 128.37, 128.26, 127.91, 121.81, 116.89, 112.13, 111.66 (d, $J_{CH-CF}$ = 26.2 Hz), 111.66 (d, $J_{C-CF}$ = 13.2 Hz), 60.55, 52.76.

$^{19}F$ NMR (376 MHz, Chloroform-*d*) δ -110.24, -112.85.

IR $\nu_{max}$ (Neat) 3690, 3052, 2961, 1685, 1625, 1584, 1470, 1350, 1061, 1006 $cm^{-1}$.

HRMS-EI (*m/z*): $[M]^+$ calcd for $C_{17}H_{13}ClF_2NO_6$, 354.0470; found, 354.0476.

**Methyl 4-chloro-5-(2,4-dichlorostyryl)-2-hydroxy-3-methoxybenzoate (10y)**

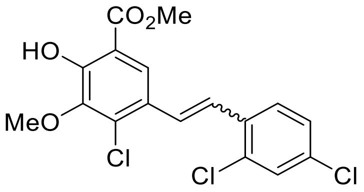


Compound **10y** (35 mg, 91%, *E/Z* = 13:87) has been prepared by following experimental procedure similar to **GP-2**, from Wittig salt **2a** (63 mg, 0.12 mmol) and 5-bromo-2-nitrobenzaldehyde (17.5 mg, 0.1 mmol).

**Data for *E*-isomer of 10y:**

Mp = 78-81$^oC$ (white solid).

$^1H$ NMR (400 MHz, $CDCl_3$) *δ:* 10.95 (s, 1H), 7.41 (d, *J* = 2.0 Hz, 1H), 7.24 (s, 1H), 6.99 (d, *J* = 2.0 Hz, 1H), 6.96 (s, 1H), 6.76 (s, 2H), 3.94 (s, 3H), 3.82 (s, 3H).

$^{13}$C NMR (100 MHz, $CDCl_3$) $\delta$: 169.9, 154.8, 145.0, 134.4, 134.3, 133.6, 133.5, 131.1, 129.3, 128.4, 127.6, 126.8, 126.2, 125.5, 111.7, 60.5, 52.6.

**Data for *Z*-isomer of 10y:**

Mp = 68-71 $^{o}$C (white solid).

$^{1}$H NMR (400 MHz, $CDCl_3$) $\delta$: 11.06 (s, 1H), 7.97 (s, 1H), 7.63 (d, $J$ = 8.8 Hz, 1H), 7.42 (d, $J$ = 2.0 Hz, 1H), 7.32 (d, $J$ = 12.2 Hz, 2H), 7.27-7.29 (m, 1H), 4.02 (s, 3H), 3.96 (s, 3H).

$^{13}$C NMR (100 MHz, $CDCl_3$) $\delta$: 170.1, 155.4, 145.1, 133.9, 129.6, 127.6, 127.5, 127.1, 127.0, 125.3, 122.1, 112.2, 60.6, 52.8.

IR $\nu_{max}$ (Neat) 3097, 3003, 2948, 2855, 1678, 1587, 1444, 1388, 1340, 1247, 1054, 960 cm$^{-1}$.

HRMS-ESI (*m/z*): [M+H]$^+$ calcd for $C_{17}H_{14}Cl_3O_4$, 386.9958; found, 386.9948.

**Methyl 4-chloro-2-hydroxy-3-methoxy-5-(2,4,6-tribromo-3-methoxystyryl)benzoate (10z)**

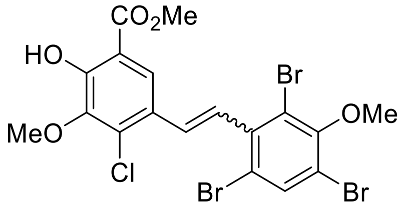


Compound **10z** (46 mg, 79%, *E/Z* = 91:9) has been prepared by following experimental procedure similar to **GP-2**, from Wittig salt **2a** (63 mg, 0.12 mmol) and 2,4,6-tribromo-3-methoxybenzaldehyde (37.3 mg, 0.1 mmol).

Mp = 131-133 $^{o}$C (colorless solid).

$^{1}$H NMR (400 MHz, $CDCl_3$) $\delta$: 10.99 (s, 1H), 10.77 (s, 1H), 7.91 (s, 1H), 7.76 (s, 1H), 7.66 (s, 1H), 7.17 (d, $J$ = 15.9 Hz, 1H), 6.93 (s, 1H), 6.83 (d, $J$ = 11.8 Hz, 1H), 6.73 (d, $J$ = 16.4 Hz, 1H), 6.35 (d, $J$ = 11.7 Hz, 1H), 3.94 (s, 3H), 3.88 (s, 3H), 3.83 (s, 3H), 3.82 (s, 3H), 3.76 (s, 3H), 3.70 (s, 3H).

$^{13}$C NMR (100 MHz, $CDCl_3$) $\delta$: 170.12, 169.83, 155.59, 154.13, 146.15, 145.16, 140.59, 138.72, 135.76, 135.56, 134.38, 132.97, 129.56, 128.92, 128.03, 126.59, 124.40, 122.12, 120.60, 119.77, 118.47, 116.81, 114.86, 113.70, 112.17, 110.37, 60.54, 60.48, 52.78, 52.53.

IR $\nu_{max}$ (Neat) 3310, 2941, 1679, 1446, 1347, 1243, 1149, 1058, 960 cm$^{-1}$

HRMS-ESI (*m/z*): [M+H]$^+$ calcd for $C_{18}H_{15}Br_3ClO_5$, 582.8158; found, 582.8156.

**Methyl 4-chloro-2-hydroxy-3-methoxy-5-(2,3,4-tribromo-5-methoxystyryl)benzoate (10aa)**

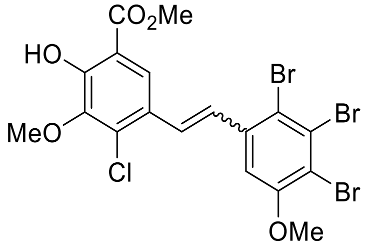


Compound **10aa** (44 mg, 75%, *E/Z* = 88:12) has been prepared by following experimental procedure similar to **GP-2**, from Wittig salt **2a** (63 mg, 0.12 mmol) and 2,3,4-tribromo-5-methoxybenzaldehyde (37.3 mg, 0.1 mmol).

Mp = 168-170 $^{o}$C (colorless solid).

$^{1}$H NMR (400 MHz, $CDCl_3$) *δ:* 11.01 (s, 1H), 10.86 (s, 1H), 7.89 (s, 1H), 7.24 (d, *J* = 16.04 Hz, 1H), 7.19 (s, 1H), 7.13 (s, 2H), 7.03 (s, 1H) 6.70 (d, *J* = 12.4 Hz, 1H), 6.65 (d, *J* = 12.3 Hz, 1H), 6.44 (s, 1H), 3.95 (s, 1H), 3.90 (s, 1H), 3.88 (s, 1H), 3.85 (s, 3H), 3.76 (s, 3H), 3.46 (s, 3H).

$^{13}$C NMR (100 MHz, $CDCl_3$) *δ:* 170.01, 169.82, 155.82, 155.54, 154.87, 145.00, 138.58, 134.21, 131.95, 130.41, 130.40, 129.34, 129.33 128.22, 128.18, 125.99, 125.66, 122.33, 122.32, 117.79, 114.99, 111.85, 111.80, 108.42, 60.61, 60.58, 56.89, 56.55, 52.86, 52.78.

IR $\nu_{max}$ (Neat) 3104, 3006, 2942, 2852, 1678, 1568, 1446, 1409, 1343, 1244, 1149, 955 $cm^{-1}$.

HRMS-ESI (*m/z*): $[M+NH_4]^+$ calcd for $C_{18}H_{18}Br_3ClNO_5$, 599.8424; found, 599.8453.

**Scheme 6: Synthesis of 2-aryl indoles**

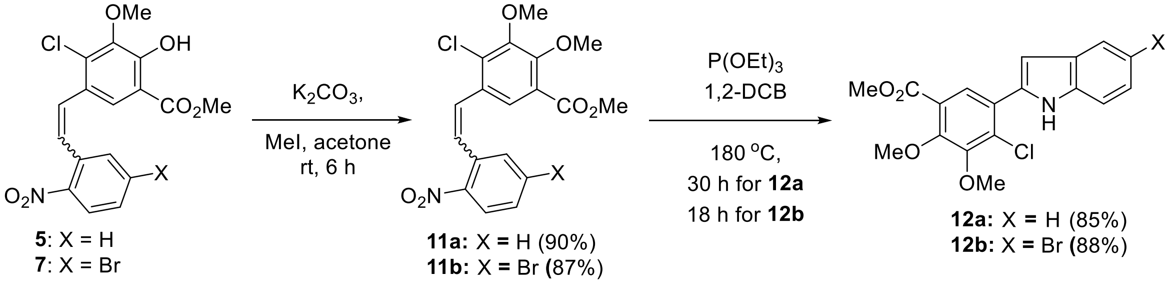


## General procedure-4 (GP-4).

To a stirred solution of **5** or **7** (0.1 mmol) in dry acetone (4 mL) was added the solid of $K_2CO_3$ (0.3 mmol) in one portion followed by the addition of methyl iodide (0.2 mmol) at 30 $^{o}$C. The reaction mixture was stirred at the same temperature over a period of under inert atmosphere. After 5 h, the solvent was removed under reduced pressure, the mass was diluted with water

and ethyl acetate. Aqueous layer was extracted with ethyl acetate (3 x 6 mL), dried with $Na_2SO_4$ and concentrated under reduced pressure. The crude was purified by column chromatography with ethyl acetate in hexanes as eluents to obtain the desired compounds.

**General procedure-5: for Cadogan-Sundberg reductive cyclization 12a-b (GP-5)**[4]

A solution of stilbene derivative (37.7 mg, 0.1 mmol) and $P(OEt)_3$ (39 mg, 0.233 mmol) in *o*-DCB (1 mL) was heated to reflux with vigorous stirring. After consumption of starting material (monitored by TLC), the solvent was removed under high vacuum (10 mm Hg, 150 °C). The reaction was cooled to room temperature and purified by silica gel column chromatography with ethyl acetate and hexanes as eluents to obtain the desired 2-arylindole derivatives. $R_f$ (20% ethyl acetate in hexanes) = 0.5.

**Methyl 4-chloro-2,3-dimethoxy-5-(2-nitrostyryl)benzoate (11a)**

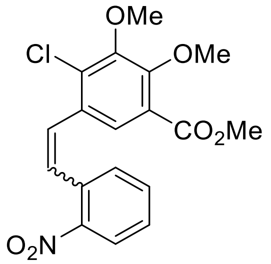


Compound **11a** prepared from compound **3** (36.3 mg, 0.100 mmol) by following a similar experimental procedure **GP-4**. The crude products were purified by silica gel column chromatography by using 12-16% ethyl acetate in hexanes to provide the compound **11a** in good yield (34 mg, 90%). $R_f$ (15% ethyl acetate in hexanes) = 0.5.

Yellow liquid.

$^1$H NMR (400 MHz, $CDCl_3$) $\delta$: 8.10-8.07 (m, 1H), 8.00 (dd, $J$ = 8.2, 1.2 Hz, 1H), 7.88 (s, 1H), 7.76 (d, $J$ = 7.7 Hz, 1H), 7.64 (td, $J$ = 7.9, 1.0 Hz, 1H), 7.55 (d, $J$ = 16.0 Hz, 1H), 7.45 (td, $J$ = 8.4, 1.3 Hz, 1H), 7.41-7.36 (m, 3H), 7.12-7.10 (m, 1H), 7.07 (d, $J$ = 11.9 Hz, 1H), 7.06 (s, 1H), 6.83 (d, $J$=11.8 Hz, 1H), 3.97 (s, 3H), 3.95 (s, 3H), 3.93 (s, 3H), 3.90 (s, 6H), 3.73 (s, 3H).

$^{13}$C NMR (100 MHz, $CDCl_3$) $\delta$: 165.46, 165.14, 153.80, 153.21, 150.75, 150.70, 148.11, 148.00, 133.33, 133.20, 132.68, 132.63, 132.60, 132.33, 131.86, 131.29, 131.01, 129.25, 128.74, 128.72, 128.58, 128.44, 127.71, 127.31, 126.90, 124.84, 124.75, 123.91, 123.66, 61.96, 61.86, 60.89, 52.49, 52.18.

IR $\nu_{max}$ (Neat) 2929, 2855, 1731, 1522, 1464, 1340, 1249, 1053, 957 cm$^{-1}$.

HRMS-ESI (*m/z*): $[M+H]^+$ calcd for $C_{18}H_{17}ClNO_6$, 378.0744; found, 378.0737.

**Methyl 5-(5-bromo-2-nitrostyryl)-4-chloro-2,3-dimethoxybenzoate (11b)**

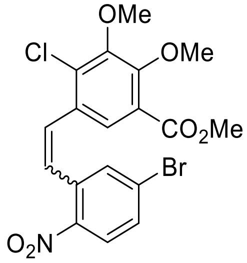


Compound **11b** prepared from compound **7** (44.2 mg, 0.100 mmol) by following a similar experimental procedure **GP-4**. The crude products were purified by silica gel column chromatography by using 15-18% ethyl acetate in hexanes to provide the compound **11b** in good yield (40 mg, 87%). $R_f$ (16% ethyl acetate in hexanes) = 0.5.

Mp = 97-100 $^oC$ (light yellow solid).

$^1H$ NMR (400 MHz, $CDCl_3$) $\delta$: 7.88 (d, $J$ = 8.5 Hz, 1H), 7.84-7.79 (m, 1H), 7.49 (t, $J$ = 8.3 Hz, 1H), 7.43 (d, $J$ = 8.8 Hz, 1H), 7.29 (d, $J$ = 15.7 Hz, 1H), 7.19 (d, $J$ = 15.7 Hz, 1H), 7.15 (s, 2H), 7.02 (s, 2H), 6.94 (d, $J$ = 11.8 Hz, 1H), 6.80 (d, $J$ = 11.8 Hz, 1H), 3.94 (s, 3H), 3.90 (s, 3H), 3.88 (s, 3H), 3.84 (s, 6H), 3.69 (s, 3H).

$^{13}C$ NMR (100 MHz, $CDCl_3$) $\delta$: 165.11, 165.01, 153.63, 150.92, 146.84, 134.51, 134.42, 132.65, 131.52, 130.44, 128.81, 128.00, 127.94, 127.21, 126.24, 123.97, 61.93, 60.98, 52.27.

IR $\nu_{max}$ (Neat) 2926, 2854, 1730, 1464, 1337, 1248, 1053, 968 $cm^{-1}$.

HRMS-ESI (*m/z*): $[M+H]^+$ calcd for $C_{18}H_{16}BrClNO_6$, 455.9850; found, 455.9857.

**Methyl 4-chloro-5-(1H-indol-2-yl)-2,3-dimethoxybenzoate (12a)**

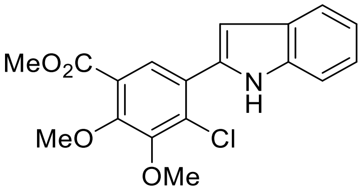


Compound **12a** (30 mg, 85%) has been prepared by following the experimental procedure similar to **GP-5** from compound **11a** (37.7 mg, 0.100 mmol).

Mp = 116-118 $^oC$ (light yellow solid).

$^1H$ NMR (400 MHz, $CDCl_3$) $\delta$: 8.68 (br, 1H), 7.86 (s, 1H), 7.66 (d, $J$ = 7.8 Hz, 1H), 7.43 (d, $J$ = 8.5 Hz, 1H), 7.23 (dd, $J$ = 8.1, 1.0 Hz, 1H), 7.14 (td, $J$ = 7.4, 1.0 Hz, 1H), 6.88 (d, $J$ = 1.6 Hz, 1H), 4.00 (s, 3H), 3.96 (s, 3H), 3.94 (s, 3H).

$^{13}C$ NMR (100 MHz, $CDCl_3$) *δ:* 165.46, 153.42, 151.34, 136.38, 133.82, 130.74, 128.12, 128.01, 127.09, 124.73, 122.87, 120.88, 120.31, 111.02, 104.12, 61.96, 60.96, 52.53.

IR $\nu_{max}$ (Neat) 3344, 2975, 2823, 1778, 1512, 1434, 1253, 1218, 1178, 1082, 970 $cm^{-1}$.

HRMS-ESI (*m/z*): $[M+H]^+$ calcd for $C_{18}H_{17}ClNO_4$, 346.0841; found, 346.0840.

**Methyl 5-(5-bromo-1H-indol-2-yl)-4-chloro-2,3-dimethoxybenzoate (12b)**

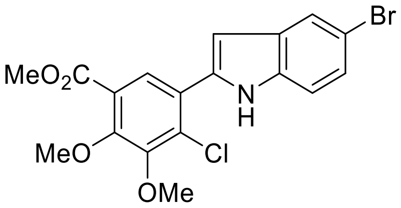


Compound **12b** (38 mg, 88%) has been prepared by following the experimental procedure similar to **GP-5**, from compound **11b** (45.6 mg, 0.100 mmol).

Yellow color solid, Mp = 122-124 $^{o}C$.

$^{1}H$ NMR (400 MHz, $CDCl_3$) *δ:* 8.78 (s, 1H), 7.83 (s, 1H), 7.77 (s, 1H), 7.31-7.27 (m, 2H), 6.79 (d, *J* = 2.0 Hz, 1H), 3.99 (s, 3H), 3.95 (s, 3H), 3.94 (s, 3H).

$^{13}C$ NMR (100 MHz, $CDCl_3$) *δ:* 165.43, 153.69, 151.35, 135.03, 134.94, 130.86, 129.82, 127.45, 127.14, 125.66, 124.75, 123.27, 113.43, 112.49, 103.50, 61.98, 60.99, 52.61.

IR $\nu_{max}$ (Neat) 3358, 2927, 2853, 1718, 1516, 1460, 1413, 1307, 1242, 1120, 1041, 883 $cm^{-1}$.

HRMS-ESI (*m/z*): $[M+H]^+$ calcd for $C_{18}H_{16}BrClNO_4$, 423.9951; found, 423.9940.

## Crystallographic data for compounds 2a, 4a, 4b, 5b, 6b and 12b

### X-Ray Structure Determination data of 2a

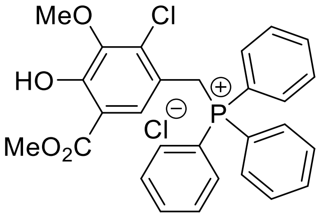


**2a**

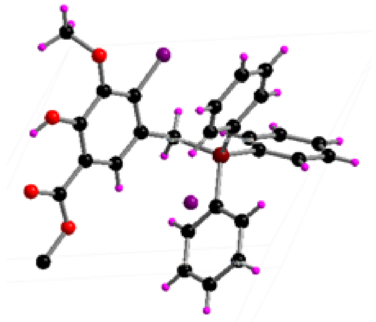

**x-ray of 2a**

**Crystal Data** for $C_{28}H_{27}Cl_2O_5P$ ($M$ =545.402 g/mol): triclinic, space group P-1 (no. 2), $a$ = 10.5392(8) Å, $b$ = 11.9681(7) Å, $c$ = 12.2460(8) Å, $\alpha$ = 91.566(5)°, $\beta$ = 107.740(6)°, $\gamma$ = 111.461(6)°, $V$ = 1352.30(18) Å$^3$, $Z$ = 2, $T$ = 300 K, μ(Cu Kα) = 3.019 mm$^{-1}$, $Dcalc$ = 1.339 g/cm$^3$, 8310 reflections measured (7.68° ≤ 2Θ ≤ 140°), 4865 unique ($R_{int}$ = 0.0279, $R_{sigma}$ = 0.0430) which were used in all calculations. The final $R_1$ was 0.0520 (I>=2u(I)) and $wR_2$ was 0.2296 (all data).

Single crystal X-ray data for **2a** compound was collected at room temperature. A suitable crystal of **2**a was selected and mounted on the SuperNova (Dual, Cu at zero, Eos) diffractometer. The crystal was kept at 298 K during data collection. Using Olex2,[5] the structure was solved with the olex2.solve[6] structure solution program using Charge Flipping and refined with the olex2.refine[7] refinement package using the Gauss-Newton minimization. The absorption corrections were performed on the basis of multi-scans. The non-hydrogen atoms were anisotropically refined. The hydrogen atoms were included in the refinement at the calculated positions riding on their carrier atoms.

Table 1 Crystal data and structure refinement for **2a**.

| | |
|---|---|
| Identification code | exp_8799 |
| Empirical formula | $C_{28}H_{27}Cl_2O_5P$ |
| Formula weight | 545.402 |
| Temperature/K | 300 |
| Crystal system | triclinic |
| Space group | P-1 |
| a/Å | 10.5392(8) |
| b/Å | 11.9681(7) |
| c/Å | 12.2460(8) |
| α/° | 91.566(5) |
| β/° | 107.740(6) |
| γ/° | 111.461(6) |
| Volume/Å$^3$ | 1352.30(18) |
| Z | 2 |

| | |
|---|---|
| $\rho_{calc}$g/cm$^3$ | 1.339 |
| μ/mm$^{-1}$ | 3.019 |
| F(000) | 571.7 |
| Crystal size/mm$^3$ | 0.04 × 0.04 × 0.02 |
| Radiation | Cu Kα (λ = 1.54184) |
| 2Θ range for data collection/° | 7.68 to 140 |
| Index ranges | -12 ≤ h ≤ 12, -14 ≤ k ≤ 12, -10 ≤ l ≤ 14 |
| Reflections collected | 8310 |
| Independent reflections | 4865 [$R_{int}$ = 0.0279, $R_{sigma}$ = 0.0430] |
| Data/restraints/parameters | 4865/0/332 |
| Goodness-of-fit on $F^2$ | 0.998 |
| Final R indexes [I>=2σ (I)] | $R_1$ = 0.0520, $wR_2$ = 0.1657 |
| Final R indexes [all data] | $R_1$ = 0.0709, $wR_2$ = 0.2296 |
| Largest diff. peak/hole / e Å$^{-3}$ | 0.44/-0.67 |

Table 2 Fractional Atomic Coordinates (×104) and Equivalent Isotropic Displacement Parameters (Å2×103) for **2a**. Ueq is defined as 1/3 of the trace of the orthogonalised UIJ tensor.

| **Atom** | **x** | **y** | **z** | **U(eq)** |
|---|---|---|---|---|
| P1 | 3762.2(10) | 2277.2(7) | 2987.8(7) | 36.0(3) |
| Cl1 | 7238.2(12) | 4496.2(9) | 5497.0(9) | 57.4(3) |
| O4 | 9382(3) | 3581(2) | 6646(2) | 49.4(6) |
| O3 | 9957(3) | 1828(3) | 5747(2) | 56.9(7) |
| O2 | 7625(3) | 155(2) | 2286(2) | 51.0(7) |
| O1 | 9456(4) | 348(3) | 3897(3) | 60.8(8) |
| C3 | 8647(4) | 3055(3) | 5515(3) | 40.6(8) |
| C6 | 7071(4) | 1953(3) | 3225(3) | 37.8(7) |
| C7 | 8469(4) | 641(3) | 3368(3) | 40.5(8) |
| C1 | 8129(4) | 1583(3) | 3869(3) | 39.6(8) |
| C4 | 7550(4) | 3372(3) | 4845(3) | 40.0(8) |
| C23 | 3878(4) | 2151(3) | 4472(3) | 41.1(8) |
| C10 | 5547(4) | 3198(3) | 2962(3) | 38.4(7) |
| C11 | 3130(4) | 772(3) | 2225(3) | 41.1(8) |
| C2 | 8926(4) | 2128(3) | 5035(3) | 40.2(8) |
| C5 | 6724(4) | 2829(3) | 3685(3) | 37.8(7) |
| C17 | 2573(4) | 3013(3) | 2338(3) | 37.6(7) |
| C24 | 4572(4) | 1439(3) | 5056(3) | 46.8(9) |

| | | | | |
|---|---|---|---|---|
| C18 | 3015(5) | 4026(4) | 1816(4) | 53.1(10) |
| C21 | 205(5) | 3055(4) | 1830(4) | 58.6(11) |
| C28 | 3463(5) | 2872(4) | 5080(4) | 51.1(9) |
| C12 | 3613(5) | 560(4) | 1333(3) | 49.6(9) |
| C27 | 3765(5) | 2899(4) | 6258(4) | 59.4(11) |
| C22 | 1154(4) | 2524(4) | 2341(4) | 53.2(10) |
| C25 | 4874(5) | 1478(4) | 6227(4) | 56.5(10) |
| C16 | 2108(5) | -179(4) | 2509(4) | 52.7(10) |
| C13 | 3124(6) | -616(4) | 774(4) | 64.7(12) |
| C20 | 635(5) | 4070(4) | 1309(4) | 57.4(10) |
| C15 | 1609(5) | -1349(4) | 1931(4) | 65.8(13) |
| C8 | 7903(6) | -792(4) | 1752(4) | 61.7(11) |
| C26 | 4498(5) | 2230(4) | 6834(4) | 59.1(11) |
| C19 | 2042(5) | 4541(4) | 1309(4) | 63.3(12) |
| C14 | 2149(6) | -1554(4) | 1084(4) | 73.7(14) |
| C9 | 10794(5) | 4478(5) | 6859(5) | 85.2(18) |
| Cl2 | 6058.2(14) | 3158.1(10) | 292.7(9) | 64.2(4) |
| O5 | 6260(5) | 5811(5) | 1349(3) | 96.6(13) |

Table 3 Anisotropic Displacement Parameters (Å$^2$×10$^3$) for **2a**. The Anisotropic displacement factor exponent takes the form: $-2\pi^2[h^2a^{*2}U_{11}+2hka^*b^*U_{12}+\ldots]$.

| **Atom** | **U11** | **U22** | **U33** | **U12** | **U13** | **U23** |
|---|---|---|---|---|---|---|
| P1 | 43.1(5) | 33.1(4) | 36.9(5) | 16.7(4) | 17.9(4) | 9.7(3) |
| Cl1 | 66.0(7) | 46.1(5) | 62.3(6) | 29.1(5) | 17.0(5) | -3.8(4) |
| O4 | 51.2(15) | 46.5(14) | 45.7(14) | 14.5(12) | 16.0(12) | 3.5(11) |
| O3 | 66.8(18) | 60.9(17) | 48.6(15) | 39.9(15) | 9.2(14) | 3.9(13) |
| O2 | 61.0(17) | 51.8(15) | 43.7(14) | 29.0(14) | 14.5(13) | 3.0(12) |
| O1 | 78(2) | 64.5(18) | 53.8(16) | 46.9(17) | 17.6(15) | 7.8(14) |
| C3 | 43.0(19) | 35.3(16) | 42.8(18) | 11.7(15) | 17.9(16) | 7.5(14) |
| C6 | 38.8(18) | 38.3(16) | 40.0(17) | 16.0(14) | 17.1(15) | 8.9(14) |
| C7 | 43.3(19) | 42.3(18) | 42.8(18) | 22.7(16) | 16.2(16) | 13.3(15) |
| C1 | 42.1(19) | 36.4(17) | 44.4(18) | 15.7(15) | 19.8(16) | 10.6(14) |
| C4 | 45.3(19) | 30.0(15) | 48.5(19) | 13.1(14) | 23.2(16) | 6.5(14) |
| C23 | 51(2) | 34.4(16) | 44.4(18) | 15.8(15) | 26.2(16) | 9.6(14) |
| C10 | 43.0(19) | 37.3(16) | 39.0(17) | 17.7(15) | 16.4(15) | 11.4(14) |
| C11 | 49(2) | 36.7(17) | 37.1(17) | 17.7(15) | 12.2(15) | 6.8(14) |

| | | | | | | |
|---|---|---|---|---|---|---|
| C2 | 38.8(18) | 40.7(17) | 46.2(18) | 20.3(15) | 15.0(15) | 13.1(15) |
| C5 | 38.5(18) | 36.3(16) | 42.4(17) | 15.0(14) | 18.0(15) | 12.0(14) |
| C17 | 37.2(18) | 41.3(17) | 33.0(15) | 15.4(15) | 9.8(14) | 9.0(14) |
| C24 | 66(2) | 43.2(19) | 42.2(19) | 28.2(18) | 24.9(18) | 12.9(15) |
| C18 | 51(2) | 52(2) | 64(2) | 21.4(19) | 28(2) | 26.0(19) |
| C21 | 45(2) | 72(3) | 66(3) | 29(2) | 21(2) | 22(2) |
| C28 | 68(3) | 49(2) | 52(2) | 33(2) | 29(2) | 12.8(17) |
| C12 | 60(2) | 48(2) | 42.2(19) | 22.3(19) | 18.2(18) | 3.0(16) |
| C27 | 76(3) | 64(3) | 51(2) | 34(2) | 32(2) | 6(2) |
| C22 | 47(2) | 53(2) | 66(2) | 19.0(18) | 27(2) | 25.1(19) |
| C25 | 70(3) | 58(2) | 49(2) | 30(2) | 24(2) | 20.6(19) |
| C16 | 59(2) | 43.4(19) | 51(2) | 15.4(18) | 17.0(19) | 14.8(17) |
| C13 | 87(3) | 57(2) | 49(2) | 32(2) | 18(2) | -3.1(19) |
| C20 | 54(2) | 71(3) | 53(2) | 39(2) | 8.3(19) | 22(2) |
| C15 | 74(3) | 40(2) | 60(3) | 10(2) | 7(2) | 10.8(19) |
| C8 | 82(3) | 59(2) | 53(2) | 37(2) | 24(2) | 1(2) |
| C26 | 69(3) | 67(3) | 41(2) | 19(2) | 27(2) | 7.9(19) |
| C19 | 65(3) | 63(3) | 75(3) | 35(2) | 26(2) | 37(2) |
| C14 | 102(4) | 45(2) | 57(3) | 27(3) | 7(3) | -4(2) |
| C9 | 52(3) | 88(4) | 81(3) | -5(3) | 18(3) | -22(3) |
| Cl2 | 89.8(8) | 59.3(6) | 56.8(6) | 30.1(6) | 41.1(6) | 16.2(5) |
| O5 | 107(3) | 106(3) | 71(2) | 54(3) | 11(2) | -22(2) |

Table 4 Bond Lengths for **2a**.

| **Atom** | **Atom** | **Length/Å** | **Atom** | **Atom** | **Length/Å** |
|---|---|---|---|---|---|
| P1 | C23 | 1.798(3) | C23 | C28 | 1.388(5) |
| P1 | C10 | 1.810(3) | C10 | C5 | 1.506(5) |
| P1 | C11 | 1.793(4) | C11 | C12 | 1.391(5) |
| P1 | C17 | 1.793(4) | C11 | C16 | 1.388(5) |
| Cl1 | C4 | 1.726(3) | C17 | C18 | 1.382(5) |
| O4 | C3 | 1.362(4) | C17 | C22 | 1.394(5) |
| O4 | C9 | 1.419(5) | C24 | C25 | 1.366(5) |
| O3 | C2 | 1.340(4) | C18 | C19 | 1.382(6) |
| O2 | C7 | 1.321(4) | C21 | C22 | 1.378(6) |
| O2 | C8 | 1.455(5) | C21 | C20 | 1.380(6) |
| O1 | C7 | 1.222(4) | C28 | C27 | 1.377(6) |
| C3 | C4 | 1.383(5) | C12 | C13 | 1.384(6) |
| C3 | C2 | 1.404(5) | C27 | C26 | 1.373(6) |
| C6 | C1 | 1.381(5) | C25 | C26 | 1.388(6) |

| | | | | | |
|---|---|---|---|---|---|
| C6 | C5 | 1.387(5) | C16 | C15 | 1.385(6) |
| C7 | C1 | 1.472(5) | C13 | C14 | 1.370(7) |
| C1 | C2 | 1.409(5) | C20 | C19 | 1.380(6) |
| C4 | C5 | 1.407(5) | C15 | C14 | 1.382(7) |
| C23 | C24 | 1.395(5) | | | |

Table 5 Bond Angles for **2a**.

| Atom | Atom | Atom | Angle/° | Atom | Atom | Atom | Angle/° |
|---|---|---|---|---|---|---|---|
| C10 | P1 | C23 | 108.56(17) | C16 | C11 | P1 | 119.0(3) |
| C11 | P1 | C23 | 107.60(16) | C16 | C11 | C12 | 120.2(4) |
| C11 | P1 | C10 | 110.65(17) | C3 | C2 | O3 | 115.9(3) |
| C17 | P1 | C23 | 111.14(16) | C1 | C2 | O3 | 124.7(3) |
| C17 | P1 | C10 | 107.84(15) | C1 | C2 | C3 | 119.4(3) |
| C17 | P1 | C11 | 111.04(17) | C4 | C5 | C6 | 116.6(3) |
| C9 | O4 | C3 | 114.8(3) | C10 | C5 | C6 | 121.3(3) |
| C8 | O2 | C7 | 116.2(3) | C10 | C5 | C4 | 122.1(3) |
| C4 | C3 | O4 | 120.4(3) | C18 | C17 | P1 | 121.9(3) |
| C2 | C3 | O4 | 120.5(3) | C22 | C17 | P1 | 118.6(3) |
| C2 | C3 | C4 | 119.0(3) | C22 | C17 | C18 | 119.4(3) |
| C5 | C6 | C1 | 122.7(3) | C25 | C24 | C23 | 119.9(4) |
| O1 | C7 | O2 | 123.0(3) | C19 | C18 | C17 | 119.4(4) |
| C1 | C7 | O2 | 113.9(3) | C20 | C21 | C22 | 121.0(4) |
| C1 | C7 | O1 | 123.1(3) | C27 | C28 | C23 | 119.9(4) |
| C7 | C1 | C6 | 121.8(3) | C13 | C12 | C11 | 119.3(4) |
| C2 | C1 | C6 | 119.5(3) | C26 | C27 | C28 | 120.0(4) |
| C2 | C1 | C7 | 118.7(3) | C21 | C22 | C17 | 120.0(4) |
| C3 | C4 | Cl1 | 116.6(3) | C26 | C25 | C24 | 120.1(4) |
| C5 | C4 | Cl1 | 120.6(3) | C15 | C16 | C11 | 119.9(4) |
| C5 | C4 | C3 | 122.8(3) | C14 | C13 | C12 | 120.1(4) |
| C24 | C23 | P1 | 118.8(3) | C19 | C20 | C21 | 118.3(4) |
| C28 | C23 | P1 | 120.9(3) | C14 | C15 | C16 | 119.2(4) |
| C28 | C23 | C24 | 119.7(3) | C25 | C26 | C27 | 120.4(4) |
| C5 | C10 | P1 | 113.8(2) | C20 | C19 | C18 | 121.8(4) |

| | | | | | | | |
|---|---|---|---|---|---|---|---|
| C12 | C11 | P1 | 120.7(3) | C15 | C14 | C13 | 121.2(4) |

Table 6 Torsion Angles for **2a**.

| **A** | **B** | **C** | **D** | **Angle/°** | **A** | **B** | **C** | **D** | **Angle/°** |
|---|---|---|---|---|---|---|---|---|---|
| P1 | C23 | C24 | C25 | 169.8(3) | O2 | C7 | C1 | C2 | 177.5(3) |
| P1 | C23 | C28 | C27 | -170.0(3) | O1 | C7 | C1 | C6 | 175.5(4) |
| P1 | C10 | C5 | C6 | 88.3(3) | O1 | C7 | C1 | C2 | -3.6(4) |
| P1 | C10 | C5 | C4 | -93.4(3) | C3 | C4 | C5 | C6 | -0.7(4) |
| P1 | C11 | C12 | C13 | 178.9(4) | C3 | C4 | C5 | C10 | -179.1(3) |
| P1 | C11 | C16 | C15 | -179.8(3) | C3 | C2 | C1 | C6 | -1.1(4) |
| P1 | C17 | C18 | C19 | 178.8(4) | C3 | C2 | C1 | C7 | 178.0(3) |
| P1 | C17 | C22 | C21 | -178.8(3) | C23 | C24 | C25 | C26 | -0.3(5) |
| Cl1 | C4 | C3 | O4 | 2.8(3) | C23 | C28 | C27 | C26 | 1.1(5) |
| Cl1 | C4 | C3 | C2 | 178.7(2) | C11 | C12 | C13 | C14 | 1.4(5) |
| Cl1 | C4 | C5 | C6 | 178.6(3) | C11 | C16 | C15 | C14 | 0.6(5) |
| Cl1 | C4 | C5 | C10 | 0.2(3) | C17 | C18 | C19 | C20 | -0.1(5) |
| O4 | C3 | C4 | C5 | -177.9(3) | C17 | C22 | C21 | C20 | 0.0(5) |
| O4 | C3 | C2 | O3 | -1.1(4) | C24 | C25 | C26 | C27 | 2.8(5) |
| O4 | C3 | C2 | C1 | 178.8(3) | C18 | C19 | C20 | C21 | -0.1(6) |
| O3 | C2 | C3 | C4 | -177.0(3) | C28 | C27 | C26 | C25 | -3.2(5) |
| O3 | C2 | C1 | C6 | 178.8(4) | C12 | C13 | C14 | C15 | 1.7(6) |
| O3 | C2 | C1 | C7 | -2.1(4) | C16 | C15 | C14 | C13 | -2.7(5) |
| O2 | C7 | C1 | C6 | -3.4(4) | | | | | |

Table 7 Hydrogen Atom Coordinates (Å×104) and Isotropic Displacement Parameters (Å2×103) for **2a**.

| **Atom** | **x** | **y** | **z** | **U(eq)** |
|---|---|---|---|---|
| H3 | 10250(40) | 1470(50) | 5362(3) | 85.4(11) |
| H6 | 6571(4) | 1601(3) | 2451(3) | 45.4(9) |
| H10a | 5530(4) | 3145(3) | 2165(3) | 46.1(9) |
| H10b | 5780(4) | 4040(3) | 3240(3) | 46.1(9) |
| H24 | 4829(4) | 938(3) | 4649(3) | 56.2(11) |
| H18 | 3957(5) | 4359(4) | 1804(4) | 63.8(12) |

| | | | | |
|---|---|---|---|---|
| H21 | -740(5) | 2723(4) | 1836(4) | 70.3(13) |
| H28 | 2982(5) | 3335(4) | 4691(4) | 61.3(11) |
| H12 | 4259(5) | 1202(4) | 1114(3) | 59.6(11) |
| H27 | 3474(5) | 3370(4) | 6664(4) | 71.3(13) |
| H22 | 847(4) | 1840(4) | 2689(4) | 63.8(12) |
| H25 | 5333(5) | 1000(4) | 6617(4) | 67.8(12) |
| H16 | 1760(5) | -30(4) | 3086(4) | 63.2(12) |
| H13 | 3457(6) | -769(4) | 188(4) | 77.7(14) |
| H20 | -7(5) | 4427(4) | 966(4) | 68.9(12) |
| H15 | 918(5) | -1989(4) | 2111(4) | 78.9(15) |
| H8a | 7055(14) | -1280(20) | 1110(20) | 92.6(17) |
| H8b | 8700(30) | -426(4) | 1480(30) | 92.6(17) |
| H8c | 8140(40) | -1290(20) | 2315(9) | 92.6(17) |
| H26 | 4743(5) | 2281(4) | 7636(4) | 71.0(13) |
| H19 | 2344(5) | 5225(4) | 960(4) | 75.9(14) |
| H14 | 1846(6) | -2344(4) | 717(4) | 88.5(17) |
| H9a | 10728(6) | 5146(19) | 6460(30) | 128(3) |
| H9b | 11259(19) | 4760(30) | 7678(6) | 128(3) |
| H9c | 11351(17) | 4132(12) | 6580(40) | 128(3) |
| H5a | 5690(80) | 6090(60) | 900(50) | 144.9(19) |
| H5b | 6110(80) | 5150(40) | 960(50) | 144.9(19) |

**X-Ray Structure Determination data of 4a.**

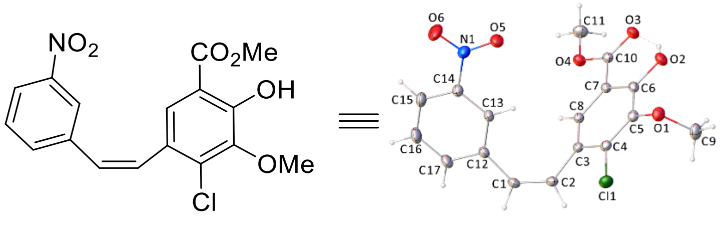


**Crystal Data** for $C_{17}H_{14}ClNO_6$ ($M$ =363.74 g/mol): triclinic, space group P-1 (no. 2), $a$ = 8.8195(4) Å, $b$ = 8.8888(4) Å, $c$ = 11.9711(6) Å, $\alpha$ = 94.2646(17)°, $\beta$ = 105.8369(16)°, $\gamma$ = 111.7489(15)°, $V$ = 822.25(7) Å$^3$, $Z$ = 2, $T$ = 300(2) K, μ(MoKα) = 0.267 mm$^{-1}$, $Dcalc$ = 1.469 g/cm$^3$, 38126 reflections measured (5.032° ≤ 2Θ ≤ 59.922°), 4767 unique ($R_{int}$ = 0.0524, $R_{sigma}$ = 0.0251) which were used in all calculations. The final $R_1$ was 0.0386 (I > 2σ(I)) and $wR_2$ was 0.1089 (all data).

X-ray intensity data from a colorless plate were collected at 100(2) K using a Bruker D8

QUEST diffractometer equipped with a PHOTON-II area detector and an Incoatec microfocus source (Mo Kα radiation, λ = 0.71073 Å). The raw area detector data frames were reduced, scaled and corrected for absorption effects using the Bruker APEX3, SAINT+ and SADABS programs.[8] The structure was solved with SHELXT.[9] Subsequent difference Fourier calculations and full-matrix least-squares refinement against $F^2$ were performed with SHELXL-2018 using OLEX2.[10]

The compound crystallizes in the triclinic system. The space group *P*-1 (No. 2) was confirmed by structure solution. The asymmetric unit consists of one molecule. All non-hydrogen atoms were refined with anisotropic displacement parameters. Hydrogen atoms bonded to carbon were located in Fourier difference maps before being placed in geometrically idealized positions and included as riding atoms with *d*(C-H) = 0.93 Å and *U*iso(H) = 1.2*U*eq(C) for arene hydrogen atoms and *d*(C-H) = 0.96 Å and *U*iso(H) = 1.5*U*eq(C) for methyl hydrogens. The methyl hydrogens were allowed to rotate as a rigid group to the orientation of maximum observed electron density. The hydroxyl hydrogen H2A was located and refined freely. The largest residual electron density peak in the final difference map is 0.23 $e^-/Å^3$, located 0.71 Å from C7.

Table 1 Crystal data and structure refinement for **4a**.

| | |
|---|---|
| Empirical formula | $C_{17}H_{14}ClNO_6$ |
| Formula weight | 363.74 |
| Temperature/K | 300(2) |
| Crystal system | triclinic |
| Space group | P-1 |
| a/Å | 8.8195(4) |
| b/Å | 8.8888(4) |
| c/Å | 11.9711(6) |
| α/° | 94.2646(17) |
| β/° | 105.8369(16) |
| γ/° | 111.7489(15) |
| Volume/Å$^3$ | 822.25(7) |
| Z | 2 |
| $\rho_{calc}$g/cm$^3$ | 1.469 |
| μ/mm$^{-1}$ | 0.267 |
| F(000) | 376.0 |
| Crystal size/mm$^3$ | 0.32 × 0.26 × 0.18 |

| | |
|---|---|
| Radiation | MoKα (λ = 0.71073) |
| 2Θ range for data collection/° | 5.032 to 59.922 |
| Index ranges | -12 ≤ h ≤ 12, -12 ≤ k ≤ 12, -16 ≤ l ≤ 16 |
| Reflections collected | 38126 |
| Independent reflections | 4767 [$R_{int}$ = 0.0524, $R_{sigma}$ = 0.0251] |
| Data/restraints/parameters | 4767/0/232 |
| Goodness-of-fit on $F^2$ | 1.064 |
| Final R indexes [I>=2σ (I)] | $R_1$ = 0.0386, $wR_2$ = 0.1009 |
| Final R indexes [all data] | $R_1$ = 0.0549, $wR_2$ = 0.1089 |
| Largest diff. peak/hole / e Å$^{-3}$ | 0.23/-0.18 |

Table 2 Fractional Atomic Coordinates (×10$^4$) and Equivalent Isotropic Displacement Parameters (Å$^2$×10$^3$) for **4a**. $U_{eq}$ is defined as 1/3 of the trace of the orthogonalised $U_{IJ}$ tensor.

| **Atom** | ***x*** | ***y*** | ***z*** | **U(eq)** |
|---|---|---|---|---|
| Cl1 | 2932.4(4) | 337.5(4) | 4462.2(3) | 52.50(12) |
| O1 | 5047.7(14) | 1500.1(12) | 2931.4(9) | 53.0(3) |
| O2 | 7798.3(15) | 4403.8(14) | 3407.1(9) | 51.0(2) |
| O3 | 9863.5(13) | 7170.7(13) | 4886.8(9) | 53.6(3) |
| O4 | 9658.5(12) | 7533.7(11) | 6700.1(8) | 45.9(2) |
| O5 | 10483.8(15) | 2588.2(17) | 7785.8(10) | 68.6(3) |
| O6 | 11620(2) | 2224(3) | 9501.8(13) | 101.9(5) |
| N1 | 10506.8(16) | 2492.4(17) | 8793.4(12) | 55.7(3) |
| C1 | 4794.8(17) | 2806.0(17) | 7891.6(12) | 44.5(3) |
| C2 | 4299.4(16) | 2825.1(16) | 6742.0(12) | 42.1(3) |
| C3 | 5318.8(15) | 3190.3(15) | 5926.9(11) | 36.8(2) |
| C4 | 4745.6(15) | 2168.4(14) | 4814.5(11) | 38.0(3) |
| C5 | 5587.4(17) | 2565.4(15) | 3986.0(11) | 40.1(3) |
| C6 | 7044.0(16) | 4052.4(15) | 4250.3(11) | 38.4(3) |
| C7 | 7651.0(15) | 5096.1(15) | 5358.3(10) | 36.1(2) |

| Atom | *x* | *y* | *z* | U(eq) |
|---|---|---|---|---|
| C8 | 6776.5(15) | 4641.1(15) | 6172.7(11) | 36.4(2) |
| C9 | 3912(2) | 1827(2) | 1975.0(13) | 65.5(4) |
| C10 | 9154.7(16) | 6679.1(16) | 5610.4(11) | 39.1(3) |
| C11 | 11118(2) | 9120.7(18) | 6978.3(15) | 57.2(4) |
| C12 | 6435.7(17) | 2924.8(15) | 8701.0(11) | 41.1(3) |
| C13 | 7722.3(17) | 2686.7(16) | 8343.2(11) | 40.3(3) |
| C14 | 9152.9(17) | 2716.6(16) | 9188.1(11) | 44.0(3) |
| C15 | 9396(2) | 2964(2) | 10386.4(13) | 57.0(4) |
| C16 | 8136(2) | 3216(2) | 10742.5(13) | 65.8(4) |
| C17 | 6692(2) | 3201(2) | 9917.5(13) | 56.5(4) |

Table 3 Anisotropic Displacement Parameters ($Å^2 \times 10^3$) for **4a**. The Anisotropic displacement factor exponent takes the form: $-2\pi^2[h^2a^{*2}U_{11}+2hka^*b^*U_{12}+\ldots]$.

| Atom | $U_{11}$ | $U_{22}$ | $U_{33}$ | $U_{23}$ | $U_{13}$ | $U_{12}$ |
|---|---|---|---|---|---|---|
| Cl1 | 42.45(19) | 45.23(17) | 59.8(2) | 6.61(15) | 12.01(15) | 11.02(14) |
| O1 | 61.6(6) | 55.3(5) | 41.7(5) | -1.2(4) | 12.4(5) | 28.4(5) |
| O2 | 58.7(6) | 59.0(6) | 43.4(5) | 11.2(5) | 28.8(5) | 23.7(5) |
| O3 | 47.0(6) | 63.2(6) | 48.5(6) | 18.7(5) | 23.3(5) | 13.3(5) |
| O4 | 43.0(5) | 44.1(5) | 45.6(5) | 7.5(4) | 17.6(4) | 10.2(4) |
| O5 | 63.5(7) | 106.6(9) | 53.9(7) | 19.3(6) | 28.0(6) | 47.1(7) |
| O6 | 88.5(10) | 183.8(17) | 73.5(9) | 41.9(10) | 23.2(8) | 97.8(12) |
| N1 | 49.3(7) | 74.5(8) | 50.5(7) | 13.4(6) | 14.4(6) | 33.9(6) |
| C1 | 39.2(7) | 53.6(7) | 47.6(7) | 13.7(6) | 24.4(6) | 18.3(6) |
| C2 | 32.2(6) | 50.0(7) | 47.8(7) | 14.2(6) | 16.9(5) | 17.2(5) |
| C3 | 33.1(6) | 44.9(6) | 37.2(6) | 14.2(5) | 12.2(5) | 19.4(5) |
| C4 | 33.8(6) | 39.8(6) | 42.3(6) | 10.4(5) | 10.1(5) | 18.2(5) |
| C5 | 43.4(7) | 44.8(6) | 35.7(6) | 6.7(5) | 11.1(5) | 23.5(5) |
| C6 | 41.2(6) | 47.8(6) | 36.4(6) | 13.3(5) | 17.4(5) | 24.6(5) |

| Atom | $U_{11}$ | $U_{22}$ | $U_{33}$ | $U_{23}$ | $U_{13}$ | $U_{12}$ |
|---|---|---|---|---|---|---|
| C7 | 34.7(6) | 43.5(6) | 35.8(6) | 12.6(5) | 13.9(5) | 19.3(5) |
| C8 | 35.4(6) | 43.5(6) | 32.6(6) | 9.1(5) | 11.6(5) | 17.8(5) |
| C9 | 74.3(11) | 72.4(10) | 37.9(8) | 6.2(7) | 8.0(7) | 25.1(9) |
| C10 | 36.0(6) | 46.9(6) | 39.3(6) | 14.4(5) | 14.6(5) | 19.4(5) |
| C11 | 46.2(8) | 46.6(7) | 66.3(10) | 5.1(7) | 18.2(7) | 6.6(6) |
| C12 | 44.3(7) | 46.0(6) | 36.7(6) | 11.2(5) | 19.2(5) | 17.3(5) |
| C13 | 43.9(7) | 49.1(6) | 30.6(6) | 9.0(5) | 14.4(5) | 20.0(5) |
| C14 | 45.5(7) | 50.8(7) | 37.9(6) | 9.3(5) | 13.9(6) | 21.5(6) |
| C15 | 62.6(9) | 73.5(10) | 34.5(7) | 10.9(7) | 8.8(6) | 32.2(8) |
| C16 | 83.1(12) | 90.3(12) | 32.5(7) | 14.3(7) | 21.7(8) | 42.0(10) |
| C17 | 66.0(10) | 75.8(10) | 41.9(7) | 15.6(7) | 30.4(7) | 34.2(8) |

Table 4 Bond Lengths for **4a**.

| Atom | Atom | Length/Å | Atom | Atom | Length/Å |
|---|---|---|---|---|---|
| Cl1 | C4 | 1.7277(13) | C3 | C4 | 1.4022(18) |
| O1 | C5 | 1.3701(16) | C3 | C8 | 1.3824(17) |
| O1 | C9 | 1.4277(18) | C4 | C5 | 1.3853(18) |
| O2 | C6 | 1.3485(15) | C5 | C6 | 1.3983(18) |
| O3 | C10 | 1.2172(15) | C6 | C7 | 1.4029(18) |
| O4 | C10 | 1.3275(16) | C7 | C8 | 1.3952(17) |
| O4 | C11 | 1.4521(16) | C7 | C10 | 1.4715(17) |
| O5 | N1 | 1.2107(16) | C12 | C13 | 1.3949(18) |
| O6 | N1 | 1.2203(17) | C12 | C17 | 1.3992(18) |
| N1 | C14 | 1.4694(18) | C13 | C14 | 1.3748(18) |
| C1 | C2 | 1.3289(18) | C14 | C15 | 1.3793(19) |
| C1 | C12 | 1.4665(19) | C15 | C16 | 1.376(2) |
| C2 | C3 | 1.4749(17) | C16 | C17 | 1.377(2) |

Table 5 Bond Angles for **4a**.

| Atom | Atom | Atom | Angle/° | Atom | Atom | Atom | Angle/° |
|---|---|---|---|---|---|---|---|
| C5 | O1 | C9 | 114.13(11) | C5 | C6 | C7 | 119.52(11) |
| C10 | O4 | C11 | 115.47(11) | C6 | C7 | C10 | 118.88(11) |
| O5 | N1 | O6 | 122.54(14) | C8 | C7 | C6 | 119.36(11) |
| O5 | N1 | C14 | 119.10(12) | C8 | C7 | C10 | 121.71(11) |
| O6 | N1 | C14 | 118.36(13) | C3 | C8 | C7 | 122.27(12) |
| C2 | C1 | C12 | 131.35(12) | O3 | C10 | O4 | 122.96(12) |
| C1 | C2 | C3 | 129.94(12) | O3 | C10 | C7 | 123.35(12) |
| C4 | C3 | C2 | 121.21(11) | O4 | C10 | C7 | 113.68(10) |
| C8 | C3 | C2 | 121.32(11) | C13 | C12 | C1 | 124.60(11) |
| C8 | C3 | C4 | 117.11(11) | C13 | C12 | C17 | 117.40(12) |
| C3 | C4 | Cl1 | 119.04(10) | C17 | C12 | C1 | 117.90(12) |
| C5 | C4 | Cl1 | 118.52(10) | C14 | C13 | C12 | 119.06(12) |
| C5 | C4 | C3 | 122.43(11) | C13 | C14 | N1 | 118.17(12) |
| O1 | C5 | C4 | 121.00(12) | C13 | C14 | C15 | 123.60(13) |
| O1 | C5 | C6 | 119.68(11) | C15 | C14 | N1 | 118.22(13) |
| C4 | C5 | C6 | 119.28(11) | C16 | C15 | C14 | 117.44(14) |
| O2 | C6 | C5 | 117.20(11) | C15 | C16 | C17 | 120.30(14) |
| O2 | C6 | C7 | 123.28(12) | C16 | C17 | C12 | 122.19(14) |

Table 6 Hydrogen Bonds for **4a**.

| D | H | A | d(D-H)/Å | d(H-A)/Å | d(D-A)/Å | D-H-A/° |
|---|---|---|---|---|---|---|
| O2 | H2A | O3 | 0.80(2) | 1.87(2) | 2.6030(15) | 153(2) |

Table 7 Torsion Angles for **4a**.

| A | B | C | D | Angle/° | A | B | C | D | Angle/° |
|---|---|---|---|---|---|---|---|---|---|
| Cl1 | C4 | C5 | O1 | -2.97(16) | C4 | C5 | C6 | C7 | 1.50(17) |
| Cl1 | C4 | C5 | C6 | 179.19(9) | C5 | C6 | C7 | C8 | -0.79(17) |
| O1 | C5 | C6 | O2 | 3.57(17) | C5 | C6 | C7 | C10 | -178.24(11) |
| O1 | C5 | C6 | C7 | -176.37(11) | C6 | C7 | C8 | C3 | -0.18(18) |

| A | B | C | D | Angle/˚ | A | B | C | D | Angle/˚ |
|---|---|---|---|---|---|---|---|---|---|
| O2 | C6 | C7 | C8 | 179.28(11) | C6 | C7 | C10 | O3 | 3.28(18) |
| O2 | C6 | C7 | C10 | 1.83(18) | C6 | C7 | C10 | O4 | -177.40(10) |
| O5 | N1 | C14 | C13 | 13.2(2) | C8 | C3 | C4 | Cl1 | 179.85(8) |
| O5 | N1 | C14 | C15 | -166.08(15) | C8 | C3 | C4 | C5 | 0.33(17) |
| O6 | N1 | C14 | C13 | -167.17(16) | C8 | C7 | C10 | O3 | -174.10(12) |
| O6 | N1 | C14 | C15 | 13.5(2) | C8 | C7 | C10 | O4 | 5.22(17) |
| N1 | C14 | C15 | C16 | 178.27(15) | C9 | O1 | C5 | C4 | 95.23(15) |
| C1 | C2 | C3 | C4 | 133.07(15) | C9 | O1 | C5 | C6 | -86.93(15) |
| C1 | C2 | C3 | C8 | -54.0(2) | C10 | C7 | C8 | C3 | 177.18(10) |
| C1 | C12 | C13 | C14 | -175.44(12) | C11 | O4 | C10 | O3 | 0.53(18) |
| C1 | C12 | C17 | C16 | 175.40(15) | C11 | O4 | C10 | C7 | -178.80(11) |
| C2 | C1 | C12 | C13 | -16.5(2) | C12 | C1 | C2 | C3 | -9.0(3) |
| C2 | C1 | C12 | C17 | 167.39(15) | C12 | C13 | C14 | N1 | -178.93(12) |
| C2 | C3 | C4 | Cl1 | -6.92(16) | C12 | C13 | C14 | C15 | 0.3(2) |
| C2 | C3 | C4 | C5 | 173.56(11) | C13 | C12 | C17 | C16 | -1.0(2) |
| C2 | C3 | C8 | C7 | -172.81(11) | C13 | C14 | C15 | C16 | -1.0(2) |
| C3 | C4 | C5 | O1 | 176.55(11) | C14 | C15 | C16 | C17 | 0.7(3) |
| C3 | C4 | C5 | C6 | -1.29(18) | C15 | C16 | C17 | C12 | 0.3(3) |
| C4 | C3 | C8 | C7 | 0.42(17) | C17 | C12 | C13 | C14 | 0.64(19) |
| C4 | C5 | C6 | O2 | -178.56(11) | | | | | |

Table 8 Hydrogen Atom Coordinates (Å×$10^4$) and Isotropic Displacement Parameters (Å$^2$×$10^3$) for **4a**.

| Atom | *x* | *y* | *z* | U(eq) |
|---|---|---|---|---|
| H2A | 8520(30) | 5320(20) | 3672(18) | 70(6) |
| H1 | 3952.52 | 2698.25 | 8245.04 | 53 |
| H2 | 3130.18 | 2568.68 | 6398.58 | 50 |
| H8 | 7188.59 | 5337.88 | 6906.23 | 44 |
| H9A | 3688.75 | 1115.01 | 1252.07 | 98 |

| Atom | *x* | *y* | *z* | U(eq) |
|---|---|---|---|---|
| H9B | 4437.2 | 2958.42 | 1904.72 | 98 |
| H9C | 2845.31 | 1627.64 | 2124.99 | 98 |
| H11A | 10821.21 | 9820.85 | 6466.25 | 86 |
| H11B | 12088.77 | 8961.18 | 6865 | 86 |
| H11C | 11409.75 | 9629.17 | 7787.47 | 86 |
| H13 | 7613.93 | 2510.13 | 7544.34 | 48 |
| H15 | 10372.37 | 2960.06 | 10932.66 | 68 |
| H16 | 8261.46 | 3398.65 | 11543.98 | 79 |
| H17 | 5859.57 | 3379 | 10177.39 | 68 |

**X-Ray Structure Determination data of 4b.**

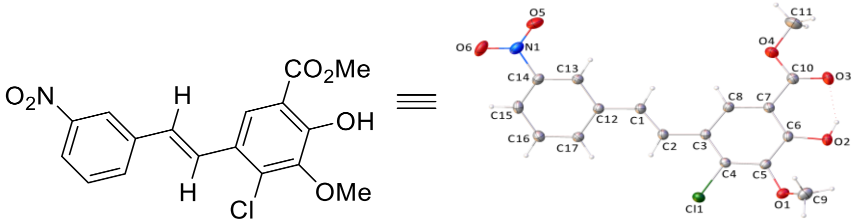


**Crystal Data** for $C_{18.2}H_{16.8}ClNO_6$ ($M$ =380.98 g/mol): monoclinic, space group $P2_1/n$ (no. 14), $a$ = 16.1708(4) Å, $b$ = 3.90140(10) Å, $c$ = 28.7704(7) Å, $\beta$ = 100.7690(10)°, $V$ = 1783.12(8) Å$^3$, $Z$ = 4, $T$ = 300(2) K, μ(MoKα) = 0.250 mm$^{-1}$, $Dcalc$ = 1.419 g/cm$^3$, 35090 reflections measured (5.394° ≤ 2Θ ≤ 52.812°), 3634 unique ($R_{int}$ = 0.0561, $R_{sigma}$ = 0.0258) which were used in all calculations. The final $R_1$ was 0.0426 (I > 2σ(I)) and $wR_2$ was 0.1270 (all data).

Table 1 Crystal data and structure refinement for **4b**.

| | |
|---|---|
| Empirical formula | $C_{18.2}H_{16.8}ClNO_6$ |
| Formula weight | 380.98 |
| Temperature/K | 300(2) |
| Crystal system | monoclinic |
| Space group | $P2_1/n$ |
| a/Å | 16.1708(4) |
| b/Å | 3.90140(10) |
| c/Å | 28.7704(7) |

| | |
|---|---|
| α/° | 90 |
| β/° | 100.7690(10) |
| γ/° | 90 |
| Volume/Å$^3$ | 1783.12(8) |
| Z | 4 |
| $\rho_{calc}$g/cm$^3$ | 1.419 |
| μ/mm$^{-1}$ | 0.250 |
| F(000) | 792.0 |
| Crystal size/mm$^3$ | 0.46 × 0.14 × 0.08 |
| Radiation | MoKα (λ = 0.71073) |
| 2Θ range for data collection/° | 5.394 to 52.812 |
| Index ranges | -20 ≤ h ≤ 20, -4 ≤ k ≤ 4, -35 ≤ l ≤ 35 |
| Reflections collected | 35090 |
| Independent reflections | 3634 [$R_{int}$ = 0.0561, $R_{sigma}$ = 0.0258] |
| Data/restraints/parameters | 3634/9/251 |
| Goodness-of-fit on F$^2$ | 1.043 |
| Final R indexes [I>=2σ (I)] | $R_1$ = 0.0426, w$R_2$ = 0.1141 |
| Final R indexes [all data] | $R_1$ = 0.0573, w$R_2$ = 0.1270 |
| Largest diff. peak/hole / e Å$^{-3}$ | 0.19/-0.23 |

Table 2 Fractional Atomic Coordinates (×10$^4$) and Equivalent Isotropic Displacement Parameters (Å$^2$×10$^3$) for **4b**. $U_{eq}$ is defined as 1/3 of the trace of the orthogonalised $U_{IJ}$ tensor.

| **Atom** | ***x*** | ***y*** | ***z*** | **U(eq)** |
|---|---|---|---|---|
| Cl1 | 6008.8(3) | 2077.1(15) | 5301.0(2) | 53.43(18) |
| O1 | 7542.0(9) | -1119(4) | 5769.2(5) | 54.9(4) |
| O2 | 8871.9(10) | -2981(4) | 5372.7(6) | 58.0(4) |
| O3 | 9471.9(10) | -3595(6) | 4586.9(6) | 80.7(6) |
| O4 | 8734.4(10) | -1549(5) | 3917.7(6) | 71.1(5) |
| O5 | 4954.1(15) | 8770(8) | 2196.7(7) | 116.0(9) |

| **Atom** | ***x*** | ***y*** | ***z*** | **U(eq)** |
|---|---|---|---|---|
| O6 | 3640.3(13) | 9677(6) | 2097.6(6) | 90.1(6) |
| N1 | 4289.8(14) | 8544(6) | 2326.0(6) | 66.3(5) |
| C1 | 5819.7(12) | 3425(6) | 3798.7(7) | 49.9(5) |
| C2 | 5931.1(12) | 2333(5) | 4236.9(7) | 45.6(5) |
| C3 | 6722.7(11) | 1157(5) | 4527.9(6) | 40.8(4) |
| C4 | 6814.5(11) | 788(5) | 5019.3(6) | 39.5(4) |
| C5 | 7528.6(11) | -564(5) | 5299.6(6) | 42.0(4) |
| C6 | 8204.4(11) | -1571(5) | 5086.2(7) | 44.7(4) |
| C7 | 8141.9(11) | -1116(5) | 4598.7(7) | 44.0(4) |
| C8 | 7408.1(11) | 200(5) | 4330.1(7) | 44.9(4) |
| C9 | 8168.9(16) | 686(7) | 6096.1(8) | 66.4(6) |
| C10 | 8847.6(13) | -2208(6) | 4376.3(8) | 53.9(5) |
| C11 | 9404(2) | -2603(10) | 3677.7(12) | 99.5(11) |
| C12 | 5021.2(11) | 4407(5) | 3498.1(6) | 42.6(4) |
| C13 | 5022.6(12) | 5950(6) | 3063.0(7) | 48.0(5) |
| C14 | 4268.9(13) | 6872(5) | 2779.6(6) | 47.4(5) |
| C15 | 3507.3(13) | 6320(6) | 2908.0(8) | 53.7(5) |
| C16 | 3503.8(13) | 4759(6) | 3336.3(8) | 55.5(5) |
| C17 | 4244.0(12) | 3818(5) | 3626.5(7) | 46.4(5) |
| C1S | 7624(15) | -1550(100) | 2329(8) | 101(3) |
| C2S | 7376(15) | 1680(100) | 2577(9) | 101(3) |
| C3S | 7663(15) | 4930(100) | 2341(8) | 101(3) |
| C4S | 7340(15) | 8180(100) | 2569(9) | 101(3) |
| C5S | 7647(15) | 11450(90) | 2359(8) | 101(3) |
| C6S | 7351(16) | 14820(100) | 2545(10) | 101(3) |

Table 3 Anisotropic Displacement Parameters ($Å^2 \times 10^3$) for **4b**. The Anisotropic displacement factor exponent takes the form: $-2\pi^2[h^2a^{*2}U_{11}+2hka^*b^*U_{12}+\ldots]$.

| Atom | $U_{11}$ | $U_{22}$ | $U_{33}$ | $U_{23}$ | $U_{13}$ | $U_{12}$ |
|---|---|---|---|---|---|---|
| Cl1 | 45.0(3) | 69.6(4) | 47.1(3) | 0.5(2) | 12.3(2) | 6.2(2) |
| O1 | 50.3(8) | 73.2(10) | 38.7(7) | 9.4(7) | 1.3(6) | -5.2(7) |
| O2 | 42.4(8) | 76.8(11) | 52.0(9) | 13.2(8) | 1.6(7) | 16.3(7) |
| O3 | 52.2(10) | 116.5(16) | 74.3(11) | 18.4(11) | 14.0(8) | 36.7(10) |
| O4 | 60.0(10) | 103.8(14) | 53.8(9) | 4.7(9) | 21.7(7) | 24.3(9) |
| O5 | 96.5(16) | 183(3) | 74.3(13) | 60.3(15) | 29.3(12) | 15.3(16) |
| O6 | 97.8(14) | 107.6(15) | 52.8(10) | 21.7(10) | -16.9(9) | 19.8(12) |
| N1 | 77.4(14) | 76.6(14) | 41.4(10) | 8.1(9) | 2.3(10) | 5.9(11) |
| C1 | 38.0(10) | 64.6(13) | 45.6(11) | 7.0(9) | 3.9(8) | 4.0(9) |
| C2 | 38.0(10) | 55.4(12) | 41.5(10) | -0.9(9) | 2.8(8) | 6.4(8) |
| C3 | 37.6(9) | 42.7(10) | 40.6(9) | 0.4(8) | 3.1(7) | 2.1(8) |
| C4 | 35.3(9) | 40.3(10) | 42.4(10) | -1.2(8) | 5.8(7) | -1.6(7) |
| C5 | 42.0(10) | 45.4(10) | 36.7(9) | 4.3(8) | 2.9(7) | -1.9(8) |
| C6 | 35.7(9) | 48.0(11) | 47.5(10) | 6.2(9) | -0.1(8) | 2.7(8) |
| C7 | 38.2(10) | 46.8(11) | 46.5(10) | 2.2(8) | 6.8(8) | 3.1(8) |
| C8 | 45.3(10) | 50.1(11) | 38.0(9) | 1.5(8) | 4.3(8) | 4.5(9) |
| C9 | 77.3(16) | 72.9(16) | 43.9(12) | -4.6(11) | -1.9(11) | -7.0(13) |
| C10 | 45.5(11) | 60.9(13) | 55.7(12) | 2.7(10) | 10.2(9) | 7.8(10) |
| C11 | 85(2) | 137(3) | 91(2) | 6.7(19) | 51.4(17) | 36.5(19) |
| C12 | 42.2(10) | 45.3(10) | 38.6(9) | -1.2(8) | 3.1(7) | 4.0(8) |
| C13 | 41.9(10) | 60.9(12) | 41.1(10) | 3.9(9) | 7.5(8) | 3.5(9) |
| C14 | 55.6(12) | 49.3(11) | 34.1(9) | -0.7(8) | 0.2(8) | 3.9(9) |
| C15 | 41.6(11) | 57.8(13) | 55.8(12) | -1.4(10) | -6.4(9) | 7.6(9) |
| C16 | 40.1(10) | 64.2(14) | 61.4(13) | 4.7(11) | 7.8(9) | 3.1(10) |
| C17 | 44.9(10) | 52.9(12) | 41.1(10) | 3.6(9) | 7.5(8) | 2.3(9) |

Table 4 Bond Lengths for **4b**.

| Atom | Atom | Length/Å | Atom | Atom | Length/Å |
|---|---|---|---|---|---|
| Cl1 | C4 | 1.7325(18) | C5 | C6 | 1.406(3) |
| O1 | C5 | 1.364(2) | C6 | C7 | 1.398(3) |
| O1 | C9 | 1.432(3) | C7 | C8 | 1.388(3) |
| O2 | C6 | 1.347(2) | C7 | C10 | 1.472(3) |
| O3 | C10 | 1.205(3) | C12 | C13 | 1.389(3) |
| O4 | C10 | 1.323(3) | C12 | C17 | 1.394(3) |
| O4 | C11 | 1.449(3) | C13 | C14 | 1.381(3) |
| O5 | N1 | 1.204(3) | C14 | C15 | 1.367(3) |
| O6 | N1 | 1.213(3) | C15 | C16 | 1.375(3) |
| N1 | C14 | 1.465(3) | C16 | C17 | 1.375(3) |
| C1 | C2 | 1.311(3) | C1S | C2S | 1.537(15) |
| C1 | C12 | 1.464(3) | C2S | C3S | 1.549(15) |
| C2 | C3 | 1.466(2) | C3S | C4S | 1.563(15) |
| C3 | C4 | 1.401(3) | C4S | C5S | 1.532(15) |
| C3 | C8 | 1.388(3) | C5S | C6S | 1.529(16) |
| C4 | C5 | 1.383(2) | | | |

Table 5 Bond Angles for **4b**.

| Atom | Atom | Atom | Angle/° | Atom | Atom | Atom | Angle/° |
|---|---|---|---|---|---|---|---|
| C5 | O1 | C9 | 116.78(16) | C8 | C7 | C6 | 119.96(17) |
| C10 | O4 | C11 | 116.3(2) | C8 | C7 | C10 | 121.10(18) |
| O5 | N1 | O6 | 122.5(2) | C7 | C8 | C3 | 122.42(17) |
| O5 | N1 | C14 | 118.6(2) | O3 | C10 | O4 | 122.4(2) |
| O6 | N1 | C14 | 118.9(2) | O3 | C10 | C7 | 123.8(2) |
| C2 | C1 | C12 | 126.91(19) | O4 | C10 | C7 | 113.79(17) |
| C1 | C2 | C3 | 126.82(18) | C13 | C12 | C1 | 119.71(17) |
| C4 | C3 | C2 | 121.67(17) | C13 | C12 | C17 | 117.62(17) |
| C8 | C3 | C2 | 121.92(17) | C17 | C12 | C1 | 122.66(17) |
| C8 | C3 | C4 | 116.35(16) | C14 | C13 | C12 | 119.65(18) |

| Atom | Atom | Atom | Angle/° | Atom | Atom | Atom | Angle/° |
|---|---|---|---|---|---|---|---|
| C3 | C4 | Cl1 | 119.67(13) | C13 | C14 | N1 | 118.48(19) |
| C5 | C4 | Cl1 | 117.17(14) | C15 | C14 | N1 | 118.86(18) |
| C5 | C4 | C3 | 123.16(16) | C15 | C14 | C13 | 122.66(18) |
| O1 | C5 | C4 | 119.66(16) | C14 | C15 | C16 | 117.80(18) |
| O1 | C5 | C6 | 121.22(16) | C17 | C16 | C15 | 120.89(19) |
| C4 | C5 | C6 | 118.96(16) | C16 | C17 | C12 | 121.38(18) |
| O2 | C6 | C5 | 116.50(17) | C1S | C2S | C3S | 110.0(19) |
| O2 | C6 | C7 | 124.40(18) | C2S | C3S | C4S | 109.3(15) |
| C7 | C6 | C5 | 119.09(16) | C5S | C4S | C3S | 111(2) |
| C6 | C7 | C10 | 118.87(17) | C6S | C5S | C4S | 115.5(16) |

Table 6 Hydrogen Bonds for **4b**.

| D | H | A | d(D-H)/Å | d(H-A)/Å | d(D-A)/Å | D-H-A/° |
|---|---|---|---|---|---|---|
| O2 | H2A | O3 | 0.80(3) | 1.92(3) | 2.632(2) | 147(3) |

Table 7 Torsion Angles for **4b**.

| A | B | C | D | Angle/° | A | B | C | D | Angle/° |
|---|---|---|---|---|---|---|---|---|---|
| Cl1 | C4 | C5 | O1 | 6.0(3) | C5 | C6 | C7 | C8 | -2.3(3) |
| Cl1 | C4 | C5 | C6 | -178.60(15) | C5 | C6 | C7 | C10 | -179.36(18) |
| O1 | C5 | C6 | O2 | -2.4(3) | C6 | C7 | C8 | C3 | 1.0(3) |
| O1 | C5 | C6 | C7 | 176.56(18) | C6 | C7 | C10 | O3 | 2.7(4) |
| O2 | C6 | C7 | C8 | 176.60(19) | C6 | C7 | C10 | O4 | -177.7(2) |
| O2 | C6 | C7 | C10 | -0.5(3) | C8 | C3 | C4 | Cl1 | 177.41(14) |
| O5 | N1 | C14 | C13 | 6.7(3) | C8 | C3 | C4 | C5 | -2.3(3) |
| O5 | N1 | C14 | C15 | -174.0(3) | C8 | C7 | C10 | O3 | -174.3(2) |
| O6 | N1 | C14 | C13 | -172.5(2) | C8 | C7 | C10 | O4 | 5.2(3) |
| O6 | N1 | C14 | C15 | 6.8(3) | C9 | O1 | C5 | C4 | -119.6(2) |
| N1 | C14 | C15 | C16 | -179.7(2) | C9 | O1 | C5 | C6 | 65.1(3) |
| C1 | C2 | C3 | C4 | 165.2(2) | C10 | C7 | C8 | C3 | 178.06(19) |

| A | B | C | D | Angle/° | A | B | C | D | Angle/° |
|---|---|---|---|---|---|---|---|---|---|
| C1 | C2 | C3 | C8 | -17.8(3) | C11 | O4 | C10 | O3 | 0.3(4) |
| C1 | C12 | C13 | C14 | 179.95(19) | C11 | O4 | C10 | C7 | -179.3(2) |
| C1 | C12 | C17 | C16 | -179.8(2) | C12 | C1 | C2 | C3 | 176.1(2) |
| C2 | C1 | C12 | C13 | 171.4(2) | C12 | C13 | C14 | N1 | 179.03(19) |
| C2 | C1 | C12 | C17 | -9.6(3) | C12 | C13 | C14 | C15 | -0.3(3) |
| C2 | C3 | C4 | Cl1 | -5.5(3) | C13 | C12 | C17 | C16 | -0.7(3) |
| C2 | C3 | C4 | C5 | 174.83(18) | C13 | C14 | C15 | C16 | -0.5(3) |
| C2 | C3 | C8 | C7 | -175.93(19) | C14 | C15 | C16 | C17 | 0.6(3) |
| C3 | C4 | C5 | O1 | -174.30(18) | C15 | C16 | C17 | C12 | 0.0(3) |
| C3 | C4 | C5 | C6 | 1.1(3) | C17 | C12 | C13 | C14 | 0.8(3) |
| C4 | C3 | C8 | C7 | 1.2(3) | C1S | C2S | C3S | C4S | 175.3(16) |
| C4 | C5 | C6 | O2 | -177.74(18) | C2S | C3S | C4S | C5S | 177.4(16) |
| C4 | C5 | C6 | C7 | 1.2(3) | C3S | C4S | C5S | C6S | 178.4(15) |

Table 8 Hydrogen Atom Coordinates (Å×$10^4$) and Isotropic Displacement Parameters (Å$^2$×$10^3$) for **4b**.

| Atom | *x* | *y* | *z* | U(eq) |
|---|---|---|---|---|
| H2A | 9217(19) | -3390(80) | 5212(10) | 85(10) |
| H1 | 6298.57 | 3594.75 | 3664.43 | 60 |
| H2 | 5458.5 | 2299.84 | 4377.95 | 55 |
| H8 | 7374.23 | 450.03 | 4005.62 | 54 |
| H9A | 8302.17 | 2799.29 | 5955.88 | 100 |
| H9B | 8666.92 | -697.53 | 6171.65 | 100 |
| H9C | 7957.8 | 1164.57 | 6379.73 | 100 |
| H11A | 9306.26 | -1663.61 | 3363.81 | 149 |
| H11B | 9417.08 | -5058.88 | 3660.37 | 149 |
| H11C | 9933.5 | -1783.65 | 3850.03 | 149 |
| H13 | 5528.33 | 6358.9 | 2962.98 | 58 |
| H15 | 3008.09 | 6977.65 | 2712.16 | 64 |

| Atom | *x* | *y* | *z* | U(eq) |
|---|---|---|---|---|
| H16 | 2994.04 | 4333.71 | 3430.86 | 67 |
| H17 | 4225.53 | 2766.52 | 3914.48 | 56 |
| H1SA | 7356.26 | -1510.29 | 2001.73 | 152 |
| H1SB | 7447.26 | -3547.93 | 2478.23 | 152 |
| H1SC | 8224 | -1599.5 | 2352.07 | 152 |
| H2SA | 7637.74 | 1630.86 | 2908.64 | 121 |
| H2SB | 6770.35 | 1733.33 | 2555.55 | 121 |
| H3SA | 7437.66 | 4895.54 | 2003.8 | 121 |
| H3SB | 8271.92 | 4982.41 | 2385.09 | 121 |
| H4SA | 6729.19 | 8168.67 | 2512.6 | 121 |
| H4SB | 7542.63 | 8149.19 | 2908.15 | 121 |
| H5SA | 8257.57 | 11434.9 | 2421.4 | 121 |
| H5SB | 7460.51 | 11392.79 | 2018.62 | 121 |
| H6SA | 6747.03 | 14899.52 | 2479.84 | 152 |
| H6SB | 7569.9 | 16709.35 | 2392.38 | 152 |
| H6SC | 7550.17 | 14959.5 | 2880.69 | 152 |

Table 9 Atomic Occupancy for **4b**.

| Atom | *Occupancy* | Atom | *Occupancy* | Atom | *Occupancy* |
|---|---|---|---|---|---|
| C1S | 0.2 | H1SA | 0.2 | H1SB | 0.2 |
| H1SC | 0.2 | C2S | 0.2 | H2SA | 0.2 |
| H2SB | 0.2 | C3S | 0.2 | H3SA | 0.2 |
| H3SB | 0.2 | C4S | 0.2 | H4SA | 0.2 |
| H4SB | 0.2 | C5S | 0.2 | H5SA | 0.2 |
| H5SB | 0.2 | C6S | 0.2 | H6SA | 0.2 |
| H6SB | 0.2 | H6SC | 0.2 | | |

**X-Ray Structure Determination data of 5b.**

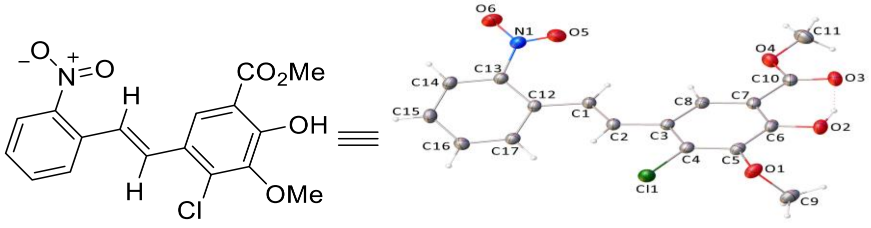


**Crystal Data** for $C_{17.9325}H_{15.865}ClNO_{6.465}$ ($M$ =384.26 g/mol): monoclinic, space group $P2_1/c$ (no. 14), $a$ = 3.8774(3) Å, $b$ = 17.4232(12) Å, $c$ = 25.4941(17) Å, $\beta$ = 91.795(2)°, $V$ = 1721.5(2) Å$^3$, $Z$ = 4, $T$ = 300(2) K, μ(MoKα) = 0.261 mm$^{-1}$, $Dcalc$ = 1.483 g/cm$^3$, 97799 reflections measured (4.676° ≤ 2Θ ≤ 52.822°), 3516 unique ($R_{int}$ = 0.0734, $R_{sigma}$ = 0.0184) which were used in all calculations. The final $R_1$ was 0.0429 (I > 2σ(I)) and $wR_2$ was 0.1201 (all data).

Table 1 Crystal data and structure refinement for **5b**.

| | |
|---|---|
| Empirical formula | $C_{17.93}H_{15.87}ClNO_{6.46}$ |
| Formula weight | 384.26 |
| Temperature/K | 300(2) |
| Crystal system | monoclinic |
| Space group | $P2_1/c$ |
| a/Å | 3.8774(3) |
| b/Å | 17.4232(12) |
| c/Å | 25.4941(17) |
| α/° | 90 |
| β/° | 91.795(2) |
| γ/° | 90 |
| Volume/Å$^3$ | 1721.5(2) |
| Z | 4 |
| $\rho_{calc}$g/cm$^3$ | 1.483 |
| μ/mm$^{-1}$ | 0.261 |
| F(000) | 797.0 |
| Crystal size/mm$^3$ | 0.54 × 0.46 × 0.2 |
| Radiation | MoKα (λ = 0.71073) |

| | |
|---|---|
| 2Θ range for data collection/° | 4.676 to 52.822 |
| Index ranges | -4 ≤ h ≤ 4, -21 ≤ k ≤ 21, -31 ≤ l ≤ 31 |
| Reflections collected | 97799 |
| Independent reflections | 3516 [$R_{int}$ = 0.0734, $R_{sigma}$ = 0.0184] |
| Data/restraints/parameters | 3516/75/282 |
| Goodness-of-fit on $F^2$ | 1.036 |
| Final R indexes [I>=2σ (I)] | $R_1$ = 0.0429, $wR_2$ = 0.1109 |
| Final R indexes [all data] | $R_1$ = 0.0564, $wR_2$ = 0.1201 |
| Largest diff. peak/hole / e Å$^{-3}$ | 0.32/-0.33 |

Table 2 Fractional Atomic Coordinates (×10$^4$) and Equivalent Isotropic Displacement Parameters (Å$^2$×10$^3$) for **5b**. $U_{eq}$ is defined as 1/3 of of the trace of the orthogonalised $U_{IJ}$ tensor.

| **Atom** | ***x*** | ***y*** | ***z*** | **U(eq)** |
|---|---|---|---|---|
| Cl1 | 3464.2(14) | 531.4(3) | 5655.0(2) | 61.94(19) |
| O1 | 2015(4) | 1036.8(9) | 6694.4(5) | 67.1(4) |
| O2 | 3406(4) | 2443.4(11) | 7115.9(5) | 69.9(4) |
| O3 | 5996(5) | 3797.2(10) | 6988.6(6) | 75.9(5) |
| O4 | 8390(4) | 4084.8(9) | 6231.7(6) | 70.7(4) |
| O5 | 5375(6) | 3397.7(10) | 4048.4(7) | 91.7(6) |
| O6 | 7597(6) | 3607.4(10) | 3312.0(7) | 96.5(6) |
| N1 | 7101(5) | 3193.0(10) | 3684.9(7) | 61.9(5) |
| C1 | 8128(5) | 2253.1(11) | 4674.4(7) | 48.9(4) |
| C2 | 6883(5) | 1791.3(11) | 5032.1(7) | 48.1(4) |
| C3 | 6057(4) | 1978.5(10) | 5573.3(6) | 45.1(4) |
| C4 | 4486(5) | 1432.0(11) | 5896.7(7) | 47.3(4) |
| C5 | 3674(5) | 1583.4(12) | 6411.5(7) | 51.3(4) |
| C6 | 4371(5) | 2309.5(12) | 6619.5(7) | 52.7(5) |
| C7 | 5984(5) | 2860.8(11) | 6313.1(7) | 49.9(4) |
| C8 | 6784(5) | 2686.4(11) | 5797.4(7) | 48.6(4) |

| Atom | *x* | *y* | *z* | U(eq) |
|---|---|---|---|---|
| C9 | 3776(6) | 766.5(16) | 7159.5(9) | 75.8(7) |
| C10 | 6764(5) | 3614.1(12) | 6545.3(7) | 57.6(5) |
| C11 | 9247(7) | 4839.2(13) | 6436.2(10) | 79.1(7) |
| C12 | 8915(4) | 1983.2(10) | 4144.0(6) | 44.7(4) |
| C13 | 8531(5) | 2415.0(11) | 3681.2(7) | 48.6(4) |
| C14 | 9348(5) | 2130.8(13) | 3194.7(7) | 57.5(5) |
| C15 | 10559(6) | 1393.8(13) | 3152.8(7) | 61.2(5) |
| C16 | 10991(5) | 953.6(12) | 3596.0(7) | 57.5(5) |
| C17 | 10224(5) | 1248.7(11) | 4080.7(7) | 50.4(4) |
| O1S | 10620(40) | 4057(10) | 5018(8) | 241(9) |
| O2S | 7570(40) | 4949(11) | 5002(12) | 135(5) |
| C1S | 13680(40) | 5204(13) | 5005(9) | 108(6) |
| C2S | 10700(40) | 4694(9) | 5001(6) | 105(5) |
| C3S | 4130(40) | 4651(11) | 5036(11) | 149(8) |
| C4S | 1730(50) | 5317(14) | 5008(17) | 135(5) |

Table 3 Anisotropic Displacement Parameters (Å$^2$×10$^3$) for **5b**. The Anisotropic displacement factor exponent takes the form: $-2\pi^2[h^2a^{*2}U_{11}+2hka^*b^*U_{12}+\ldots]$.

| Atom | $U_{11}$ | $U_{22}$ | $U_{33}$ | $U_{23}$ | $U_{13}$ | $U_{12}$ |
|---|---|---|---|---|---|---|
| Cl1 | 78.6(4) | 53.3(3) | 54.2(3) | 1.2(2) | 6.5(2) | -11.2(2) |
| O1 | 71.4(9) | 85.4(11) | 44.9(7) | 15.4(7) | 6.0(6) | -16.3(8) |
| O2 | 89.6(11) | 82.6(12) | 38.5(7) | -5.3(7) | 17.6(7) | 0.8(9) |
| O3 | 106.9(12) | 74.7(10) | 46.4(8) | -17.4(7) | 8.5(8) | 6.4(9) |
| O4 | 98.3(11) | 56.6(9) | 57.6(9) | -13.5(7) | 9.6(8) | -10.8(8) |
| O5 | 136.4(16) | 72.6(11) | 67.1(11) | 1.0(9) | 16.9(11) | 31.7(11) |
| O6 | 162.4(19) | 62.6(10) | 64.9(10) | 20.4(9) | 7.7(11) | 3.0(11) |
| N1 | 86.0(12) | 53.3(10) | 46.3(9) | 2.4(8) | -3.3(9) | -3.6(9) |
| C1 | 63.7(11) | 46.8(10) | 36.2(9) | -2.2(7) | 3.6(8) | -4.1(8) |

| Atom | $U_{11}$ | $U_{22}$ | $U_{33}$ | $U_{23}$ | $U_{13}$ | $U_{12}$ |
|---|---|---|---|---|---|---|
| C2 | 56.2(10) | 50.9(10) | 37.1(9) | -3.8(8) | 2.7(7) | -2.1(8) |
| C3 | 50.3(9) | 50.4(10) | 34.8(8) | 1.4(7) | 2.4(7) | 0.9(8) |
| C4 | 51.2(10) | 51.5(10) | 39.2(9) | 1.5(8) | 1.5(7) | 0.5(8) |
| C5 | 52.7(10) | 62.7(12) | 38.8(9) | 7.8(8) | 5.1(8) | -0.9(9) |
| C6 | 56.7(10) | 68.8(12) | 32.7(9) | -0.8(8) | 5.4(7) | 6.6(9) |
| C7 | 56.4(10) | 56.0(11) | 37.4(9) | -3.7(8) | 1.4(7) | 5.1(8) |
| C8 | 57.6(10) | 52.4(10) | 36.0(9) | 0.6(8) | 3.8(7) | 0.7(8) |
| C9 | 84.4(15) | 88.8(17) | 54.7(13) | 23.4(12) | 7.9(11) | -0.4(13) |
| C10 | 69.1(12) | 60.2(12) | 43.1(10) | -5.9(9) | -1.0(9) | 8.3(10) |
| C11 | 99.0(18) | 56.4(13) | 82.1(16) | -16.6(12) | 3.5(13) | -7.1(12) |
| C12 | 50.4(9) | 49.1(10) | 34.8(8) | -1.6(7) | 3.9(7) | -7.4(8) |
| C13 | 58.8(10) | 48.5(10) | 38.6(9) | 1.0(7) | 2.9(8) | -6.5(8) |
| C14 | 71.8(12) | 65.5(13) | 35.4(9) | 3.3(9) | 5.3(8) | -9.8(10) |
| C15 | 74.6(13) | 71.8(14) | 38.1(10) | -8.1(9) | 13.7(9) | -5.1(11) |
| C16 | 68.5(12) | 55.7(11) | 48.9(11) | -5.9(9) | 10.1(9) | 2.1(9) |
| C17 | 59.6(11) | 52.9(10) | 39.0(9) | 2.0(8) | 4.5(8) | -1.7(8) |
| O1S | 317(17) | 180(11) | 228(15) | 8(12) | 55(13) | -9(12) |
| O2S | 169(8) | 136(10) | 100(6) | 1(8) | 8(7) | -33(7) |
| C1S | 120(12) | 145(15) | 59(7) | -2(9) | 4(11) | -59(11) |
| C2S | 128(8) | 120(8) | 68(6) | -4(7) | 11(7) | -45(7) |
| C3S | 148(10) | 163(11) | 137(11) | -12(9) | 3(9) | 29(8) |
| C4S | 169(8) | 136(10) | 100(6) | 1(8) | 8(7) | -33(7) |

Table 4 Bond Lengths for **5b**.

| Atom | Atom | Length/Å | Atom | Atom | Length/Å |
|---|---|---|---|---|---|
| Cl1 | C4 | 1.7273(19) | C5 | C6 | 1.395(3) |
| O1 | C5 | 1.368(2) | C6 | C7 | 1.398(3) |
| O1 | C9 | 1.429(3) | C7 | C8 | 1.394(2) |

| Atom | Atom | Length/Å | Atom | Atom | Length/Å |
|---|---|---|---|---|---|
| O2 | C6 | 1.351(2) | C7 | C10 | 1.468(3) |
| O3 | C10 | 1.220(2) | C12 | C13 | 1.403(2) |
| O4 | C10 | 1.319(3) | C12 | C17 | 1.388(3) |
| O4 | C11 | 1.449(3) | C13 | C14 | 1.381(3) |
| O5 | N1 | 1.213(2) | C14 | C15 | 1.373(3) |
| O6 | N1 | 1.214(2) | C15 | C16 | 1.371(3) |
| N1 | C13 | 1.465(3) | C16 | C17 | 1.380(3) |
| C1 | C2 | 1.319(2) | O1S | C2S | 1.111(12) |
| C1 | C12 | 1.473(2) | O2S | C2S | 1.293(13) |
| C2 | C3 | 1.463(2) | O2S | C3S | 1.437(14) |
| C3 | C4 | 1.410(2) | C1S | C2S | 1.457(12) |
| C3 | C8 | 1.384(3) | C3S | C4S | 1.489(14) |
| C4 | C5 | 1.384(2) | | | |

Table 5 Bond Angles for **5b**.

| Atom | Atom | Atom | Angle/° | Atom | Atom | Atom | Angle/° |
|---|---|---|---|---|---|---|---|
| C5 | O1 | C9 | 116.53(16) | C8 | C7 | C10 | 121.75(18) |
| C10 | O4 | C11 | 117.02(17) | C3 | C8 | C7 | 122.34(18) |
| O5 | N1 | O6 | 121.9(2) | O3 | C10 | O4 | 122.3(2) |
| O5 | N1 | C13 | 119.66(17) | O3 | C10 | C7 | 123.7(2) |
| O6 | N1 | C13 | 118.43(18) | O4 | C10 | C7 | 114.07(16) |
| C2 | C1 | C12 | 122.07(17) | C13 | C12 | C1 | 125.53(17) |
| C1 | C2 | C3 | 127.71(18) | C17 | C12 | C1 | 119.23(16) |
| C4 | C3 | C2 | 120.81(16) | C17 | C12 | C13 | 115.22(16) |
| C8 | C3 | C2 | 122.69(16) | C12 | C13 | N1 | 121.40(16) |
| C8 | C3 | C4 | 116.50(16) | C14 | C13 | N1 | 115.79(17) |
| C3 | C4 | Cl1 | 120.21(13) | C14 | C13 | C12 | 122.77(18) |
| C5 | C4 | Cl1 | 117.06(14) | C15 | C14 | C13 | 119.60(18) |

| Atom | Atom | Atom | Angle/° | Atom | Atom | Atom | Angle/° |
|---|---|---|---|---|---|---|---|
| C5 | C4 | C3 | 122.73(17) | C16 | C15 | C14 | 119.49(18) |
| O1 | C5 | C4 | 119.47(18) | C15 | C16 | C17 | 120.35(19) |
| O1 | C5 | C6 | 121.31(16) | C16 | C17 | C12 | 122.54(18) |
| C4 | C5 | C6 | 119.10(17) | C2S | O2S | C3S | 138.5(17) |
| O2 | C6 | C5 | 117.19(18) | O1S | C2S | O2S | 108.3(18) |
| O2 | C6 | C7 | 123.13(19) | O1S | C2S | C1S | 129.3(15) |
| C5 | C6 | C7 | 119.68(16) | O2S | C2S | C1S | 122.3(15) |
| C6 | C7 | C10 | 118.64(16) | O2S | C3S | C4S | 107.2(13) |
| C8 | C7 | C6 | 119.60(18) | | | | |

Table 6 Hydrogen Bonds for **5b**.

| D | H | A | d(D-H)/Å | d(H-A)/Å | d(D-A)/Å | D-H-A/° |
|---|---|---|---|---|---|---|
| O2 | H2A | O3 | 0.85(3) | 1.79(3) | 2.588(3) | 155(3) |

Table 7 Torsion Angles for **5b**.

| A | B | C | D | Angle/° | A | B | C | D | Angle/° |
|---|---|---|---|---|---|---|---|---|---|
| Cl1 | C4 | C5 | O1 | -2.5(2) | C5 | C6 | C7 | C8 | 1.9(3) |
| Cl1 | C4 | C5 | C6 | -178.38(14) | C5 | C6 | C7 | C10 | -178.54(17) |
| O1 | C5 | C6 | O2 | 1.4(3) | C6 | C7 | C8 | C3 | -0.4(3) |
| O1 | C5 | C6 | C7 | -178.18(17) | C6 | C7 | C10 | O3 | -2.2(3) |
| O2 | C6 | C7 | C8 | -177.65(18) | C6 | C7 | C10 | O4 | 177.80(18) |
| O2 | C6 | C7 | C10 | 1.9(3) | C8 | C3 | C4 | Cl1 | 179.88(13) |
| O5 | N1 | C13 | C12 | -20.3(3) | C8 | C3 | C4 | C5 | 0.3(3) |
| O5 | N1 | C13 | C14 | 157.6(2) | C8 | C7 | C10 | O3 | 177.3(2) |
| O6 | N1 | C13 | C12 | 162.0(2) | C8 | C7 | C10 | O4 | -2.7(3) |
| O6 | N1 | C13 | C14 | -20.1(3) | C9 | O1 | C5 | C4 | 119.8(2) |
| N1 | C13 | C14 | C15 | -177.12(19) | C9 | O1 | C5 | C6 | -64.4(3) |
| C1 | C2 | C3 | C4 | 175.05(19) | C10 | C7 | C8 | C3 | -179.90(18) |
| C1 | C2 | C3 | C8 | -5.6(3) | C11 | O4 | C10 | O3 | 0.0(3) |

| A | B | C | D | Angle/° | A | B | C | D | Angle/° |
|---|---|---|---|---|---|---|---|---|---|
| C1 | C12 | C13 | N1 | -3.0(3) | C11 | O4 | C10 | C7 | -179.91(18) |
| C1 | C12 | C13 | C14 | 179.35(18) | C12 | C1 | C2 | C3 | 178.66(17) |
| C1 | C12 | C17 | C16 | 179.36(18) | C12 | C13 | C14 | C15 | 0.7(3) |
| C2 | C1 | C12 | C13 | 145.5(2) | C13 | C12 | C17 | C16 | -2.0(3) |
| C2 | C1 | C12 | C17 | -36.0(3) | C13 | C14 | C15 | C16 | -1.0(3) |
| C2 | C3 | C4 | Cl1 | -0.7(2) | C14 | C15 | C16 | C17 | -0.1(3) |
| C2 | C3 | C4 | C5 | 179.70(17) | C15 | C16 | C17 | C12 | 1.7(3) |
| C2 | C3 | C8 | C7 | 179.90(17) | C17 | C12 | C13 | N1 | 178.47(17) |
| C3 | C4 | C5 | O1 | 177.15(17) | C17 | C12 | C13 | C14 | 0.8(3) |
| C3 | C4 | C5 | C6 | 1.2(3) | C2S | O2S | C3S | C4S | -180(2) |
| C4 | C3 | C8 | C7 | -0.7(3) | C3S | O2S | C2S | O1S | 3(4) |
| C4 | C5 | C6 | O2 | 177.28(17) | C3S | O2S | C2S | C1S | -174(3) |
| C4 | C5 | C6 | C7 | -2.3(3) | | | | | |

Table 8 Hydrogen Atom Coordinates (Å×$10^4$) and Isotropic Displacement Parameters (Å$^2$×$10^3$) for **5b**.

| Atom | *x* | *y* | *z* | U(eq) |
|---|---|---|---|---|
| H2A | 3960(70) | 2911(17) | 7161(10) | 85(9) |
| H1 | 8519.85 | 2765.3 | 4759.8 | 59 |
| H2 | 6483.19 | 1286.73 | 4927.88 | 58 |
| H8 | 7843.14 | 3058.81 | 5596.63 | 58 |
| H9A | 6214.56 | 756.11 | 7105.04 | 114 |
| H9B | 2991.69 | 258.35 | 7239.27 | 114 |
| H9C | 3305.45 | 1103.4 | 7446.46 | 114 |
| H11A | 10649.52 | 5105.72 | 6191.61 | 119 |
| H11B | 10493.5 | 4787.57 | 6765.57 | 119 |
| H11C | 7164.24 | 5124.12 | 6486.87 | 119 |
| H14 | 9080.17 | 2437.03 | 2897.47 | 69 |
| H15 | 11081.11 | 1194.41 | 2826.36 | 73 |

| Atom | *x* | *y* | *z* | U(eq) |
|---|---|---|---|---|
| H16 | 11806.47 | 453.52 | 3569.56 | 69 |
| H17 | 10597.69 | 943.75 | 4376.41 | 61 |
| H1SA | 15702.32 | 4926.28 | 5122.13 | 162 |
| H1SB | 13300.95 | 5626.1 | 5237.64 | 162 |
| H1SC | 14010.95 | 5395.23 | 4656.58 | 162 |
| H3SA | 3886.24 | 4377.16 | 5364.22 | 179 |
| H3SB | 3633.17 | 4299.09 | 4748.83 | 179 |
| H4SA | 2627.31 | 5700.78 | 4779.08 | 203 |
| H4SB | 1497.39 | 5530.1 | 5352.5 | 203 |
| H4SC | -489.4 | 5150.38 | 4874.47 | 203 |

Table 9 Atomic Occupancy for **5b**.

| Atom | *Occupancy* | Atom | *Occupancy* | Atom | *Occupancy* |
|---|---|---|---|---|---|
| O1S | 0.233(2) | O2S | 0.233(2) | C1S | 0.233(2) |
| H1SA | 0.233(2) | H1SB | 0.233(2) | H1SC | 0.233(2) |
| C2S | 0.233(2) | C3S | 0.233(2) | H3SA | 0.233(2) |
| H3SB | 0.233(2) | C4S | 0.233(2) | H4SA | 0.233(2) |
| H4SB | 0.233(2) | H4SC | 0.233(2) | | |

**X-Ray Structure Determination data of 6b.**

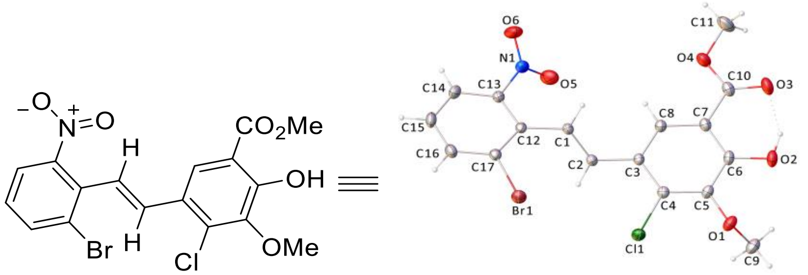


**Crystal Data** for $C_{17}H_{13}BrClNO_6$ (*M* =442.64 g/mol): triclinic, space group P-1 (no. 2), *a* = 3.8821(2) Å, *b* = 12.8863(6) Å, *c* = 18.4419(8) Å, *α* = 71.7550(10)°, *β* = 88.923(2)°, *γ* = 81.450(2)°, *V* = 866.08(7) Å$^3$, *Z* = 2, *T* = 300(2) K, μ(MoKα) = 2.562 mm$^{-1}$, *Dcalc* = 1.697 g/cm$^3$, 16839 reflections measured (4.654° ≤ 2Θ ≤ 52.778°), 3529 unique ($R_{int}$ = 0.0534, $R_{sigma}$ = 0.0389) which were used in all calculations. The final $R_1$ was 0.0368 (I > 2σ(I)) and $wR_2$ was 0.0793 (all data).

Table 1 Crystal data and structure refinement for **6b**.

| | |
|---|---|
| Empirical formula | $C_{17}H_{13}BrClNO_6$ |
| Formula weight | 442.64 |
| Temperature/K | 300(2) |
| Crystal system | triclinic |
| Space group | P-1 |
| a/Å | 3.8821(2) |
| b/Å | 12.8863(6) |
| c/Å | 18.4419(8) |
| α/° | 71.7550(10) |
| β/° | 88.923(2) |
| γ/° | 81.450(2) |
| Volume/Å$^3$ | 866.08(7) |
| Z | 2 |
| $\rho_{calc}$g/cm$^3$ | 1.697 |
| μ/mm$^{-1}$ | 2.562 |
| F(000) | 444.0 |
| Crystal size/mm$^3$ | 0.42 × 0.08 × 0.04 |
| Radiation | MoKα (λ = 0.71073) |
| 2Θ range for data collection/° | 4.654 to 52.778 |
| Index ranges | $-4 \leq h \leq 4$, $-16 \leq k \leq 16$, $-22 \leq l \leq 23$ |
| Reflections collected | 16839 |
| Independent reflections | 3529 [$R_{int}$ = 0.0534, $R_{sigma}$ = 0.0389] |
| Data/restraints/parameters | 3529/0/241 |
| Goodness-of-fit on F$^2$ | 1.048 |
| Final R indexes [I>=2σ (I)] | $R_1$ = 0.0368, w$R_2$ = 0.0710 |
| Final R indexes [all data] | $R_1$ = 0.0551, w$R_2$ = 0.0793 |
| Largest diff. peak/hole / e Å$^{-3}$ | 0.27/-0.34 |

Table 2 Fractional Atomic Coordinates (×10$^4$) and Equivalent Isotropic Displacement Parameters

(Å$^2$×10$^3$) for **6b**. $U_{eq}$ is defined as 1/3 of of the trace of the orthogonalised $U_{IJ}$ tensor.

| **Atom** | ***x*** | ***y*** | ***z*** | **U(eq)** |
|---|---|---|---|---|
| Br1 | 3331.4(9) | 2326.2(3) | 1229.2(2) | 54.78(12) |
| Cl1 | 2215(2) | 5725.1(6) | 503.8(4) | 53.5(2) |
| O1 | 4640(6) | 7740.8(16) | 493.8(12) | 54.5(6) |
| O2 | 6058(7) | 8336.0(17) | 1730.4(15) | 60.5(6) |
| O3 | 6884(7) | 7748.7(19) | 3198.3(15) | 71.4(7) |
| O4 | 5356(7) | 6157.3(19) | 3918.1(13) | 63.7(6) |
| O5 | -29(8) | 2902(2) | 4091.4(14) | 78.4(8) |
| O6 | 2997(10) | 1397(2) | 4769.4(15) | 102.6(11) |
| N1 | 1487(8) | 1972(2) | 4179.8(14) | 57.2(7) |
| C1 | 3188(7) | 3266(2) | 2679.7(15) | 37.0(6) |
| C2 | 2007(7) | 4175(2) | 2120.4(15) | 36.9(6) |
| C3 | 3015(7) | 5264(2) | 2034.7(15) | 35.5(6) |
| C4 | 3178(7) | 6037(2) | 1311.2(15) | 37.5(6) |
| C5 | 4201(7) | 7060(2) | 1214.0(16) | 41.3(7) |
| C6 | 5046(8) | 7335(2) | 1854.8(18) | 43.7(7) |
| C7 | 4836(7) | 6587(2) | 2584.9(17) | 41.7(6) |
| C8 | 3823(7) | 5564(2) | 2662.2(16) | 40.0(6) |
| C9 | 2204(9) | 8728(2) | 237(2) | 59.0(9) |
| C10 | 5784(8) | 6892(3) | 3250.0(19) | 49.9(8) |
| C11 | 6273(12) | 6441(4) | 4589(2) | 84.6(13) |
| C12 | 2215(7) | 2165(2) | 2808.6(15) | 35.6(6) |
| C13 | 1503(8) | 1513(2) | 3541.3(16) | 43.1(7) |
| C14 | 712(9) | 455(3) | 3724.6(19) | 55.7(8) |
| C15 | 583(9) | -1(3) | 3150(2) | 60.0(9) |
| C16 | 1281(9) | 589(2) | 2414.3(19) | 53.1(8) |
| C17 | 2137(7) | 1650(2) | 2245.7(16) | 40.2(6) |

Table 3 Anisotropic Displacement Parameters (Å$^2$×10$^3$) for **6b**. The Anisotropic displacement

factor exponent takes the form: $-2\pi^2[h^2a^{*2}U_{11}+2hka^*b^*U_{12}+\ldots]$.

| **Atom** | **$U_{11}$** | **$U_{22}$** | **$U_{33}$** | **$U_{23}$** | **$U_{13}$** | **$U_{12}$** |
|---|---|---|---|---|---|---|
| Br1 | 64.4(2) | 60.4(2) | 43.28(18) | -20.71(15) | 7.79(14) | -12.14(16) |
| Cl1 | 70.8(6) | 50.4(4) | 40.2(4) | -14.4(3) | 0.6(4) | -11.8(4) |
| O1 | 62.9(14) | 36.3(11) | 55.0(13) | -4.4(10) | 15.5(11) | -1.9(10) |
| O2 | 74.1(17) | 38.9(12) | 75.3(17) | -21.1(12) | 14.5(13) | -24.6(11) |
| O3 | 88.6(19) | 61.0(15) | 84.5(17) | -41.4(14) | 7.8(14) | -33.4(14) |
| O4 | 82.5(18) | 70.0(15) | 52.1(13) | -30.6(12) | 2.8(12) | -28.8(13) |
| O5 | 106(2) | 76.7(18) | 58.4(15) | -35.0(14) | -0.3(14) | -1.5(16) |
| O6 | 174(3) | 79.9(19) | 47.6(15) | -7.5(14) | -32.4(18) | -24(2) |
| N1 | 80(2) | 55.7(17) | 37.3(14) | -9.7(13) | 2.2(14) | -26.2(16) |
| C1 | 37.5(16) | 36.7(14) | 39.2(14) | -13.2(12) | -0.2(12) | -10.2(12) |
| C2 | 36.8(15) | 37.7(14) | 39.4(14) | -14.9(12) | 1.3(12) | -9.3(12) |
| C3 | 35.3(15) | 31.0(13) | 41.4(14) | -13.3(12) | 2.2(12) | -4.9(11) |
| C4 | 33.9(15) | 36.1(14) | 42.7(15) | -14.1(12) | 3.5(12) | -2.5(11) |
| C5 | 42.8(17) | 32.2(14) | 45.8(16) | -8.9(13) | 11.7(13) | -4.5(12) |
| C6 | 41.2(17) | 31.5(14) | 60.5(18) | -16.2(14) | 10.0(14) | -8.7(12) |
| C7 | 39.1(16) | 39.0(15) | 51.9(17) | -20.8(14) | 4.8(13) | -7.7(12) |
| C8 | 41.9(16) | 35.4(14) | 43.8(15) | -13.5(12) | 3.9(13) | -7.0(12) |
| C9 | 59(2) | 40.9(17) | 67(2) | -3.5(16) | 4.5(17) | -3.5(15) |
| C10 | 47.5(18) | 49.7(18) | 63(2) | -30.7(16) | 6.5(15) | -12.9(14) |
| C11 | 107(3) | 109(3) | 64(2) | -52(2) | 4(2) | -42(3) |
| C12 | 32.6(15) | 32.5(13) | 40.4(14) | -9.2(12) | -2.9(11) | -5.4(11) |
| C13 | 47.4(18) | 39.9(15) | 42.5(15) | -11.7(13) | -3.2(13) | -11.1(13) |
| C14 | 65(2) | 43.7(17) | 53.3(19) | -2.4(15) | 0.4(16) | -21.3(16) |
| C15 | 68(2) | 35.7(16) | 78(2) | -13.6(17) | -2.7(18) | -19.5(15) |
| C16 | 60(2) | 45.3(17) | 61(2) | -25.3(16) | -5.2(16) | -12.1(15) |
| C17 | 38.5(16) | 38.9(15) | 43.3(15) | -12.5(13) | -1.0(12) | -6.0(12) |

Table 4 Bond Lengths for **6b**.

| Atom | Atom | Length/Å | Atom | Atom | Length/Å |
|---|---|---|---|---|---|
| Br1 | C17 | 1.885(3) | C3 | C4 | 1.400(4) |
| Cl1 | C4 | 1.720(3) | C3 | C8 | 1.384(4) |
| O1 | C5 | 1.367(3) | C4 | C5 | 1.390(4) |
| O1 | C9 | 1.421(4) | C5 | C6 | 1.394(4) |
| O2 | C6 | 1.354(3) | C6 | C7 | 1.398(4) |
| O3 | C10 | 1.218(3) | C7 | C8 | 1.396(4) |
| O4 | C10 | 1.323(4) | C7 | C10 | 1.468(4) |
| O4 | C11 | 1.459(4) | C12 | C13 | 1.397(4) |
| O5 | N1 | 1.217(3) | C12 | C17 | 1.399(4) |
| O6 | N1 | 1.210(4) | C13 | C14 | 1.378(4) |
| N1 | C13 | 1.473(4) | C14 | C15 | 1.369(5) |
| C1 | C2 | 1.322(4) | C15 | C16 | 1.375(5) |
| C1 | C12 | 1.470(3) | C16 | C17 | 1.393(4) |
| C2 | C3 | 1.472(3) | | | |

Table 5 Bond Angles for **6b**.

| Atom | Atom | Atom | Angle/° | Atom | Atom | Atom | Angle/° |
|---|---|---|---|---|---|---|---|
| C5 | O1 | C9 | 116.2(2) | C6 | C7 | C10 | 118.8(3) |
| C10 | O4 | C11 | 115.7(3) | C8 | C7 | C6 | 119.4(3) |
| O5 | N1 | C13 | 118.9(3) | C8 | C7 | C10 | 121.8(3) |
| O6 | N1 | O5 | 123.3(3) | C3 | C8 | C7 | 121.9(3) |
| O6 | N1 | C13 | 117.8(3) | O3 | C10 | O4 | 122.1(3) |
| C2 | C1 | C12 | 126.4(3) | O3 | C10 | C7 | 123.3(3) |
| C1 | C2 | C3 | 123.8(3) | O4 | C10 | C7 | 114.6(3) |
| C4 | C3 | C2 | 120.9(2) | C13 | C12 | C1 | 120.7(2) |
| C8 | C3 | C2 | 121.6(2) | C13 | C12 | C17 | 114.4(2) |
| C8 | C3 | C4 | 117.5(2) | C17 | C12 | C1 | 124.7(2) |
| C3 | C4 | Cl1 | 120.3(2) | C12 | C13 | N1 | 119.3(2) |

| Atom | Atom | Atom | Angle/° | Atom | Atom | Atom | Angle/° |
|---|---|---|---|---|---|---|---|
| C5 | C4 | Cl1 | 117.7(2) | C14 | C13 | N1 | 115.6(3) |
| C5 | C4 | C3 | 122.0(2) | C14 | C13 | C12 | 125.1(3) |
| O1 | C5 | C4 | 119.6(3) | C15 | C14 | C13 | 118.2(3) |
| O1 | C5 | C6 | 120.9(2) | C14 | C15 | C16 | 120.0(3) |
| C4 | C5 | C6 | 119.3(2) | C15 | C16 | C17 | 120.7(3) |
| O2 | C6 | C5 | 117.1(3) | C12 | C17 | Br1 | 122.1(2) |
| O2 | C6 | C7 | 123.1(3) | C16 | C17 | Br1 | 116.3(2) |
| C5 | C6 | C7 | 119.9(2) | C16 | C17 | C12 | 121.5(3) |

Table 6 Hydrogen Bonds for **6b**.

| D | H | A | d(D-H)/Å | d(H-A)/Å | d(D-A)/Å | D-H-A/° |
|---|---|---|---|---|---|---|
| O2 | H2A | O3 | 0.92(4) | 1.74(4) | 2.585(4) | 151(4) |

Table 7 Torsion Angles for **6b**.

| A | B | C | D | Angle/° | A | B | C | D | Angle/° |
|---|---|---|---|---|---|---|---|---|---|
| Cl1 | C4 | C5 | O1 | -4.6(4) | C4 | C5 | C6 | C7 | -0.4(4) |
| Cl1 | C4 | C5 | C6 | -179.2(2) | C5 | C6 | C7 | C8 | 0.9(4) |
| O1 | C5 | C6 | O2 | 4.8(4) | C5 | C6 | C7 | C10 | 179.2(3) |
| O1 | C5 | C6 | C7 | -175.0(3) | C6 | C7 | C8 | C3 | 0.0(4) |
| O2 | C6 | C7 | C8 | -178.9(3) | C6 | C7 | C10 | O3 | -3.0(5) |
| O2 | C6 | C7 | C10 | -0.6(4) | C6 | C7 | C10 | O4 | 177.1(3) |
| O5 | N1 | C13 | C12 | 45.8(4) | C8 | C3 | C4 | Cl1 | 180.0(2) |
| O5 | N1 | C13 | C14 | -132.8(3) | C8 | C3 | C4 | C5 | 1.7(4) |
| O6 | N1 | C13 | C12 | -134.3(3) | C8 | C7 | C10 | O3 | 175.3(3) |
| O6 | N1 | C13 | C14 | 47.2(4) | C8 | C7 | C10 | O4 | -4.6(4) |
| N1 | C13 | C14 | C15 | 178.1(3) | C9 | O1 | C5 | C4 | 112.1(3) |
| C1 | C2 | C3 | C4 | 148.7(3) | C9 | O1 | C5 | C6 | -73.4(4) |
| C1 | C2 | C3 | C8 | -31.5(4) | C10 | C7 | C8 | C3 | -178.3(3) |
| C1 | C12 | C13 | N1 | 4.4(4) | C11 | O4 | C10 | O3 | 0.2(5) |

| A | B | C | D | Angle/° | A | B | C | D | Angle/° |
|---|---|---|---|---|---|---|---|---|---|
| C1 | C12 | C13 | C14 | -177.2(3) | C11 | O4 | C10 | C7 | -179.9(3) |
| C1 | C12 | C17 | Br1 | 0.4(4) | C12 | C1 | C2 | C3 | 178.3(3) |
| C1 | C12 | C17 | C16 | 178.2(3) | C12 | C13 | C14 | C15 | -0.4(5) |
| C2 | C1 | C12 | C13 | -136.1(3) | C13 | C12 | C17 | Br1 | -175.5(2) |
| C2 | C1 | C12 | C17 | 48.2(4) | C13 | C12 | C17 | C16 | 2.3(4) |
| C2 | C3 | C4 | Cl1 | -0.3(4) | C13 | C14 | C15 | C16 | 0.9(5) |
| C2 | C3 | C4 | C5 | -178.5(3) | C14 | C15 | C16 | C17 | 0.3(5) |
| C2 | C3 | C8 | C7 | 178.9(3) | C15 | C16 | C17 | Br1 | 175.9(3) |
| C3 | C4 | C5 | O1 | 173.7(2) | C15 | C16 | C17 | C12 | -2.0(5) |
| C3 | C4 | C5 | C6 | -0.9(4) | C17 | C12 | C13 | N1 | -179.6(3) |
| C4 | C3 | C8 | C7 | -1.3(4) | C17 | C12 | C13 | C14 | -1.1(4) |
| C4 | C5 | C6 | O2 | 179.4(3) | | | | | |

Table 8 Hydrogen Atom Coordinates (Å×$10^4$) and Isotropic Displacement Parameters (Å$^2$×$10^3$) for **6b**.

| Atom | *x* | *y* | *z* | U(eq) |
|---|---|---|---|---|
| H2A | 6490(100) | 8370(30) | 2210(20) | 82(13) |
| H1 | 4792.22 | 3330.53 | 3025.63 | 44 |
| H2 | 450.05 | 4124.35 | 1760.3 | 44 |
| H8 | 3687.17 | 5069.13 | 3149.08 | 48 |
| H9A | 2868.92 | 9176.56 | -251.87 | 89 |
| H9B | 2174.12 | 9129.14 | 597.22 | 89 |
| H9C | -75.01 | 8546.84 | 191.11 | 89 |
| H11A | 8696.93 | 6518.02 | 4580.41 | 127 |
| H11B | 5837.11 | 5866.63 | 5042.78 | 127 |
| H11C | 4888.89 | 7125.64 | 4584.43 | 127 |
| H14 | 276.98 | 61.96 | 4225.45 | 67 |
| H15 | 24.13 | -709.63 | 3255.92 | 72 |

| Atom | *x* | *y* | *z* | U(eq) |
|---|---|---|---|---|
| H16 | 1179.93 | 276.56 | 2025.43 | 64 |

**X-Ray Structure Determination data of 12b.**

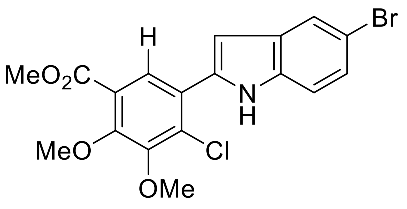


**12b**

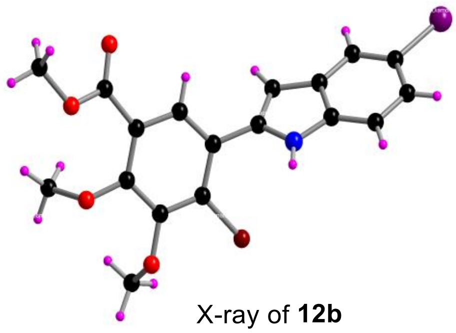

X-ray of **12b**

**Crystal Data** for $C_{18}H_{15}NO_4ClBr$ (*M* =424.68 g/mol): monoclinic, Space group $P2_1/c$, $a$ = 11.4876(3) Å, $b$ = 6.85064(16) Å, $c$ = 22.4379(6) Å, $\alpha$ = 90°, $\beta$ = 94.426(3)°, $\gamma$ = 90°, $V$ = 1760.54(8) Å$^3$, $Z$ = 4, $T$ = 300 K, μ(Cu Kα) = 4.784 mm$^{-1}$, *Dcalc* = 1.6021 g/cm$^3$, 6007 reflections measured (7.72° ≤ 2Θ ≤ 139.8°), 3238 [$R_{int}$ = 0.0180, $R_{sigma}$ = 0.0272] which were used in all calculations. The final $R_1$ was 0.0774 (I>=2u(I)) and $wR_2$ was 0.2059 (all data).

Single crystal X-ray data for **12b** compound was collected at room temperature. A suitable crystal of **12b** was selected and mounted on the SuperNova (Dual, Cu at zero, Eos) diffractometer. The crystal was kept at 298 K during data collection. Using Olex2,[5] the structure was solved with the olex2.solve[6] structure solution program using Charge Flipping and refined with the olex2.refine[7] refinement package using the Gauss-Newton minimization. The absorption corrections were performed on the basis of multi-scans. The non-hydrogen atoms were anisotropically refined. The hydrogen atoms were included in the refinement at the calculated positions riding on their carrier atoms.

| Table 1 Crystal data and structure refinement for **12b**. | |
|---|---|
| Identification code | exp_8897 |
| Empirical formula | $C_{18}H_{15}NO_4ClBr$ |
| Formula weight | 424.68 |
| Temperature/K | 293 |
| Crystal system | monoclinic |
| Space group | $P2_1/c$ |
| a/Å | 11.4876(3) |
| b/Å | 6.85064(16) |
| c/Å | 22.4379(6) |
| α/° | 90 |
| β/° | 94.426(3) |
| γ/° | 90 |

| | |
|---|---|
| Volume/Å$^3$ | 1760.54(8) |
| Z | 4 |
| $\rho_{calc}$g/cm$^3$ | 1.6021 |
| μ/mm$^{-1}$ | 4.784 |
| F(000) | 857.1 |
| Crystal size/mm$^3$ | 0.09 × 0.07 × 0.05 |
| Radiation | Cu Kα (λ = 1.54184) |
| 2Θ range for data collection/° | 7.72 to 139.8 |
| Index ranges | -11 ≤ h ≤ 13, -8 ≤ k ≤ 5, -27 ≤ l ≤ 26 |
| Reflections collected | 6007 |
| Independent reflections | 3238 [$R_{int}$ = 0.0180, $R_{sigma}$ = 0.0272] |
| Data/restraints/parameters | 3238/0/229 |
| Goodness-of-fit on F$^2$ | 1.039 |
| Final R indexes [I>=2σ (I)] | $R_1$ = 0.0637, w$R_2$ = 0.1875 |
| Final R indexes [all data] | $R_1$ = 0.0774, w$R_2$ = 0.2059 |
| Largest diff. peak/hole / e Å$^{-3}$ | 1.85/-0.67 |

Table 2 Fractional Atomic Coordinates (×10$^4$) and Equivalent Isotropic Displacement Parameters (Å$^2$×10$^3$) for **12b**. $U_{eq}$ is defined as 1/3 of the trace of the orthogonalised $U_{IJ}$ tensor.

| Atom | *x* | *y* | *z* | U(eq) |
|---|---|---|---|---|
| Br1 | 8935.5(7) | 7917.4(12) | 167.2(3) | 95.5(3) |
| Cl1 | 14509.3(12) | 7046(2) | 2830.5(8) | 82.6(5) |
| N1 | 11752(3) | 6893(5) | 2495.9(17) | 52.5(8) |
| O3 | 12015(3) | -1279(5) | 3612.5(15) | 65.6(9) |
| O2 | 15652(3) | 5013(5) | 3858.4(15) | 61.5(8) |
| O1 | 15003(4) | 1613(5) | 4326.1(16) | 72.6(10) |
| C3 | 13986(4) | 4959(6) | 3149(2) | 53.7(10) |
| C18 | 11052(4) | 7395(6) | 2003(2) | 50.3(10) |
| C15 | 9827(4) | 7673(7) | 916(2) | 59.8(11) |
| C11 | 12178(4) | 5046(7) | 2430(2) | 53.5(10) |
| C14 | 10408(4) | 5972(7) | 1047(2) | 61.7(12) |
| C12 | 11755(4) | 4333(7) | 1893(2) | 59.9(11) |
| C4 | 12933(4) | 4114(6) | 2911(2) | 52.3(10) |
| C5 | 12606(4) | 2392(7) | 3159(2) | 54.2(10) |

| | | | | |
|---|---|---|---|---|
| C1 | 14313(4) | 2360(7) | 3864(2) | 55.7(11) |
| C7 | 12750(4) | -301(7) | 3883(2) | 60.7(11) |
| C16 | 9839(4) | 9258(7) | 1311(2) | 58.9(11) |
| O4 | 13126(4) | -721(7) | 4438.6(18) | 99.8(16) |
| C6 | 13265(4) | 1487(7) | 3638.1(19) | 55.6(10) |
| C13 | 11035(4) | 5800(6) | 1602(2) | 52.3(10) |
| C2 | 14662(4) | 4106(6) | 3619(2) | 53.0(10) |
| C10 | 16710(4) | 4333(10) | 3644(3) | 80.1(16) |
| C17 | 10449(4) | 9129(6) | 1860(2) | 53.3(10) |
| C9 | 15678(9) | -317(14) | 4178(5) | 138(3) |
| C8 | 12649(8) | -2515(13) | 4674(4) | 133(4) |

Table 3 Anisotropic Displacement Parameters ($Å^2\times10^3$) for **12b**. The Anisotropic displacement factor exponent takes the form: $-2\pi^2[h^2a^{*2}U_{11}+2hka^*b^*U_{12}+\ldots]$.

| **Atom** | **$U_{11}$** | **$U_{22}$** | **$U_{33}$** | **$U_{12}$** | **$U_{13}$** | **$U_{23}$** |
|---|---|---|---|---|---|---|
| Br1 | 117.3(6) | 99.1(6) | 65.9(4) | 35.5(4) | -20.6(4) | -13.4(3) |
| Cl1 | 60.7(7) | 72.5(8) | 113.9(12) | -10.8(6) | 3.2(7) | 34.0(8) |
| N1 | 51.3(19) | 49.8(19) | 57(2) | 2.1(16) | 4.7(16) | -5.7(16) |
| O3 | 76(2) | 62.5(19) | 56.8(18) | -18.6(17) | -3.5(16) | -2.1(16) |
| O2 | 51.8(16) | 57.5(18) | 75(2) | -7.7(14) | 5.5(15) | -6.2(16) |
| O1 | 92(3) | 62.0(19) | 60(2) | -16.4(18) | -21.9(18) | 6.3(16) |
| C3 | 52(2) | 47(2) | 64(3) | -3.0(18) | 14(2) | 2(2) |
| C18 | 44(2) | 52(2) | 55(2) | -2.4(17) | 7.0(18) | -3.4(19) |
| C15 | 60(3) | 66(3) | 54(3) | 5(2) | 5(2) | -4(2) |
| C11 | 52(2) | 50(2) | 59(2) | -0.3(18) | 8.2(19) | 2(2) |
| C14 | 72(3) | 57(3) | 57(3) | 5(2) | 4(2) | -10(2) |
| C12 | 71(3) | 47(2) | 62(3) | 5(2) | 9(2) | -7(2) |
| C4 | 53(2) | 53(2) | 52(2) | 5.3(19) | 9.6(18) | 0.3(19) |
| C5 | 56(2) | 53(2) | 53(2) | -6.0(19) | 0.4(19) | -6.7(19) |
| C1 | 60(3) | 56(2) | 50(2) | -8(2) | -1(2) | -2.8(19) |
| C7 | 68(3) | 61(3) | 51(2) | -13(2) | -5(2) | 0(2) |
| C16 | 55(2) | 54(2) | 67(3) | 7(2) | 7(2) | -1(2) |

| | | | | | | |
|---|---|---|---|---|---|---|
| O4 | 120(3) | 105(3) | 69(2) | -62(3) | -33(2) | 33(2) |
| C6 | 67(3) | 54(2) | 45(2) | -12(2) | -1.1(19) | -2.1(19) |
| C13 | 55(2) | 48(2) | 54(2) | 1.2(19) | 9.5(19) | -7.8(19) |
| C2 | 50(2) | 54(2) | 56(2) | -3.1(19) | 9.5(19) | -10(2) |
| C10 | 55(3) | 99(4) | 87(4) | -2(3) | 10(3) | 7(3) |
| C17 | 49(2) | 48(2) | 64(3) | 2.7(18) | 6.7(19) | -10(2) |
| C9 | 151(8) | 116(7) | 147(8) | 33(6) | -4(6) | -4(6) |
| C8 | 154(7) | 143(7) | 93(5) | -92(6) | -44(5) | 63(5) |

Table 4 Bond Lengths for **12b.**

| Atom | Atom | Length/Å | Atom | Atom | Length/Å |
|---|---|---|---|---|---|
| Br1 | C15 | 1.907(5) | C15 | C16 | 1.400(7) |
| Cl1 | C3 | 1.727(4) | C11 | C12 | 1.354(7) |
| N1 | C18 | 1.360(6) | C11 | C4 | 1.477(6) |
| N1 | C11 | 1.369(6) | C14 | C13 | 1.395(6) |
| O3 | C7 | 1.205(5) | C12 | C13 | 1.428(6) |
| O2 | C2 | 1.369(5) | C4 | C5 | 1.369(6) |
| O2 | C10 | 1.420(6) | C5 | C6 | 1.409(7) |
| O1 | C1 | 1.356(6) | C1 | C6 | 1.404(6) |
| O1 | C9 | 1.581(10) | C1 | C2 | 1.388(7) |
| C3 | C4 | 1.407(6) | C7 | O4 | 1.320(6) |
| C3 | C2 | 1.389(6) | C7 | C6 | 1.483(6) |
| C18 | C13 | 1.415(6) | C16 | C17 | 1.372(7) |
| C18 | C17 | 1.400(6) | O4 | C8 | 1.460(7) |
| C15 | C14 | 1.363(7) | | | |

Table 5 Bond Angles for **12b.**

| Atom | Atom | Atom | Angle/° | Atom | Atom | Atom | Angle/° |
|---|---|---|---|---|---|---|---|
| C11 | N1 | C18 | 109.8(4) | C6 | C5 | C4 | 122.7(4) |
| C10 | O2 | C2 | 115.1(4) | C6 | C1 | O1 | 123.3(4) |
| C9 | O1 | C1 | 115.0(5) | C2 | C1 | O1 | 117.3(4) |
| C4 | C3 | Cl1 | 120.0(4) | C2 | C1 | C6 | 119.3(4) |
| C2 | C3 | Cl1 | 118.0(3) | O4 | C7 | O3 | 121.3(4) |

| | | | | | | | |
|---|---|---|---|---|---|---|---|
| C2 | C3 | C4 | 121.9(4) | C6 | C7 | O3 | 123.9(4) |
| C13 | C18 | N1 | 107.5(4) | C6 | C7 | O4 | 114.8(4) |
| C17 | C18 | N1 | 130.7(4) | C17 | C16 | C15 | 119.8(4) |
| C17 | C18 | C13 | 121.8(4) | C8 | O4 | C7 | 114.9(4) |
| C14 | C15 | Br1 | 119.0(4) | C1 | C6 | C5 | 118.9(4) |
| C16 | C15 | Br1 | 117.9(4) | C7 | C6 | C5 | 116.1(4) |
| C16 | C15 | C14 | 123.1(5) | C7 | C6 | C1 | 125.0(4) |
| C12 | C11 | N1 | 108.8(4) | C14 | C13 | C18 | 119.0(4) |
| C4 | C11 | N1 | 121.0(4) | C12 | C13 | C18 | 105.8(4) |
| C4 | C11 | C12 | 130.1(4) | C12 | C13 | C14 | 135.2(4) |
| C13 | C14 | C15 | 118.3(4) | C3 | C2 | O2 | 120.4(4) |
| C13 | C12 | C11 | 108.0(4) | C1 | C2 | O2 | 119.5(4) |
| C11 | C4 | C3 | 122.5(4) | C1 | C2 | C3 | 120.1(4) |
| C5 | C4 | C3 | 117.1(4) | C16 | C17 | C18 | 118.1(4) |
| C5 | C4 | C11 | 120.3(4) | | | | |

Table 6 Hydrogen Atom Coordinates ($Å\times10^4$) and Isotropic Displacement Parameters ($Å^2\times10^3$) for **12b.**

| **Atom** | ***x*** | ***y*** | ***z*** | **U(eq)** |
|---|---|---|---|---|
| H1 | 11904(3) | 7627(5) | 2803.1(17) | 63.0(10) |
| H14 | 10388(4) | 4953(7) | 772(2) | 74.0(14) |
| H12 | 11906(4) | 3104(7) | 1741(2) | 71.9(13) |
| H5 | 11921(4) | 1795(7) | 3005(2) | 65.0(12) |
| H16 | 9436(4) | 10395(7) | 1201(2) | 70.7(13) |
| H10a | 16770(20) | 4800(60) | 3245(9) | 120(2) |
| H10b | 17359(5) | 4800(60) | 3899(13) | 120(2) |
| H10c | 16714(19) | 2932(10) | 3640(20) | 120(2) |
| H17 | 10460(4) | 10162(6) | 2129(2) | 64.0(12) |
| H9a | 15460(60) | -700(70) | 3773(12) | 208(5) |
| H9b | 16504(9) | -80(40) | 4230(40) | 208(5) |
| H9c | 15480(60) | -1340(40) | 4440(30) | 208(5) |
| H8a | 12560(70) | -3470(40) | 4361(10) | 199(6) |

| | | | | |
|---|---|---|---|---|
| H8b | 13170(40) | -3000(70) | 4990(30) | 199(6) |
| H8c | 11900(30) | -2250(30) | 4820(30) | 199(6) |

**Copies of NMR spectra of new compounds**

**$^{1}$H NMR (400 MHz, $CDCl_3$), $^{13}$C NMR (100 MHz, $CDCl_3$) & $^{31}$P of 2a**

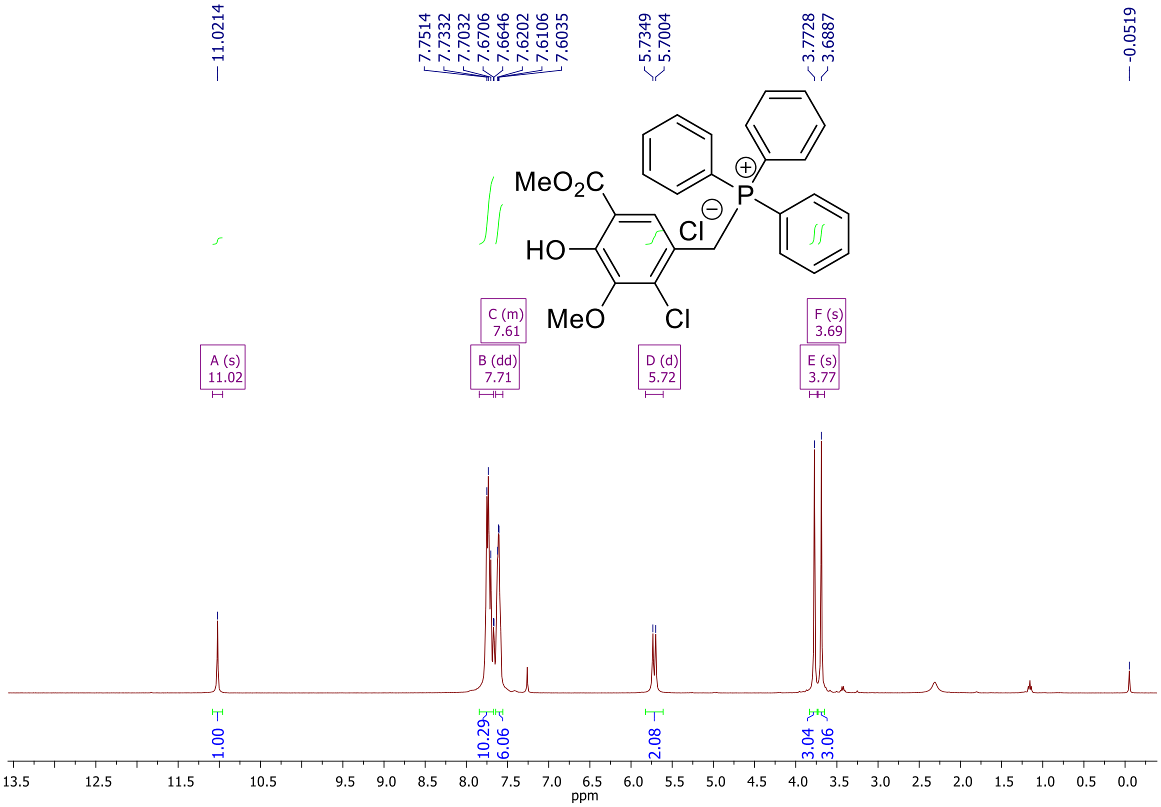

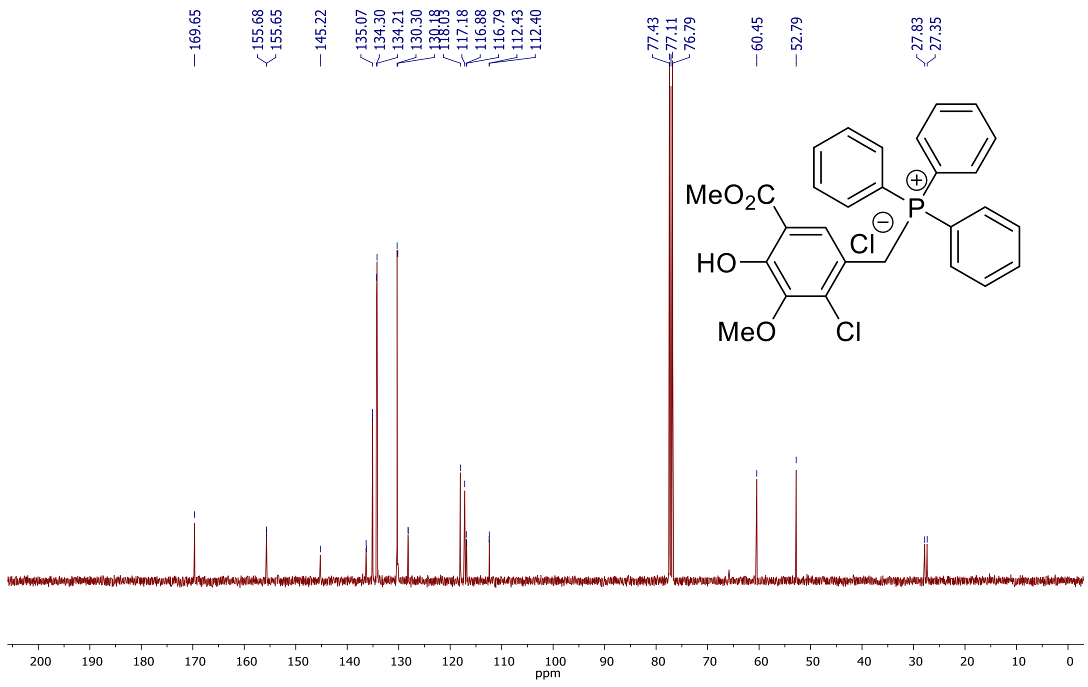

169.65
155.68
155.65
145.22
135.07
134.30
134.21
130.30
117.18
116.88
116.79
112.43
112.40
77.43
77.11
76.79
60.45
52.79
27.83
27.35
MeO2C
HO
MeO
Cl
P
ppm


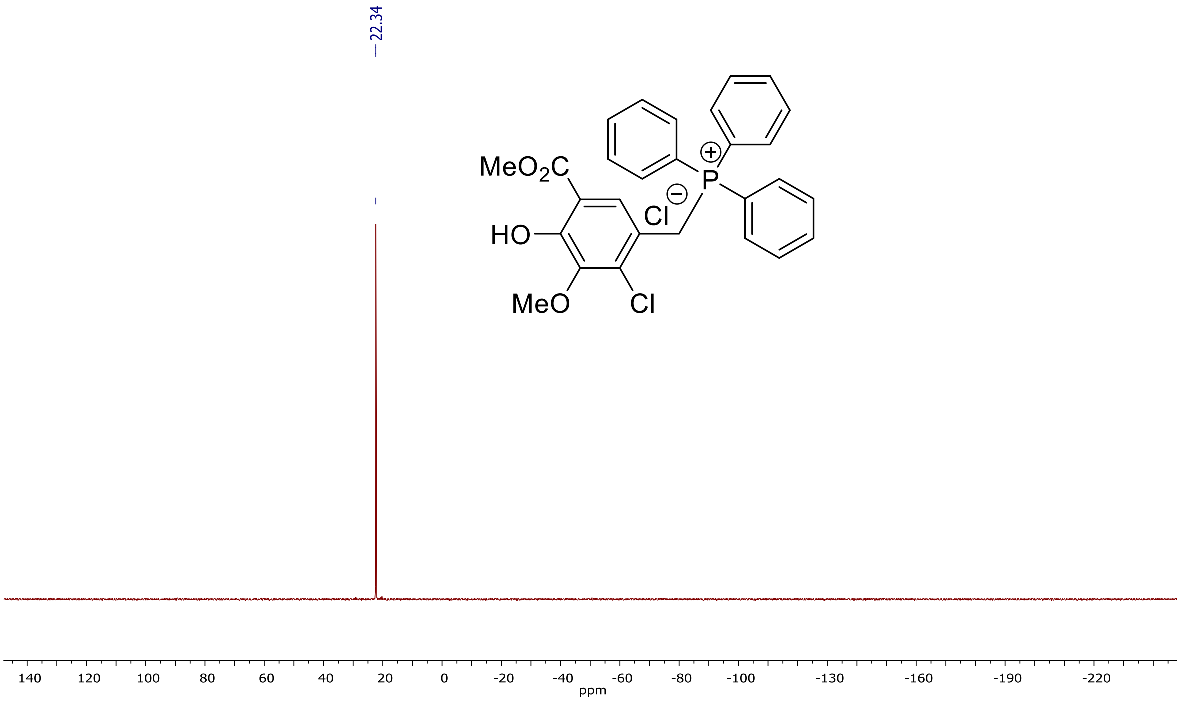

22.34
MeO2C
HO
MeO
Cl
P
ppm

**$^{1}H$ NMR (400 MHz), $^{13}C$ NMR (100 MHz,) and $^{31}P$ (162 MHz) in DMSO-D$_6$ of 2b**

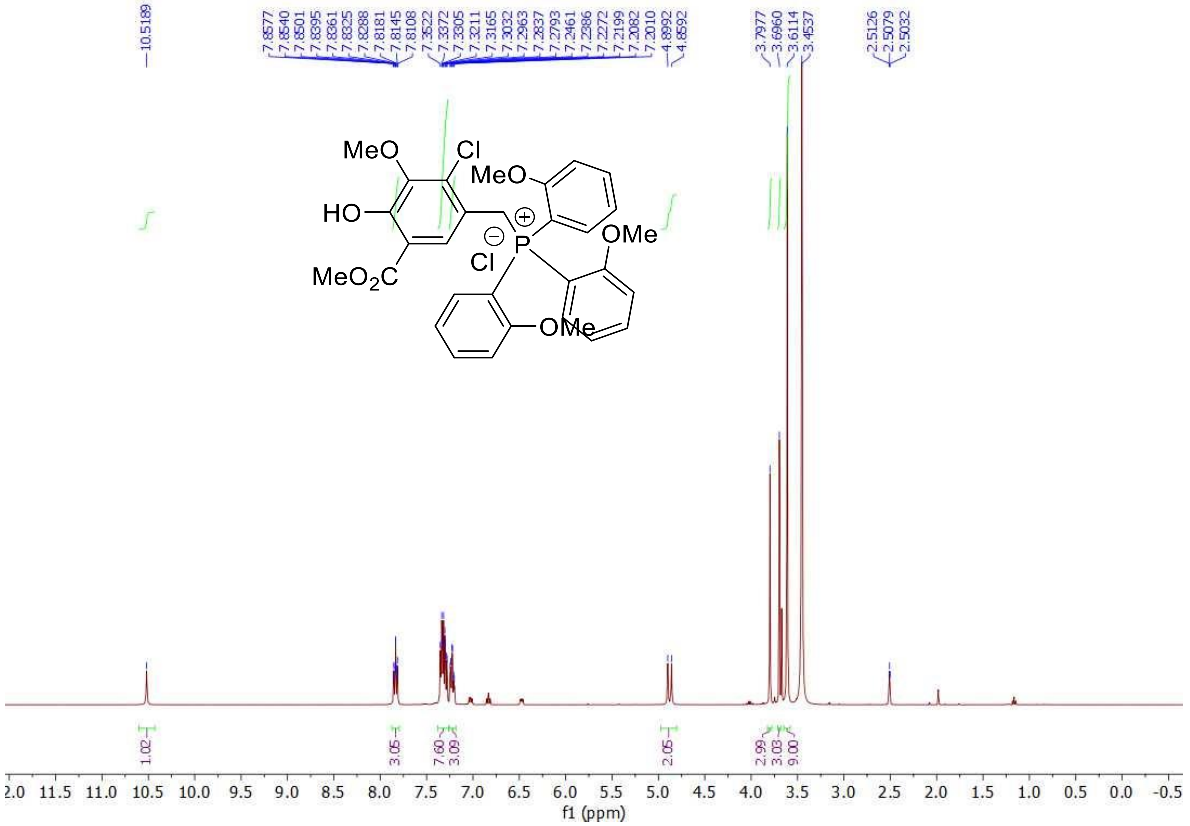


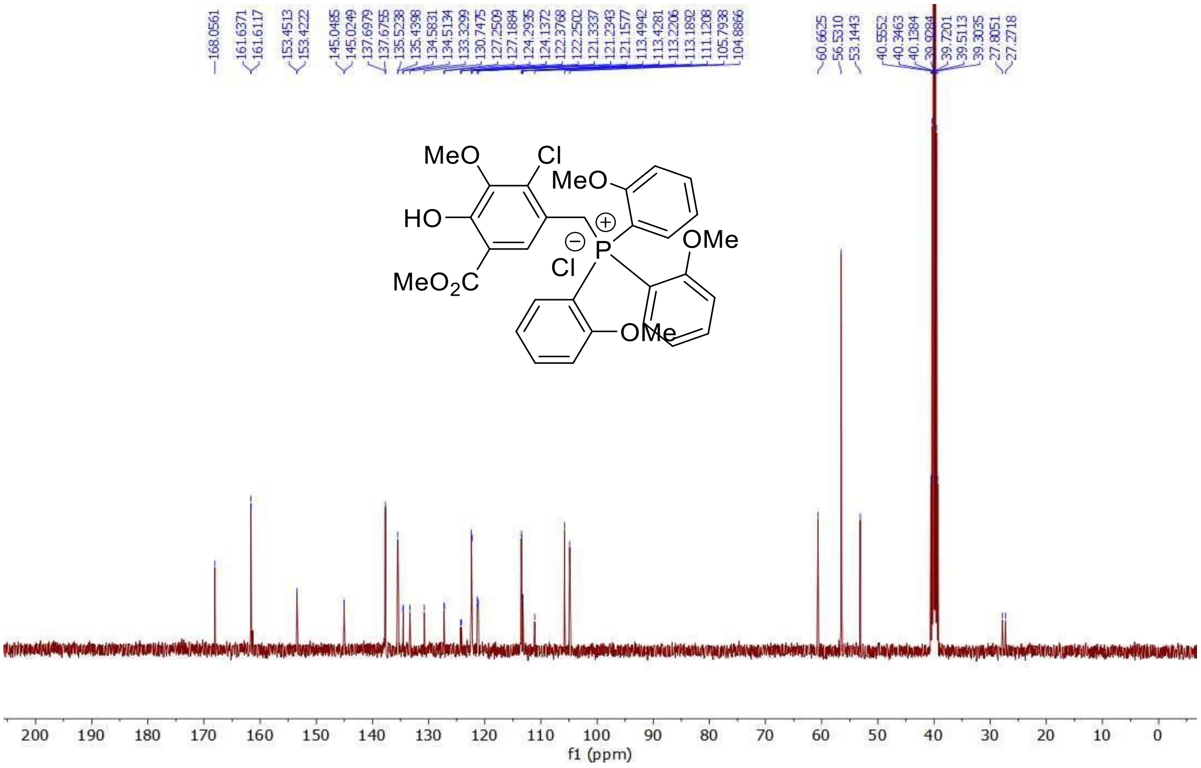

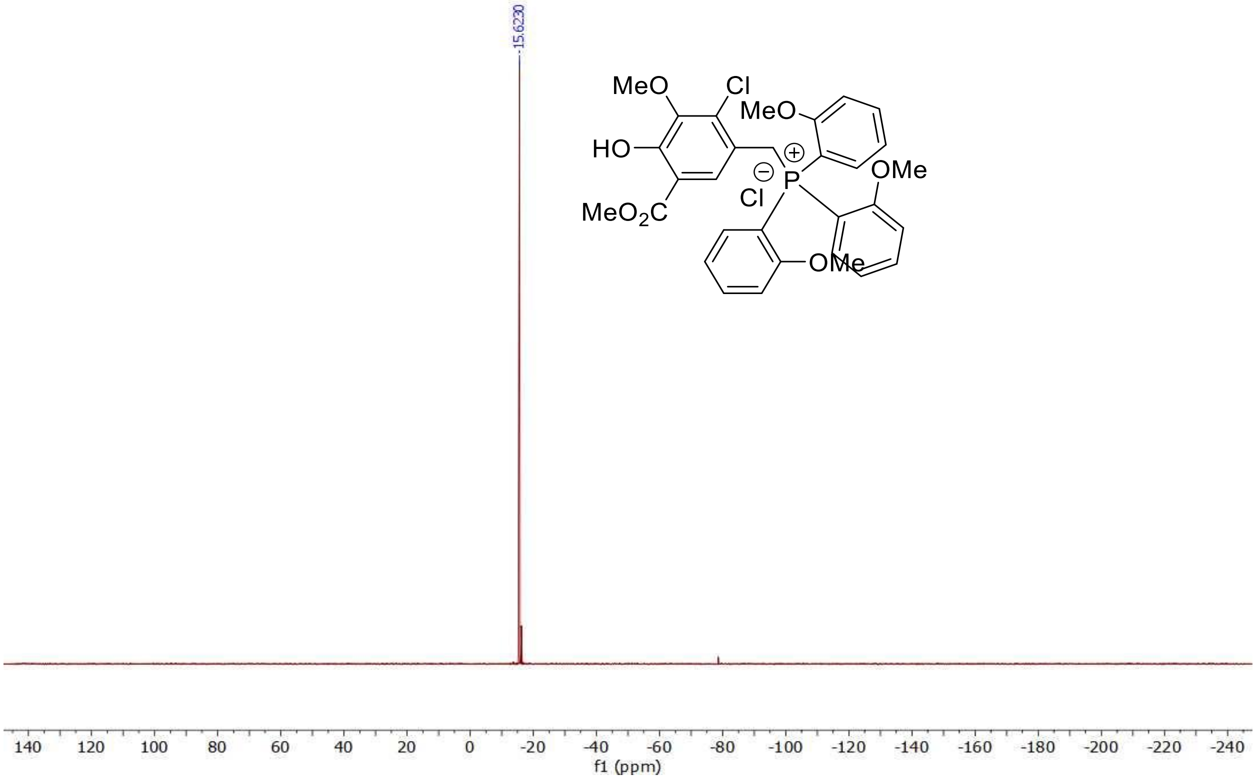


$^{1}$H NMR (400 MHz, $CDCl_3$), $^{13}$C NMR (100 MHz, $CDCl_3$) & $^{31}$P of 2c

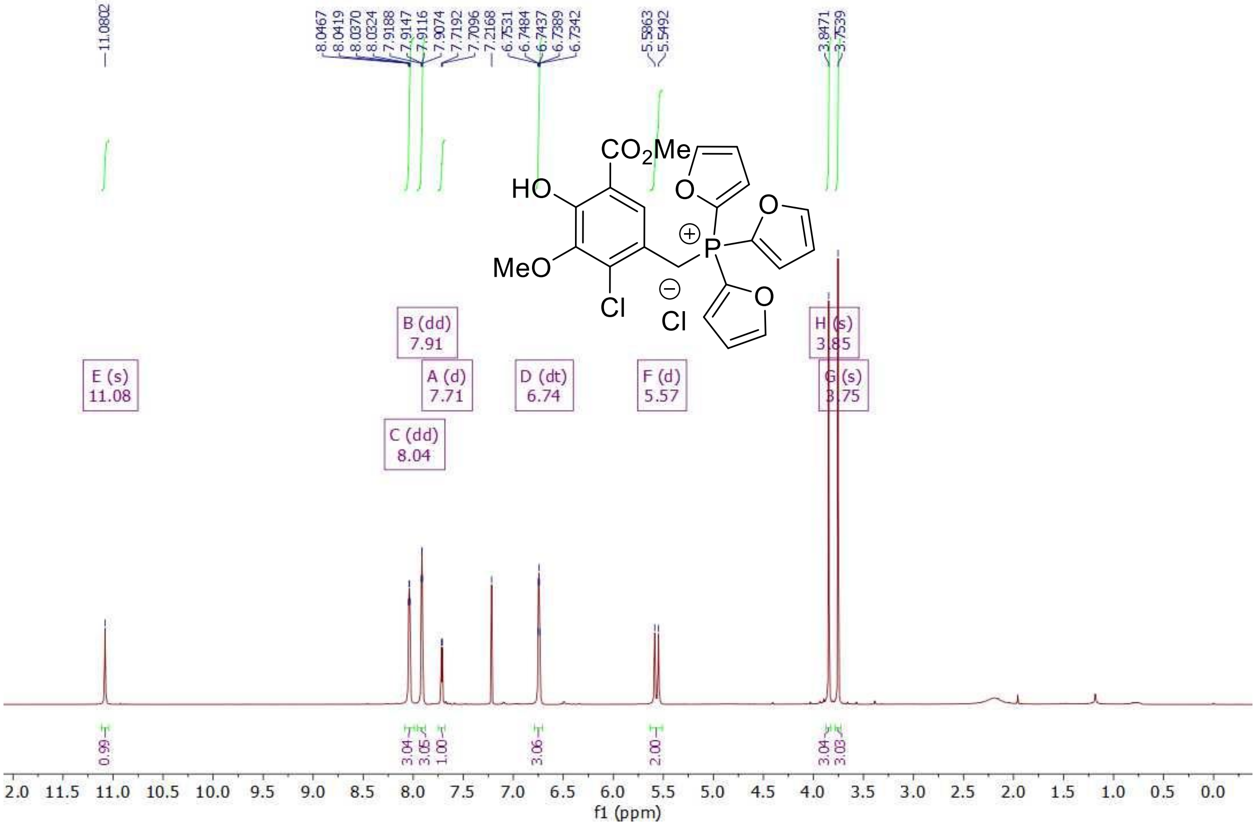

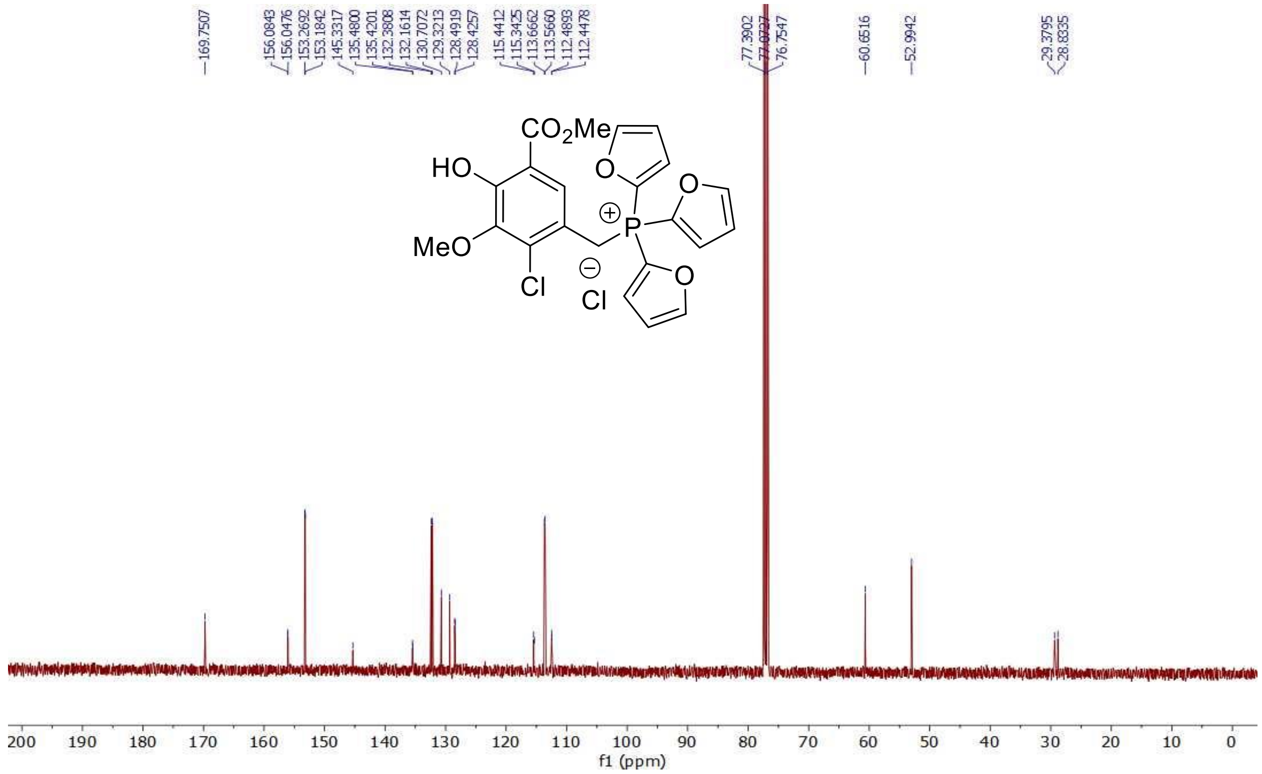
169.7507
156.0843
156.0476
153.2692
153.1842
145.3317
135.4800
135.4201
132.3808
132.1614
130.7072
129.3213
128.4919
128.4257
115.4412
115.3425
113.6662
113.5660
112.4893
112.4478
77.3902
77.0727
76.7547
60.6516
52.9942
29.3795
28.8335
CO2Me
HO
MeO
Cl
Cl
O
P
200 190 180 170 160 150 140 130 120 110 100 90 80 70 60 50 40 30 20 10 0
f1 (ppm)

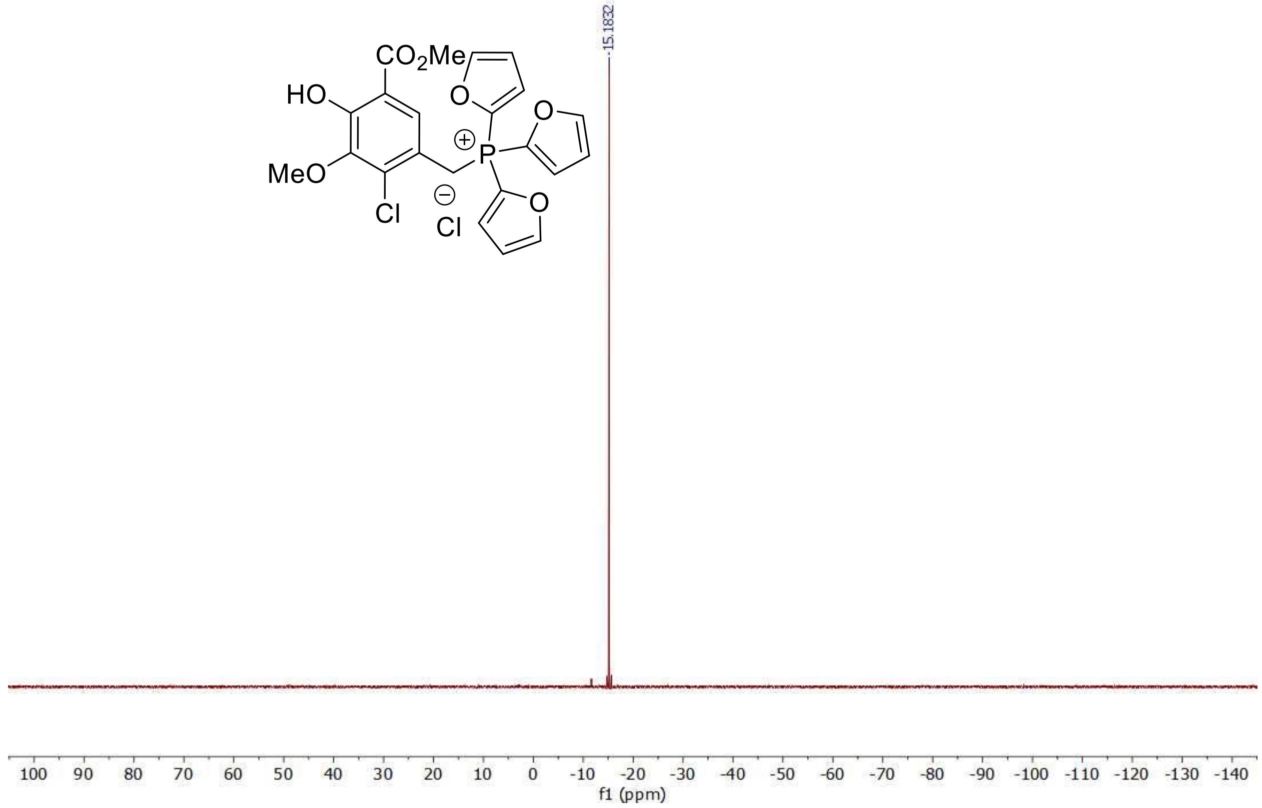
-15.1832
CO2Me
HO
MeO
Cl
Cl
O
P
100 90 80 70 60 50 40 30 20 10 0 -10 -20 -30 -40 -50 -60 -70 -80 -90 -100 -110 -120 -130 -140
f1 (ppm)

**$^1$H NMR (400 MHz, CDCl$_3$), $^{13}$C NMR (100 MHz, CDCl$_3$) and $^{31}$P of 2d.**

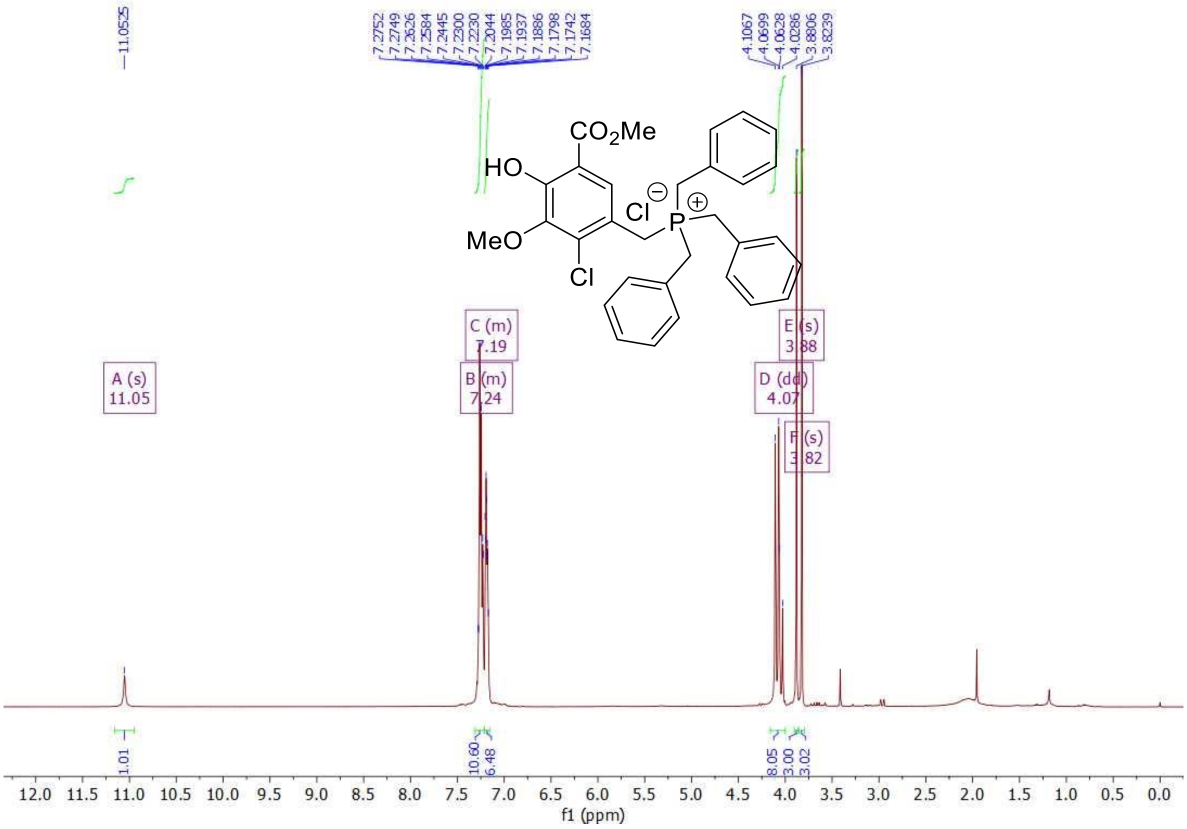


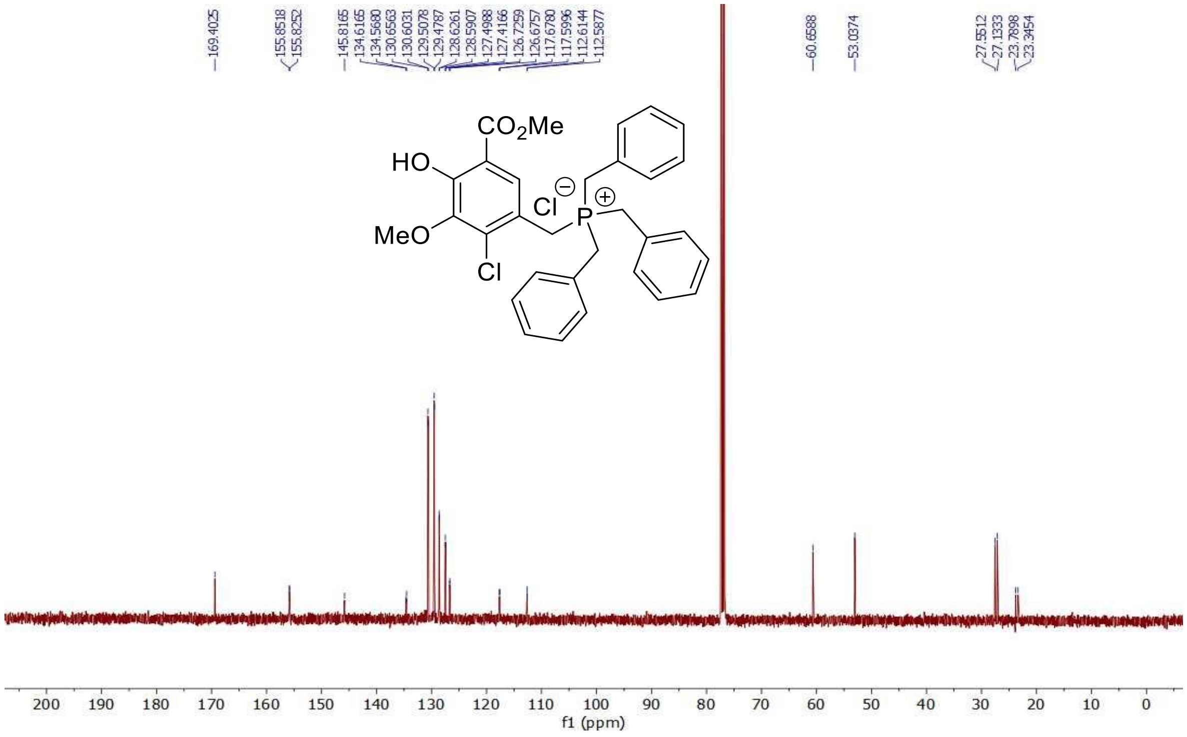

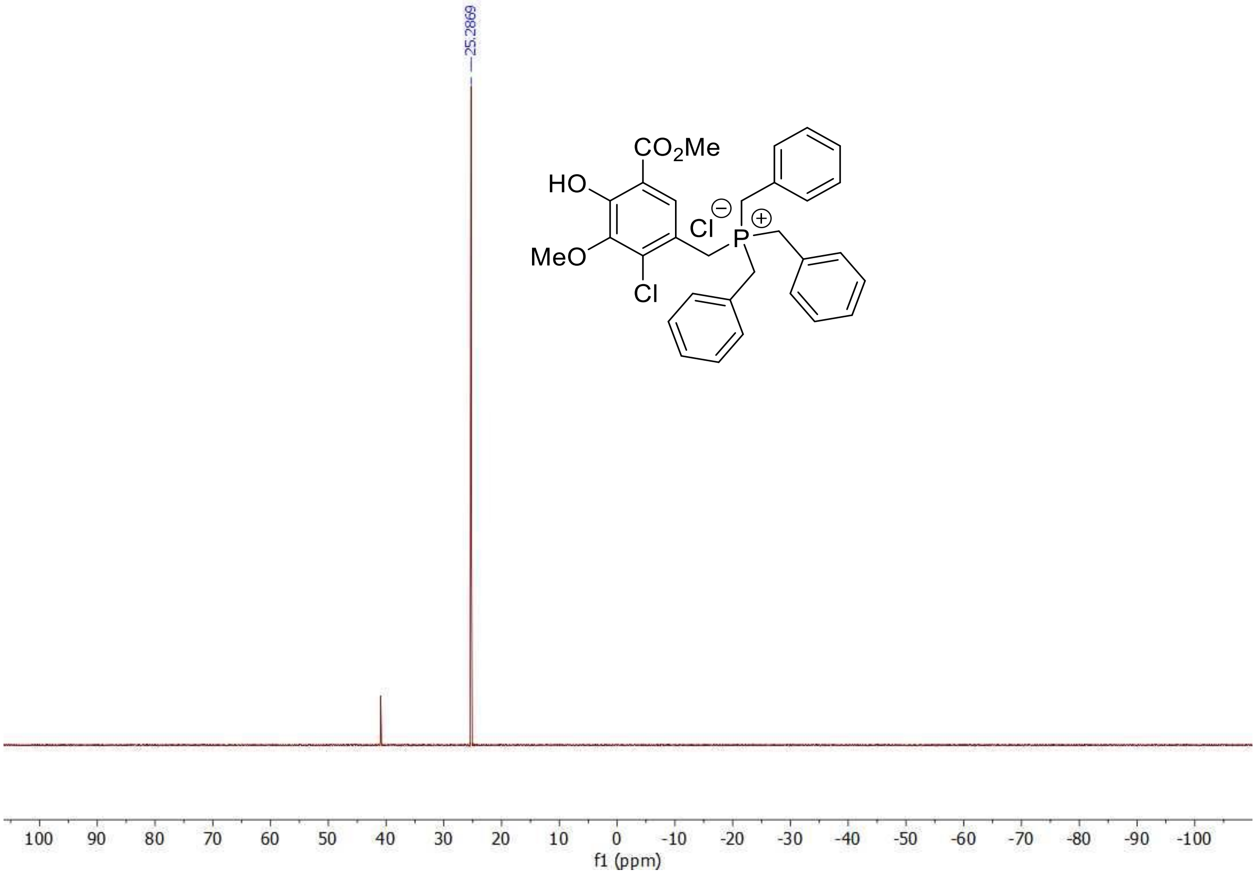


**$^{1}$H NMR (400 MHz, CDCl3), $^{13}$C NMR (100 MHz, CDCl3) & $^{31}$P of 2e**

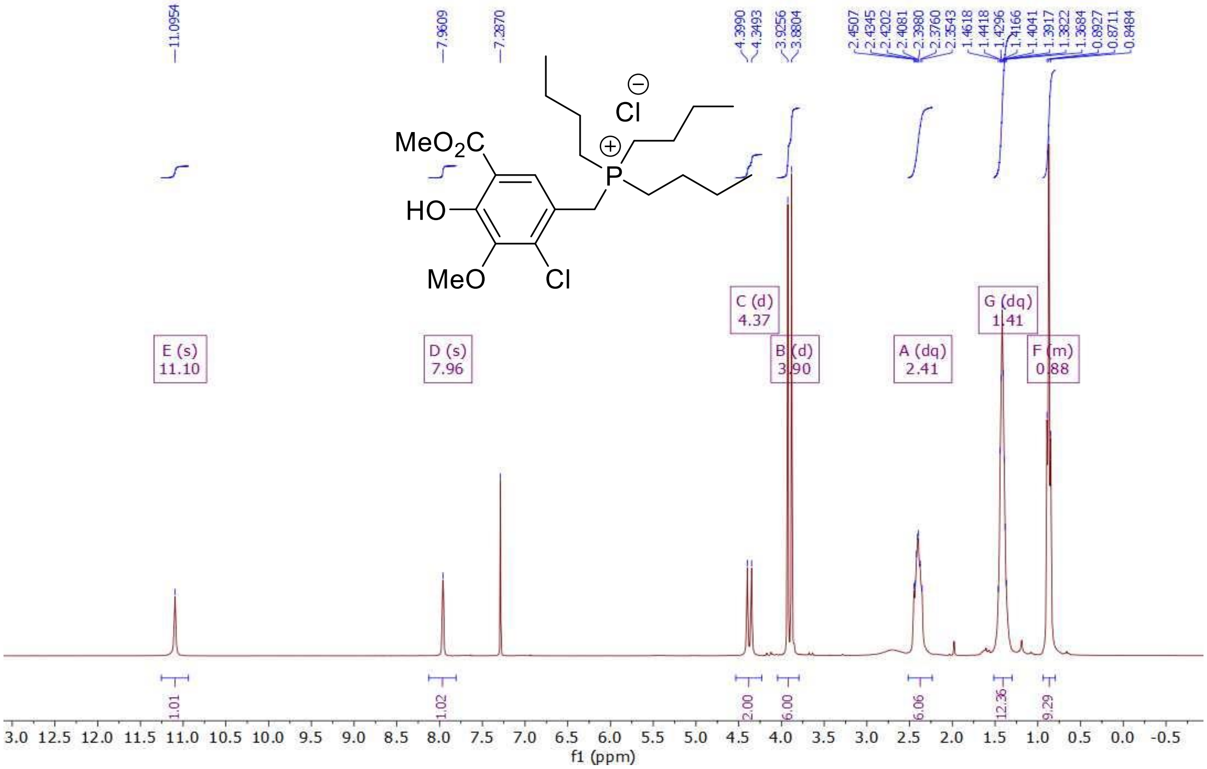

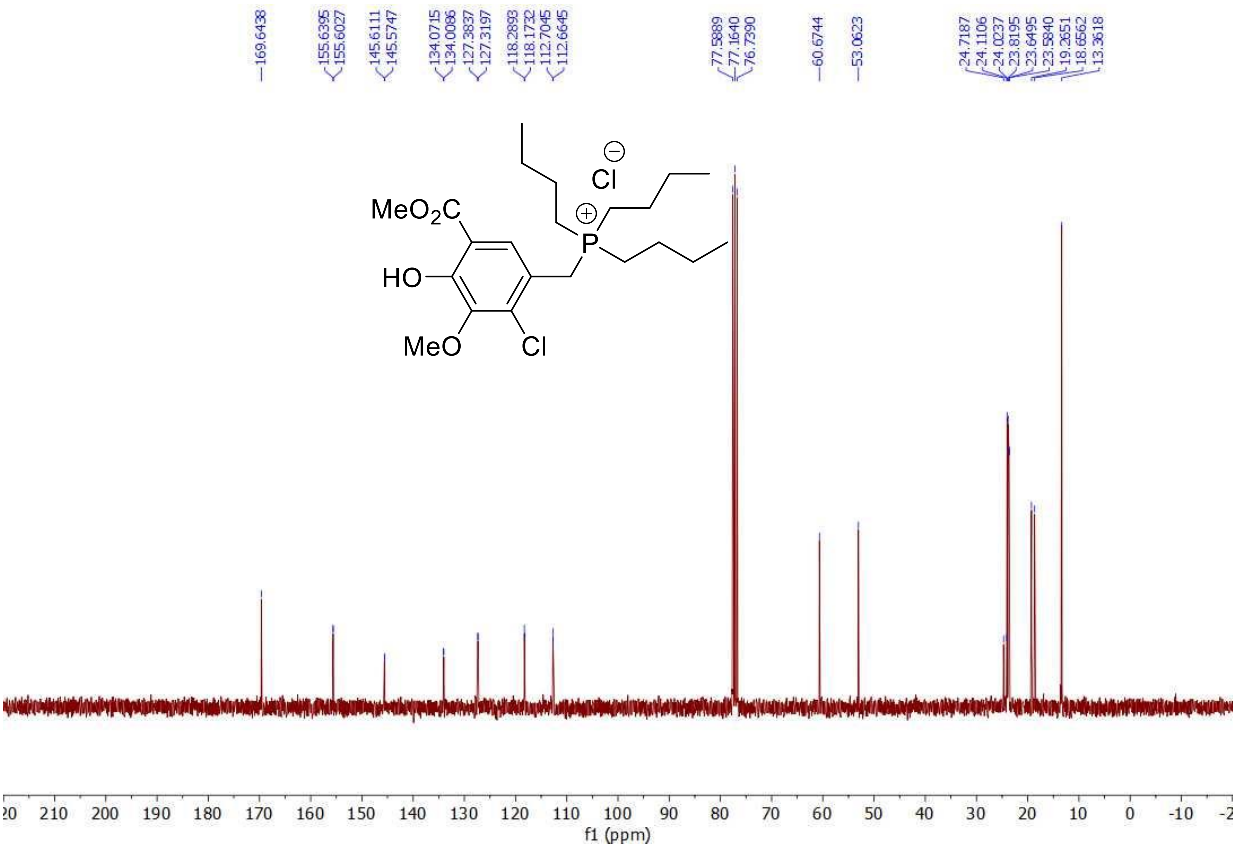
169.6438
155.6395
155.6027
145.6111
145.5747
134.0715
134.0086
127.3837
127.3197
118.2893
118.1732
112.7045
112.6645
77.5889
77.1640
76.7390
60.6744
53.0623
24.7187
24.1106
24.0237
23.8195
23.6495
23.5840
19.3651
18.6562
13.3618
MeO2C
HO
MeO
Cl
P
Cl
f1 (ppm)

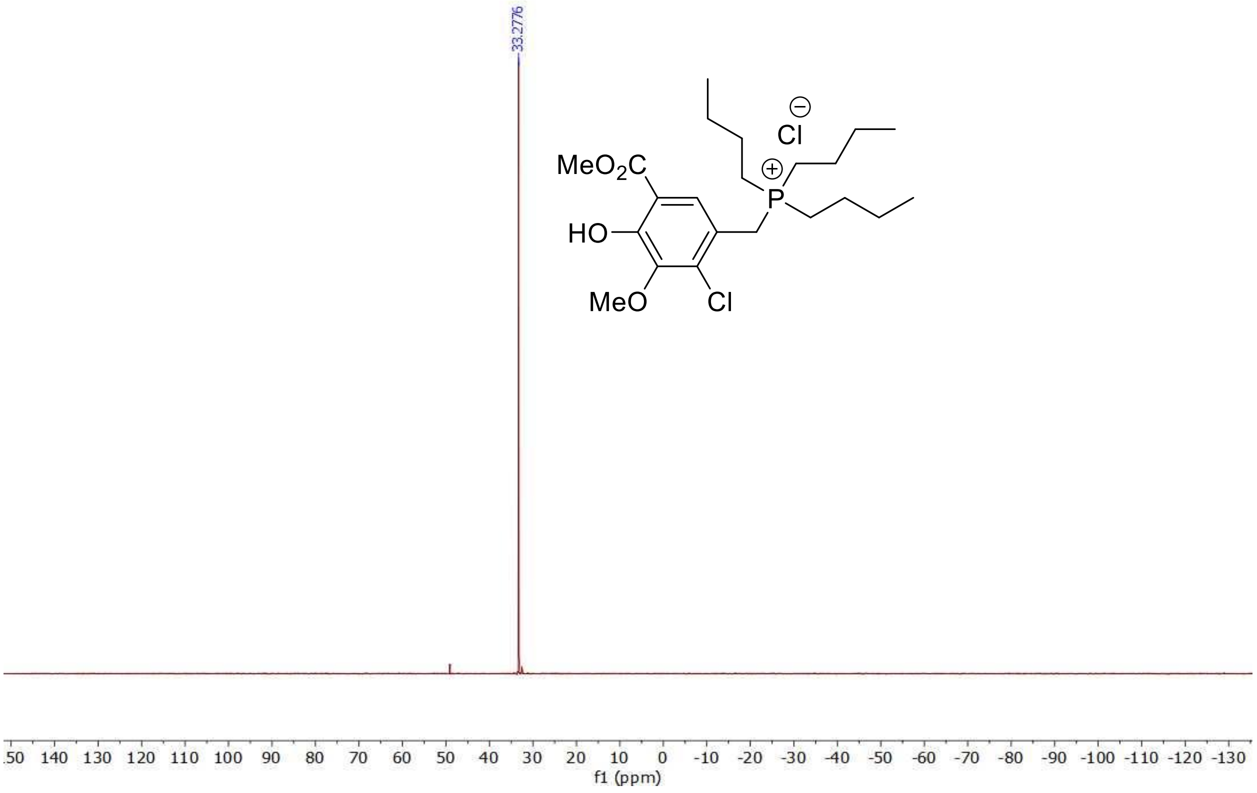
33.2776
MeO2C
HO
MeO
Cl
P
Cl
f1 (ppm)

**$^{1}$H NMR (400 MHz, CDCl3), $^{13}$C NMR (100 MHz, CDCl3) and $^{31}$P of 2f**

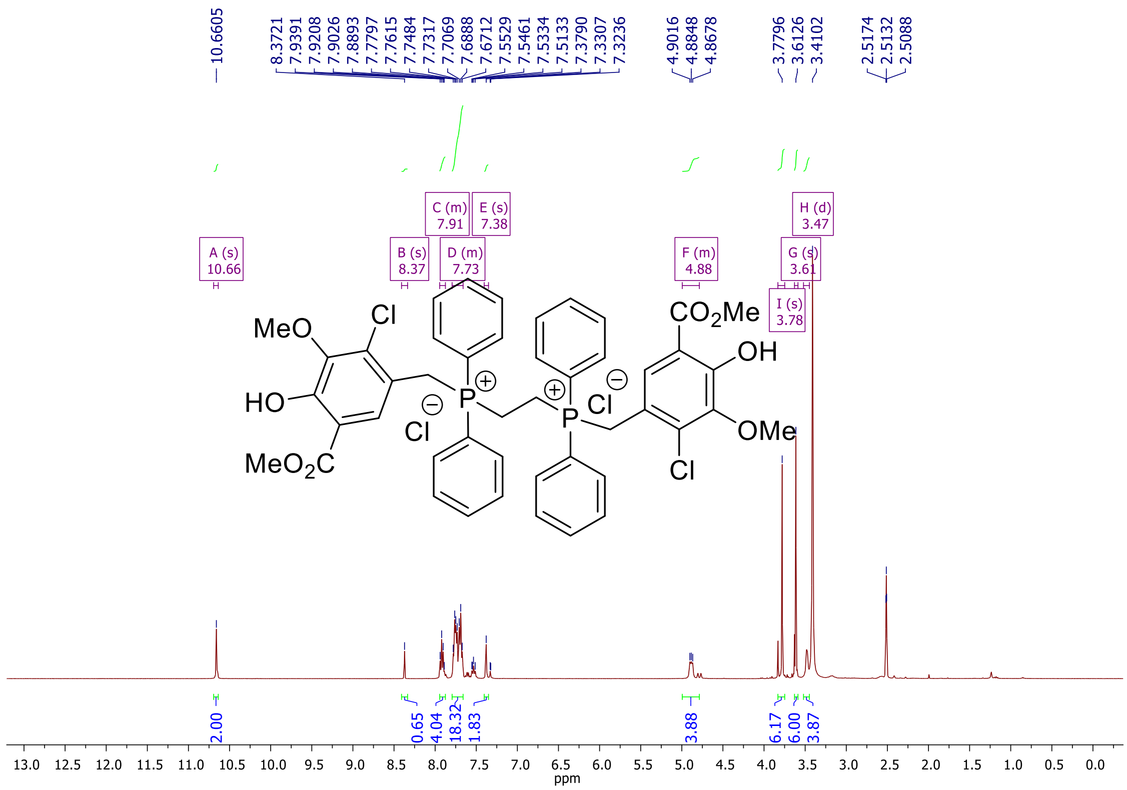

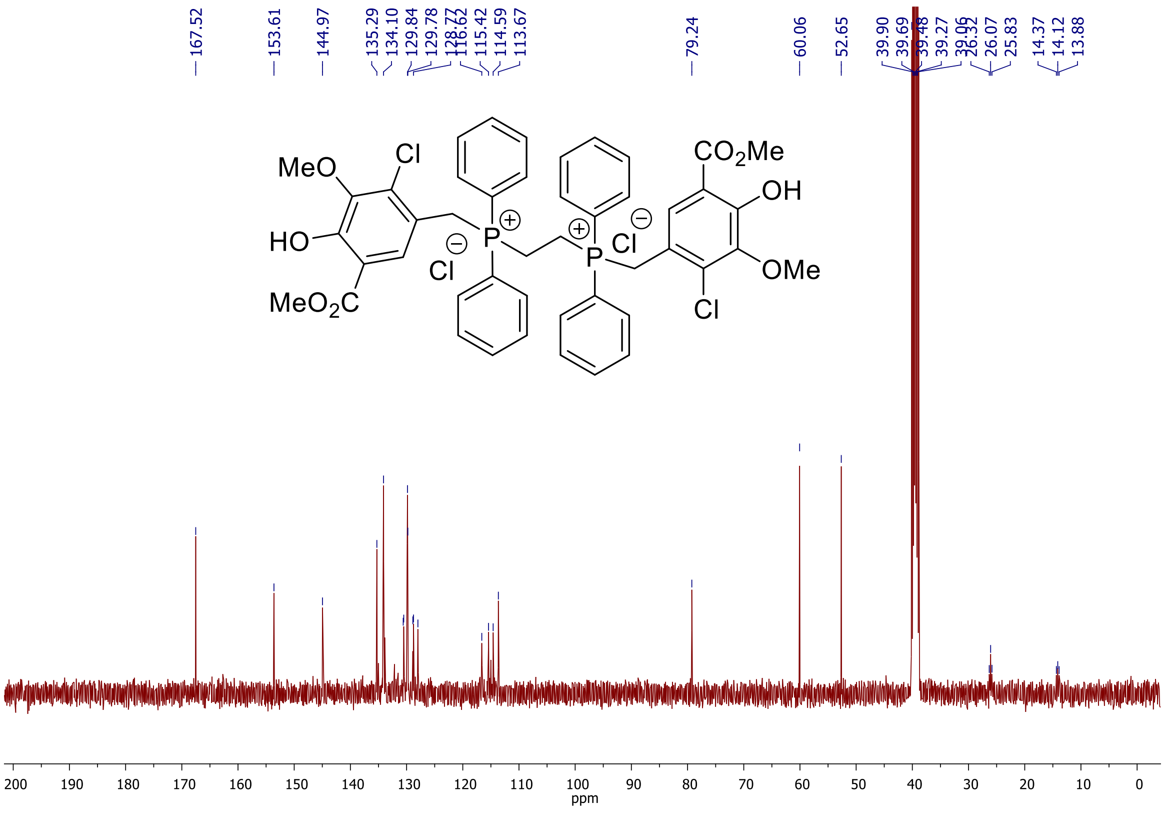

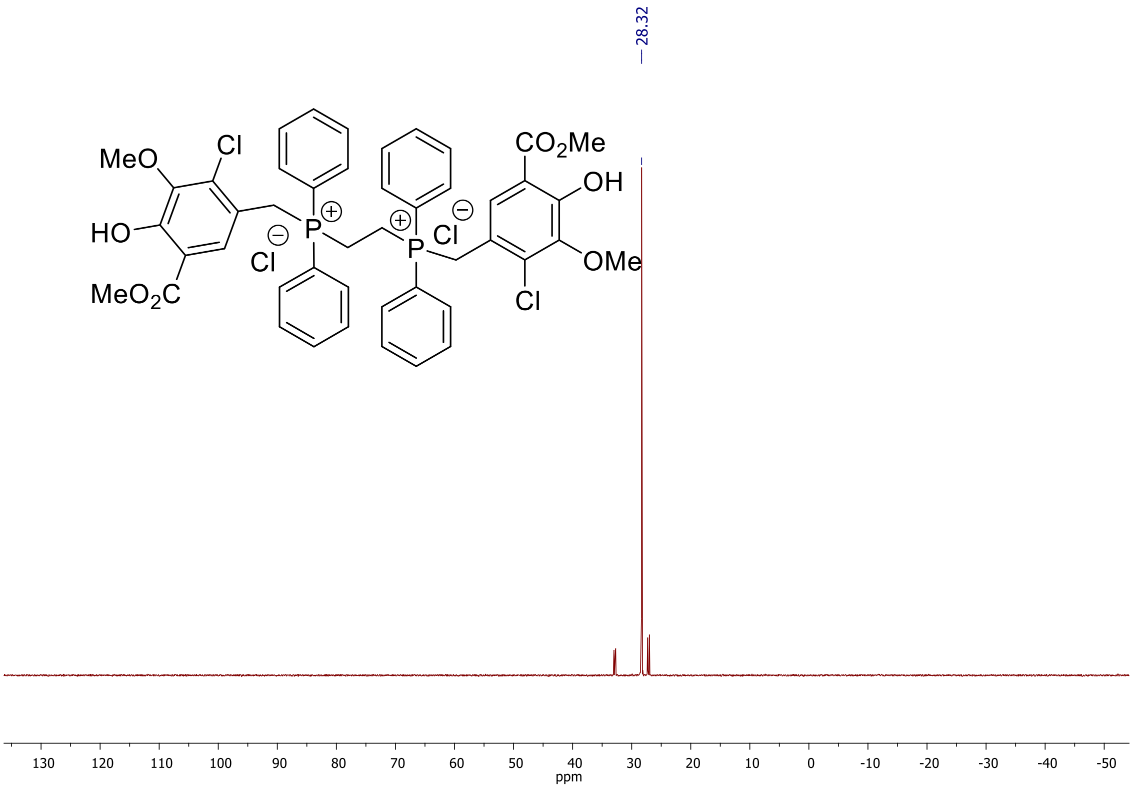


**$^{1}$H NMR (400 MHz, CDCl$_3$), $^{13}$C NMR (100 MHz, CDCl$_3$) & $^{31}$P (161 MHz, CDCl$_3$) of 2g**

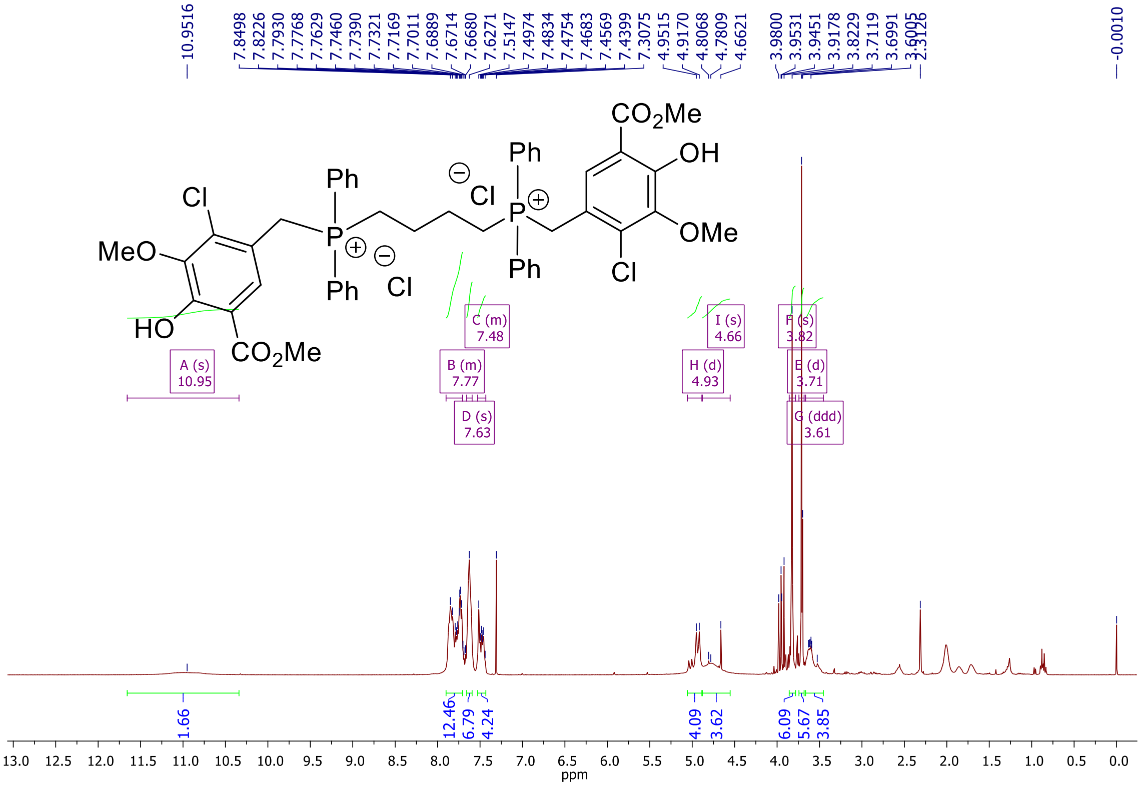

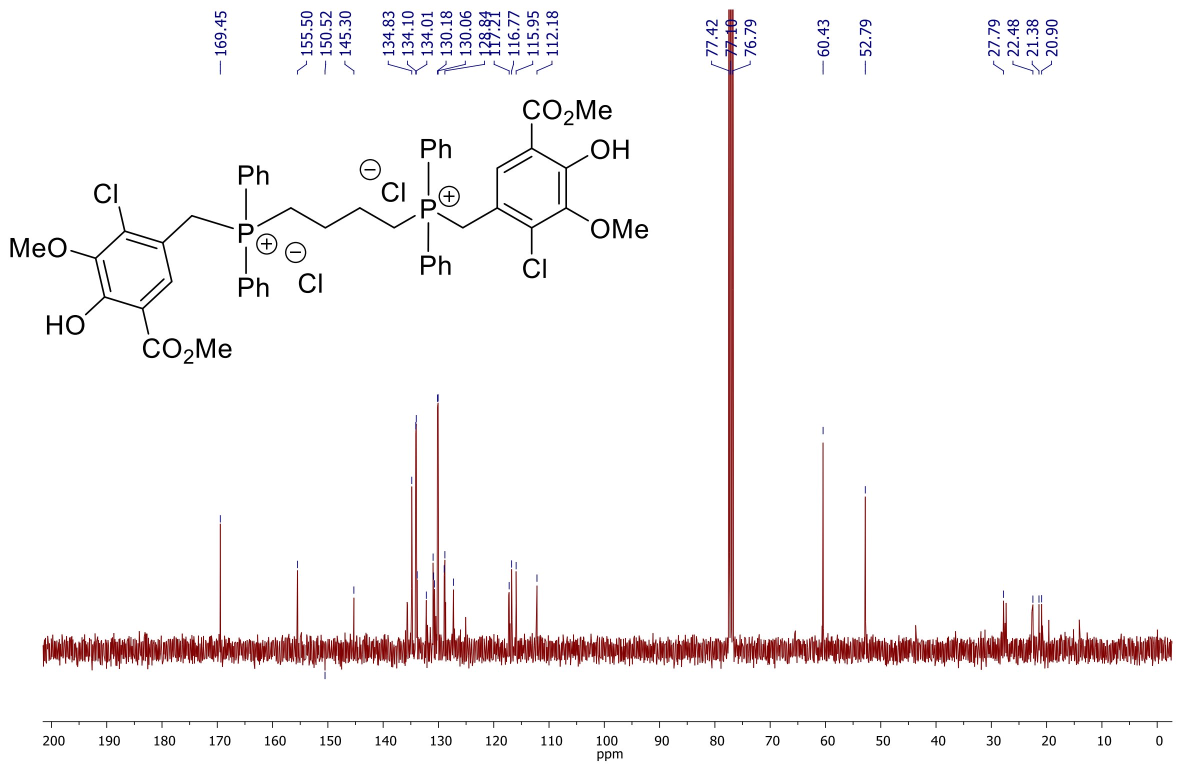

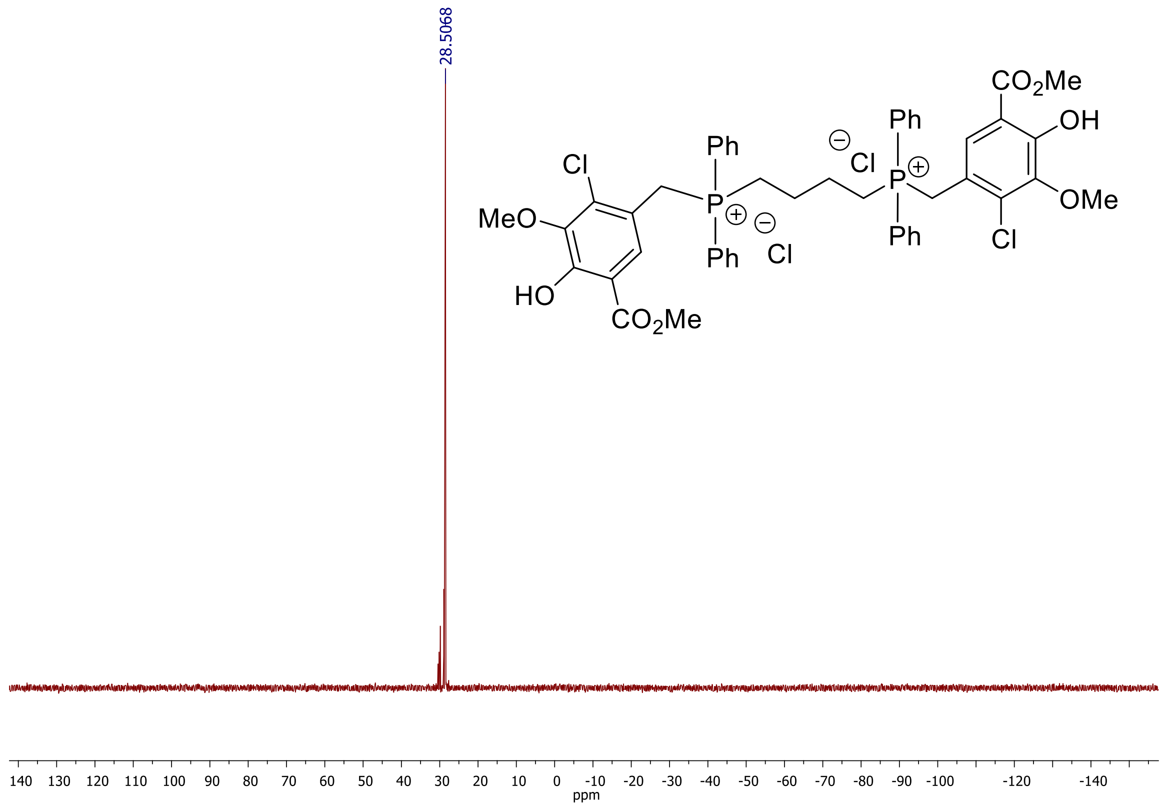

**$^1$H NMR (400 MHz, $CDCl_3$) of 3ab crude mixture after a quick $SIO_2$ filtration**

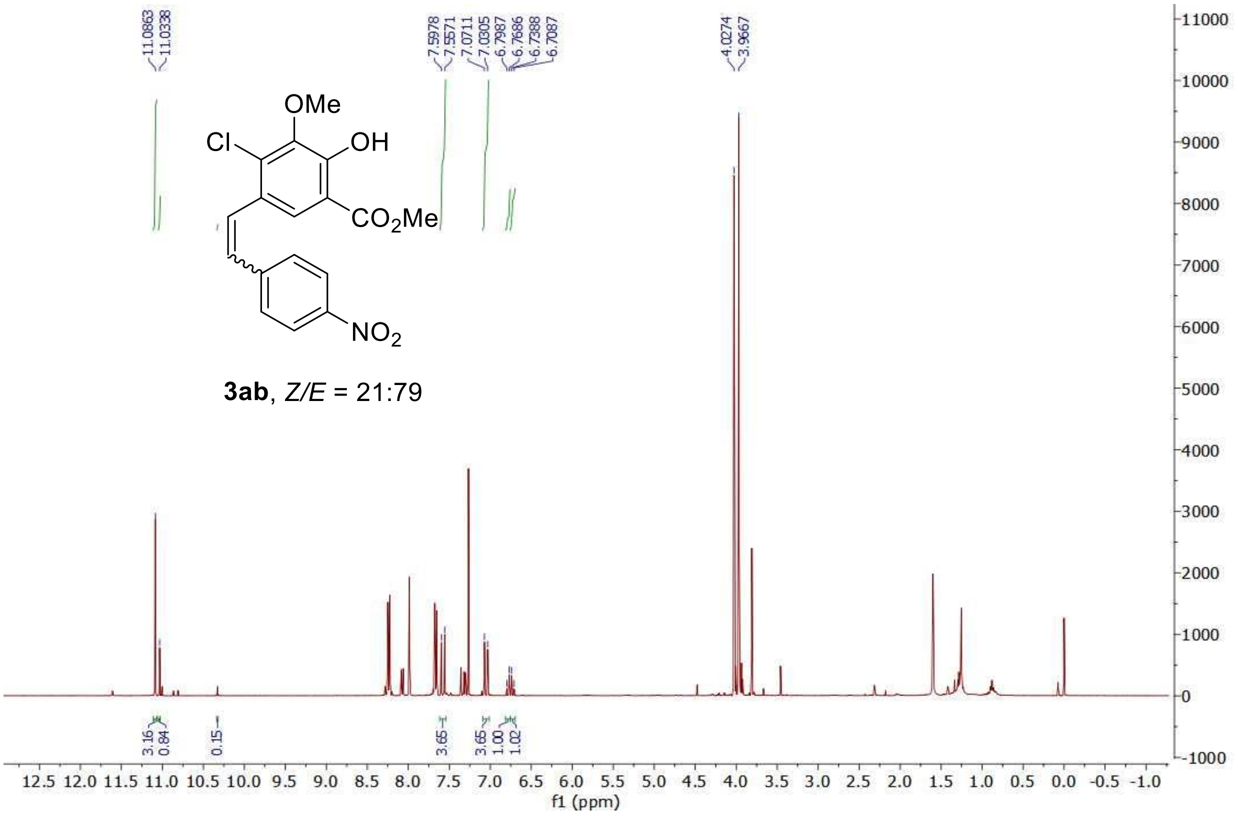

## $^{1}H$ NMR (400 MHz, $CDCl_3$) and $^{13}C$ NMR (100 MHz, $CDCl_3$) of *Z*-isomer of 3a

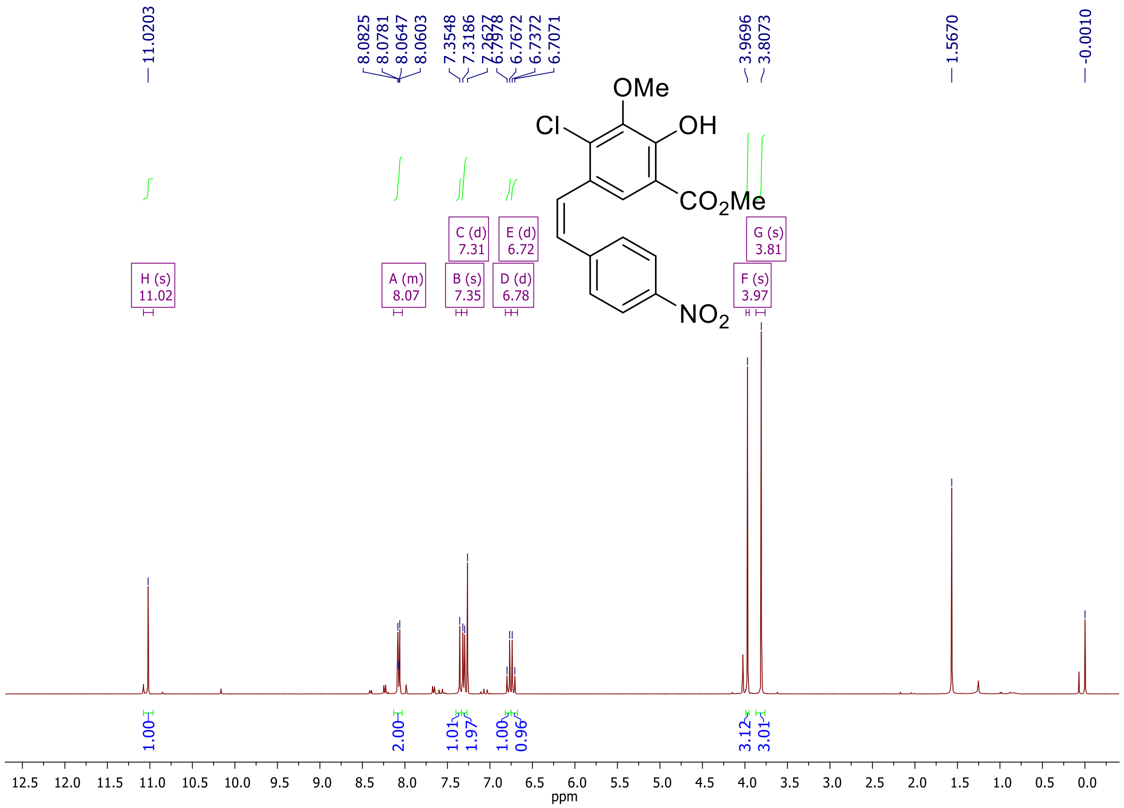

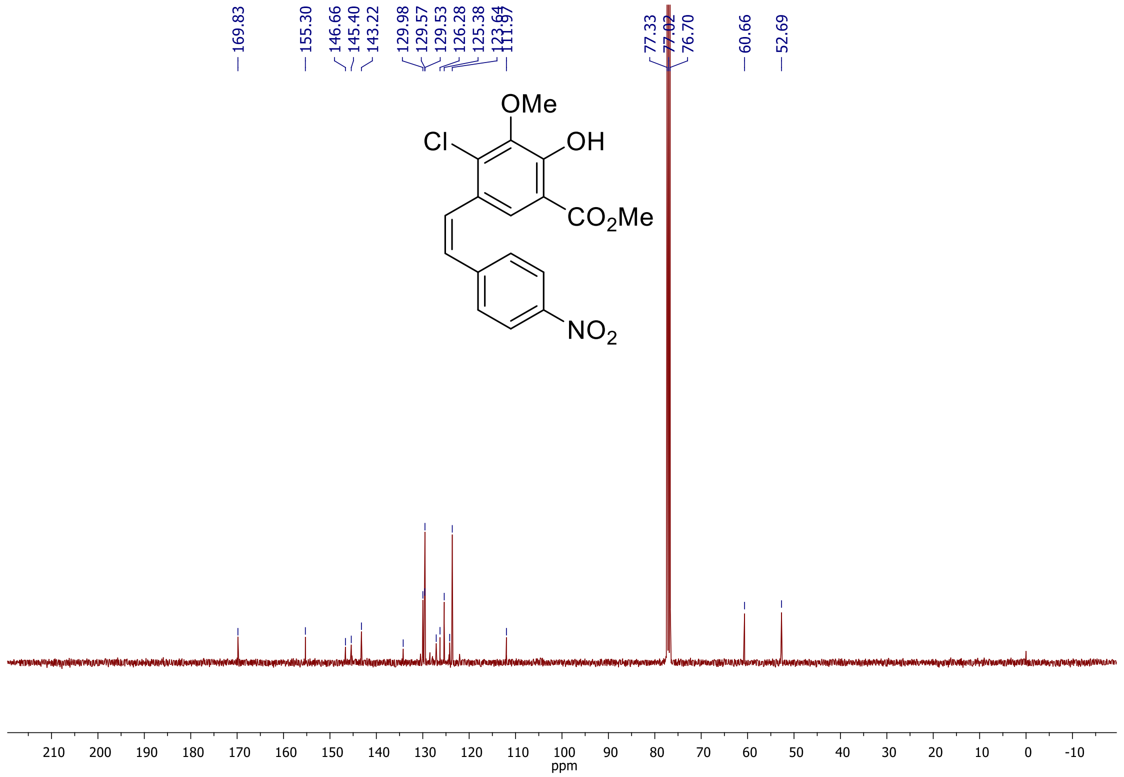

**$^{1}$H NMR (400 MHz, CDCl3) and $^{13}$C NMR (100 MHz, CDCl3) of *E*-isomer of 3b**

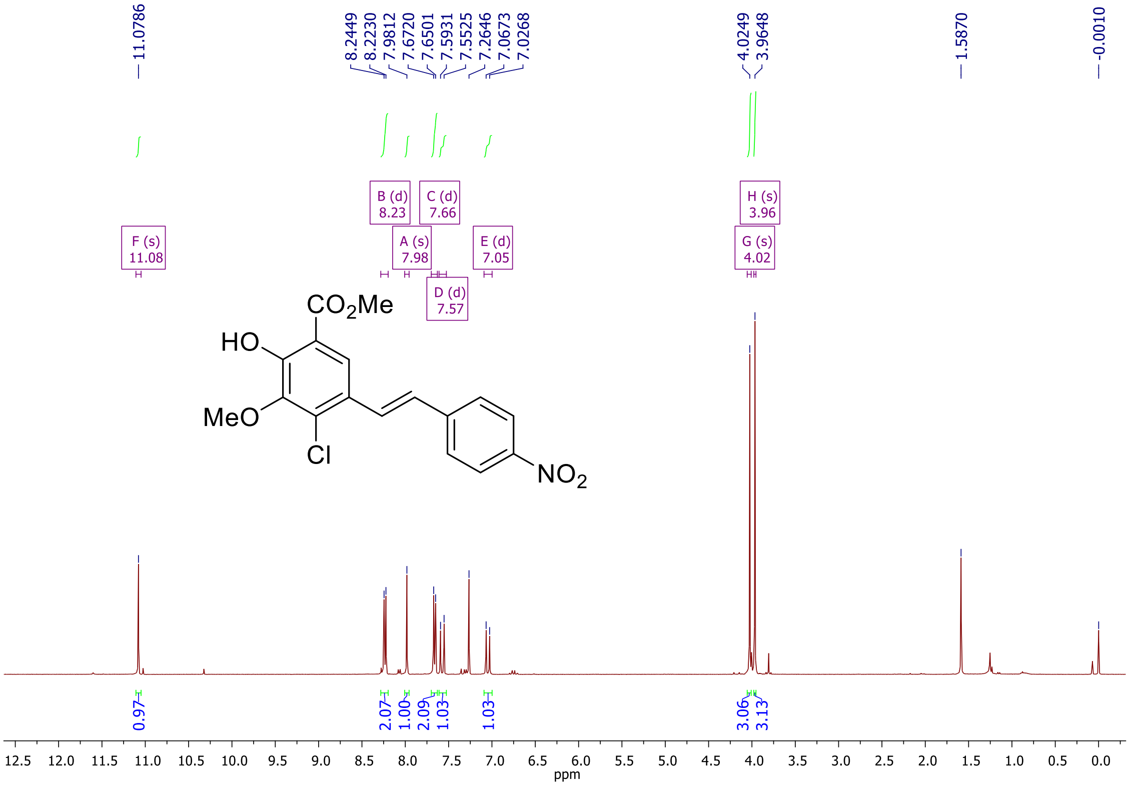

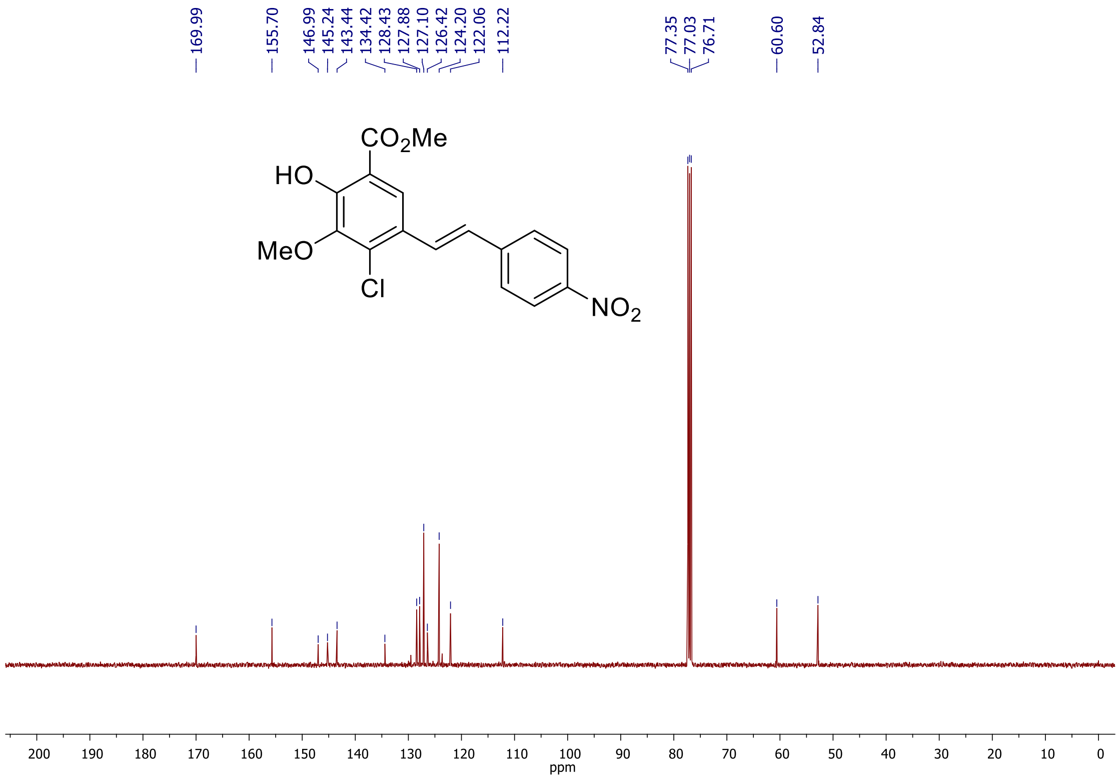

# $^{1}H$ NMR (400 MHz, $CDCl_3$) and $^{13}C$ NMR (100 MHz, $CDCl_3$) of 4a,b

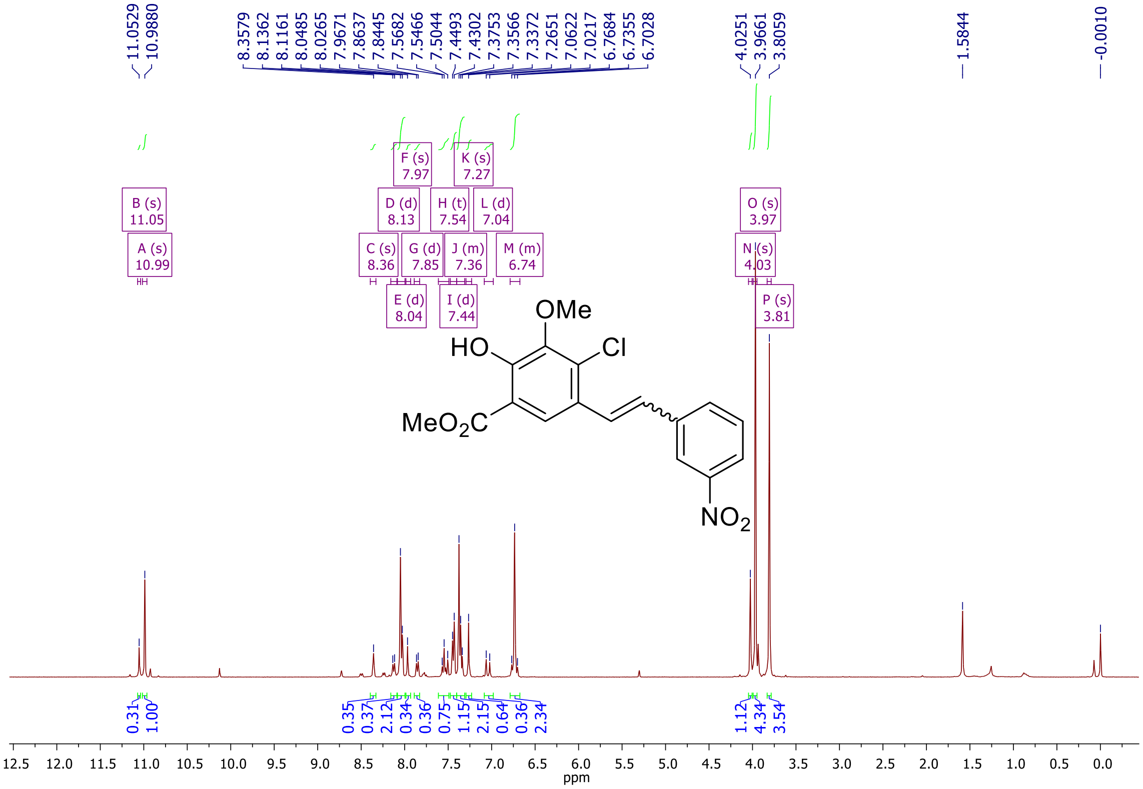

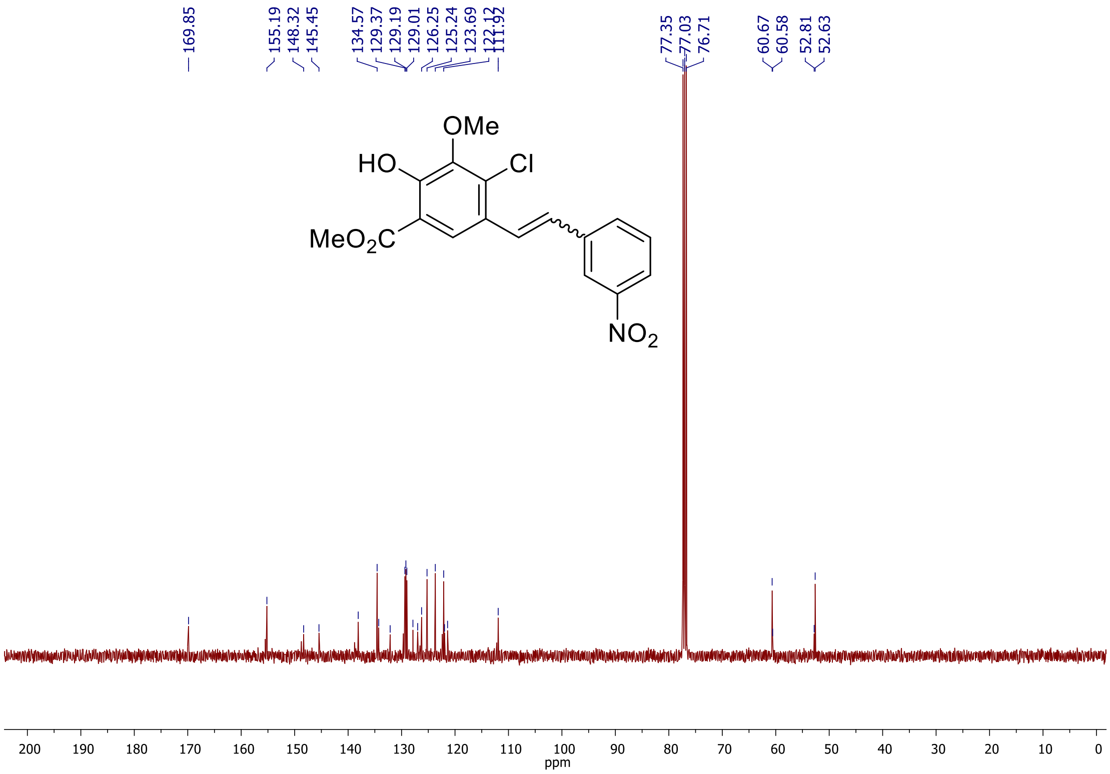

**$^1$H NMR (400 MHz, $CDCl_3$) of 5ab crude mixture after a quick $SIO_2$ filtration**

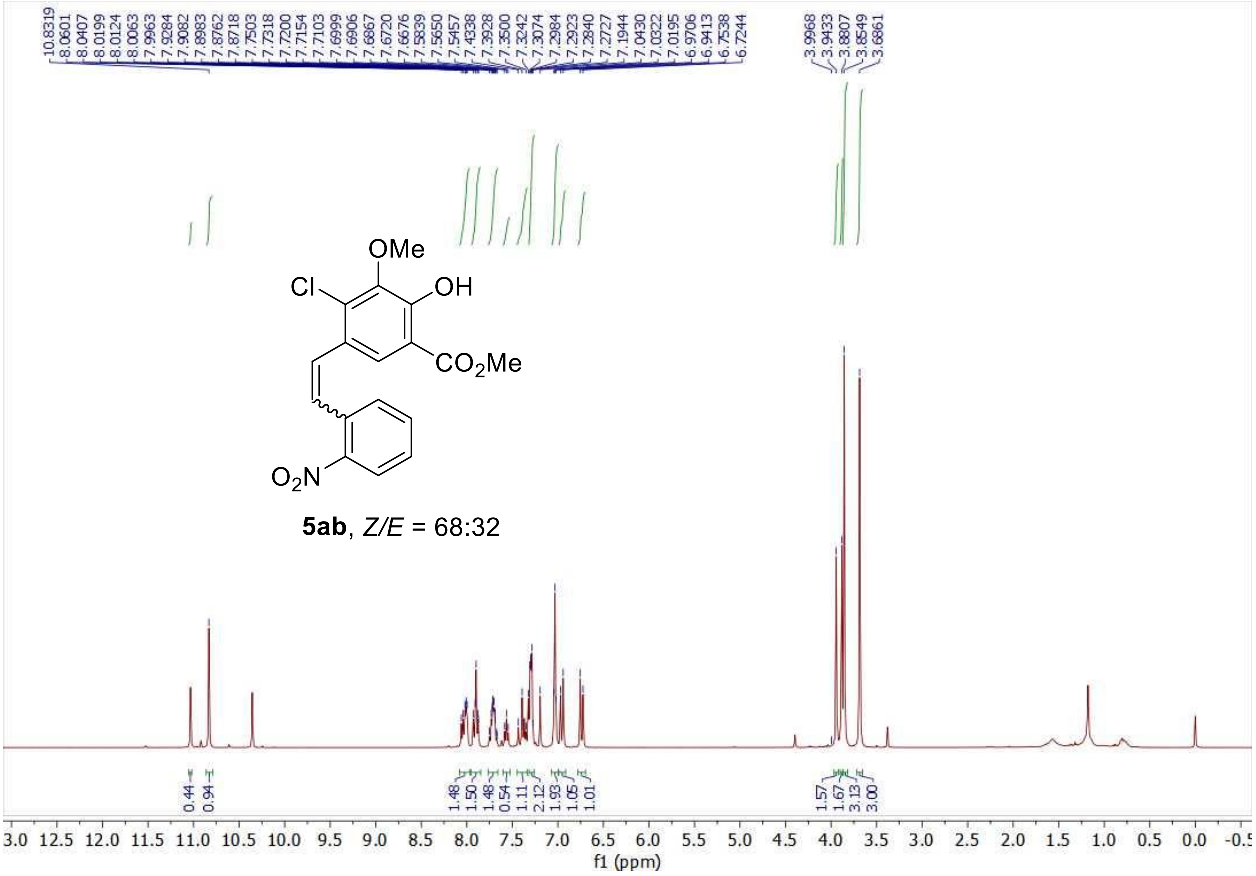

**$^{1}$H NMR (400 MHz, $CDCl_3$) and $^{13}$C NMR (100 MHz, $CDCl_3$) of 5a,b**

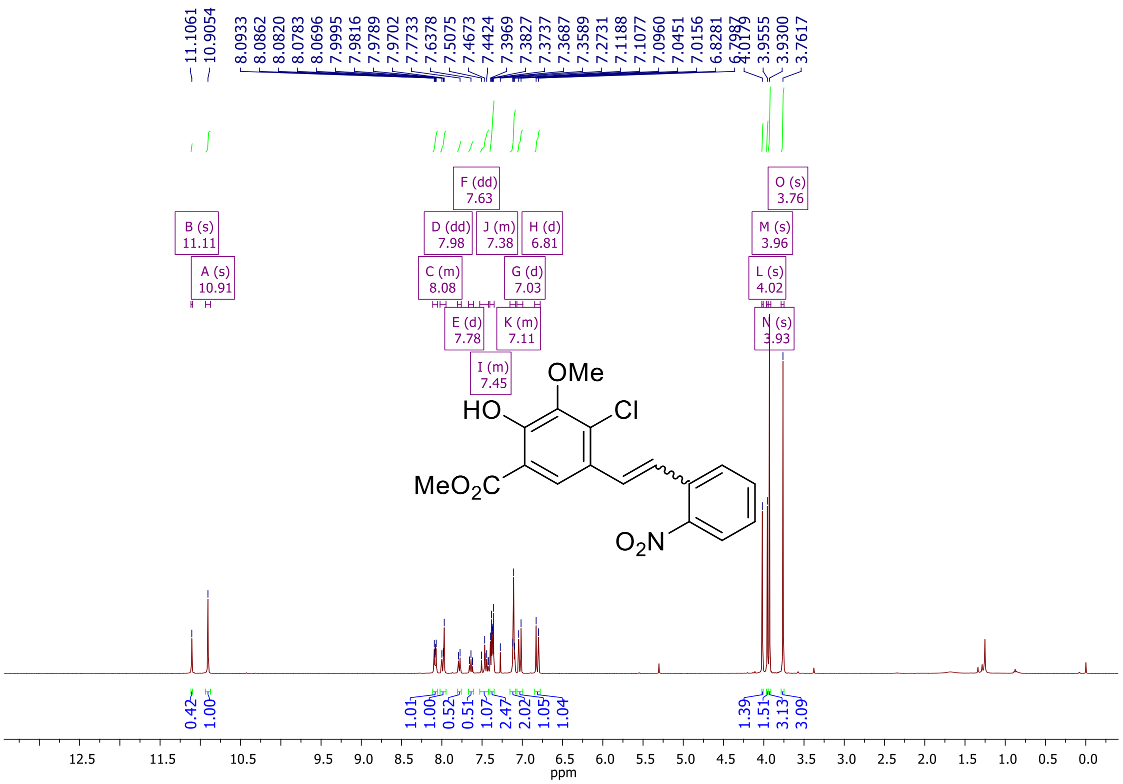


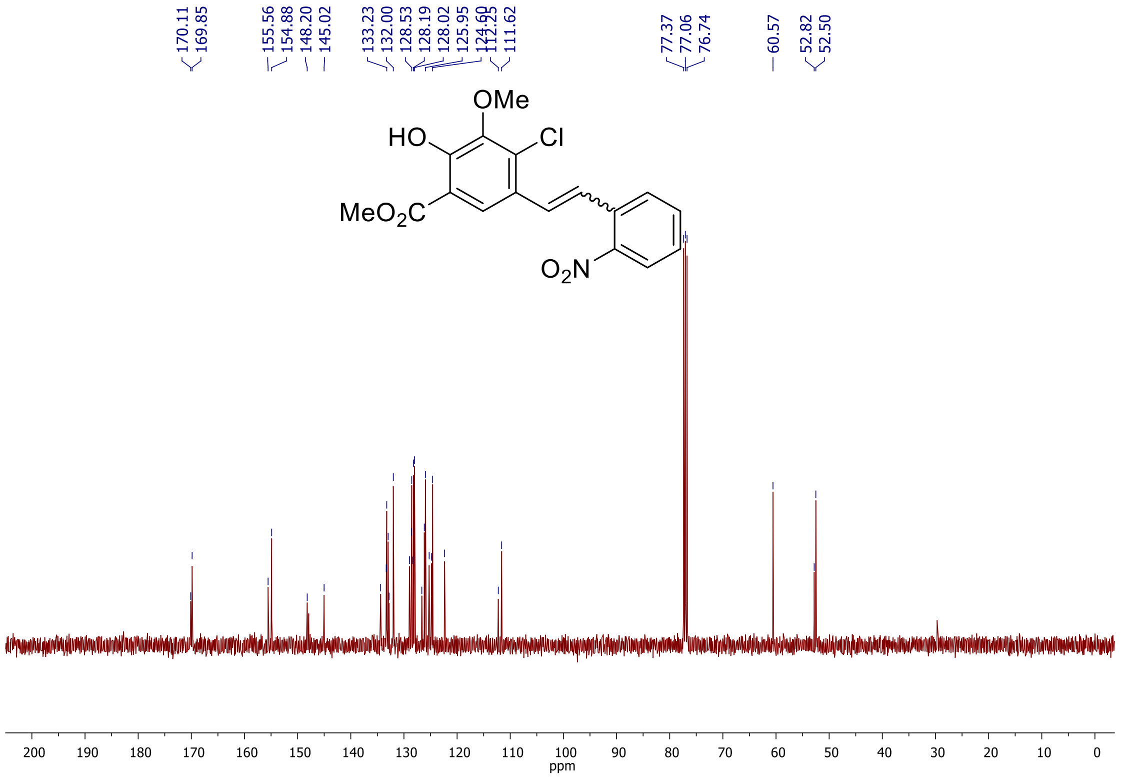

**$^{1}$H NMR (400 MHz, $CDCl_3$) and $^{13}$C NMR (100 MHz, $CDCl_3$) of 6ab after quick $SiO_2$ filtration**

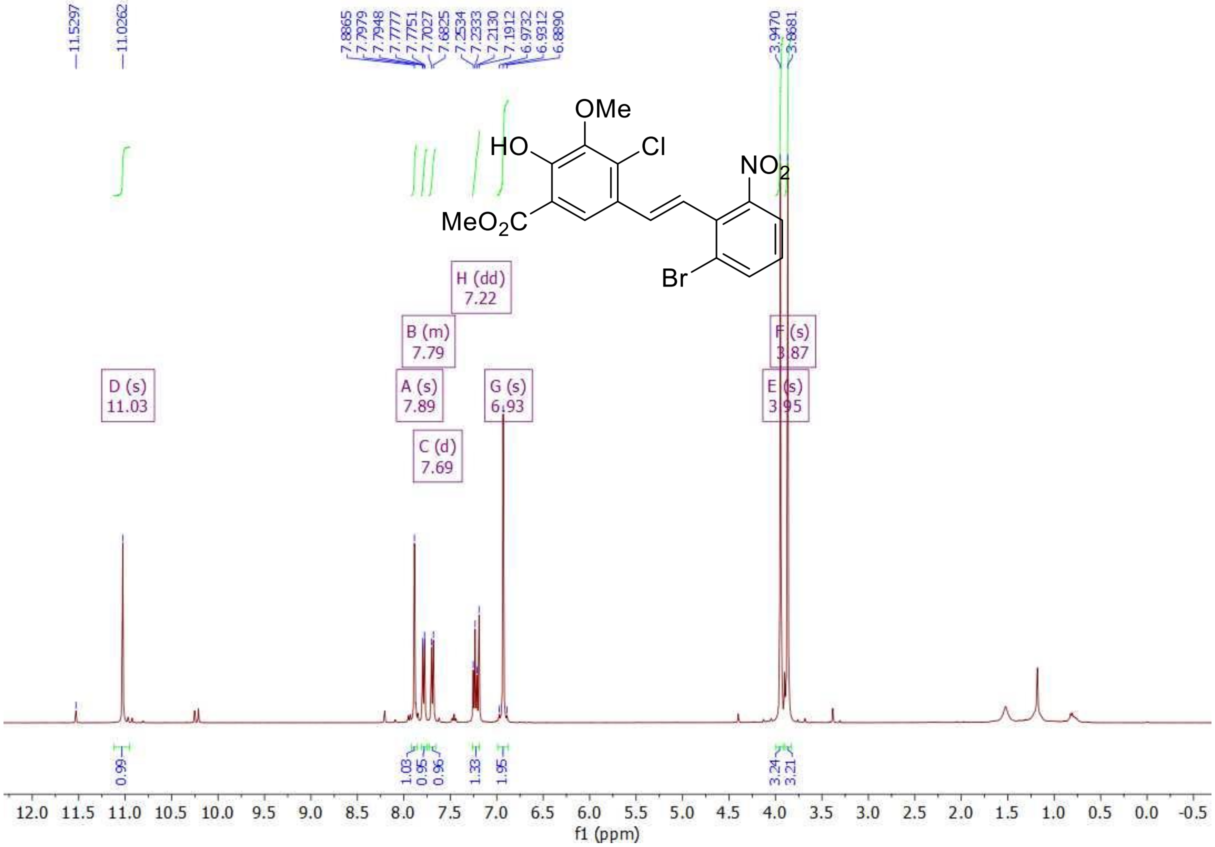


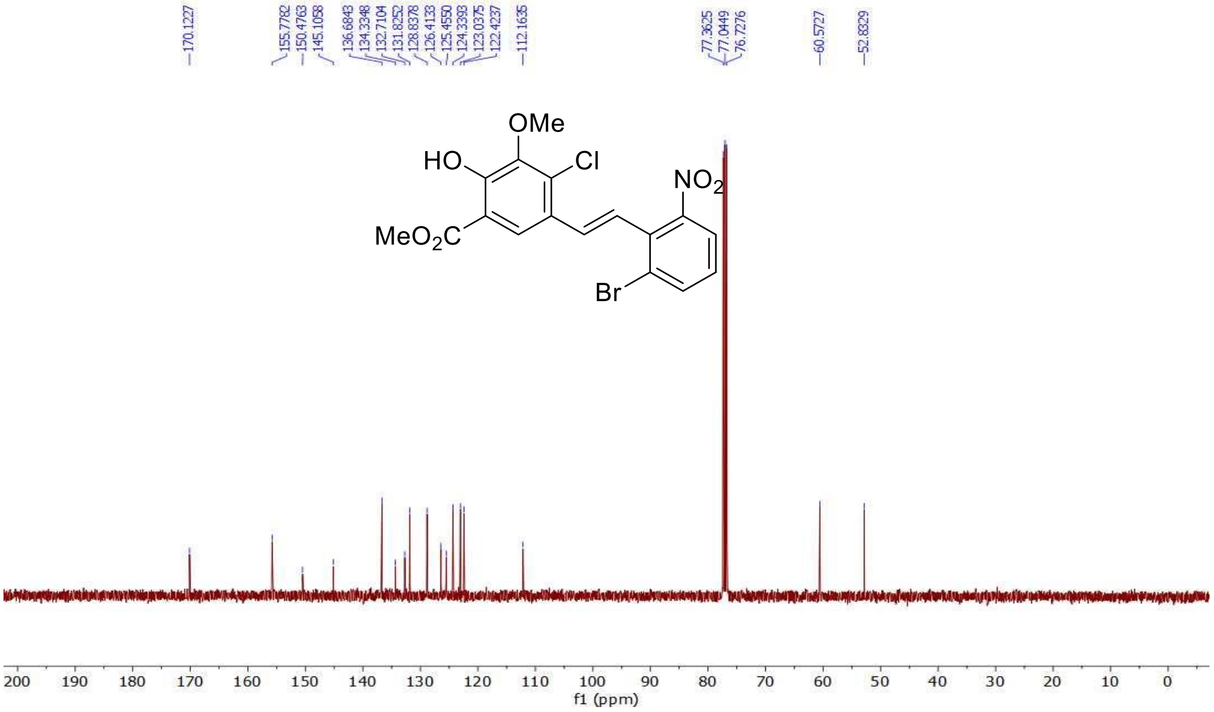

## $^{1}$H NMR (400 MHz, CDCl$_3$) and $^{13}$C NMR (100 MHz, CDCl$_3$) of 7ab

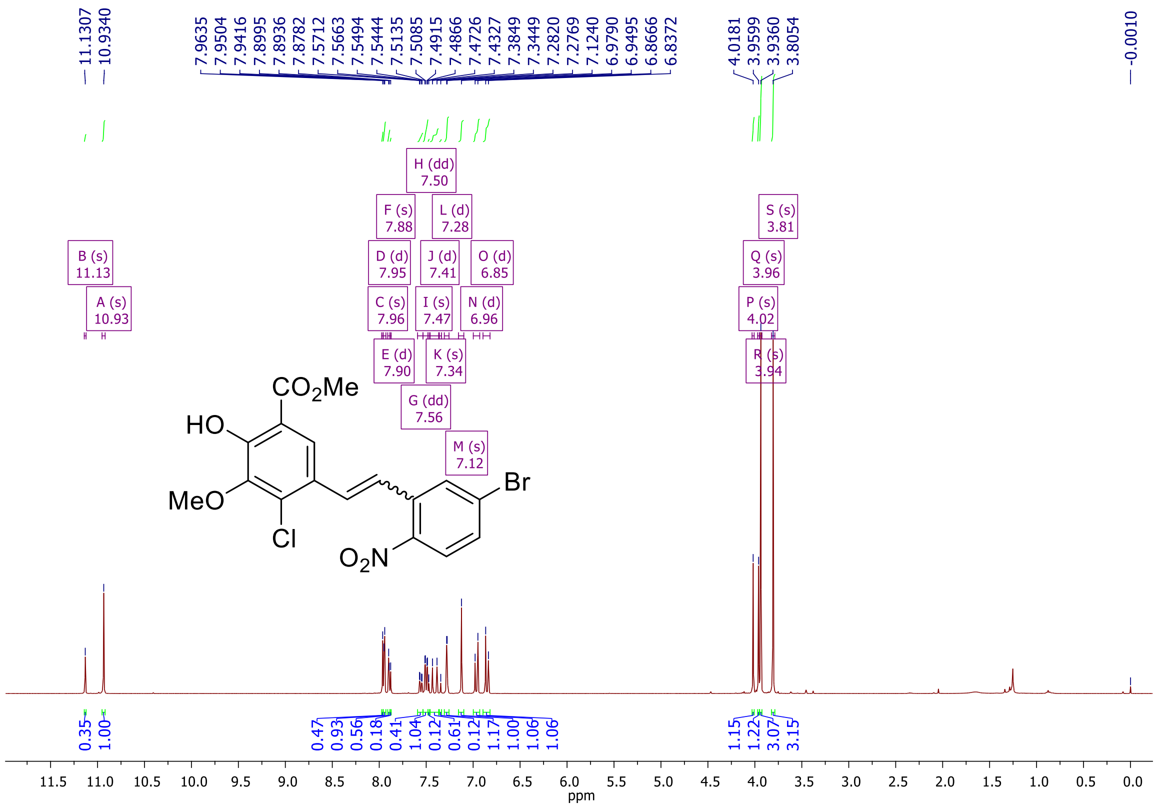


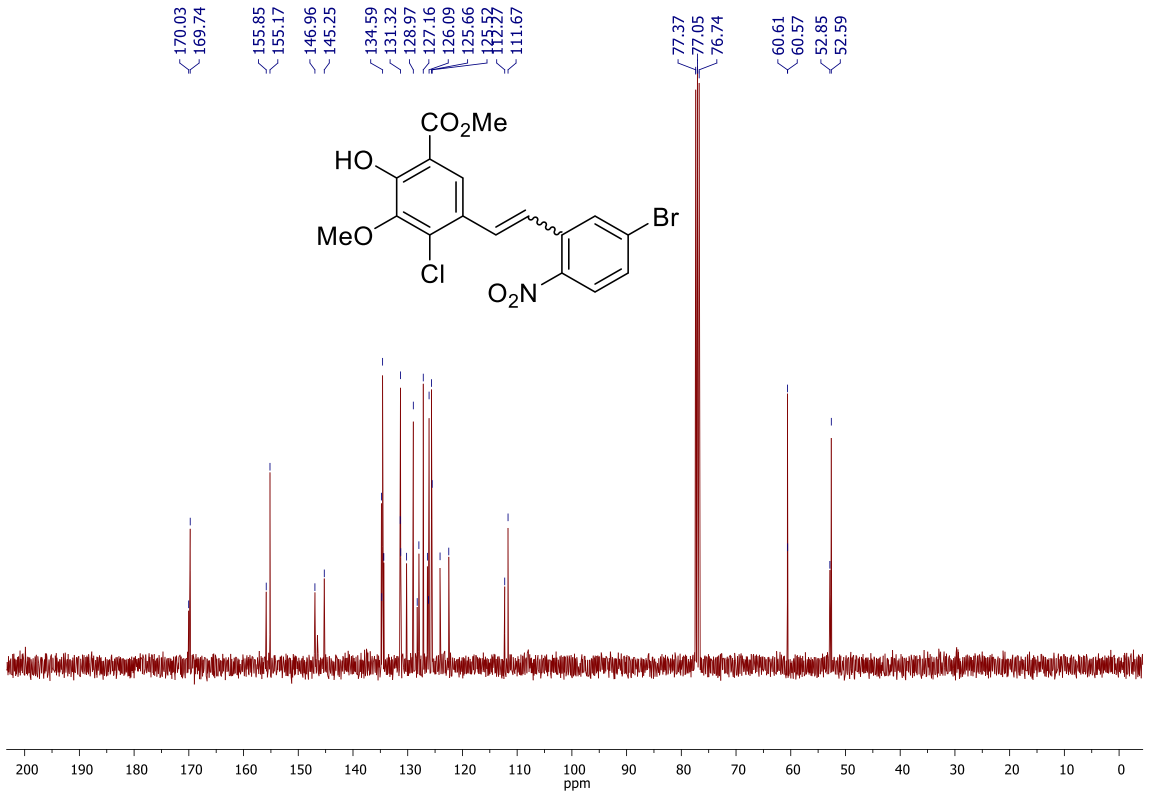

**$^1$H NMR (400 MHz, CDCl$_3$) and $^{13}$C NMR (100 MHz, CDCl$_3$) of 8ab**

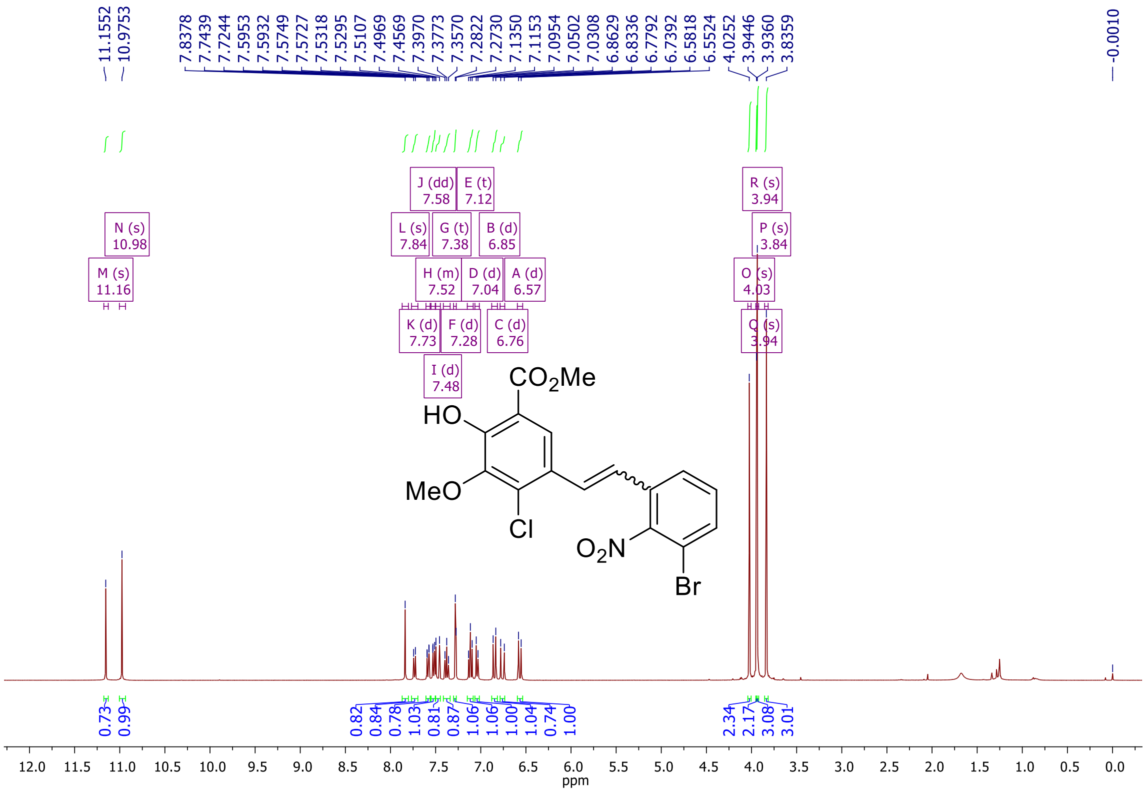

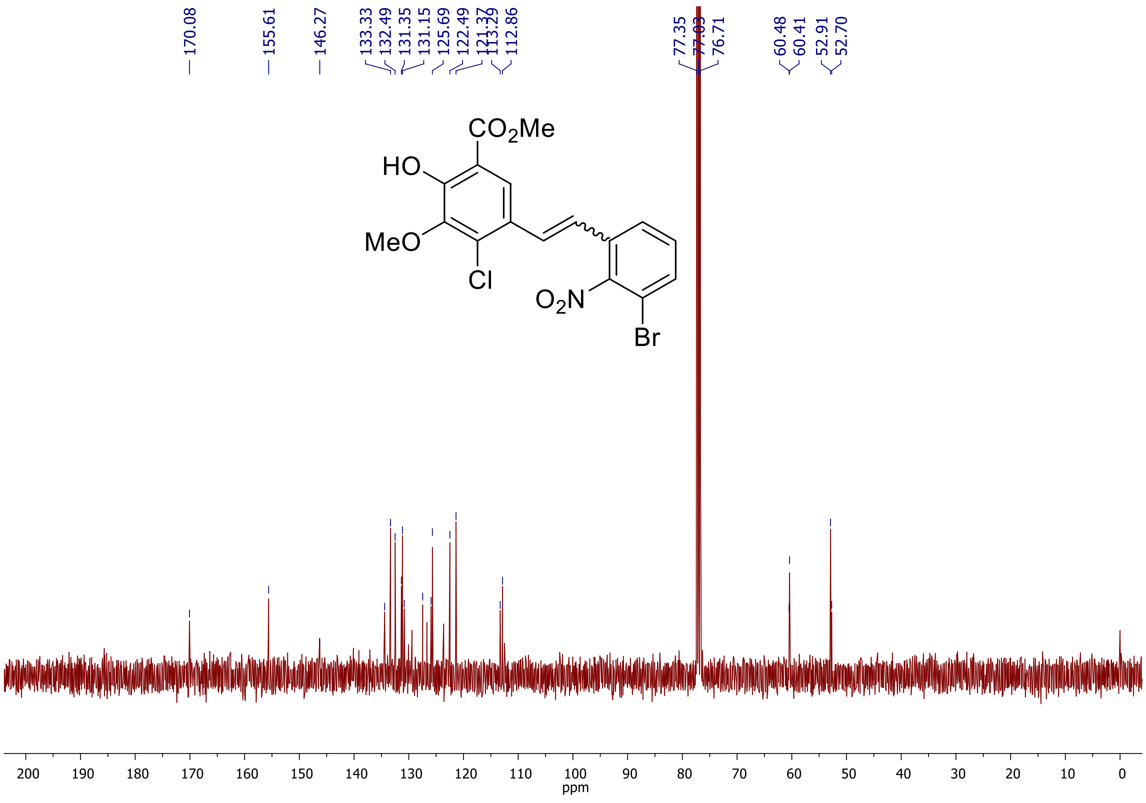

**$^{1}$H NMR (400 MHz, $CDCl_3$) and $^{13}$C NMR (100 MHz, $CDCl_3$) of 9a**

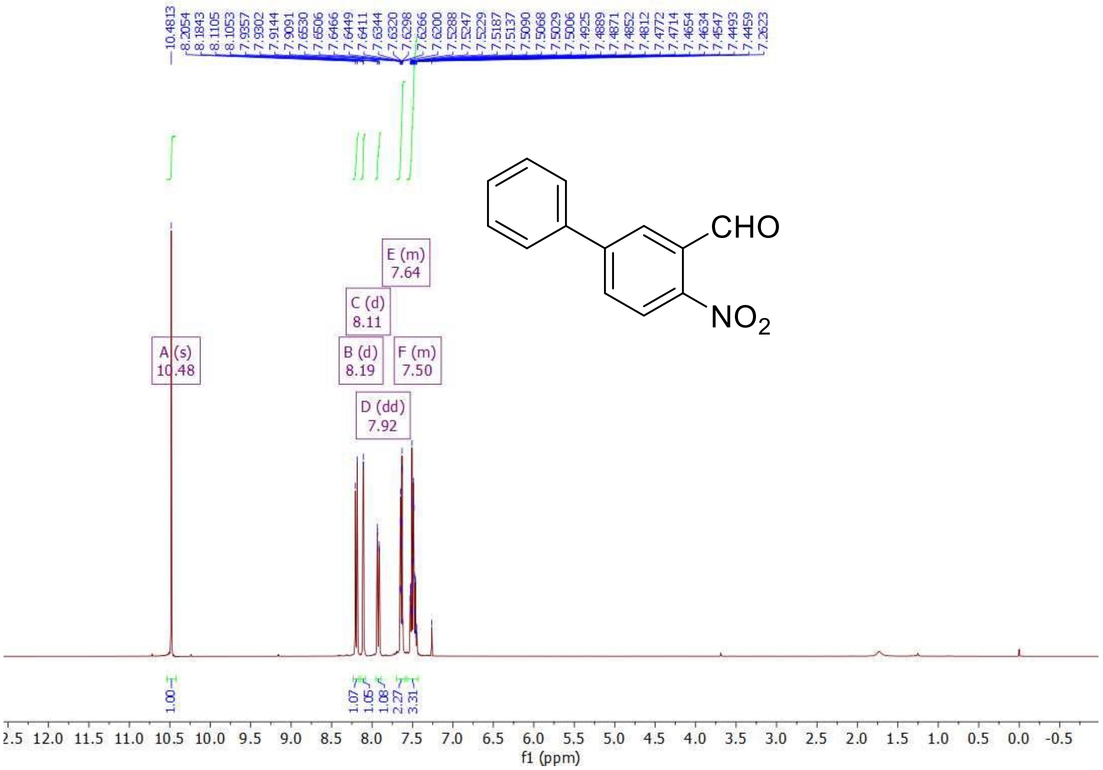


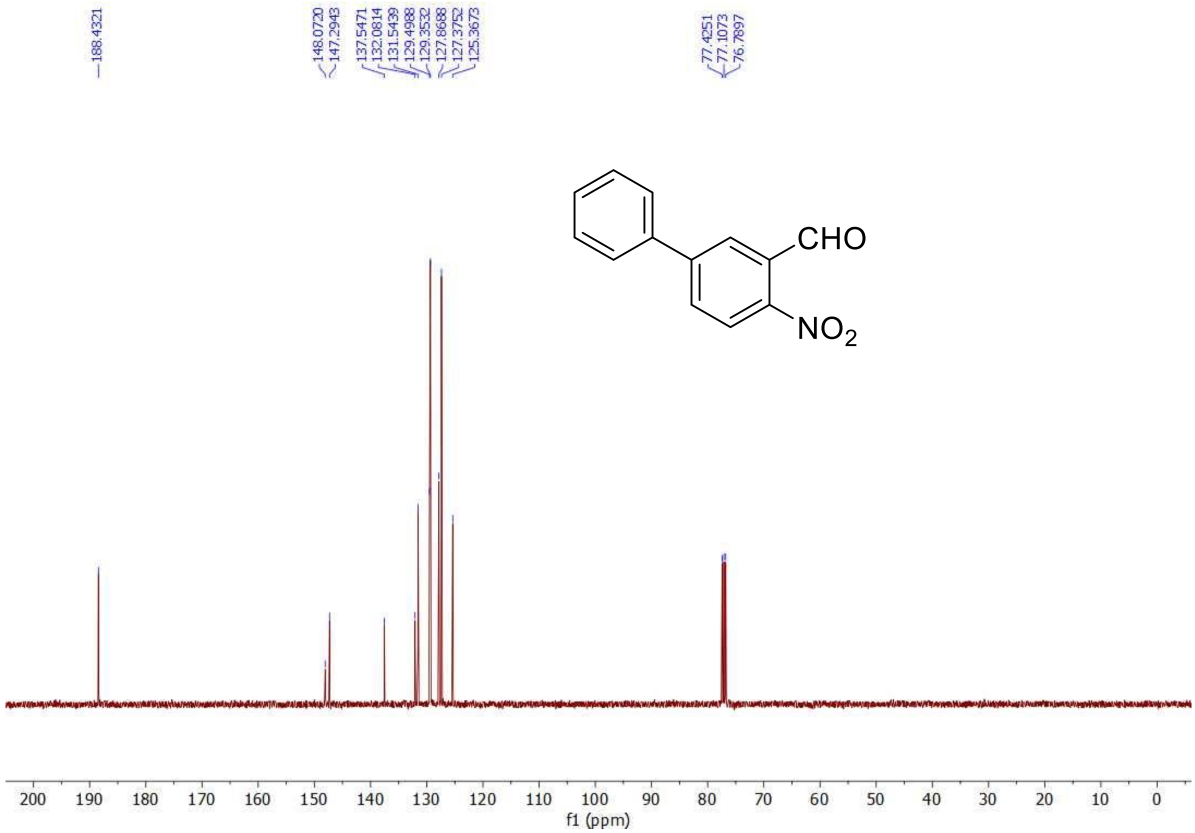

**$^1H$ NMR (400 MHz, $CDCl_3$) and $^{13}C$ NMR (100 MHz, $CDCl_3$) of 9b**

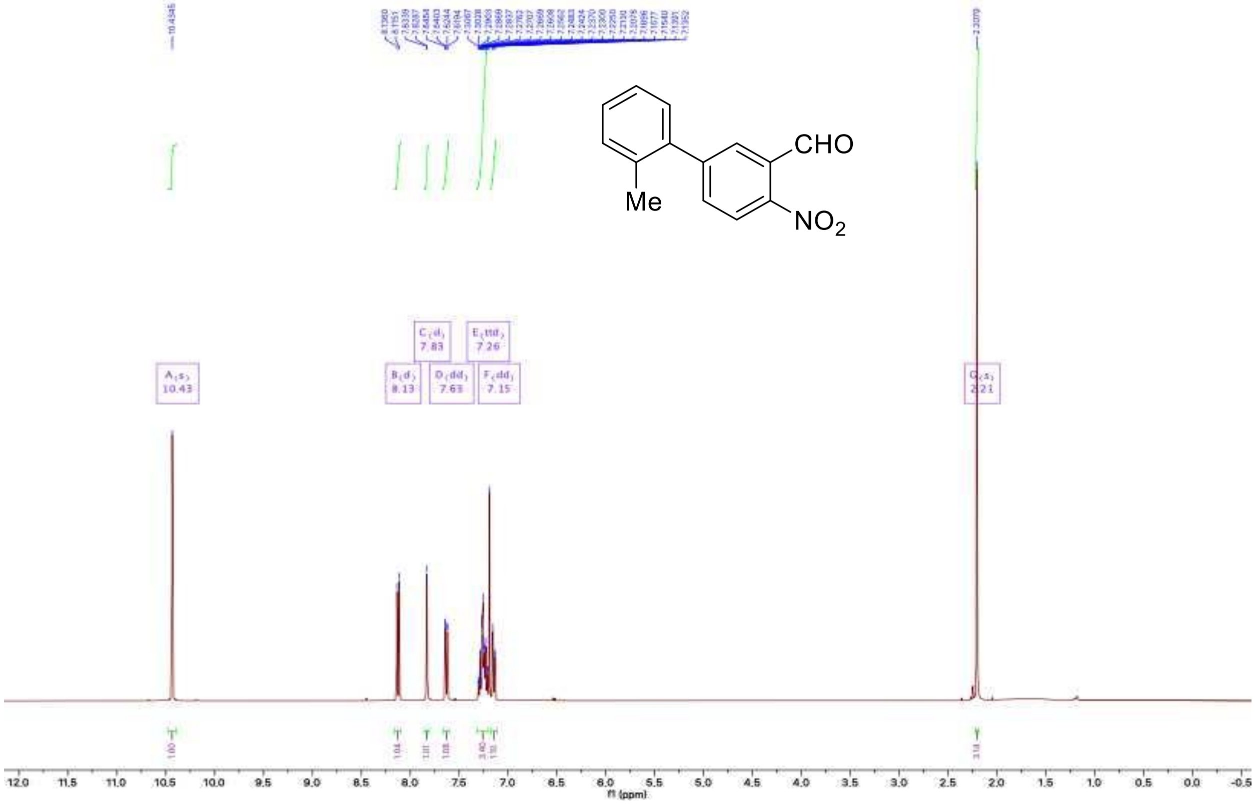


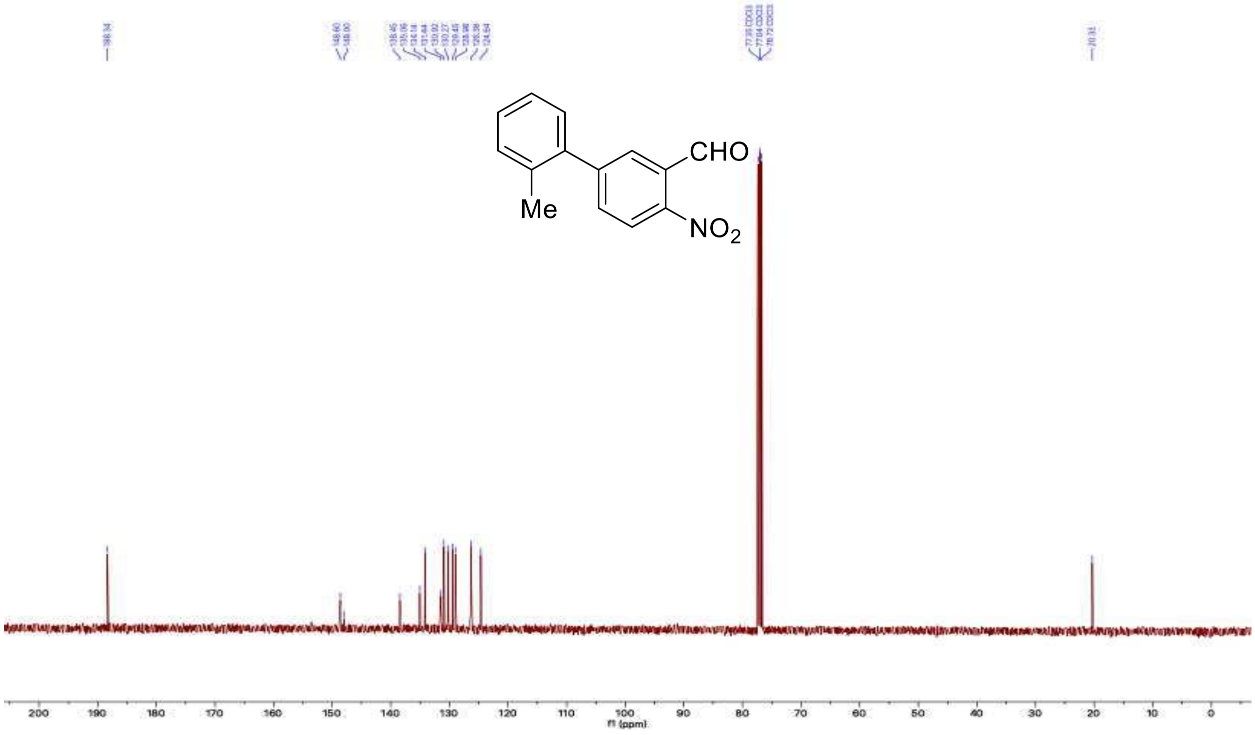


1

**H NMR (400 MHz, $CDCl_3$) and $^{13}$C NMR (100 MHz, $CDCl_3$) of 9c**

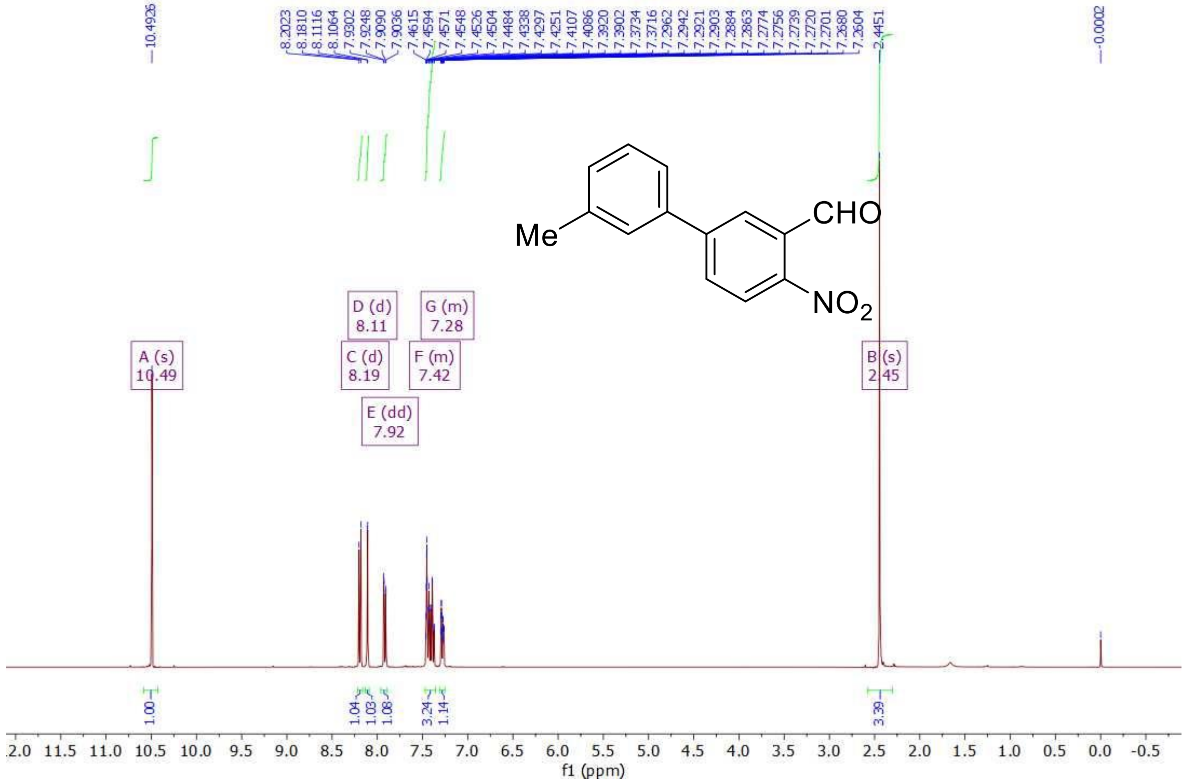


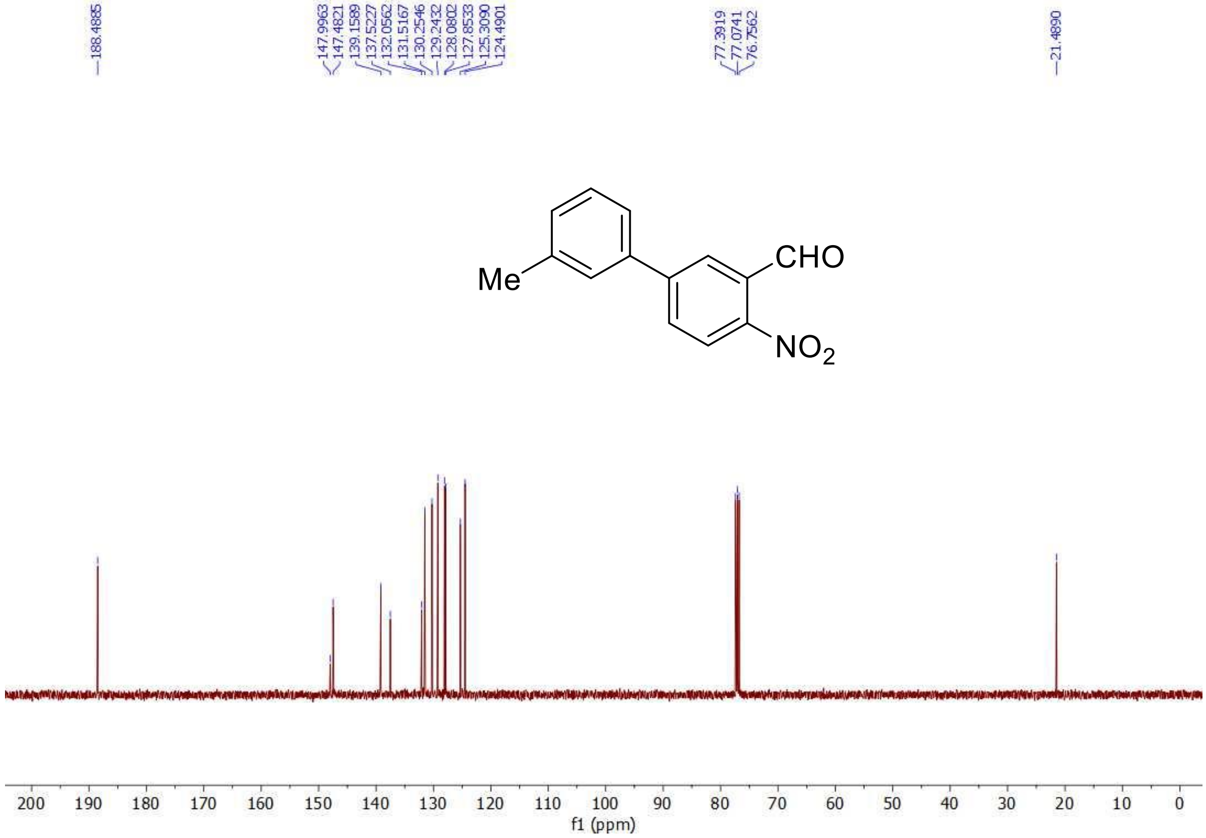

**$^{1}$H NMR (400 MHz, CDCl3) and $^{13}$C NMR (100 MHz, CDCl3) of 9d**

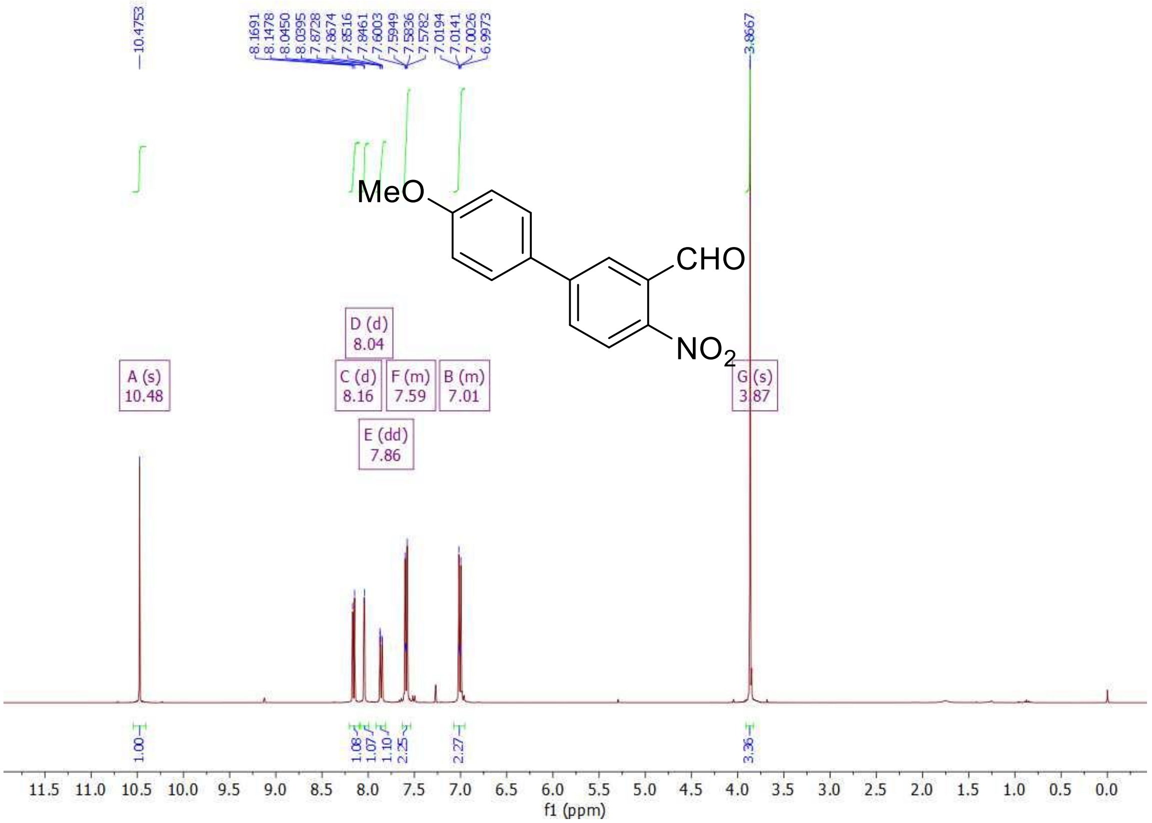


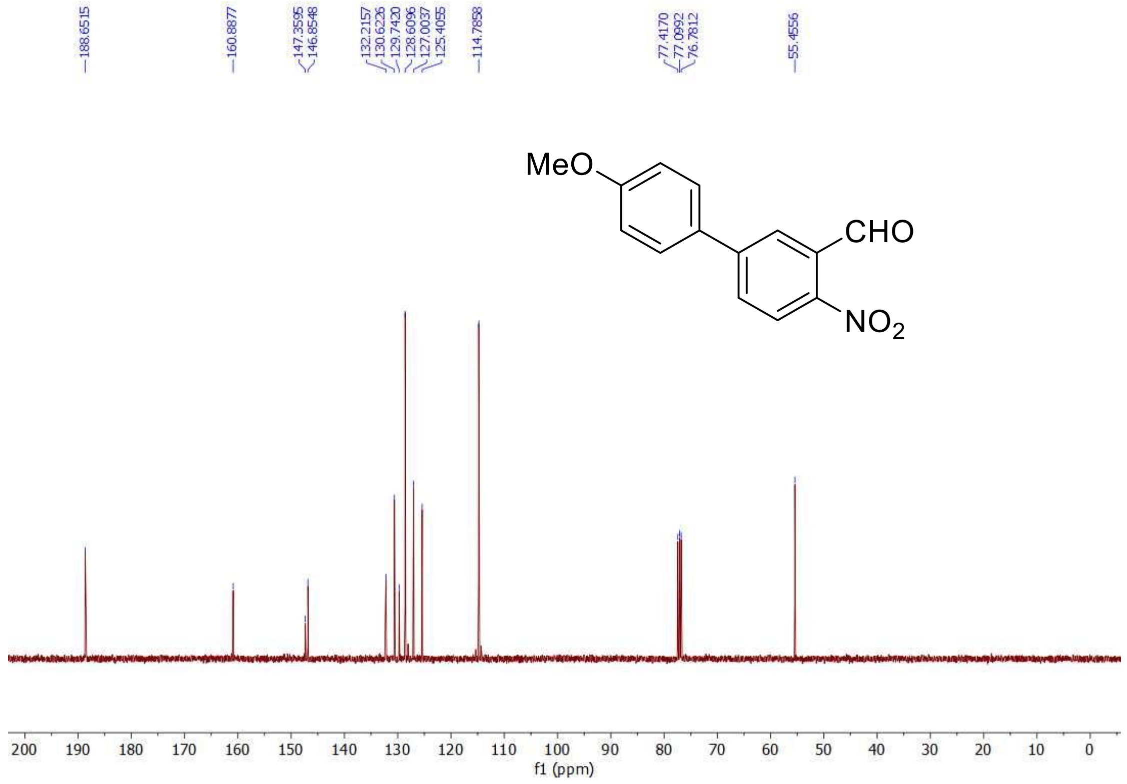

**$^{1}$H NMR (400 MHz, CDCl$_3$) and $^{13}$C NMR (100 MHz, CDCl$_3$) of 9e**

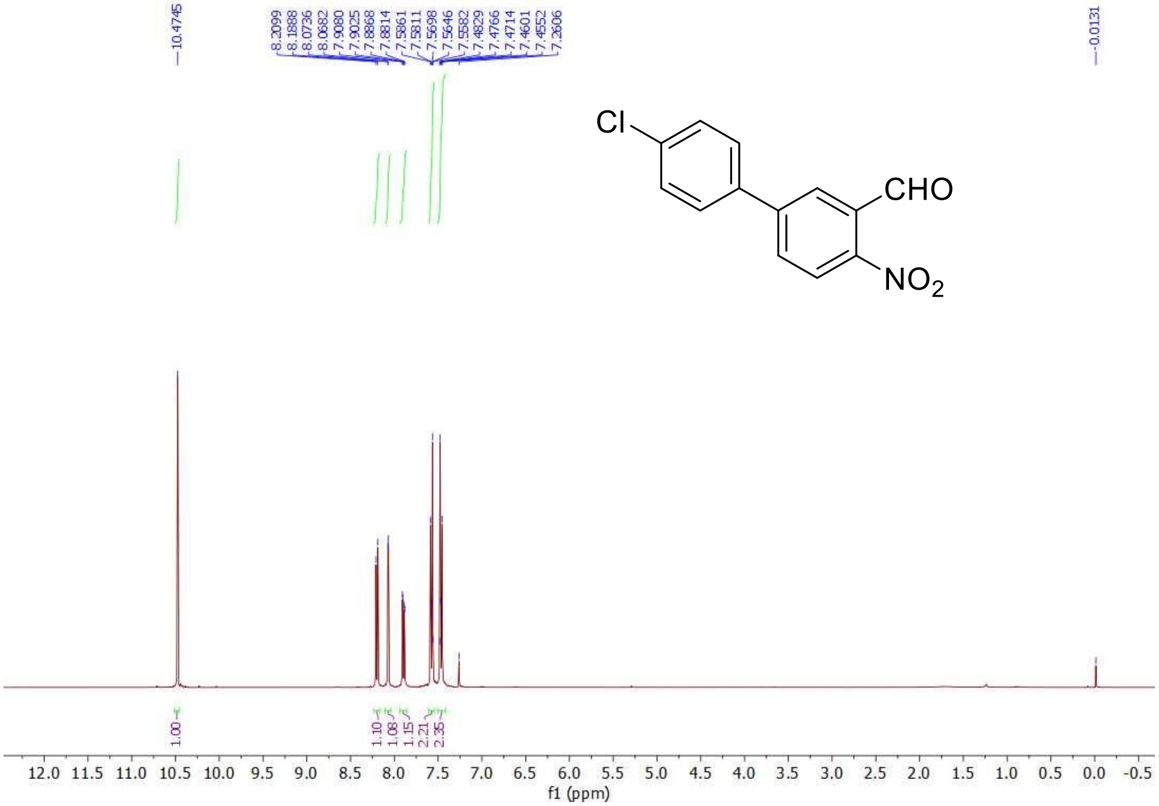

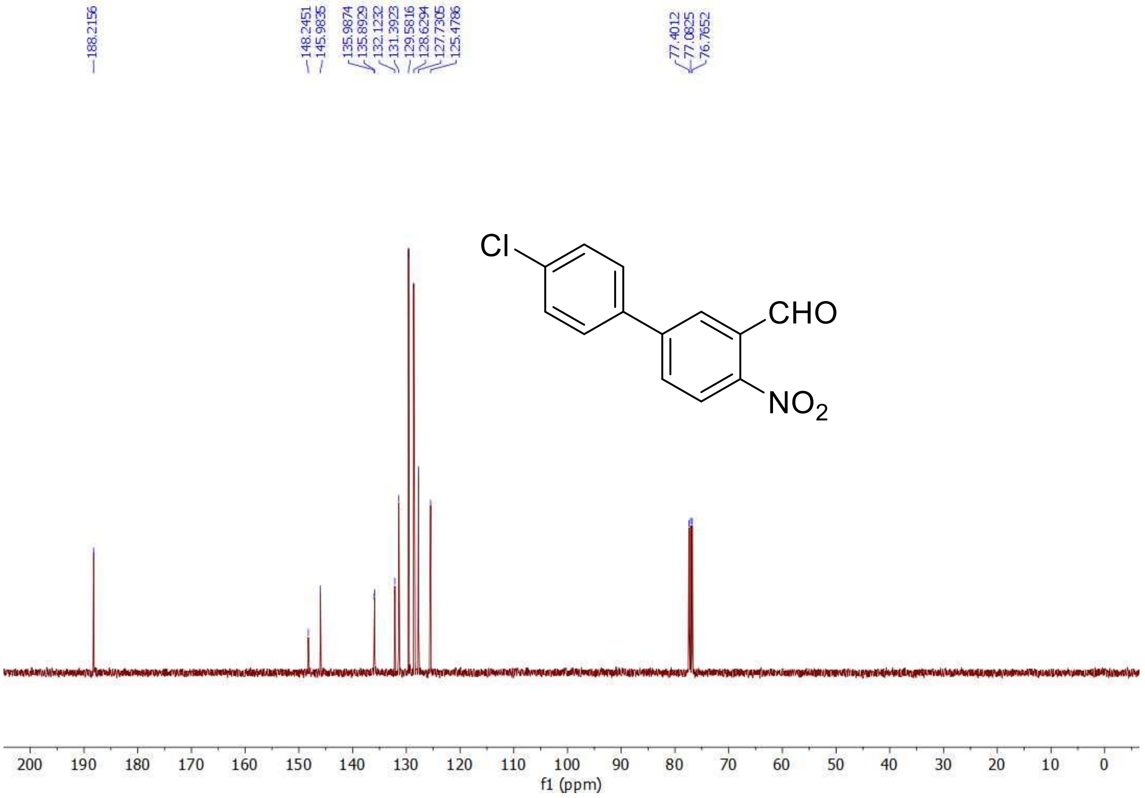

**$^{1}$H NMR (400 MHz, CDCl$_3$) and $^{13}$C NMR (100 MHz, CDCl$_3$) of 9f**

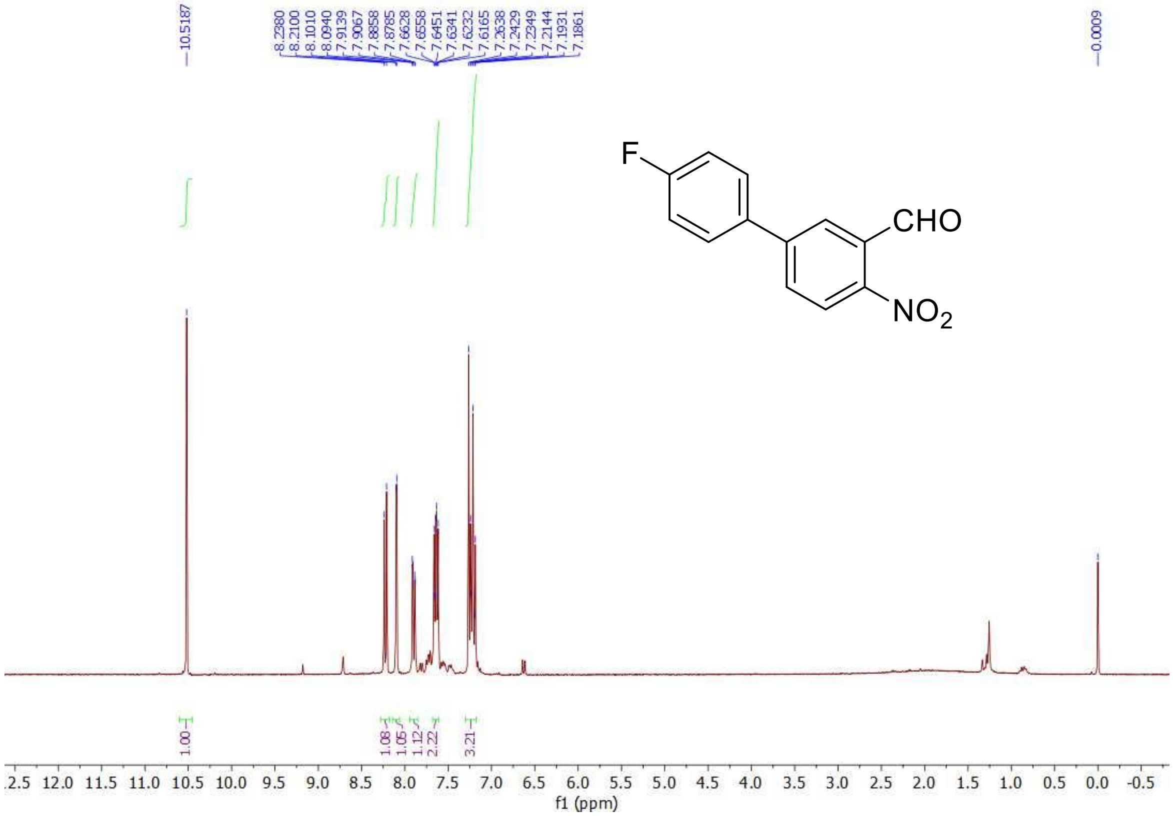


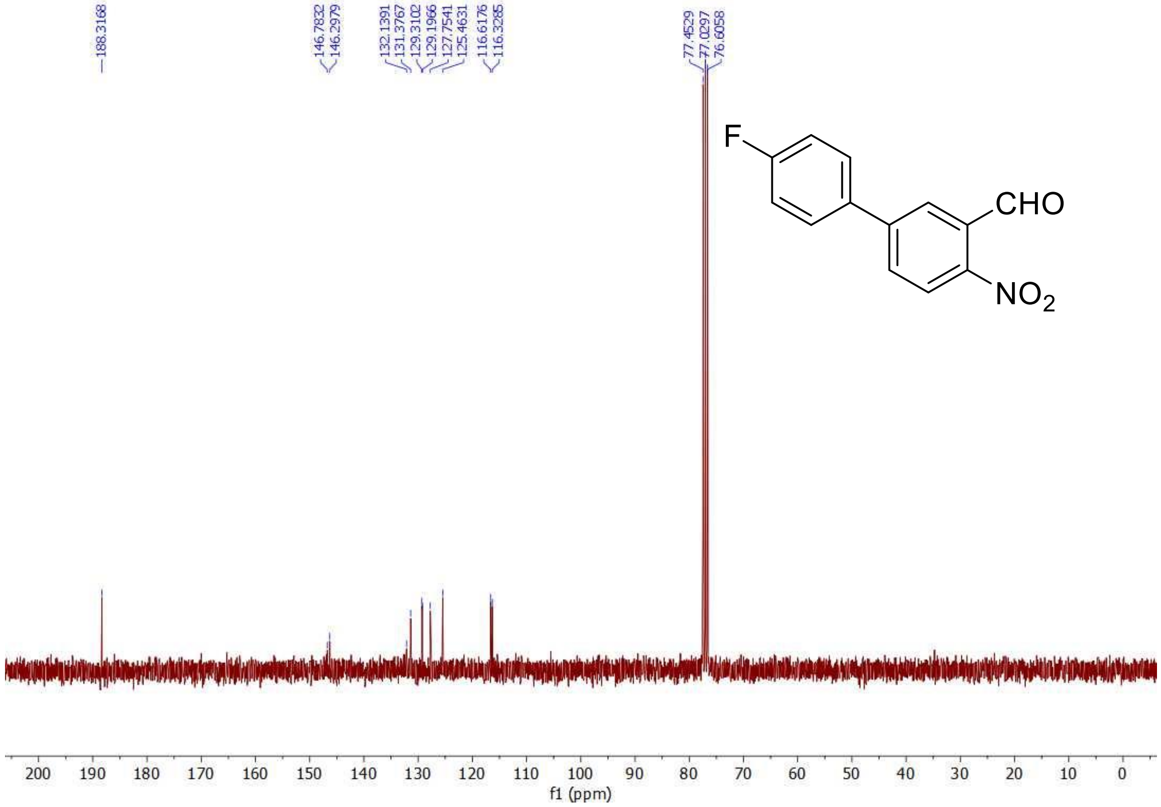

**$^{1}H$ NMR (400 MHz, $CDCl_3$) and $^{13}C$ NMR (100 MHz, $CDCl_3$) of 9g**

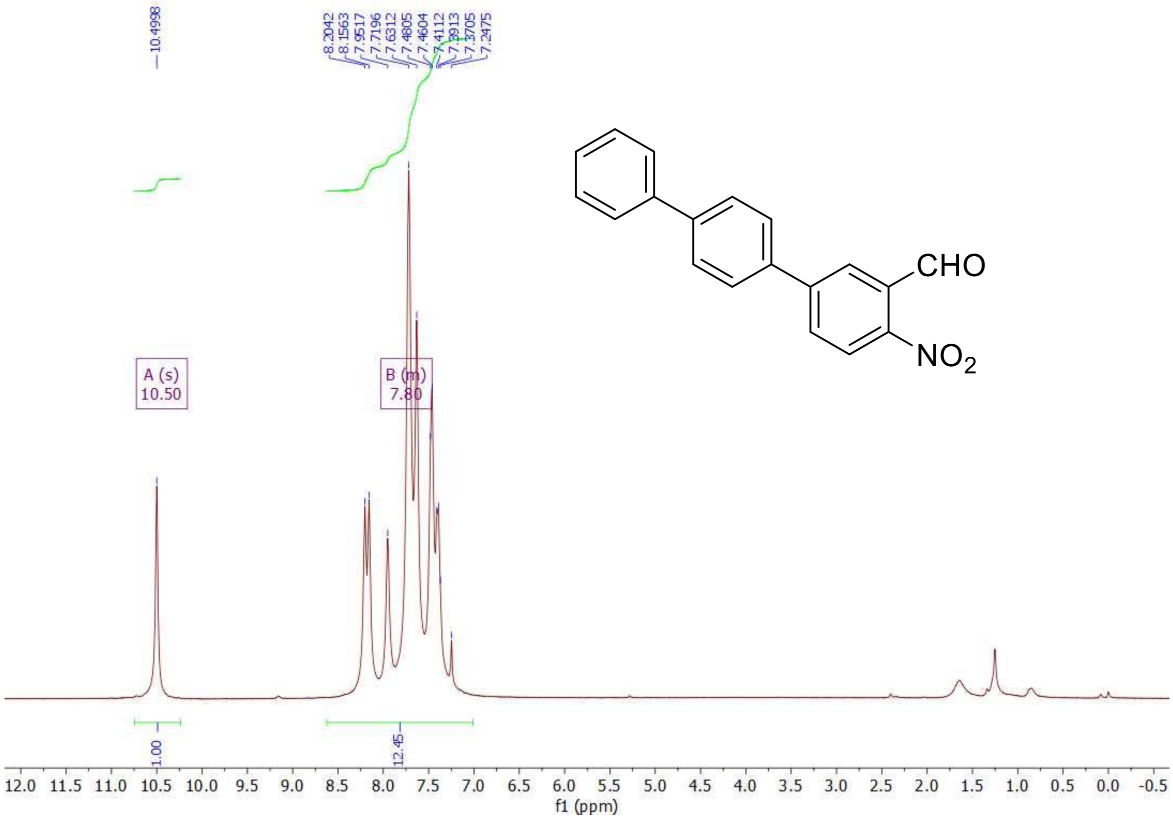


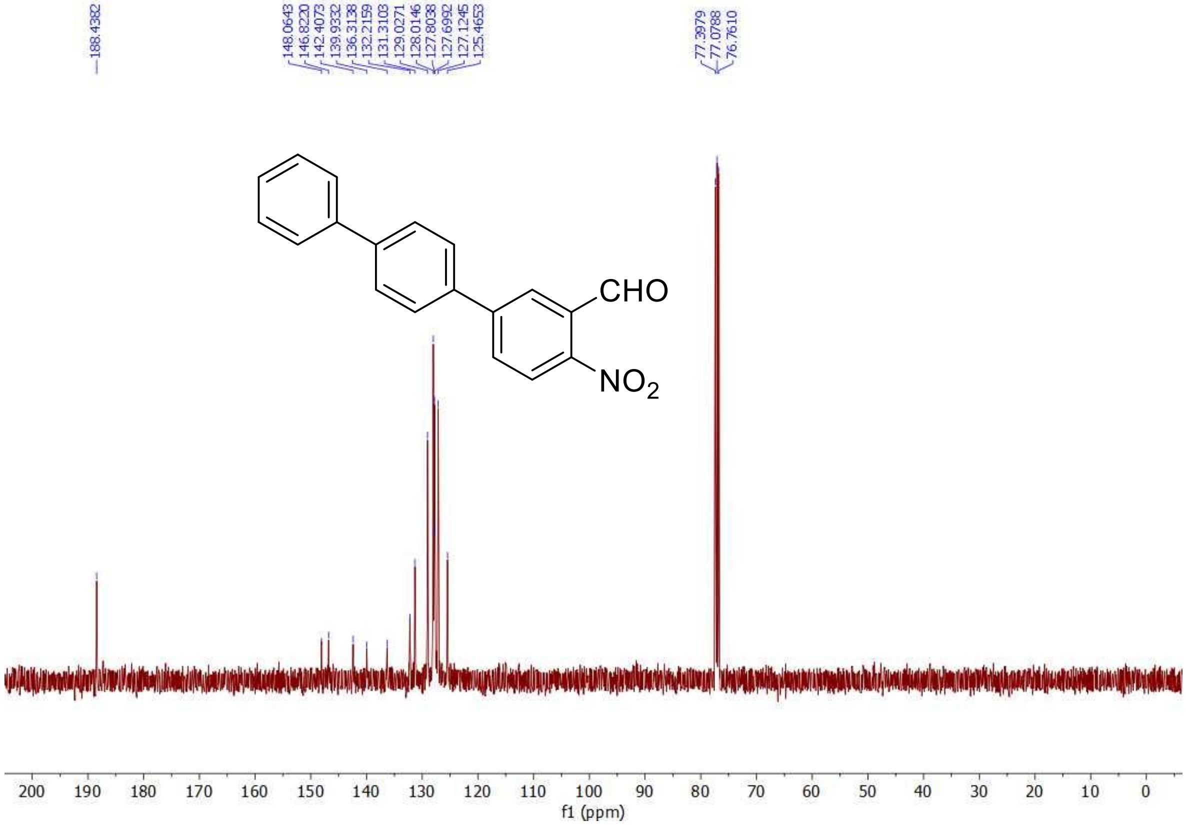

**$^{1}H$ NMR (400 MHz, $CDCl_3$) and $^{13}C$ NMR (100 MHz, $CDCl_3$) of 9h**

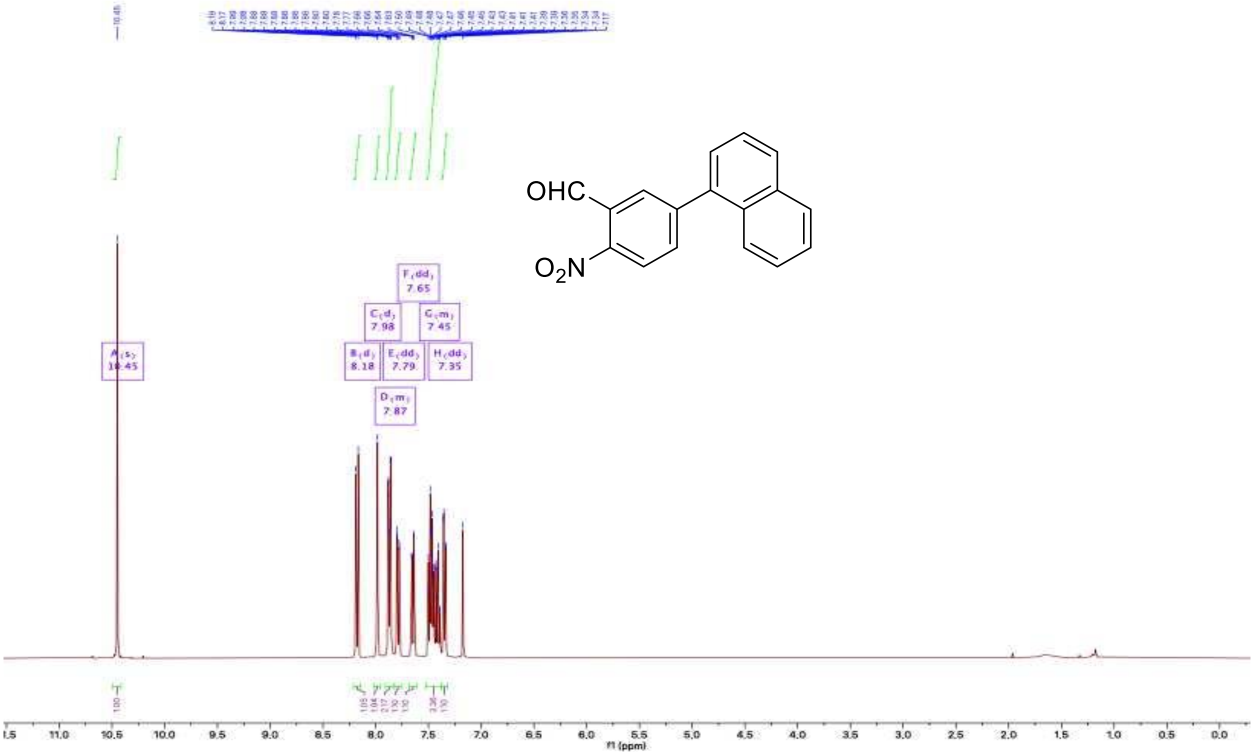


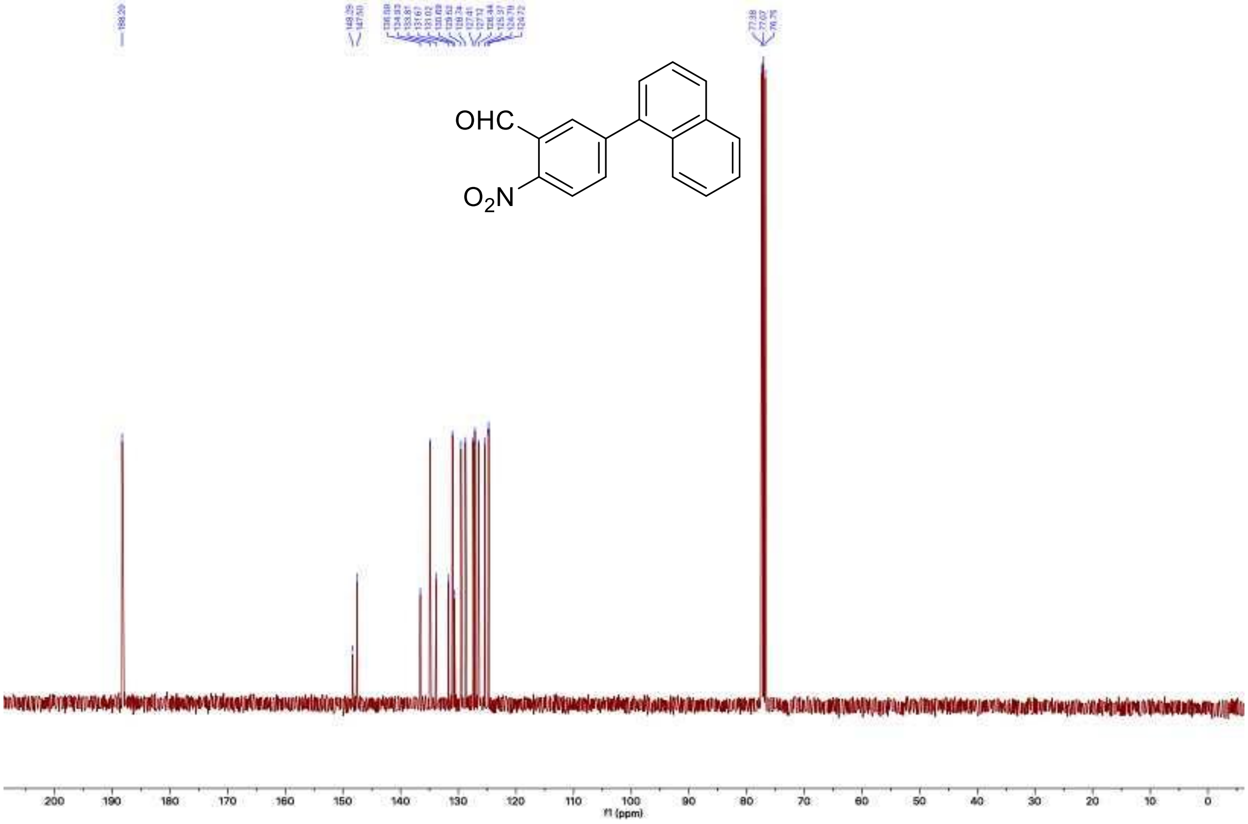

**$^{1}H$ NMR (400 MHz, $CDCl_3$) and $^{13}C$ NMR (100 MHz, $CDCl_3$) of 9i**

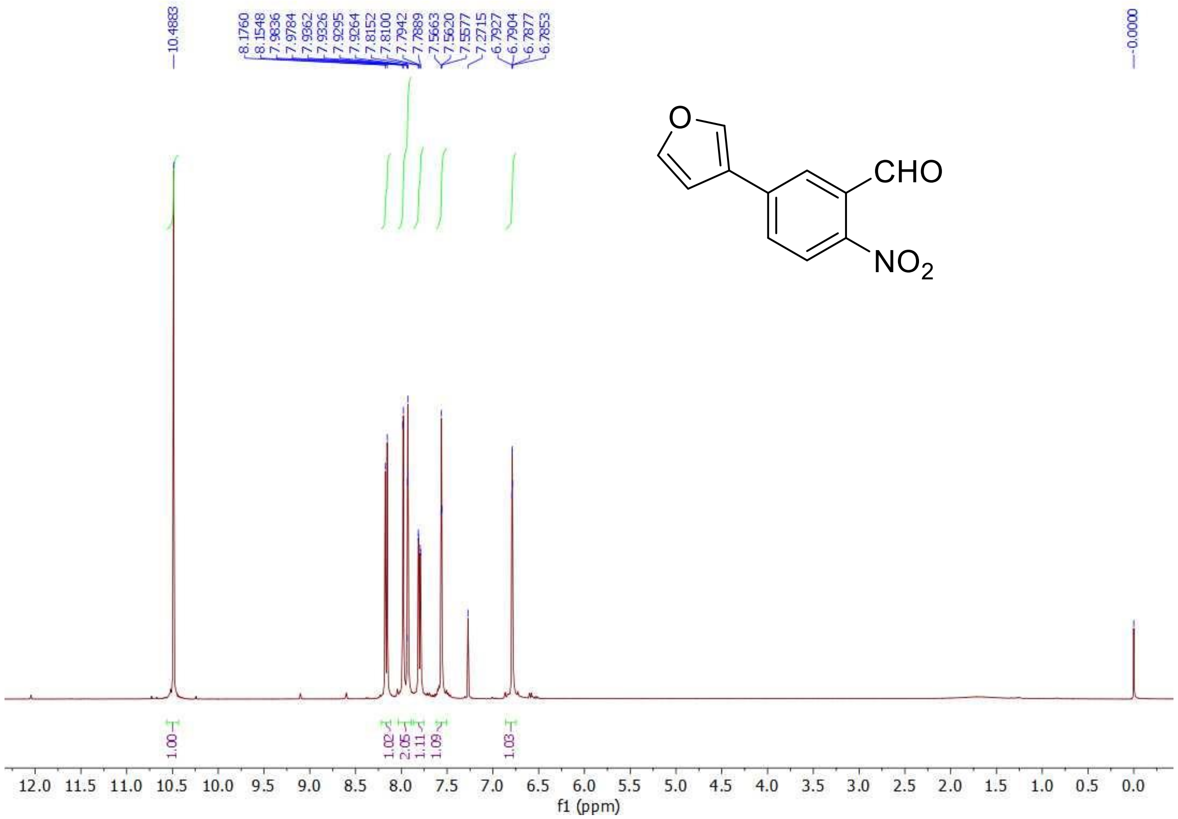


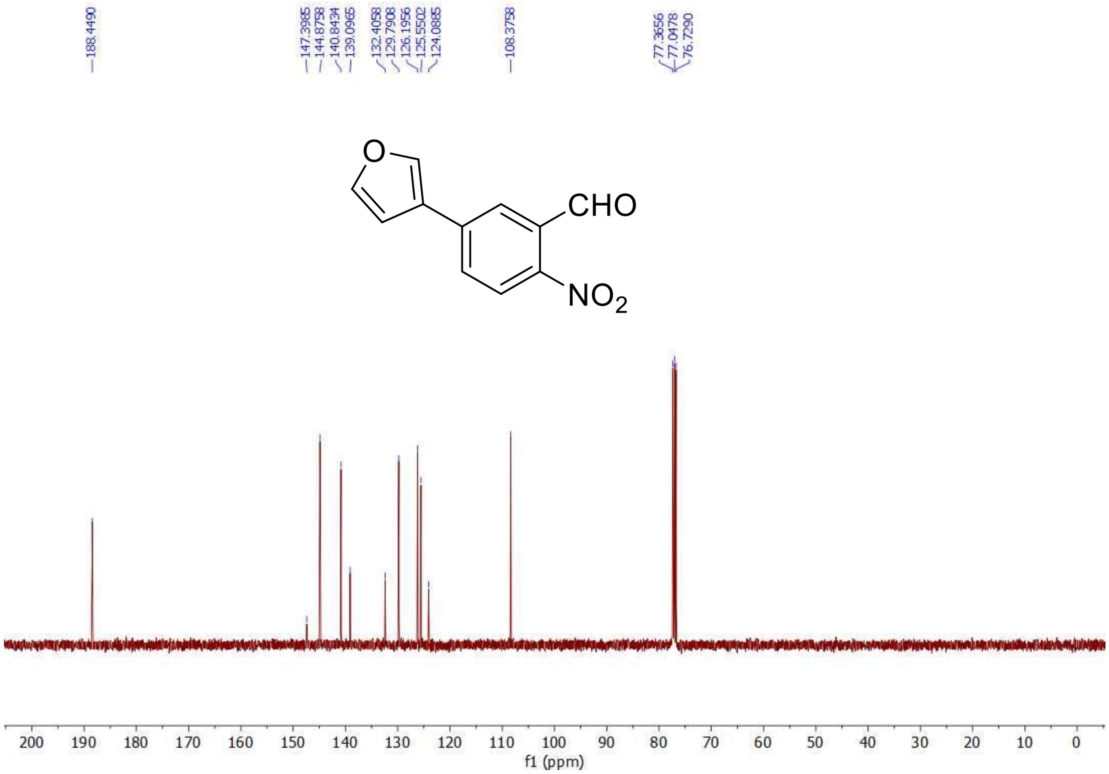

# $^{1}H$ NMR (400 MHz, $CDCl_3$) and $^{13}C$ NMR (100 MHz, $CDCl_3$) of 10a

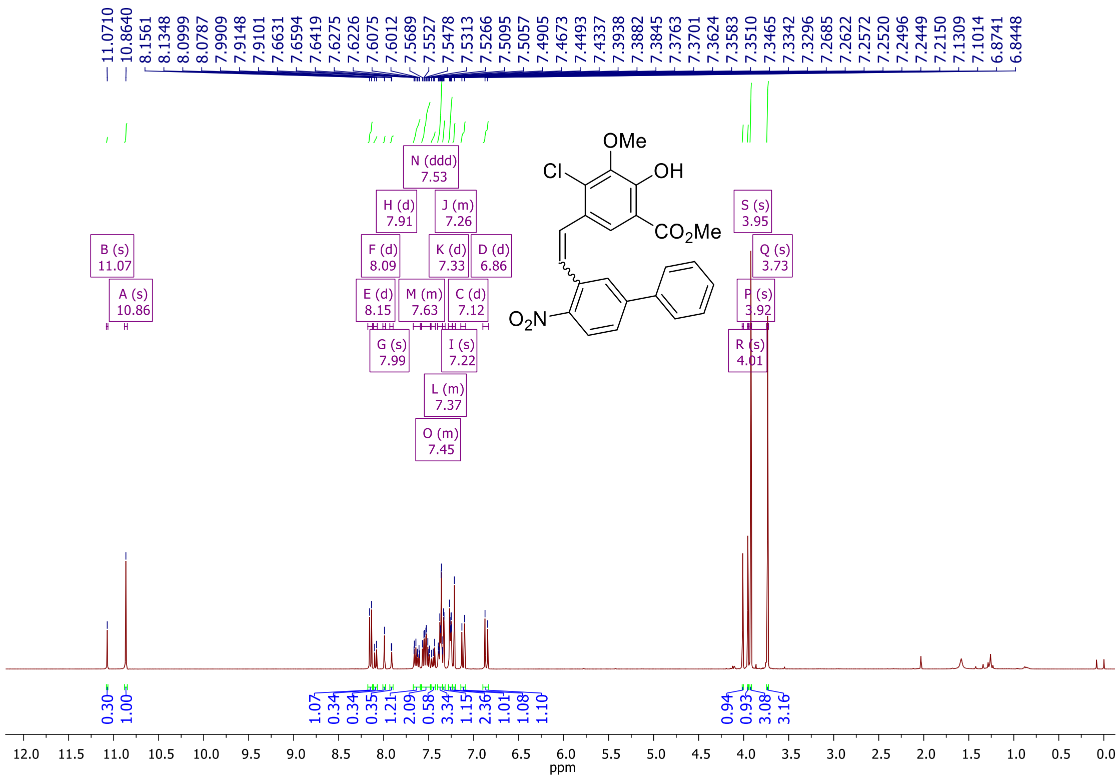

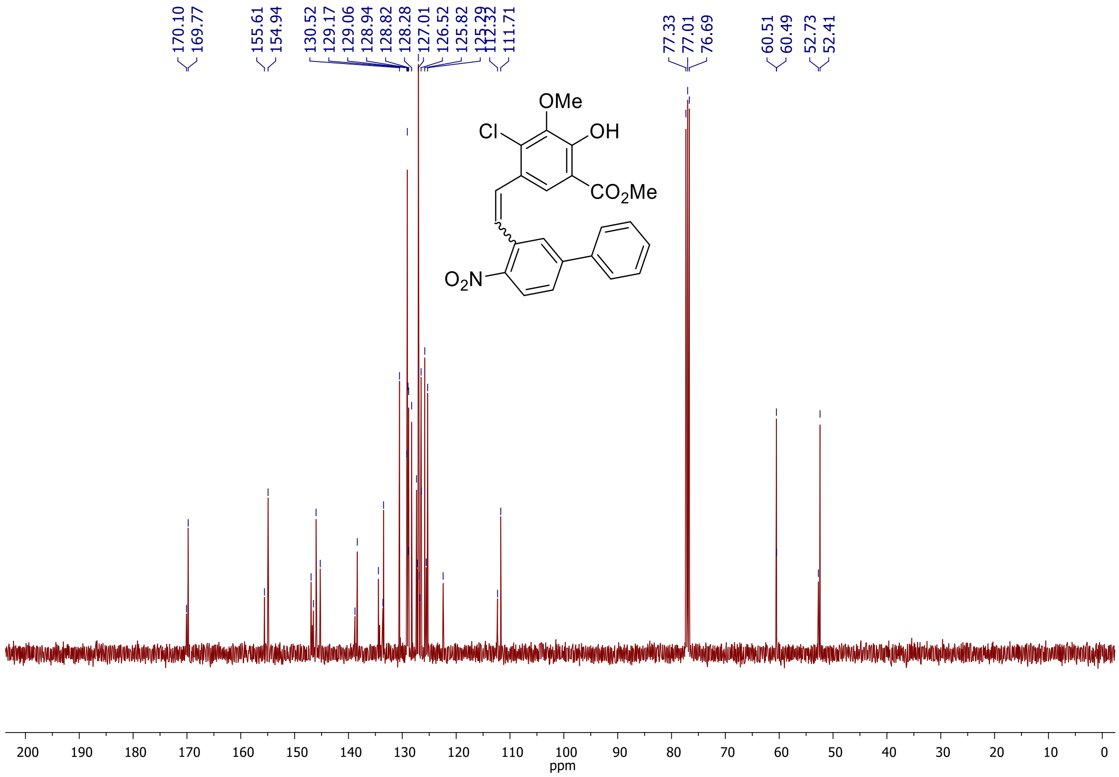

## $^{1}H$ NMR (400 MHz, $CDCl_3$) and $^{13}C$ NMR (100 MHz, $CDCl_3$) of 10b

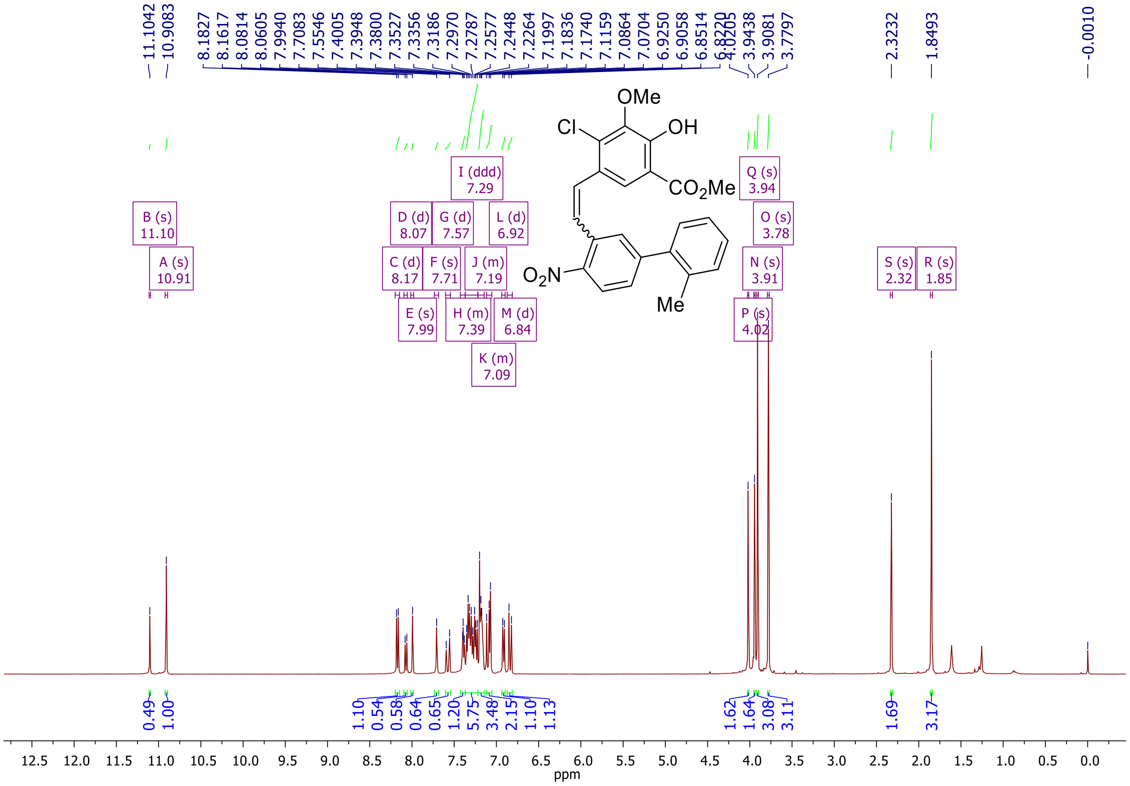

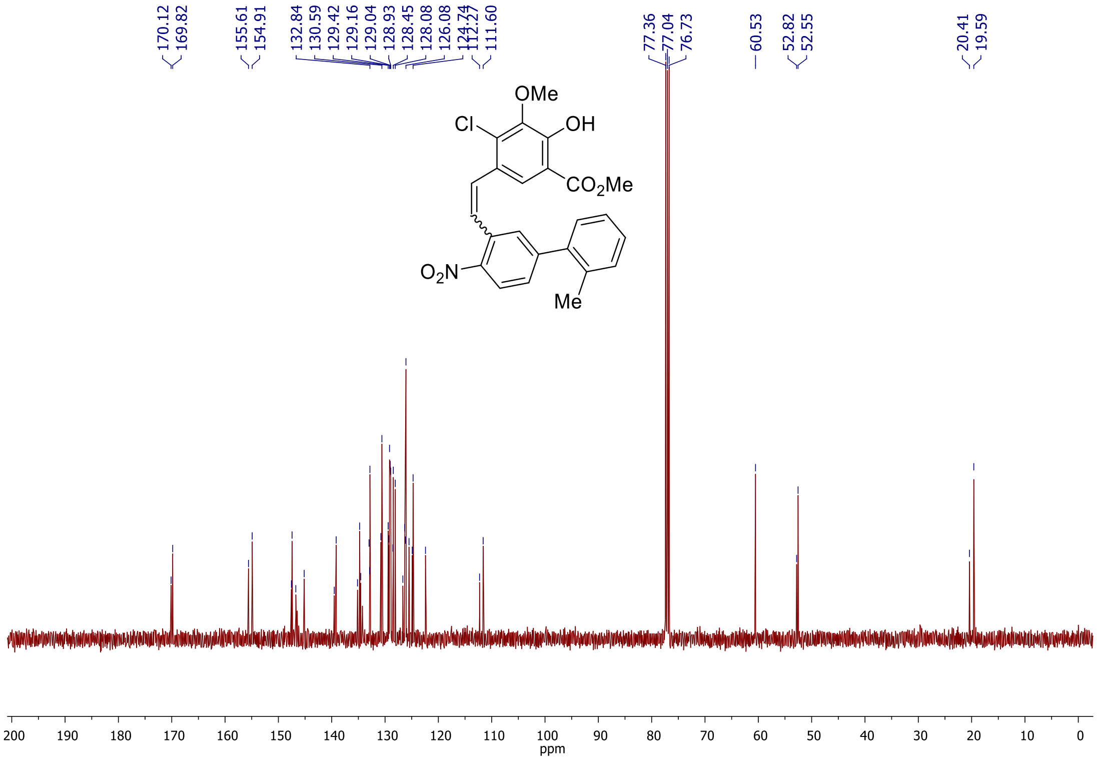

# $^1$H NMR (400 MHz, $CDCl_3$) and $^{13}$C NMR (100 MHz, $CDCl_3$) of 10c

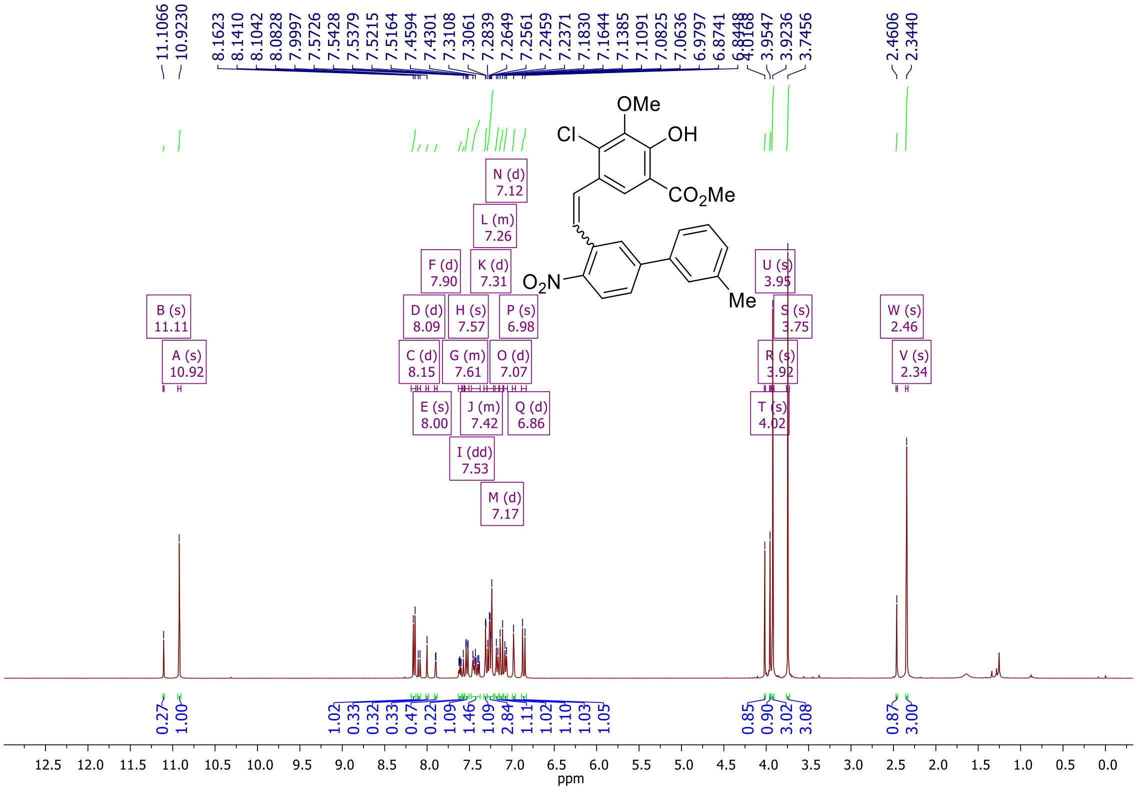


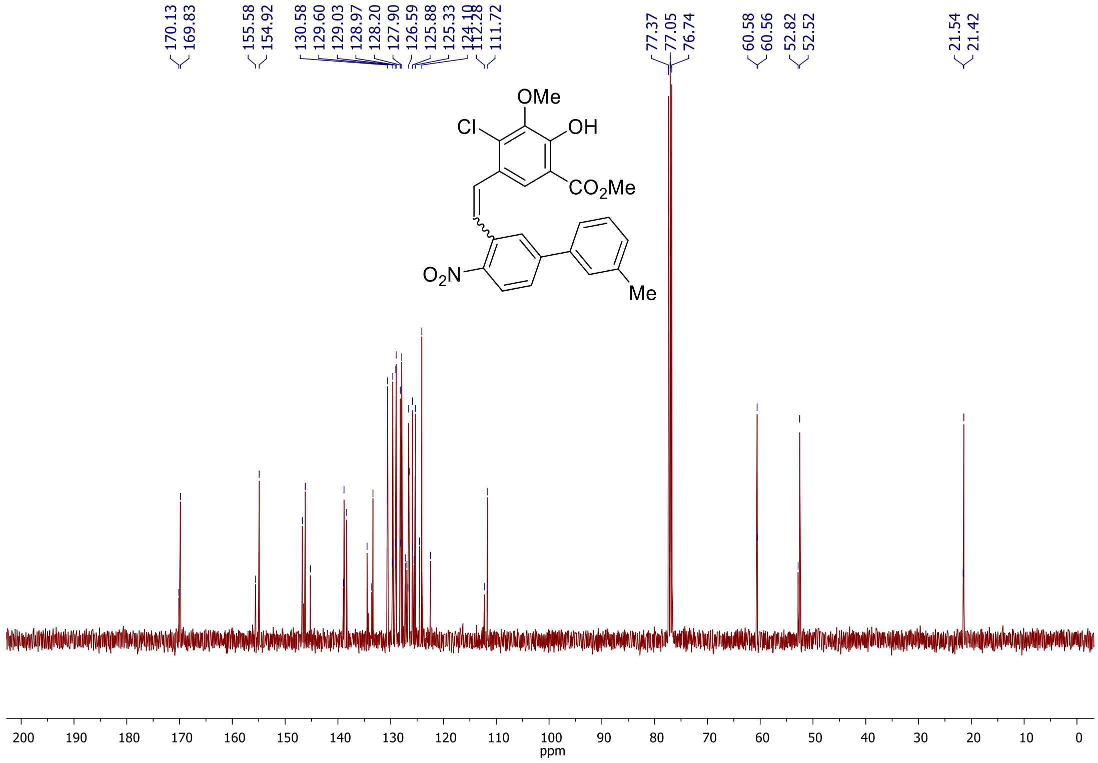

## $^{1}H$ NMR (400 MHz, $CDCl_3$) and $^{13}C$ NMR (100 MHz, $CDCl_3$) of 10d

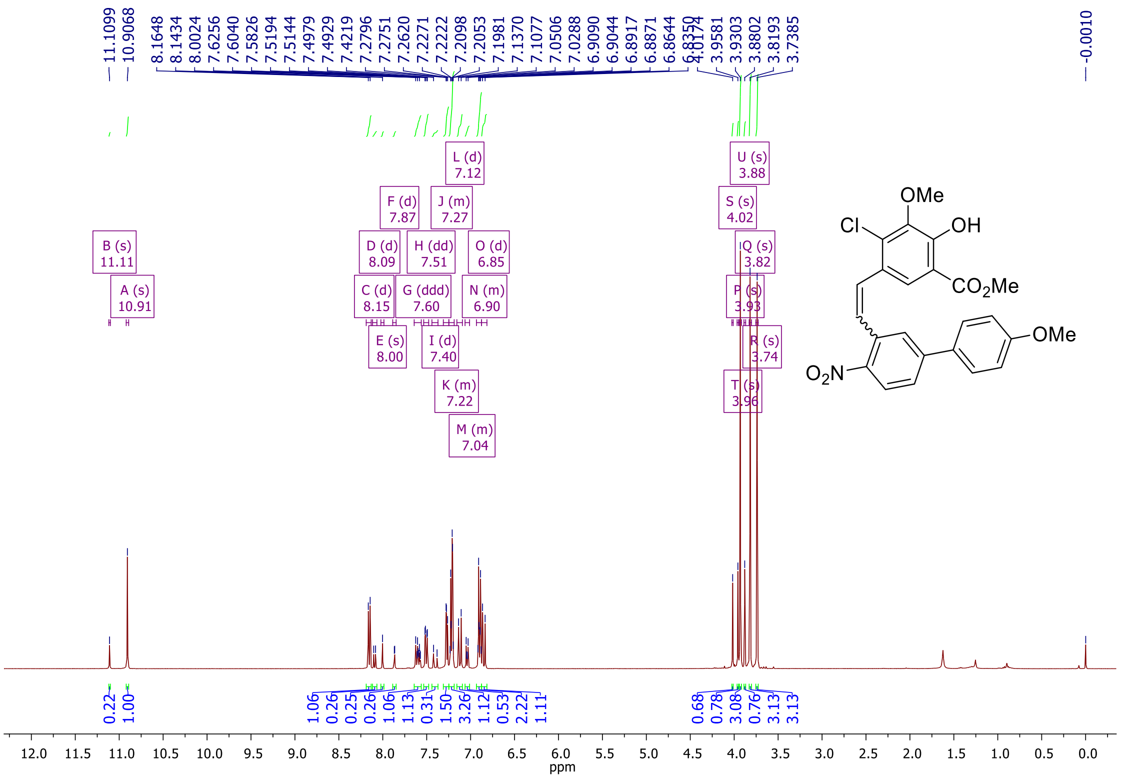


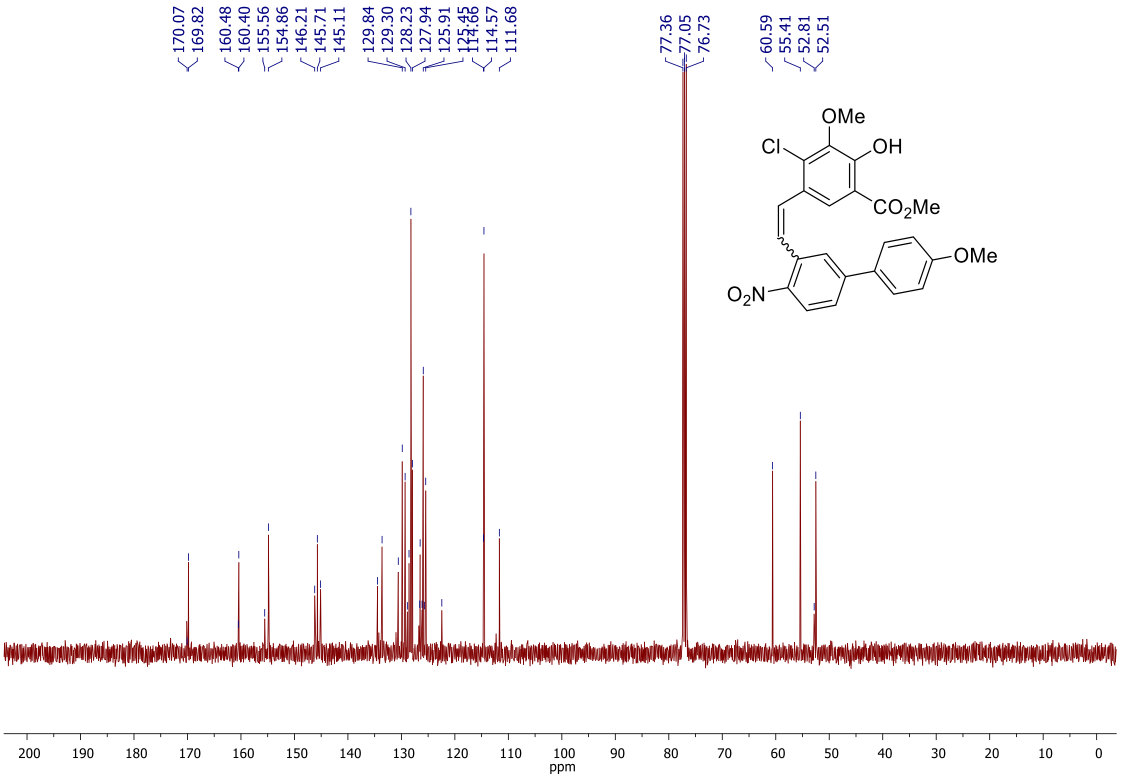

**$^{1}H$ NMR (400 MHz, $CDCl_3$) and $^{13}C$ NMR (100 MHz, $CDCl_3$) of 10e**

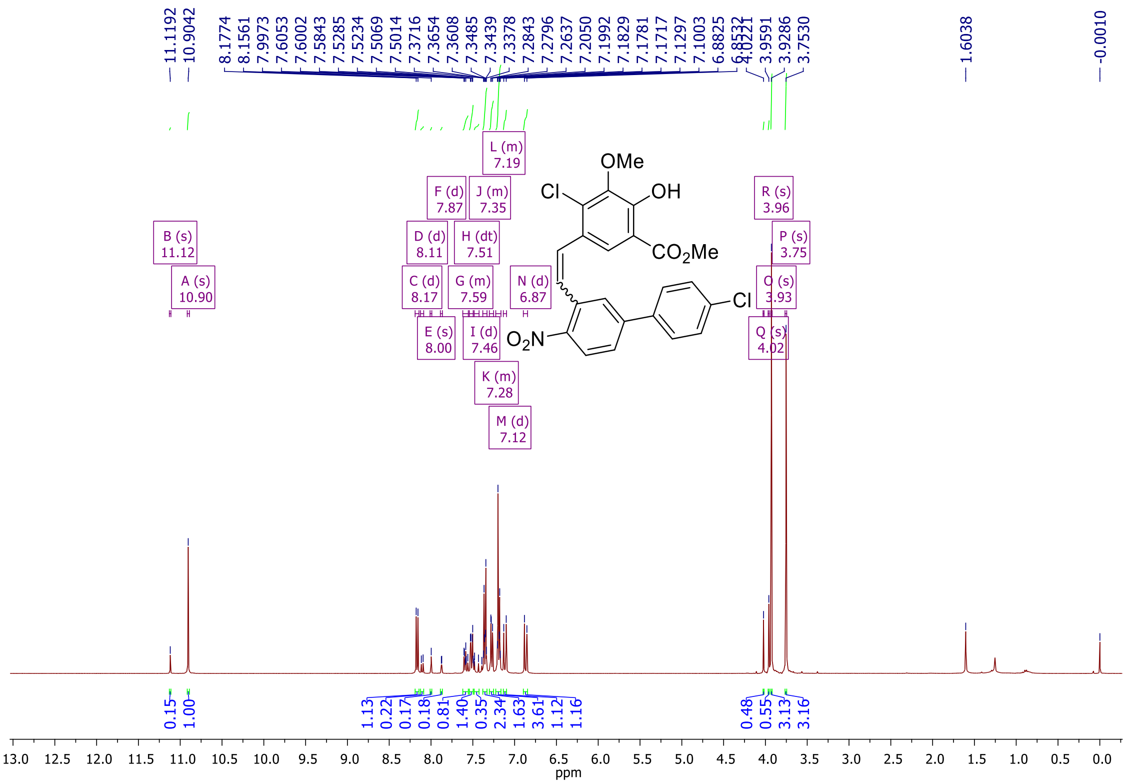

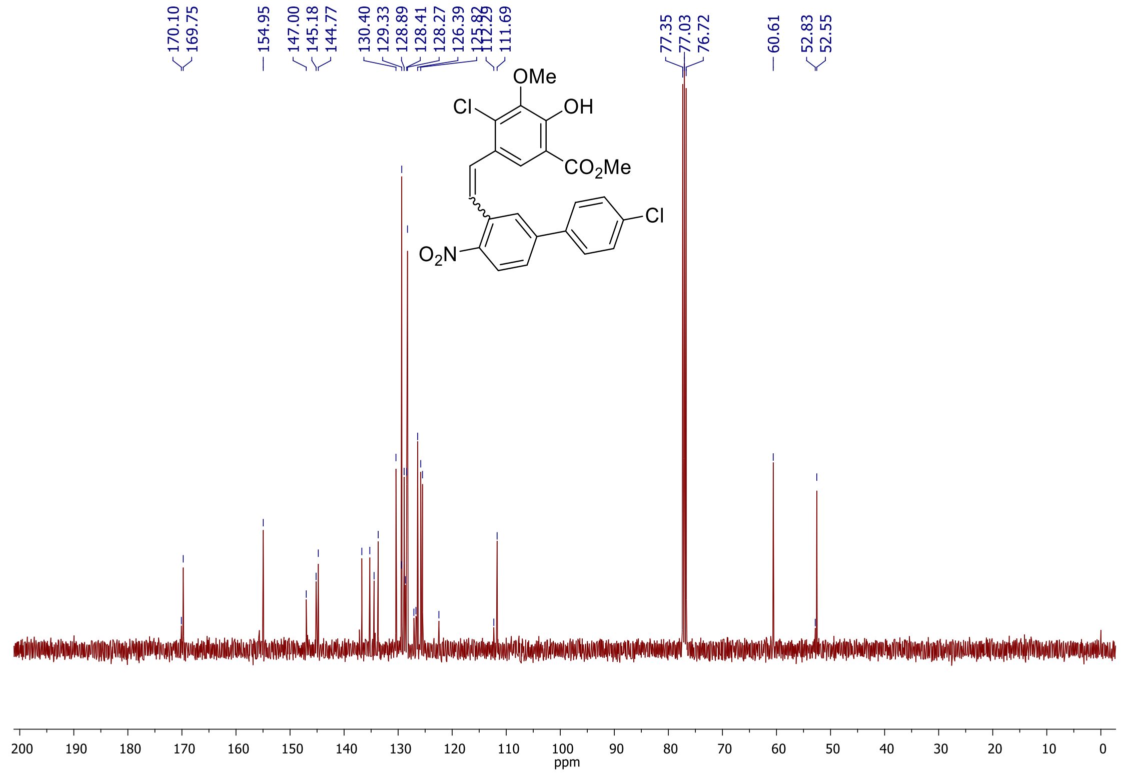

## $^{1}$H NMR (400 MHz, $CDCl_3$) and $^{13}$C NMR (100 MHz, $CDCl_3$) of 10f

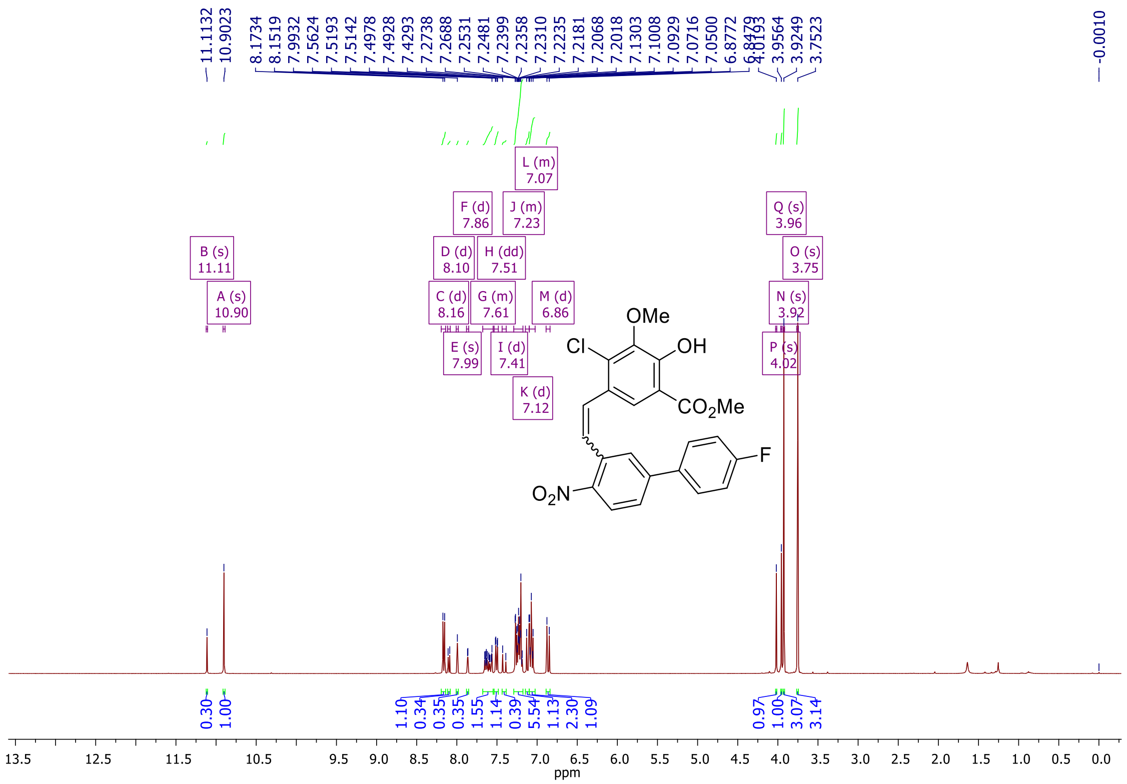

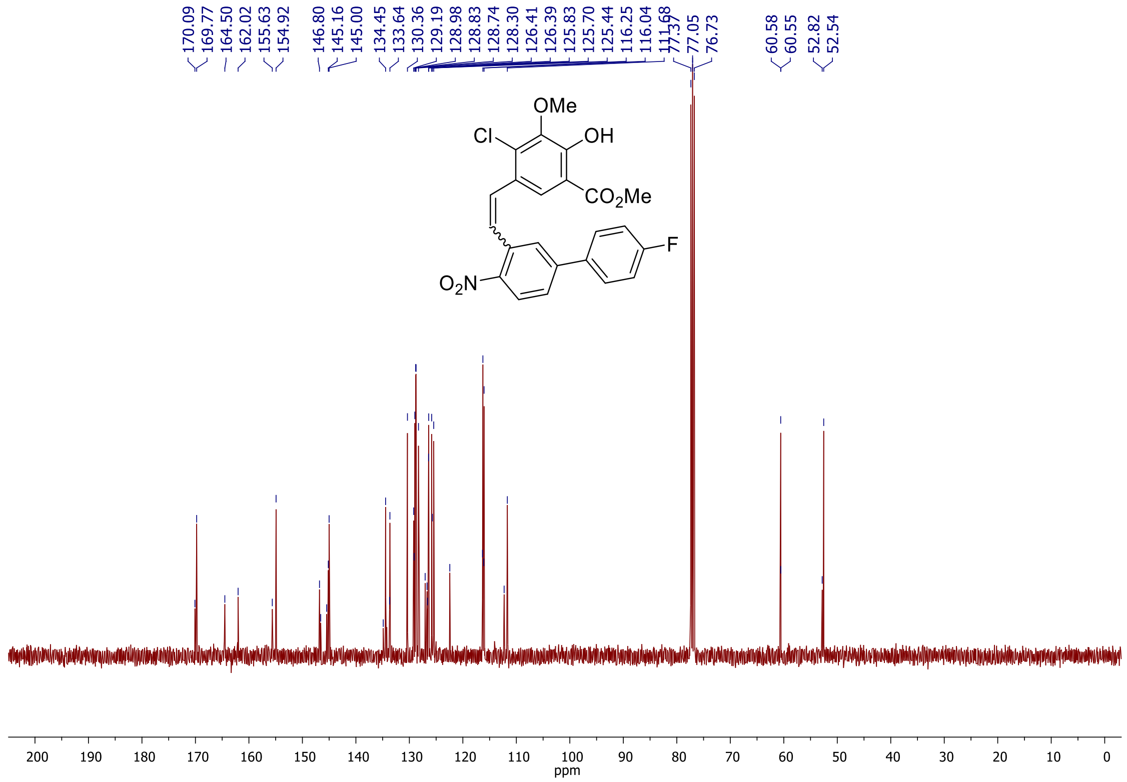

## $^1H$ NMR (400 MHz, $CDCl_3$) of 10g crude reaction mixture without $SiO_2$ filtration

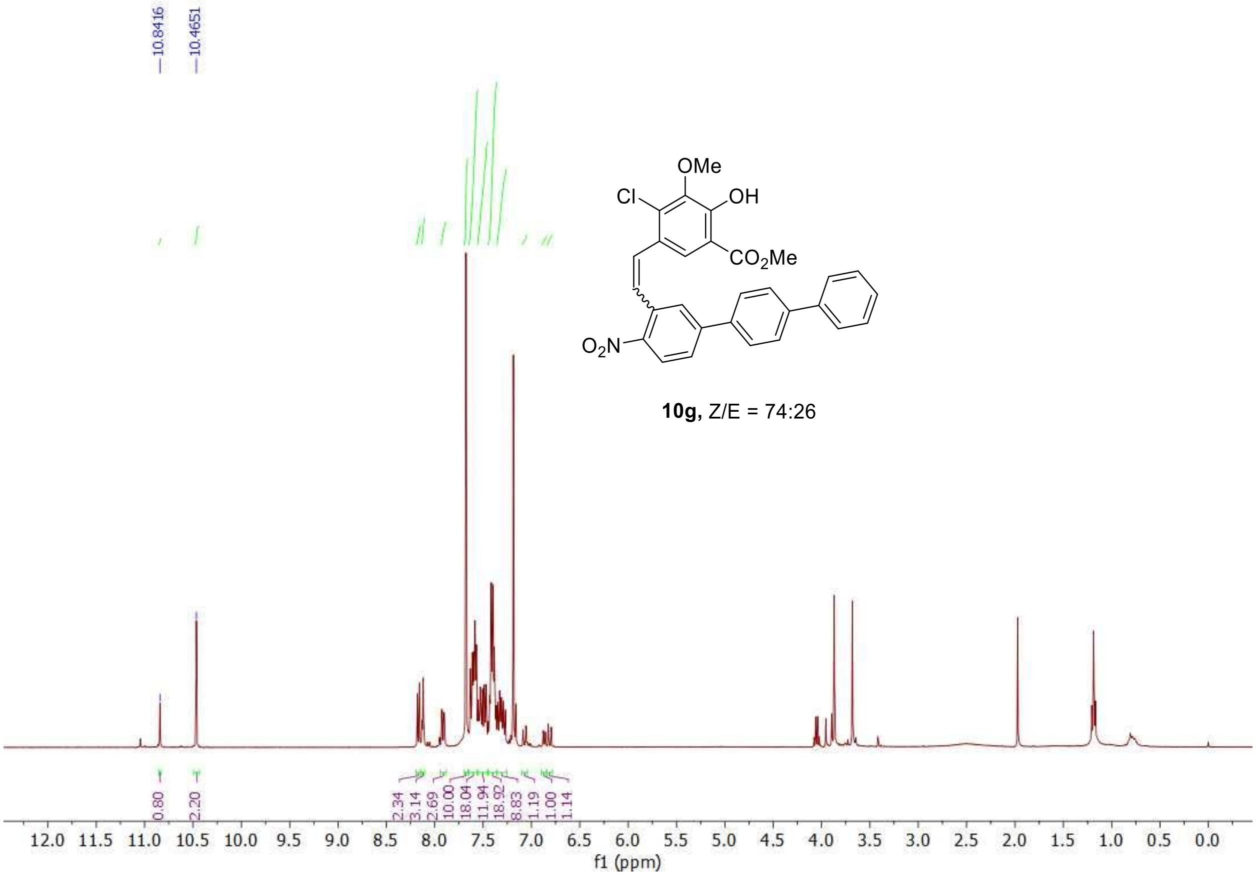

## $^{1}$H NMR (400 MHz, $CDCl_3$) and $^{13}$C NMR (100 MHz, $CDCl_3$) of 10g

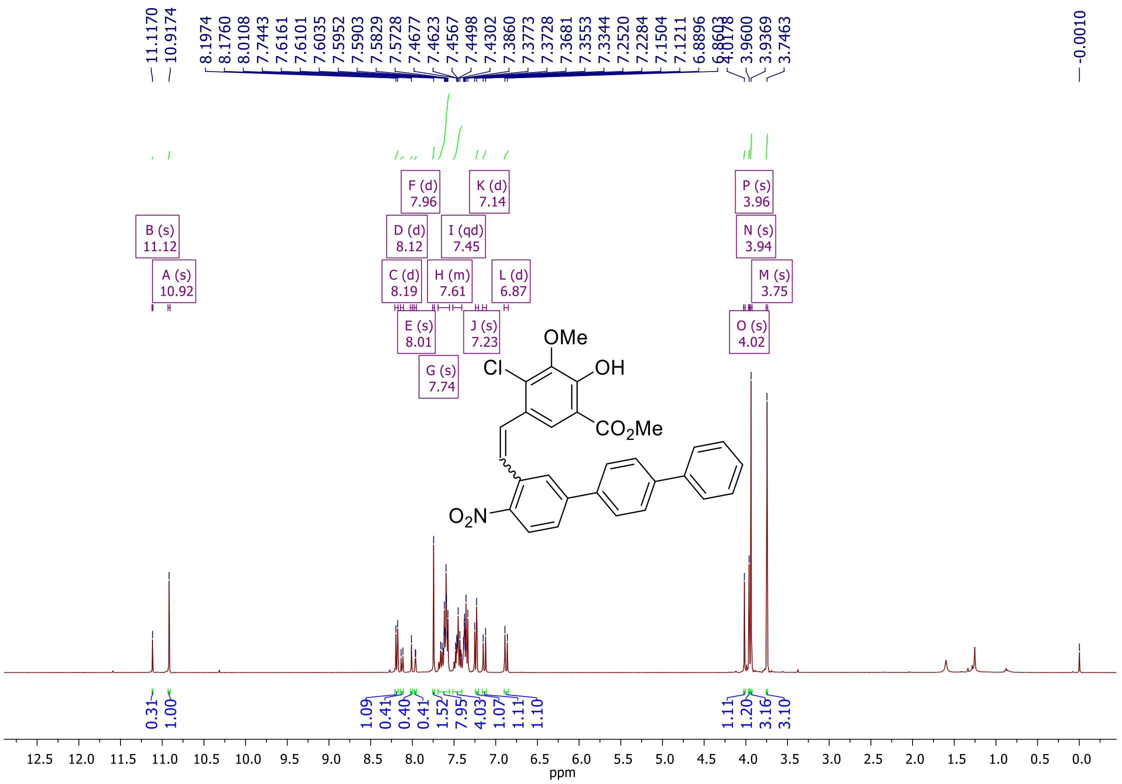

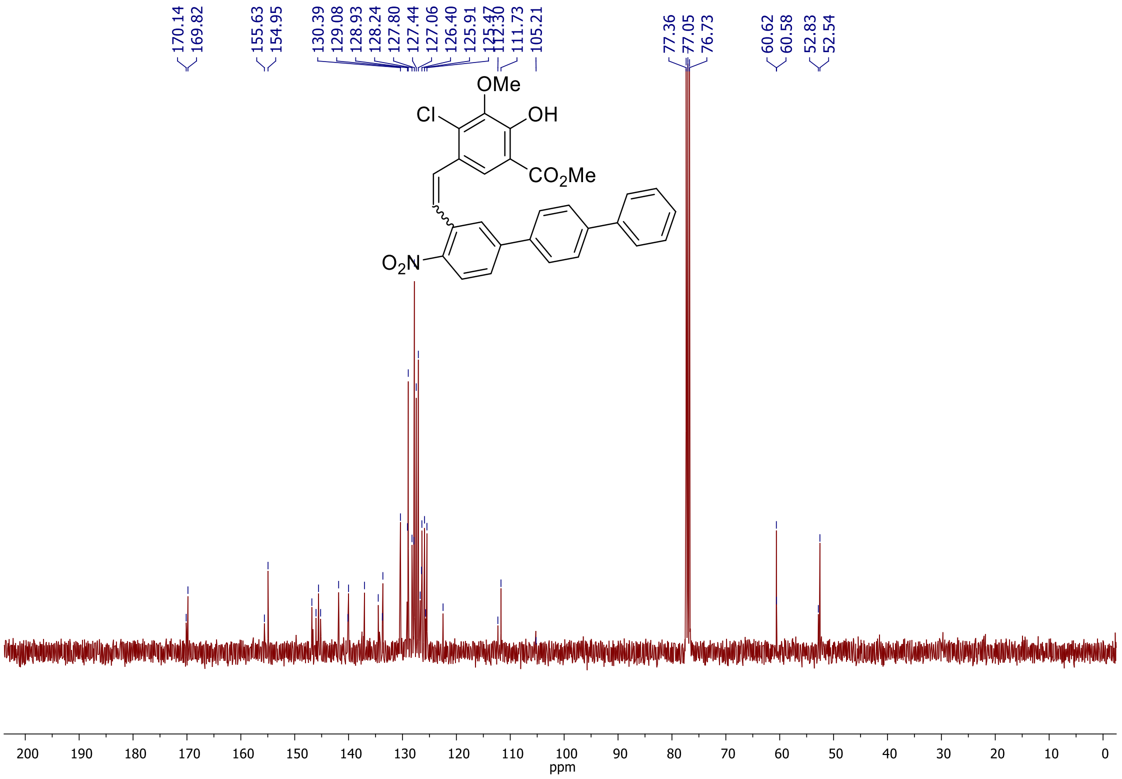

**$^{1}$H NMR (400 MHz, $CDCl_3$) and $^{13}$C NMR (100 MHz, $CDCl_3$) of 10h**

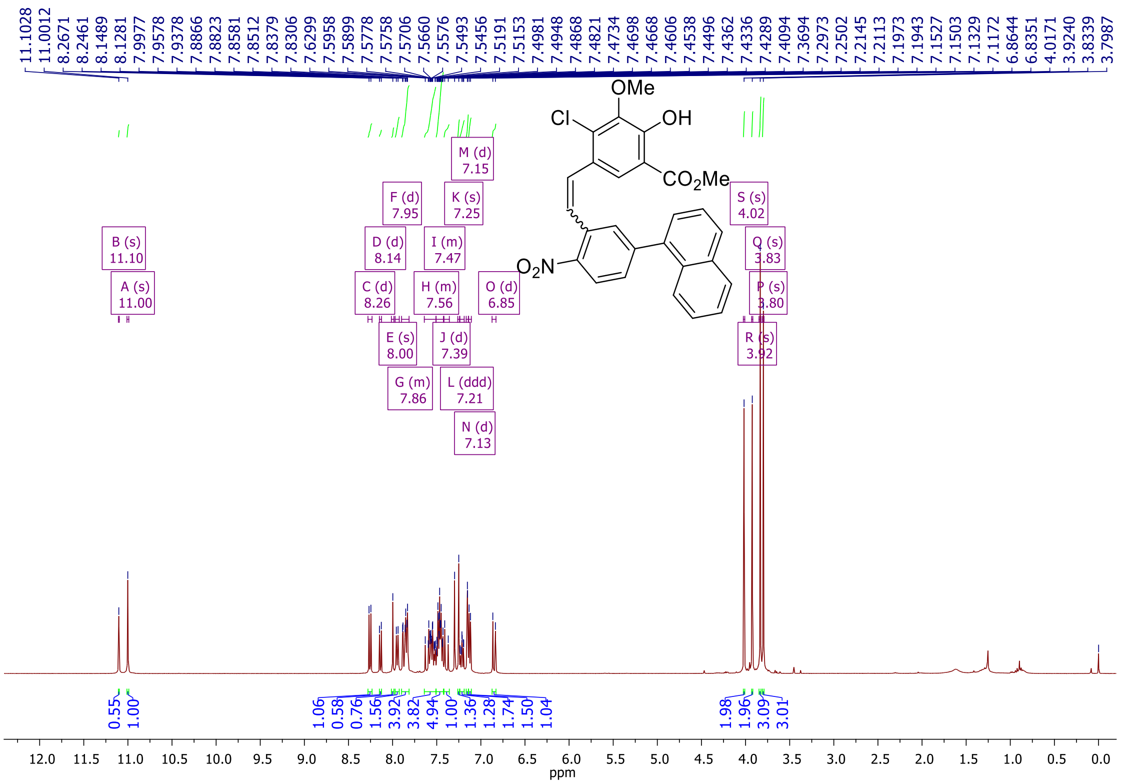

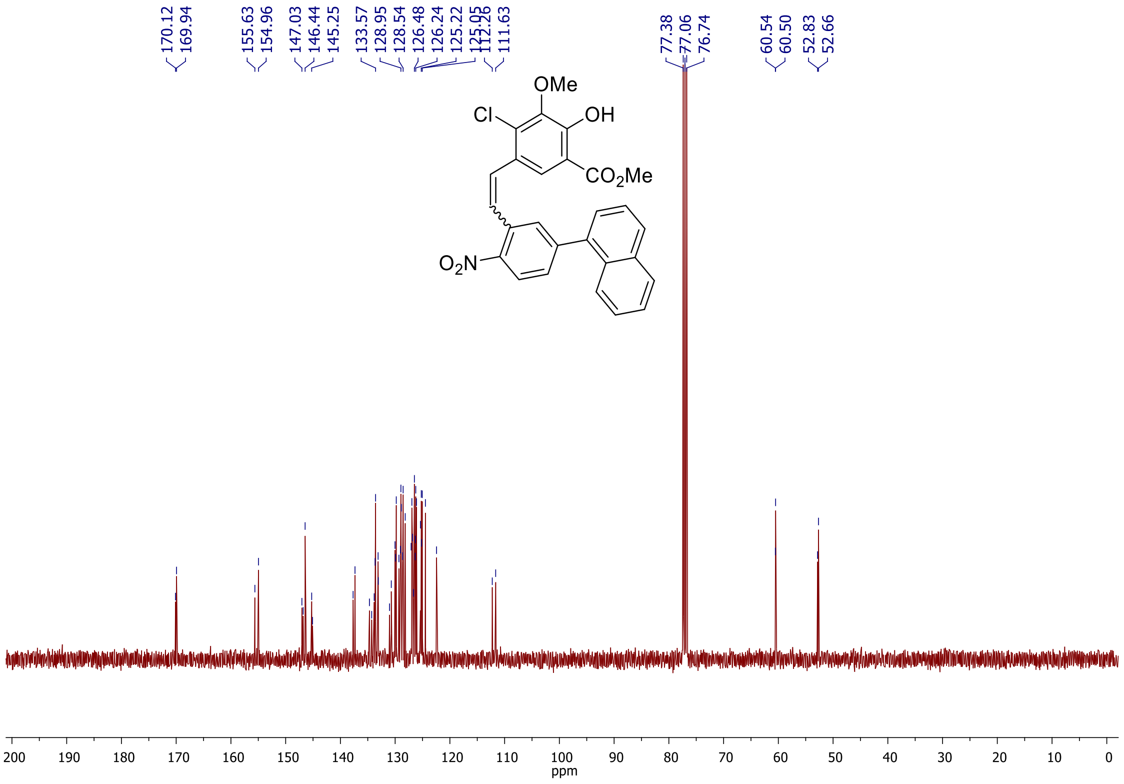

## $^1$H NMR (400 MHz, $CDCl_3$) and $^{13}$C NMR (100 MHz, $CDCl_3$) of 10i

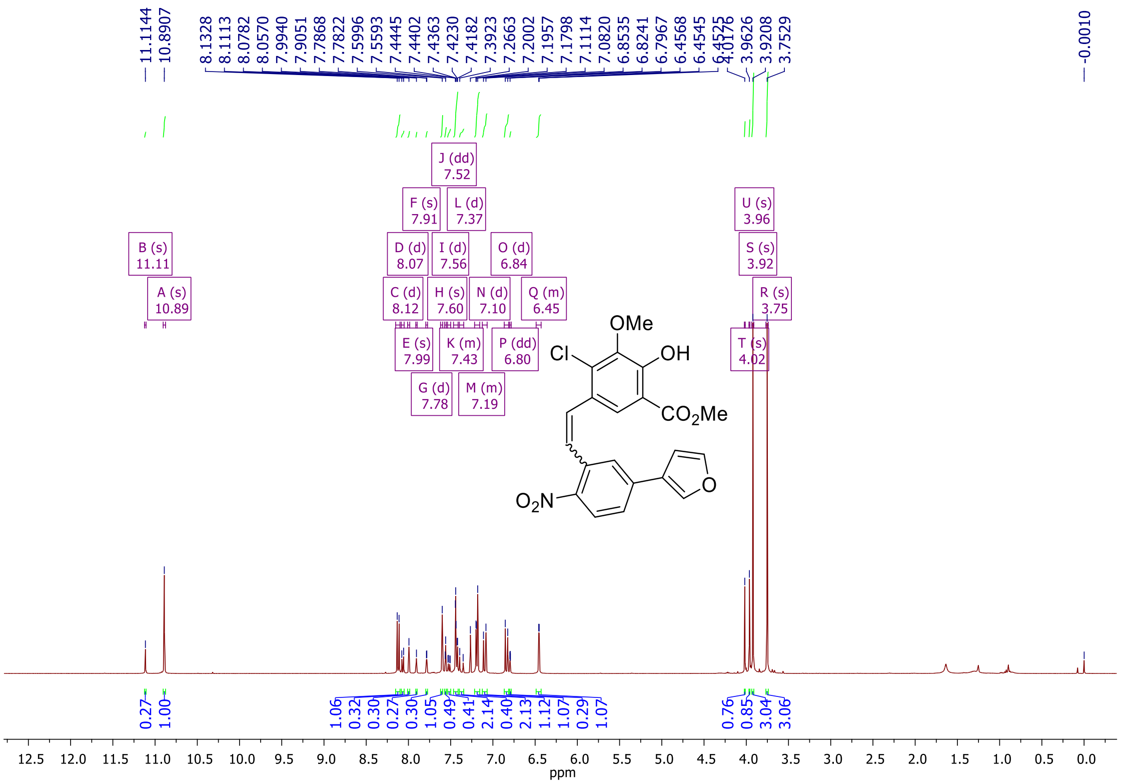

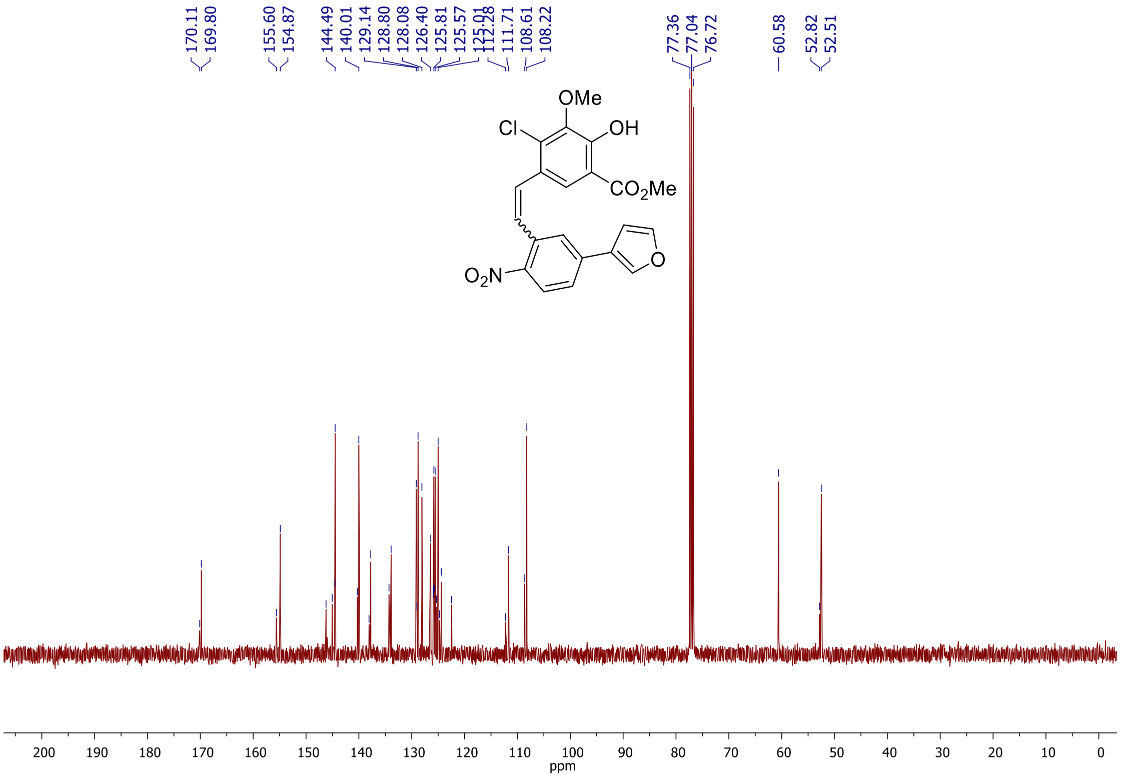

**$^{1}H$ NMR (400 MHz, $CDCl_3$) and $^{13}C$ NMR (100 MHz, $CDCl_3$) of 10j-OMe**

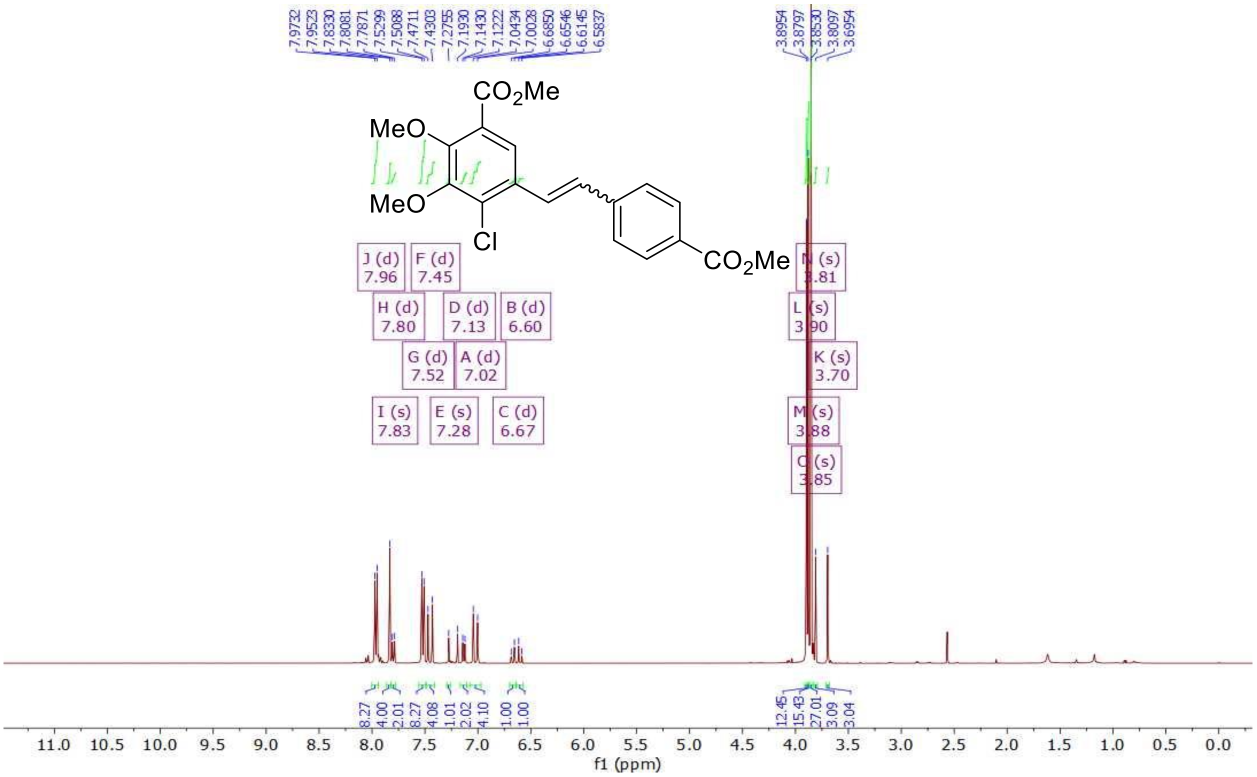

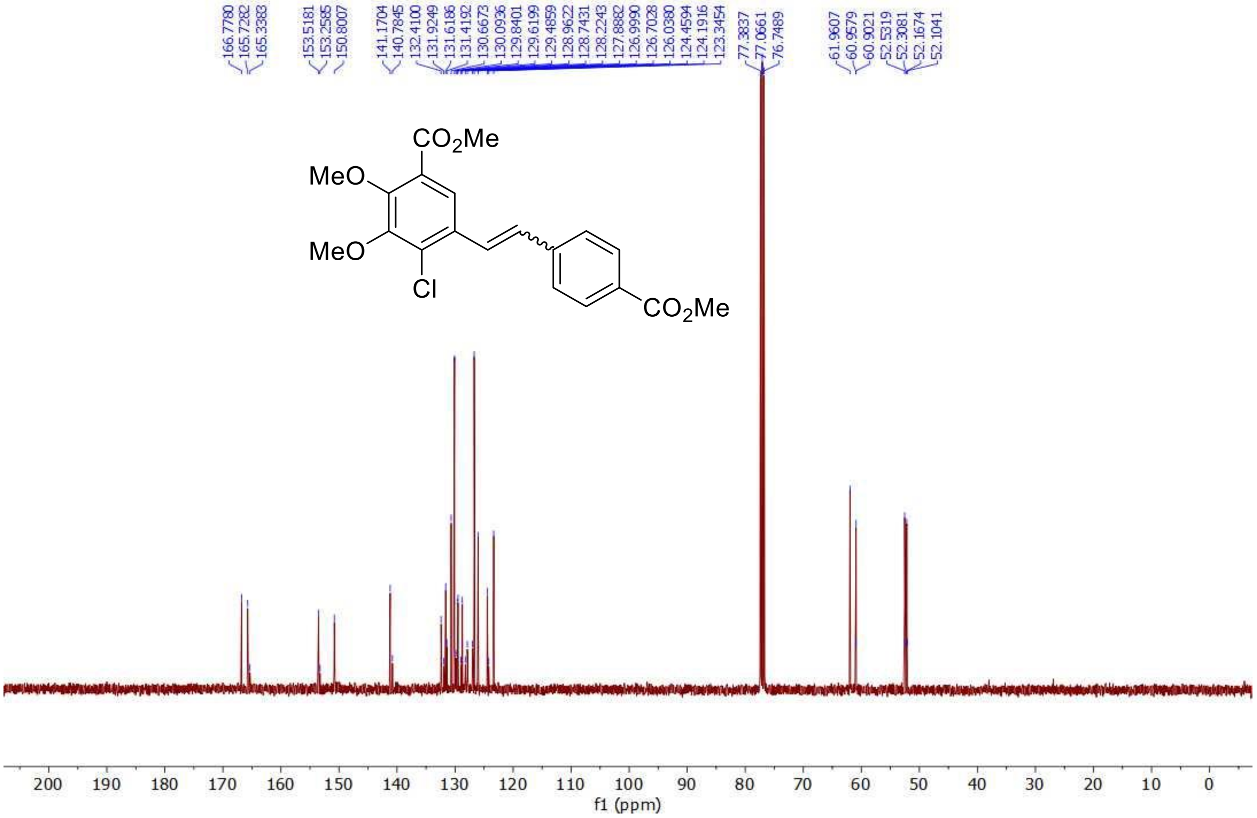

**$^{1}H$ NMR (400 MHz, $CDCl_3$) and $^{13}C$ NMR (100 MHz, $CDCl_3$) of *trans*-10k-OMe**

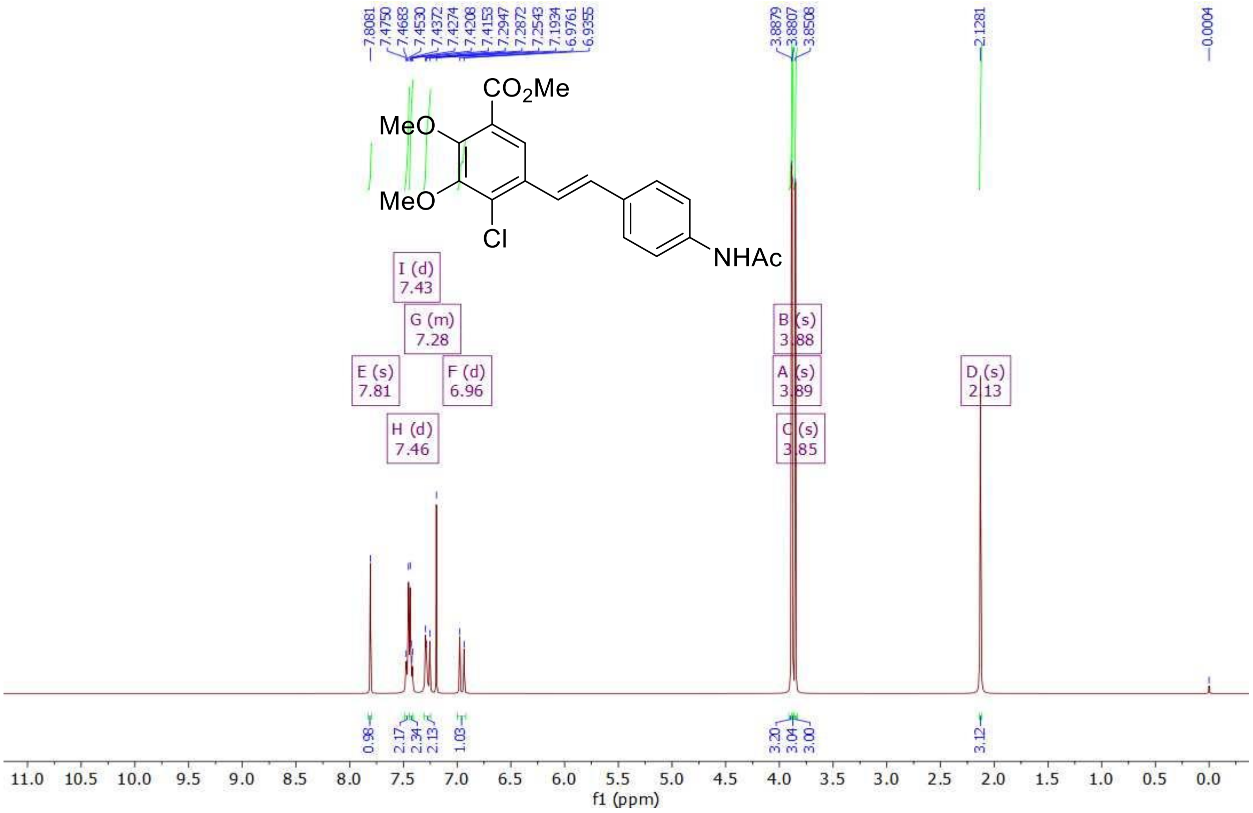


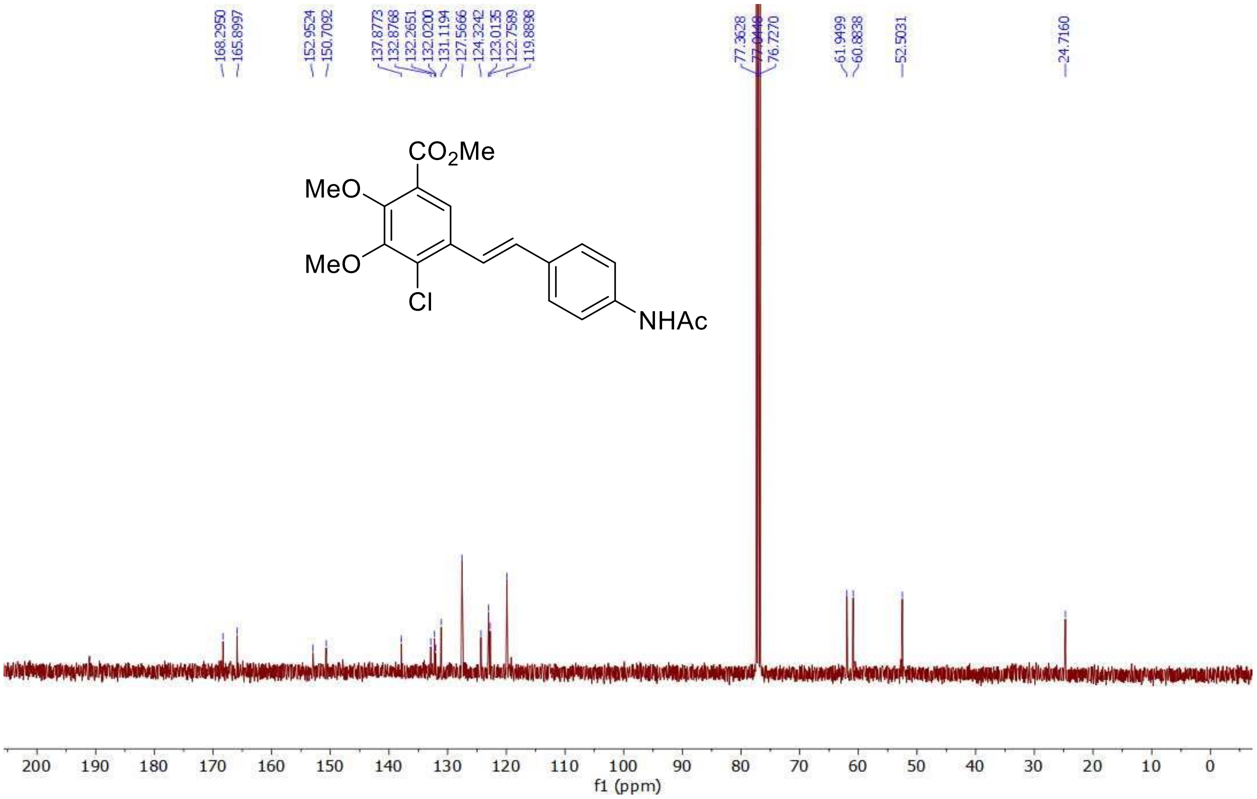

**$^1$H NMR (400 MHz, CDCl$_3$) and $^{13}$C NMR (100 MHz, CDCl$_3$) of 10l**

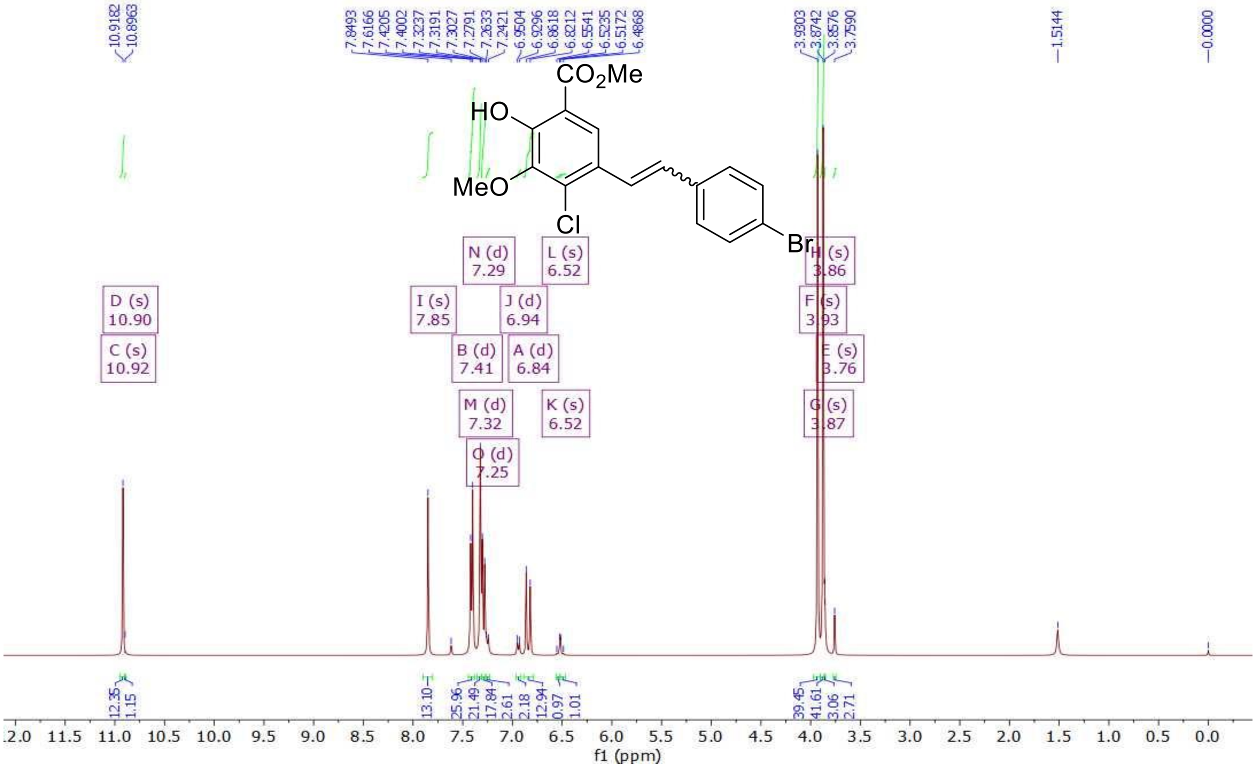

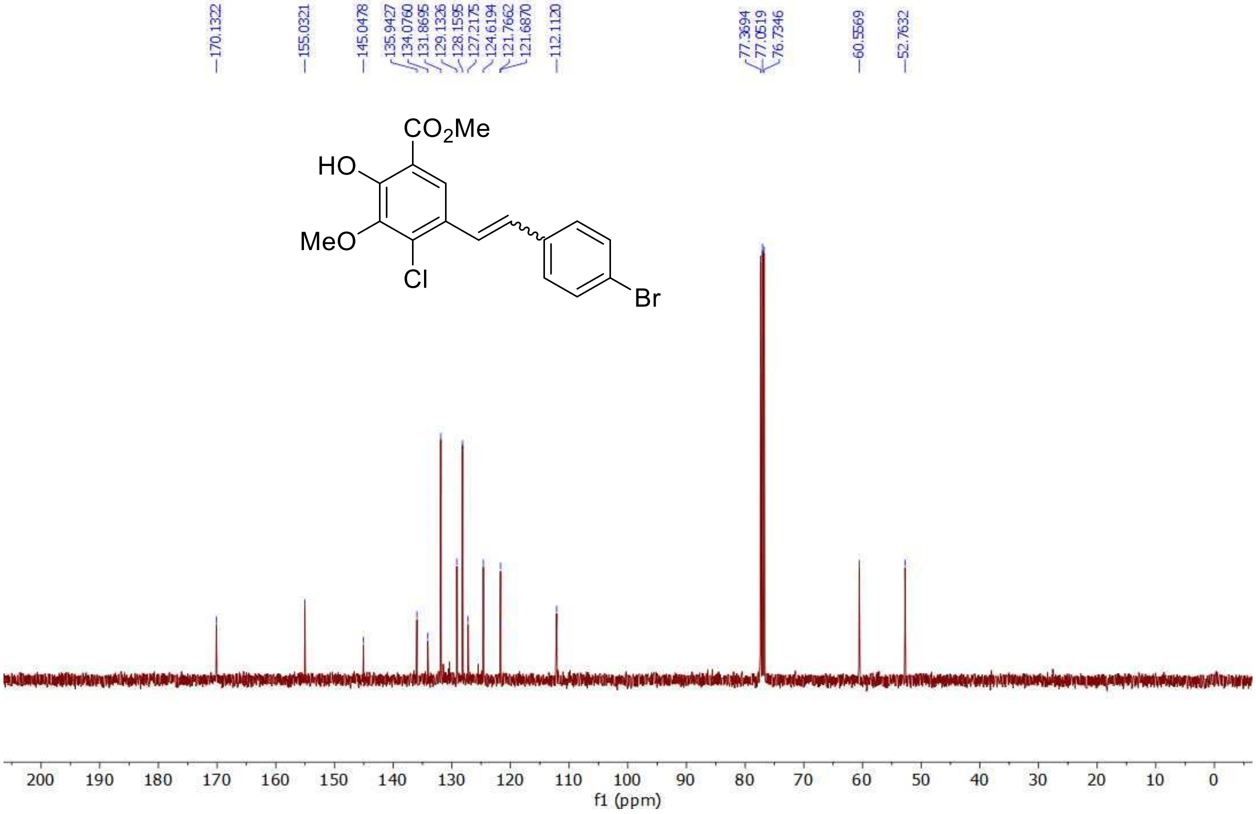

**$^{1}$H NMR (400 MHz, $CDCl_3$) and $^{13}$C NMR (100 MHz, $CDCl_3$) of 10m**

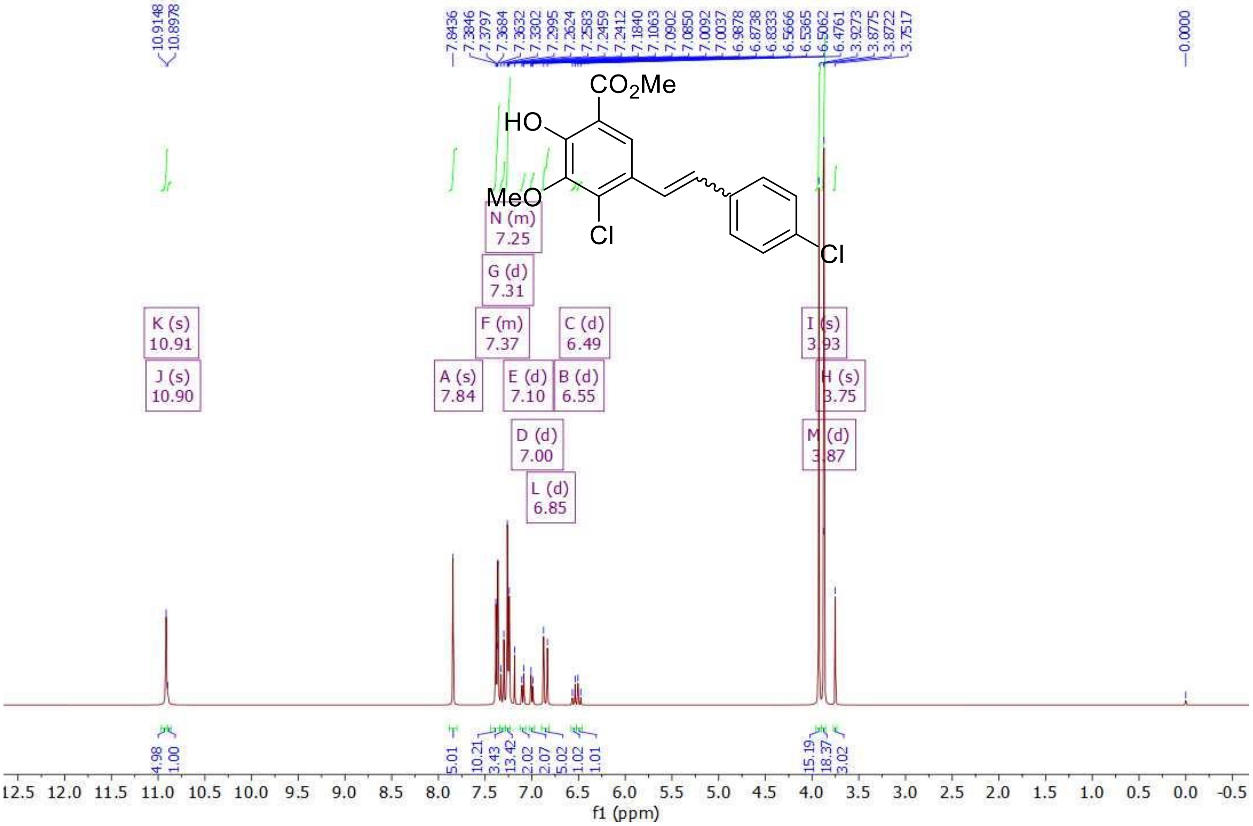


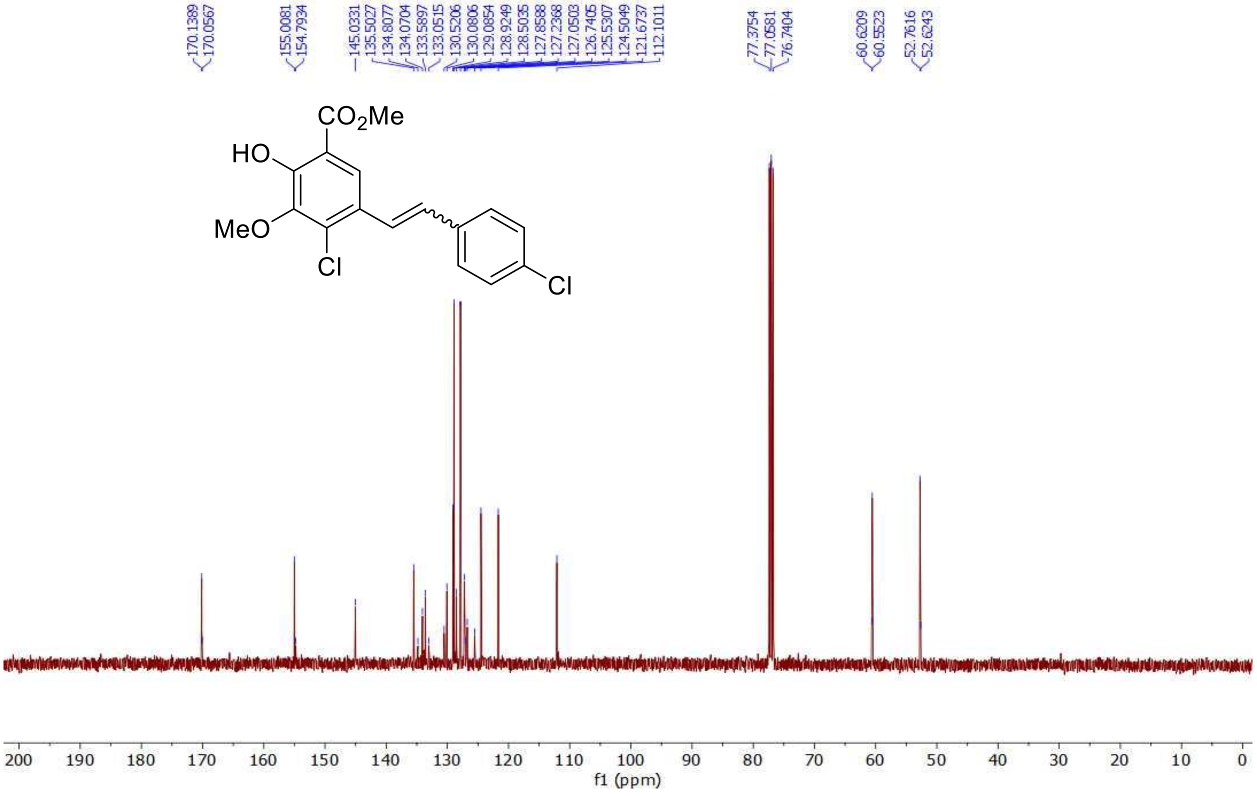

**$^{1}$H NMR (400 MHz, CDCl$_3$) and $^{13}$C NMR (100 MHz, CDCl$_3$) of 10n**

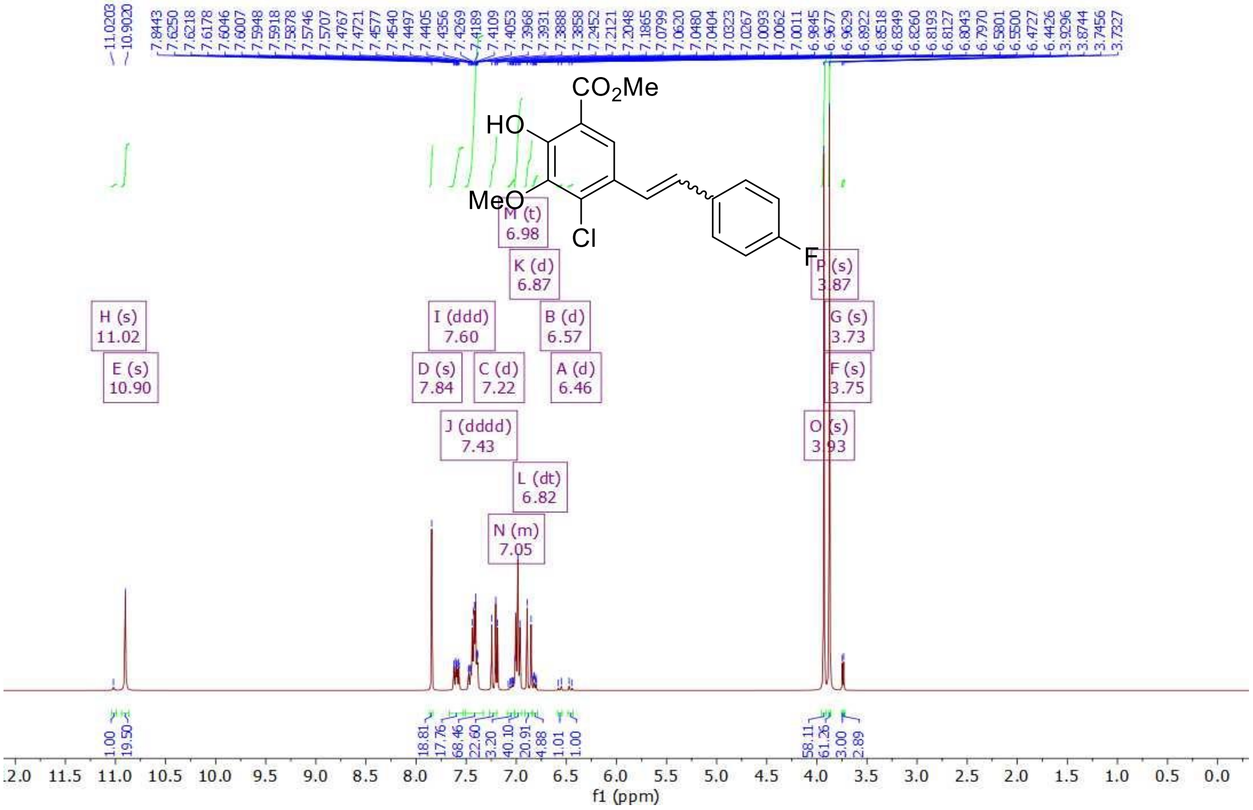

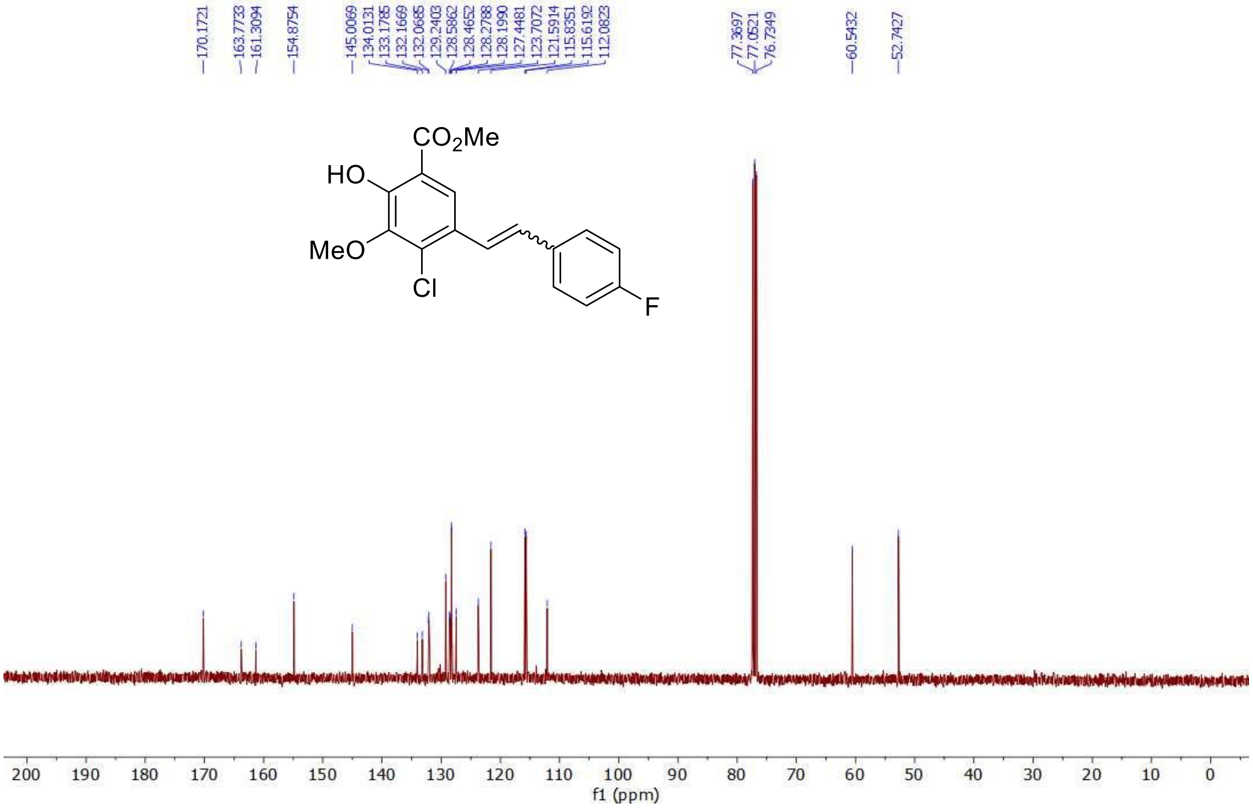

**$^{1}$H NMR (400 MHz, $CDCl_3$) and $^{13}$C NMR (100 MHz, $CDCl_3$) of 10o**

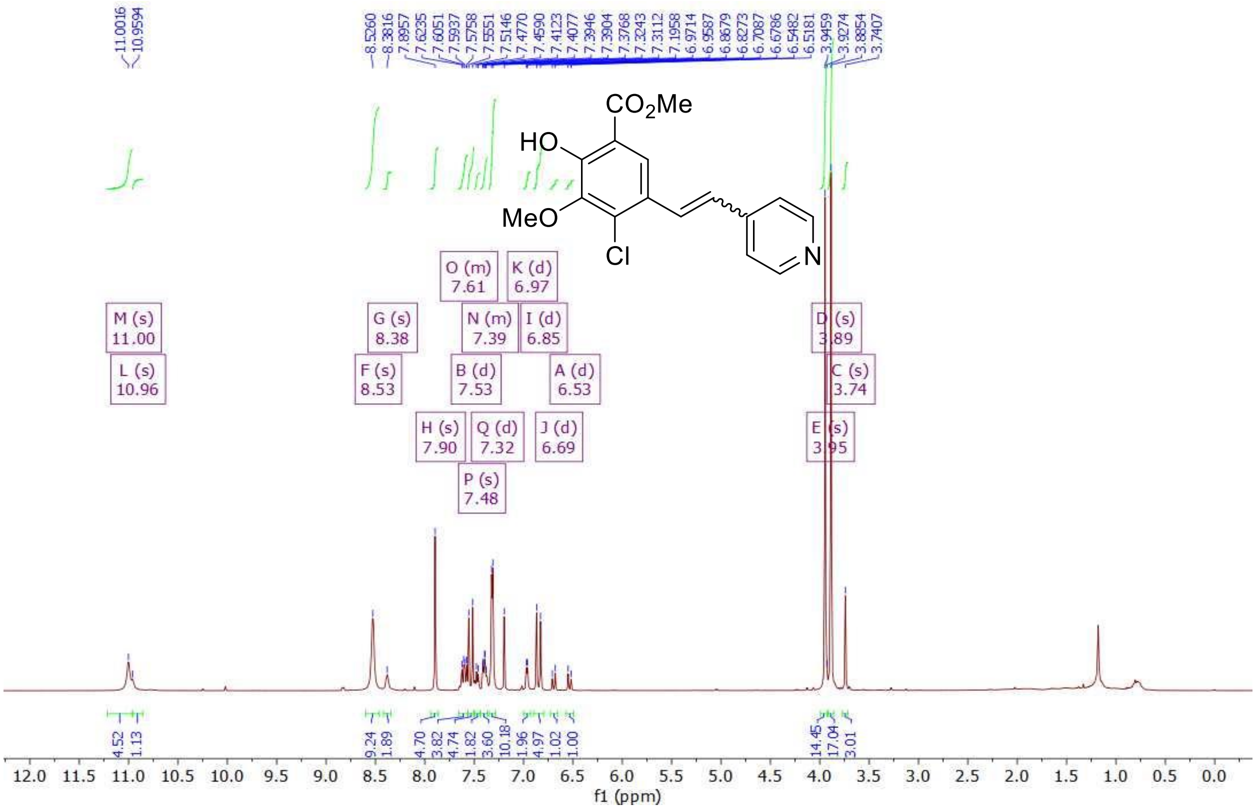


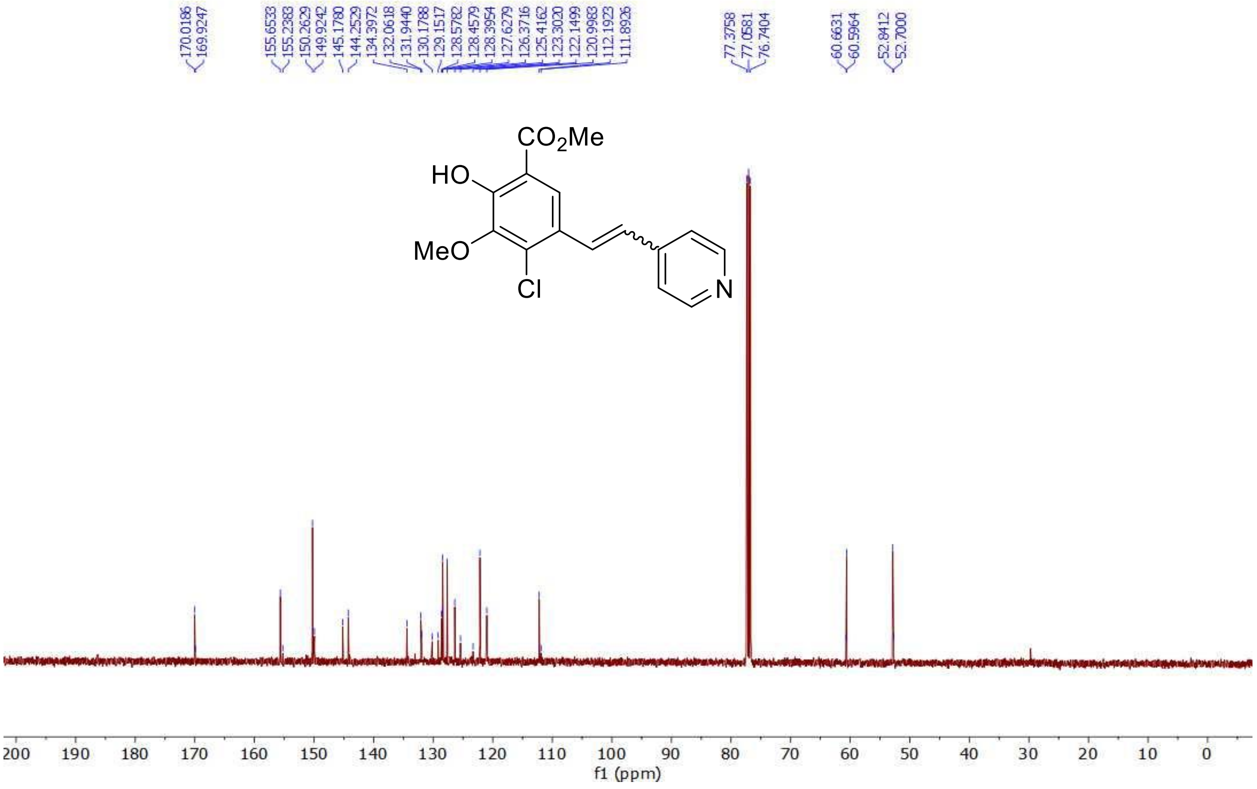

**$^{1}$H NMR (400 MHz, $CDCl_3$) and $^{13}$C NMR (100 MHz, $CDCl_3$) of 10p**

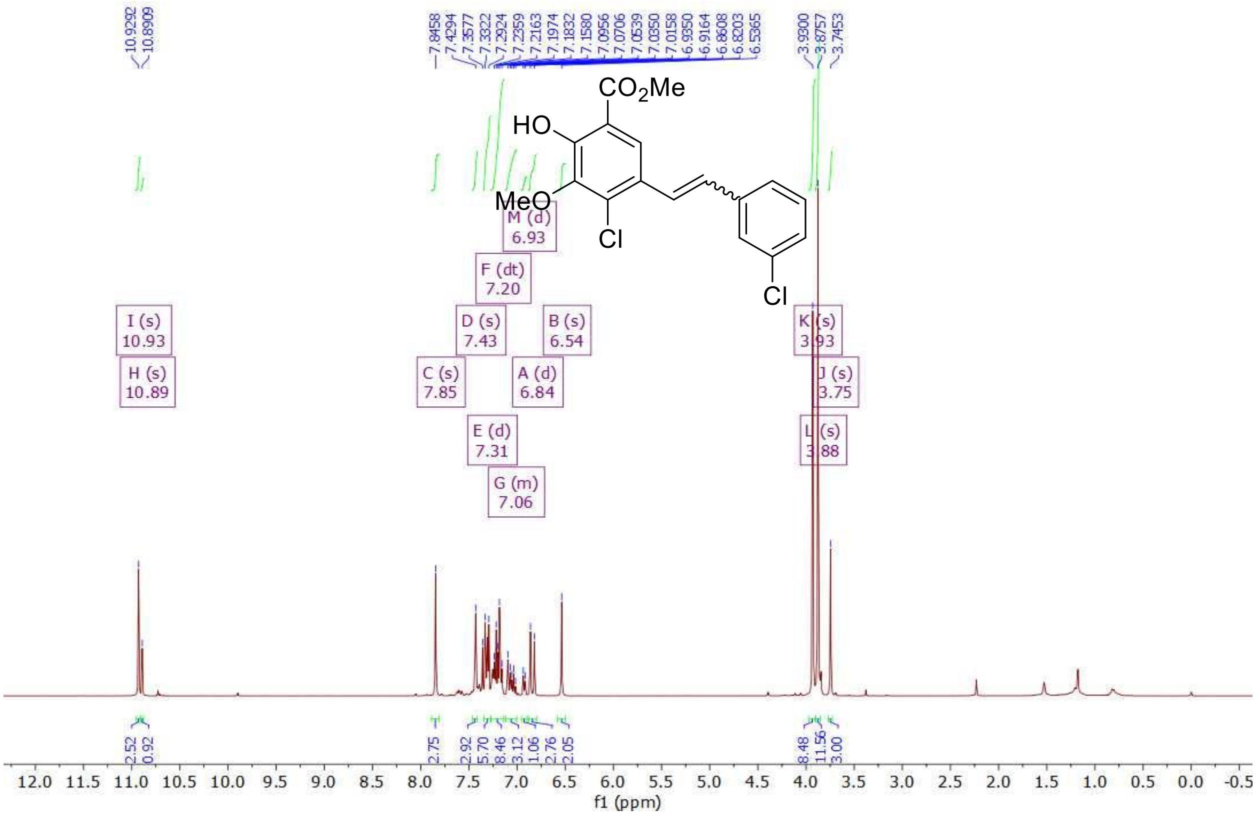

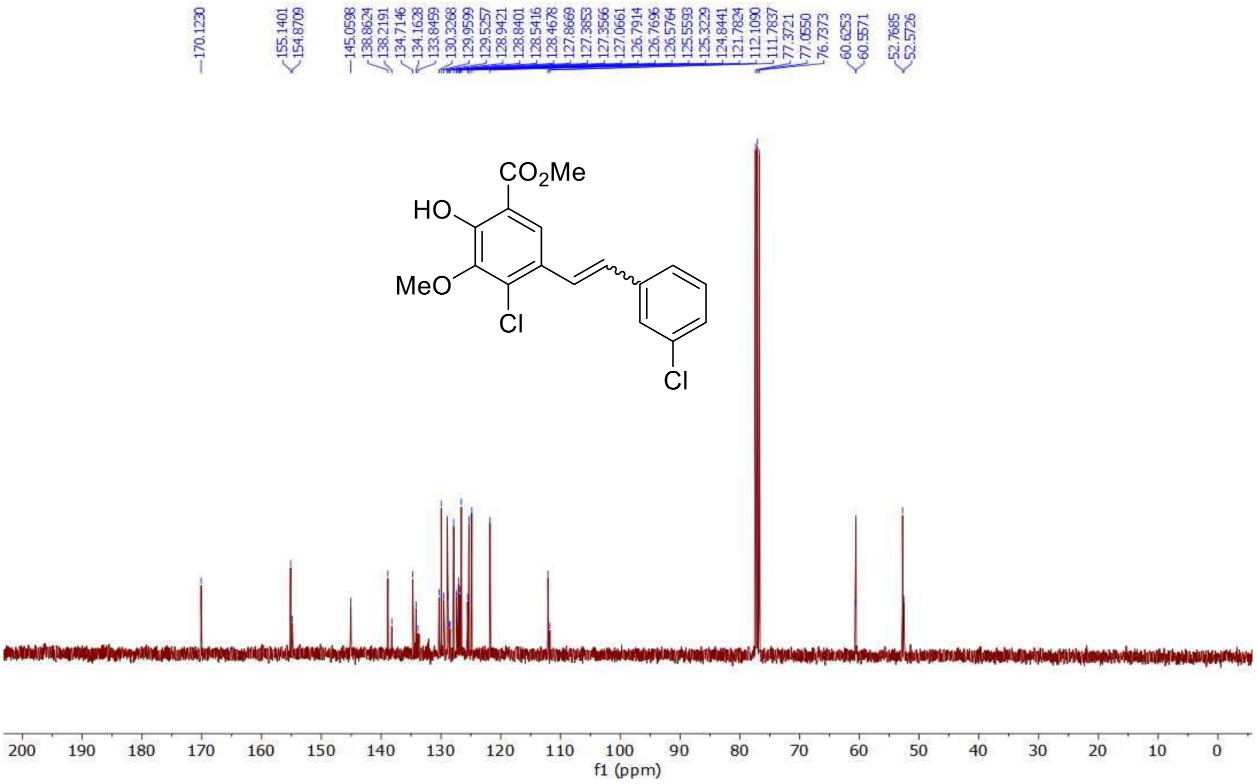

**$^{1}$H NMR (400 MHz, $CDCl_3$) and $^{13}$C NMR (100 MHz, $CDCl_3$) of 10q**

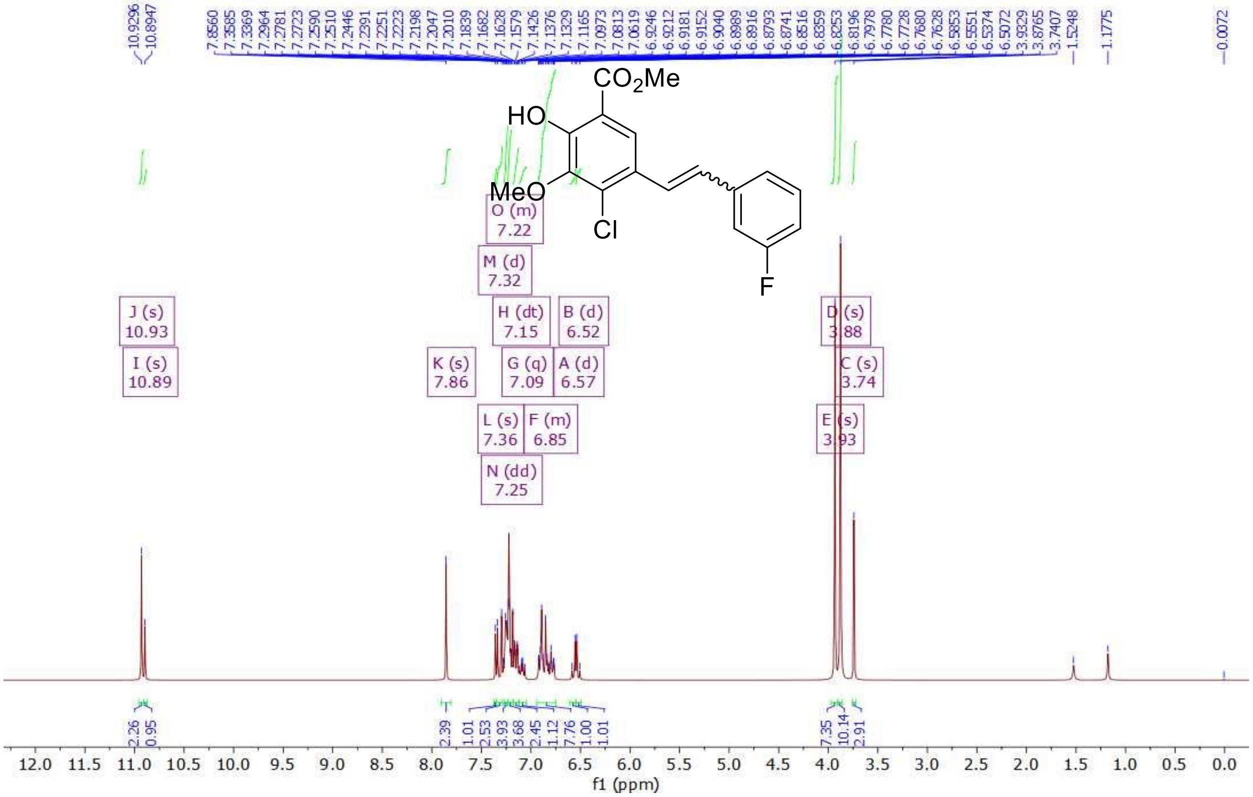


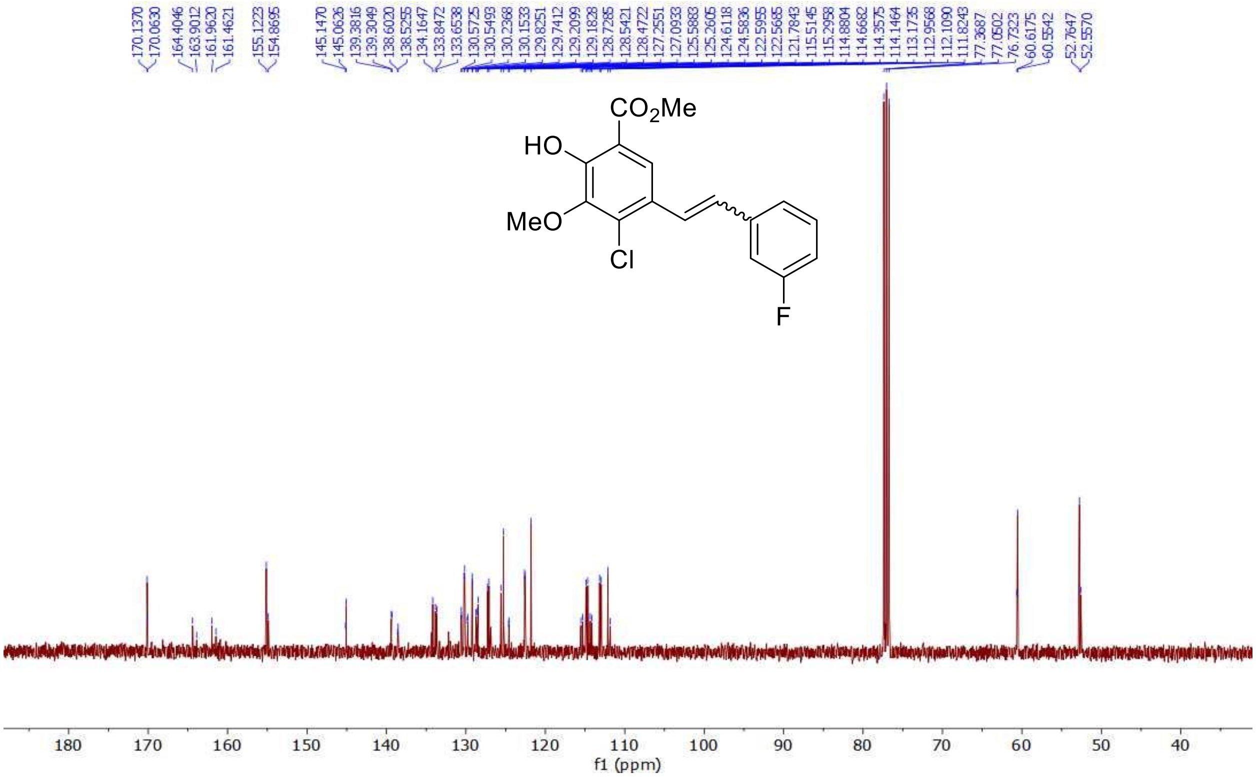

## $^{1}H$ NMR (400 MHz, $CDCl_3$) and $^{13}C$ NMR (100 MHz, $CDCl_3$) of 10r

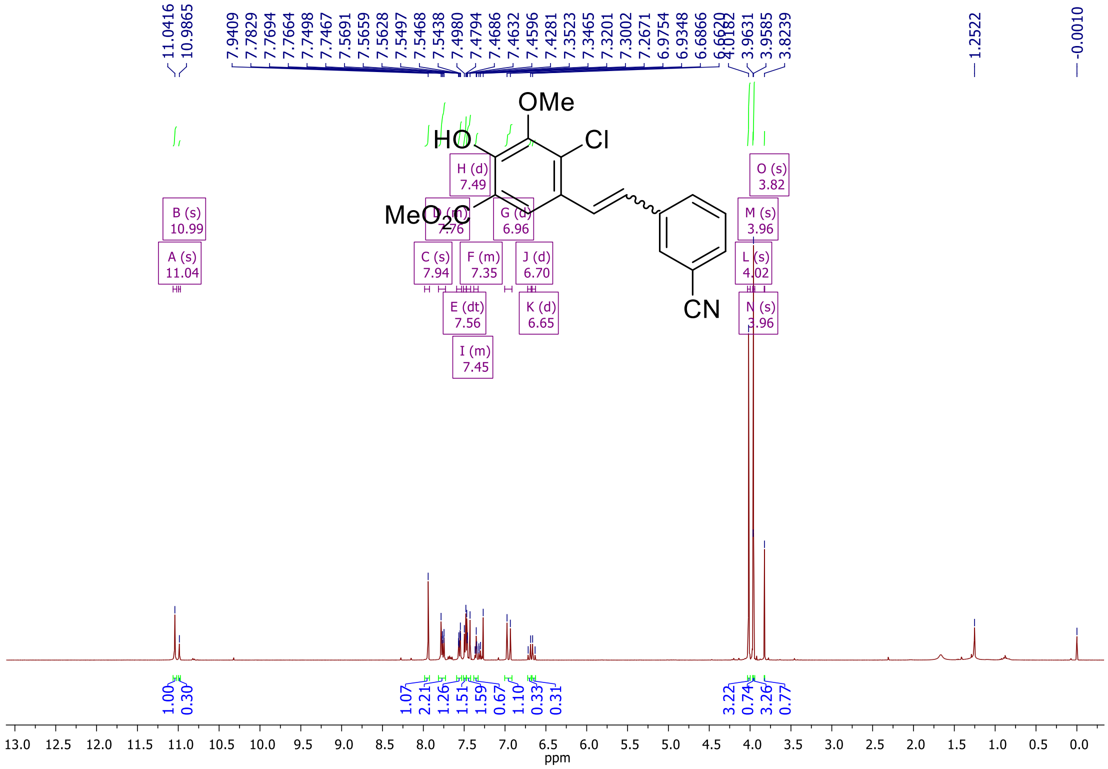

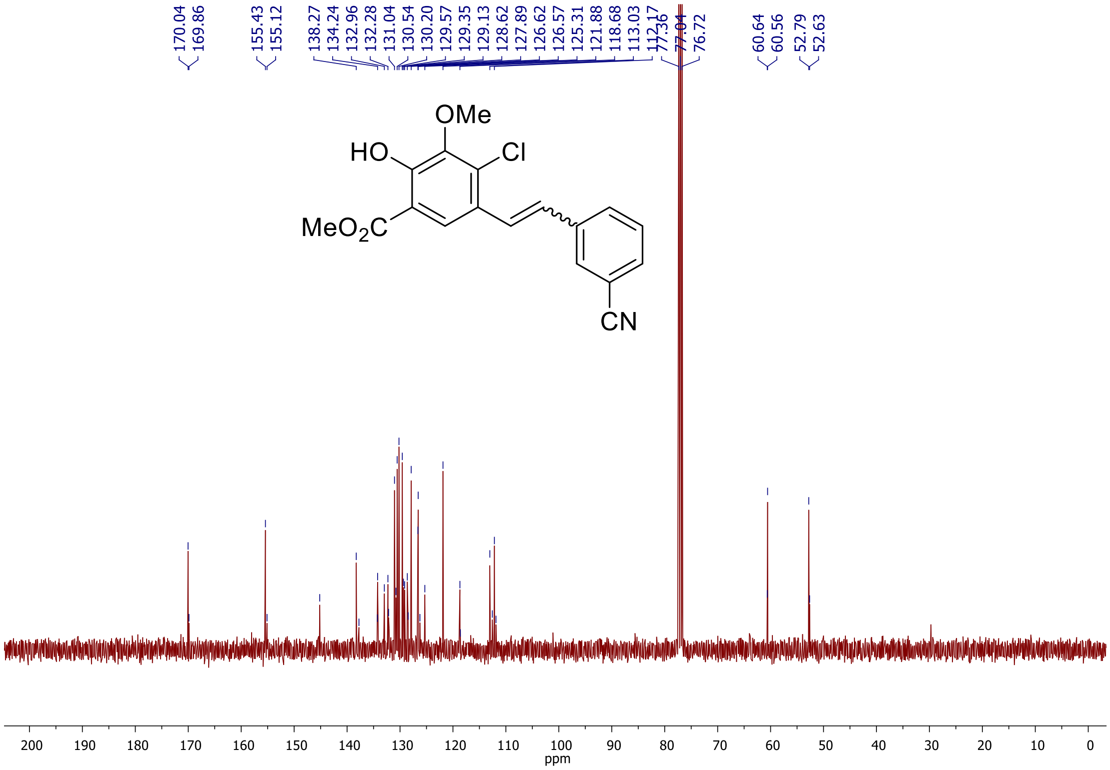

**$^{1}$H NMR (400 MHz, $CDCl_3$) and $^{13}$C NMR (100 MHz, $CDCl_3$) of 10s**

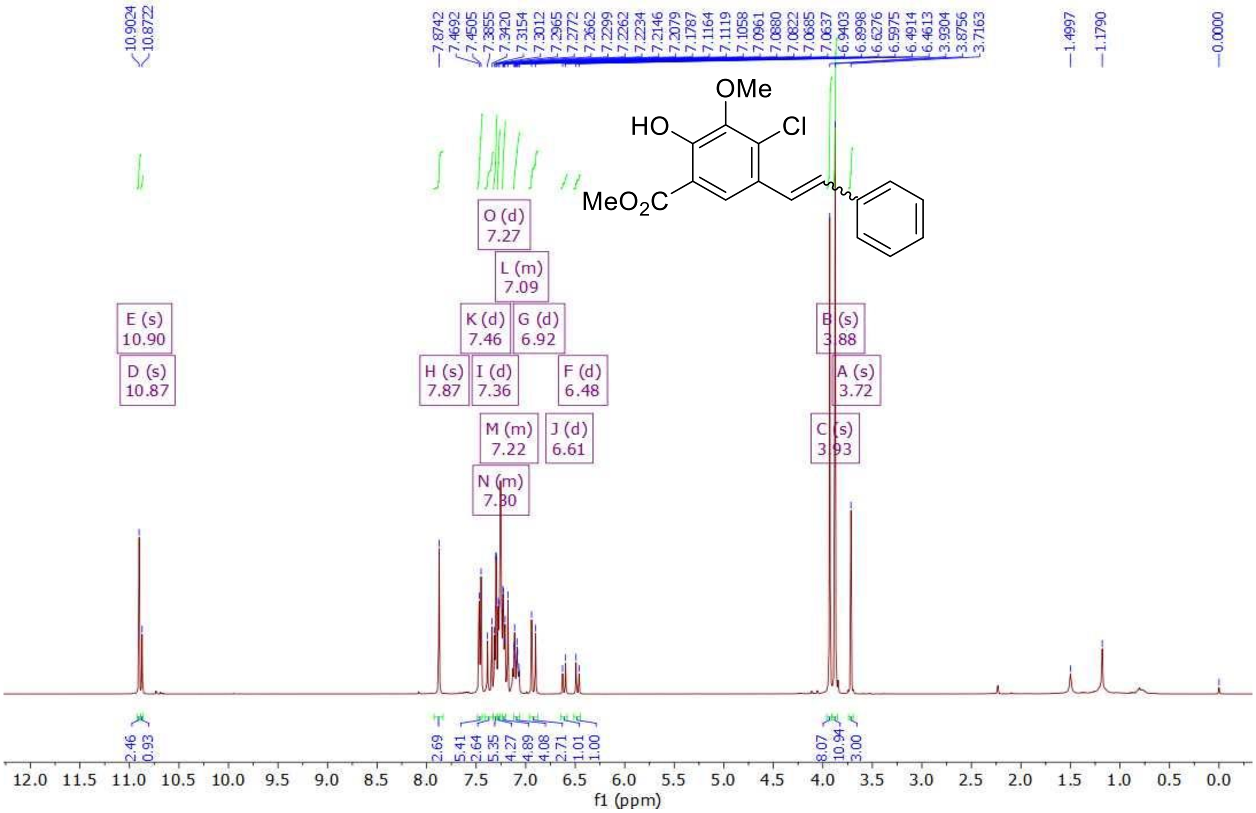


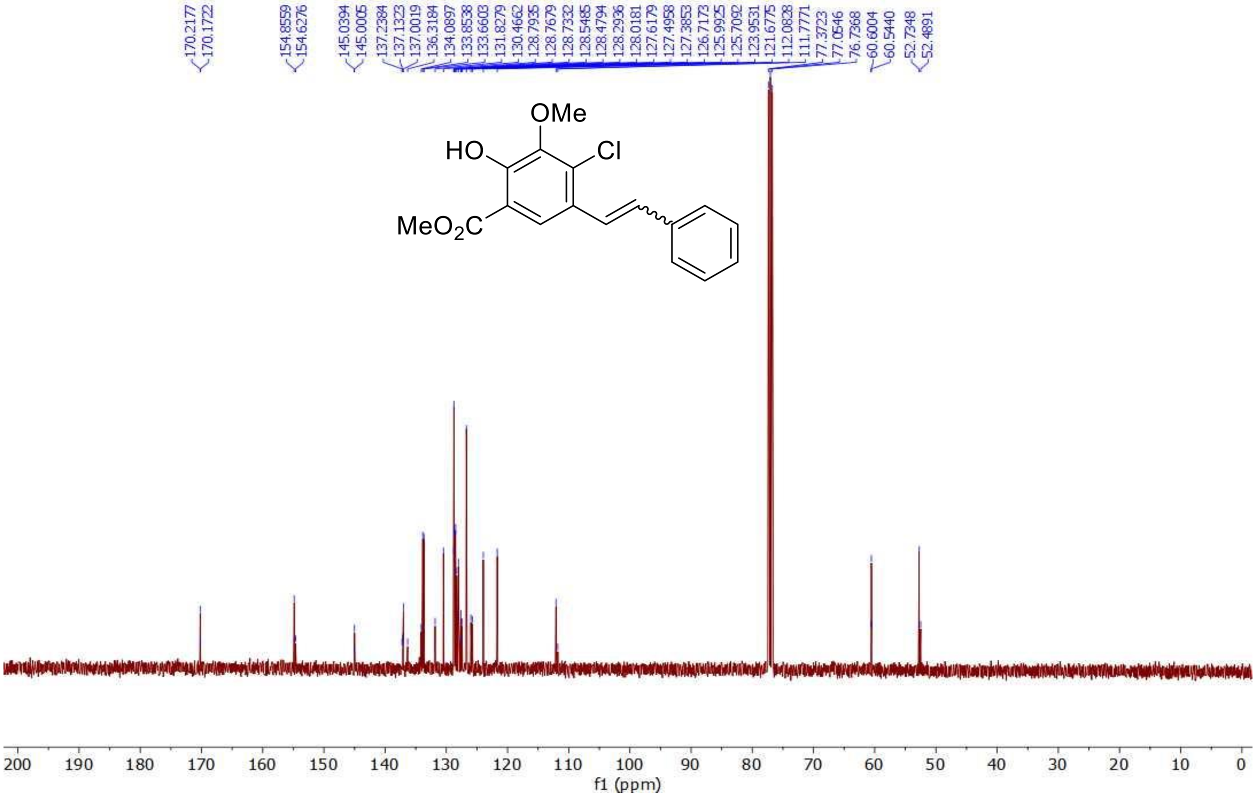

**$^1$H NMR (400 MHz, CDCl$_3$) and $^{13}$C NMR (100 MHz, CDCl$_3$) of 10u**

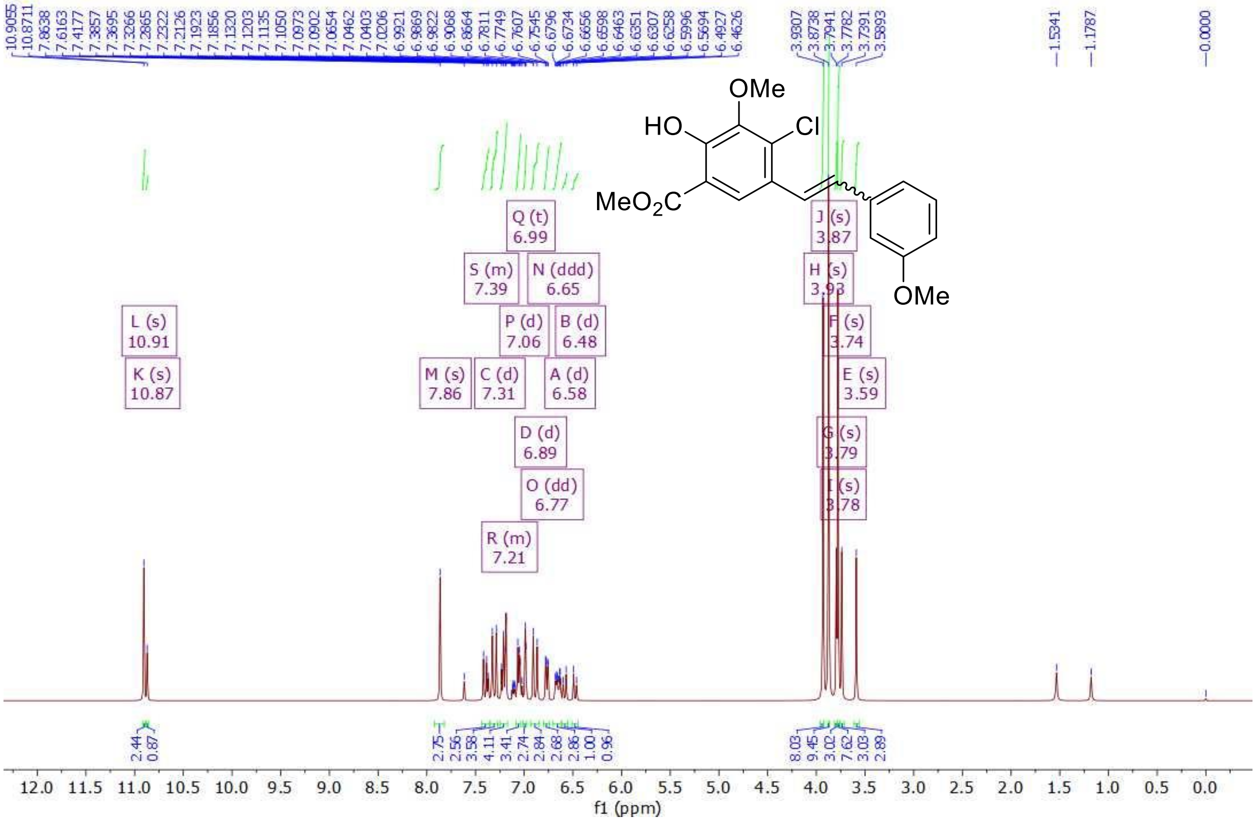

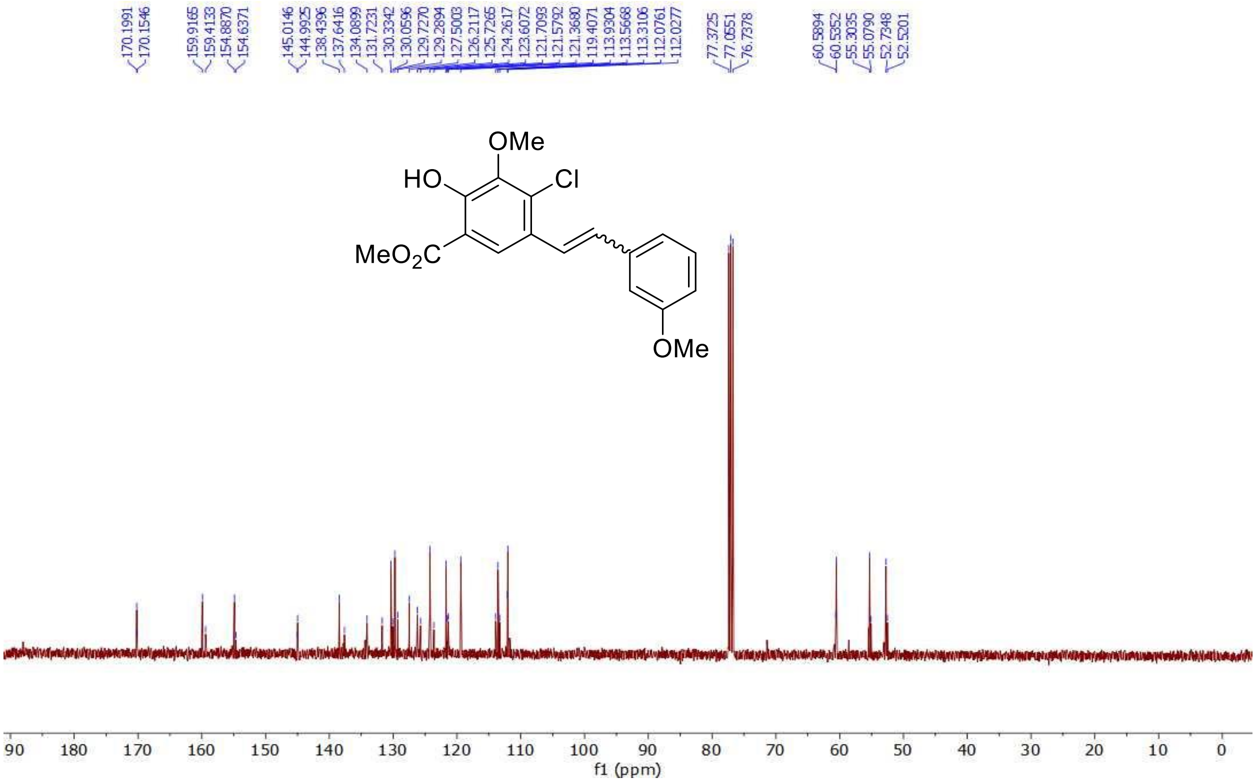

**$^{1}$H NMR (400 MHz, $CDCl_3$) and $^{13}$C NMR (100 MHz, $CDCl_3$) of 10v**

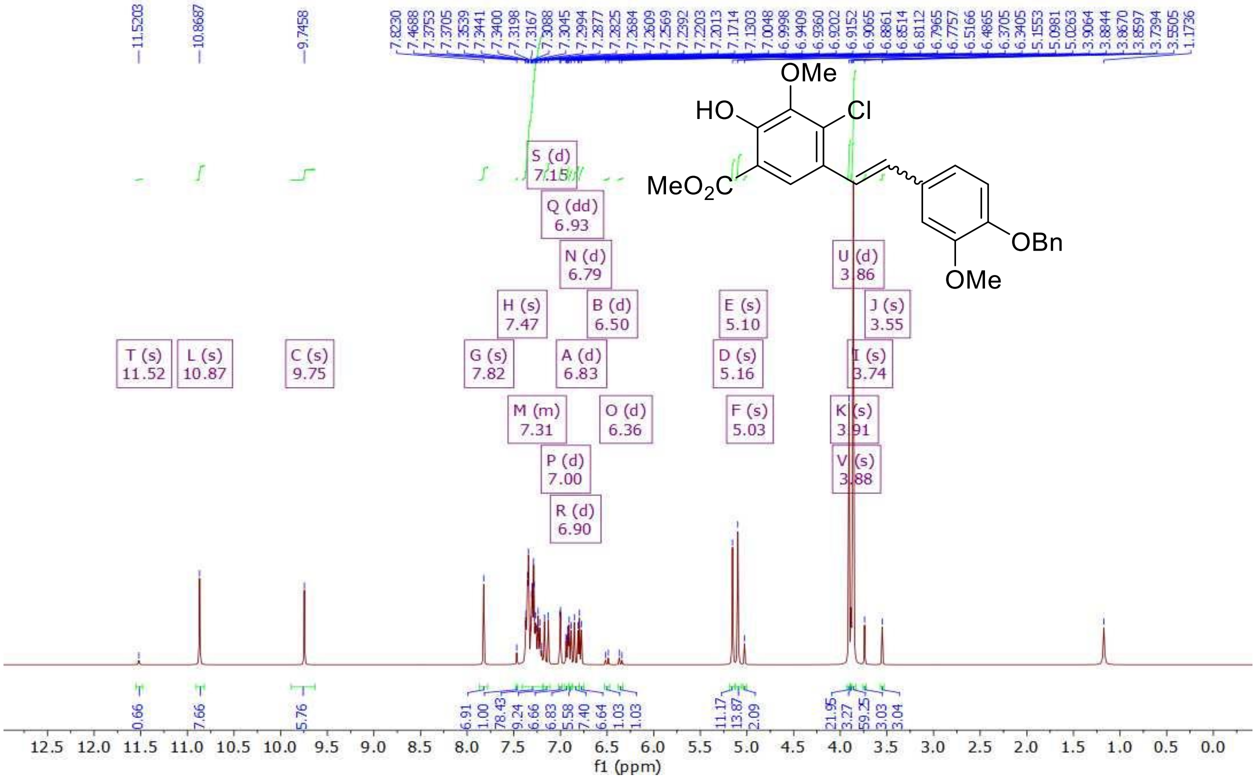

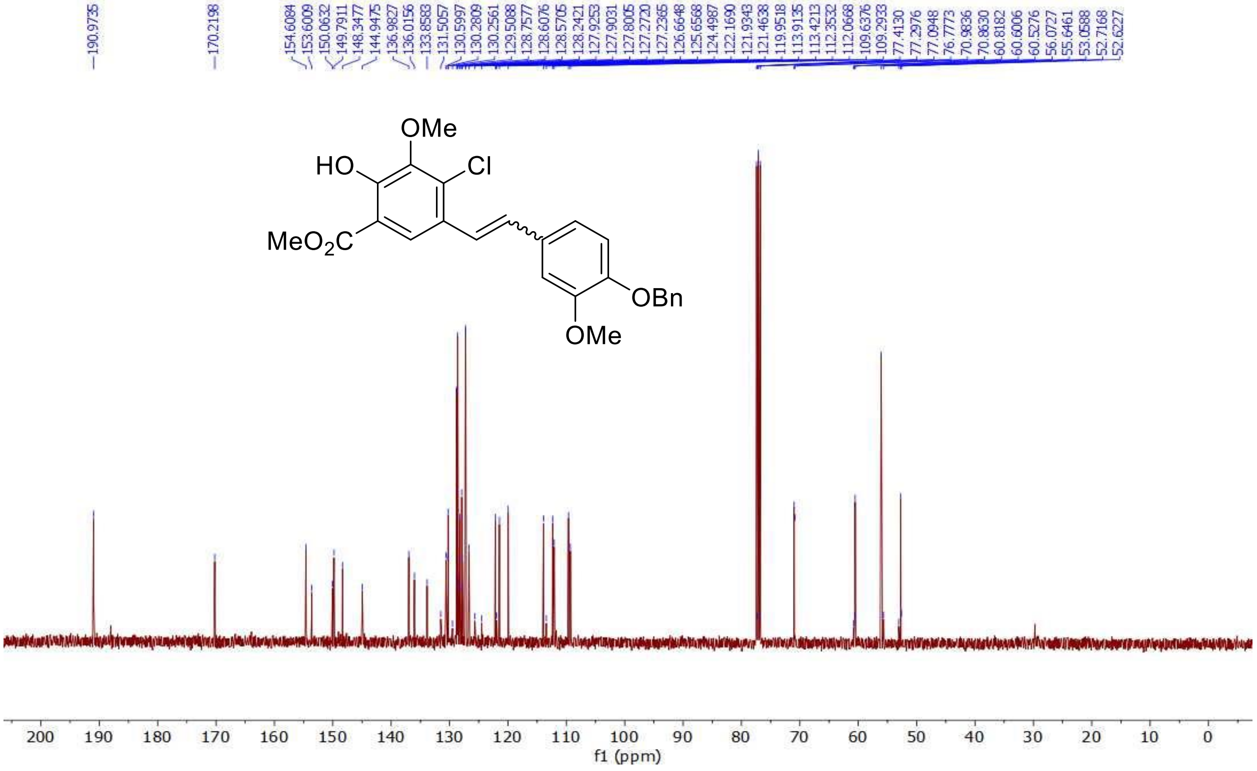

**$^{1}$H NMR (400 MHz, CDCl3) and $^{13}$C NMR (100 MHz, CDCl3) of *trans*-10v-OMe**

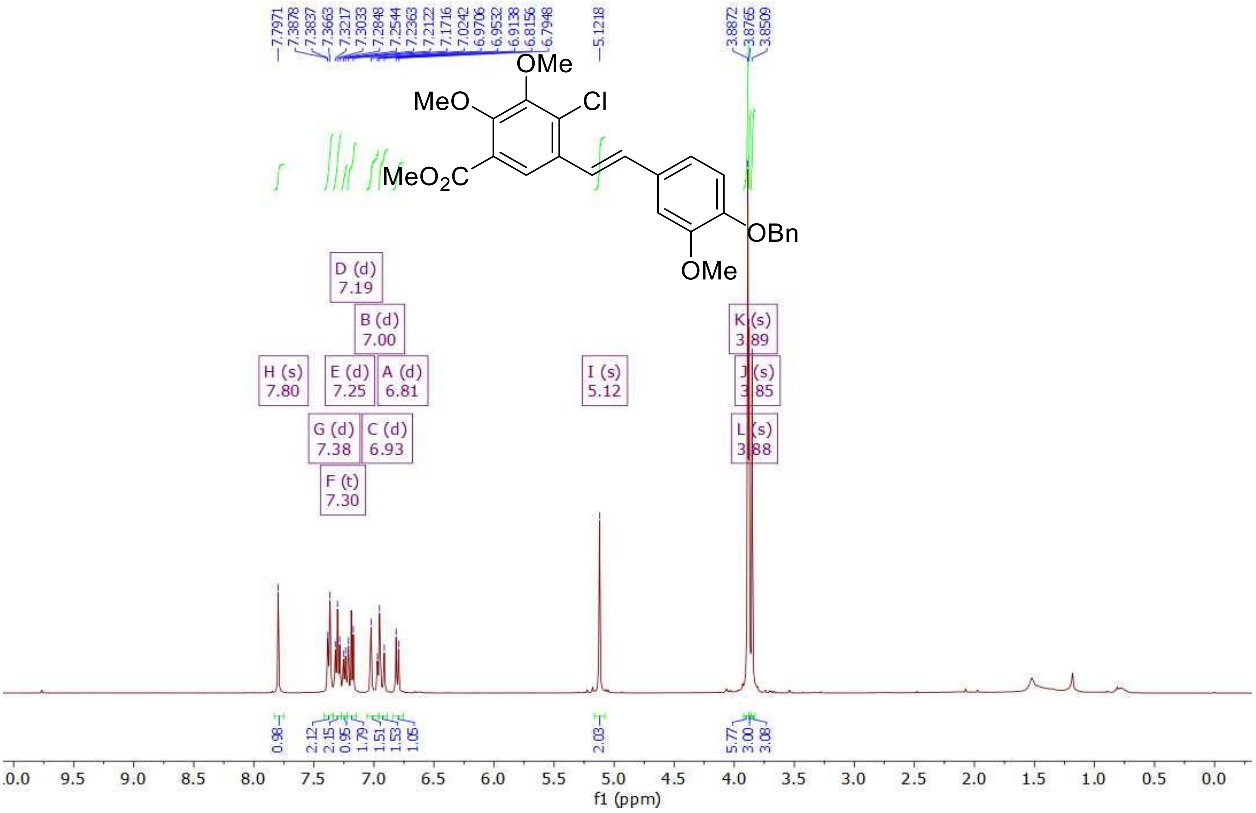

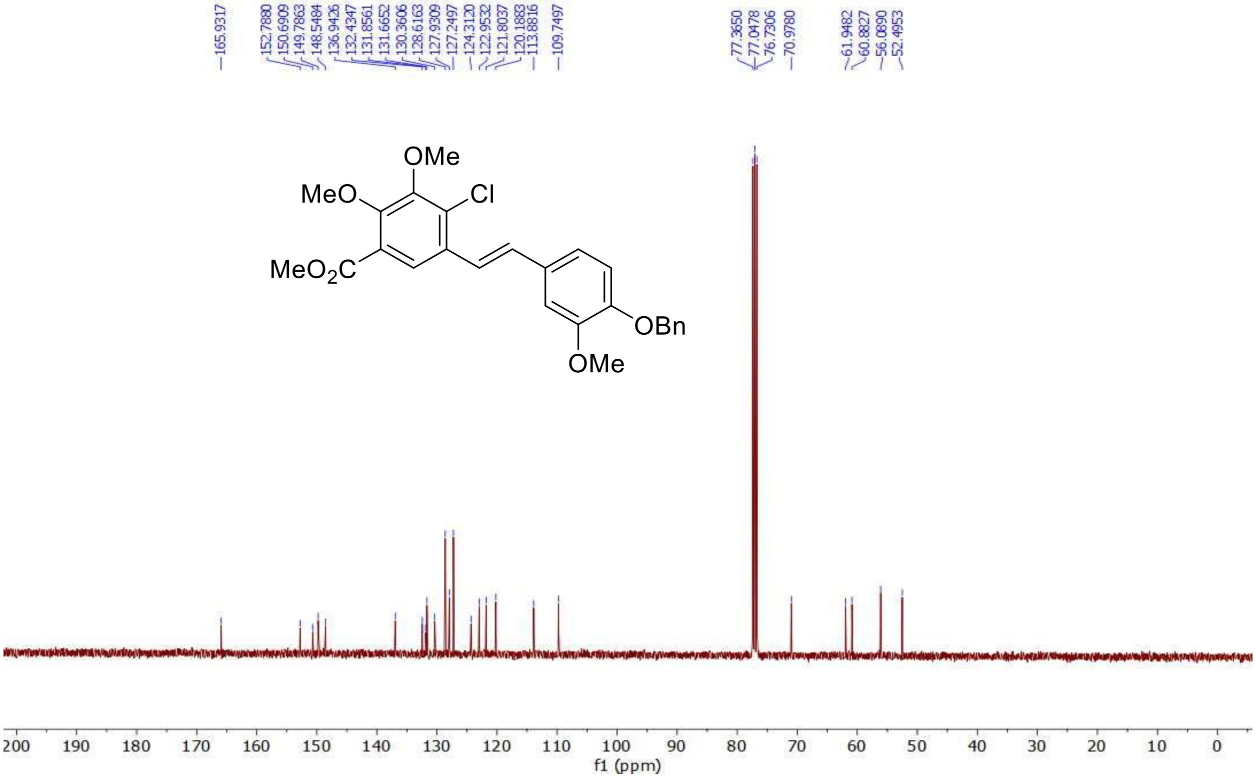

**$^{1}$H NMR (400 MHz, $CDCl_3$) and $^{13}$C NMR (100 MHz, $CDCl_3$) of 10w**

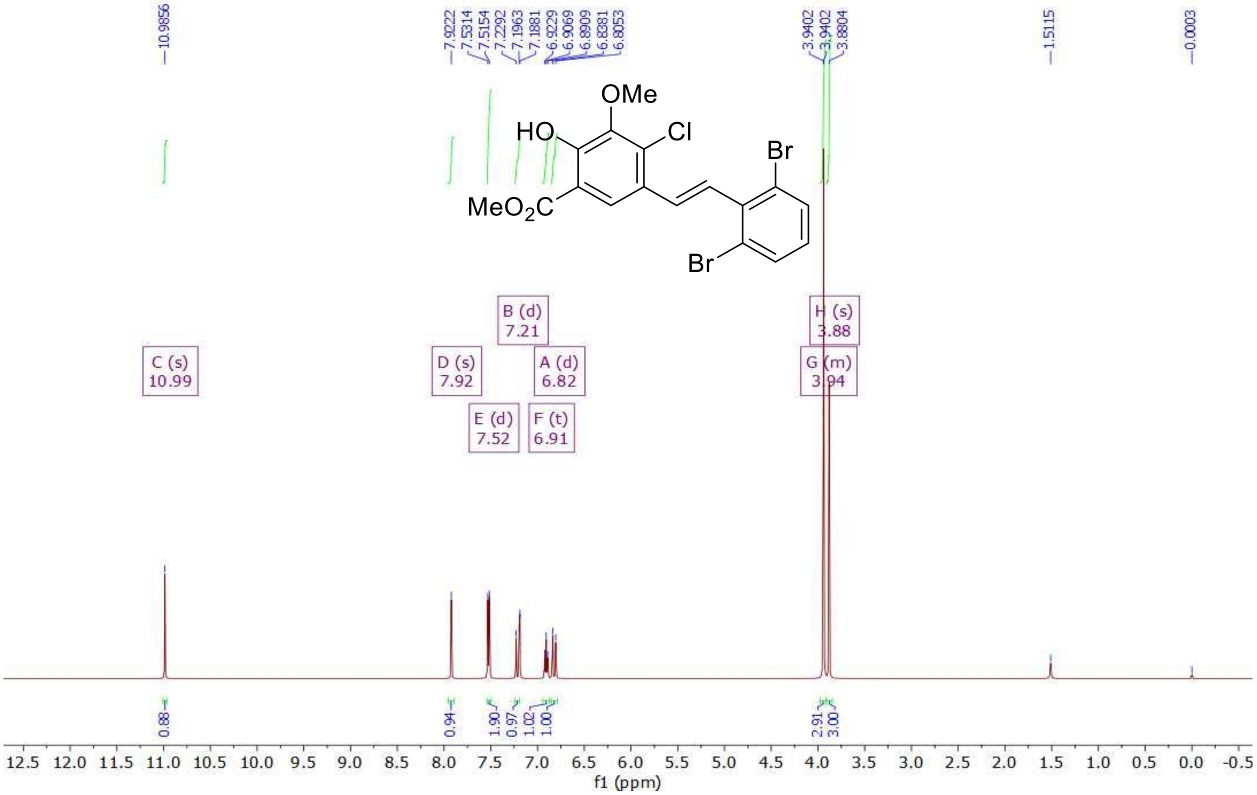


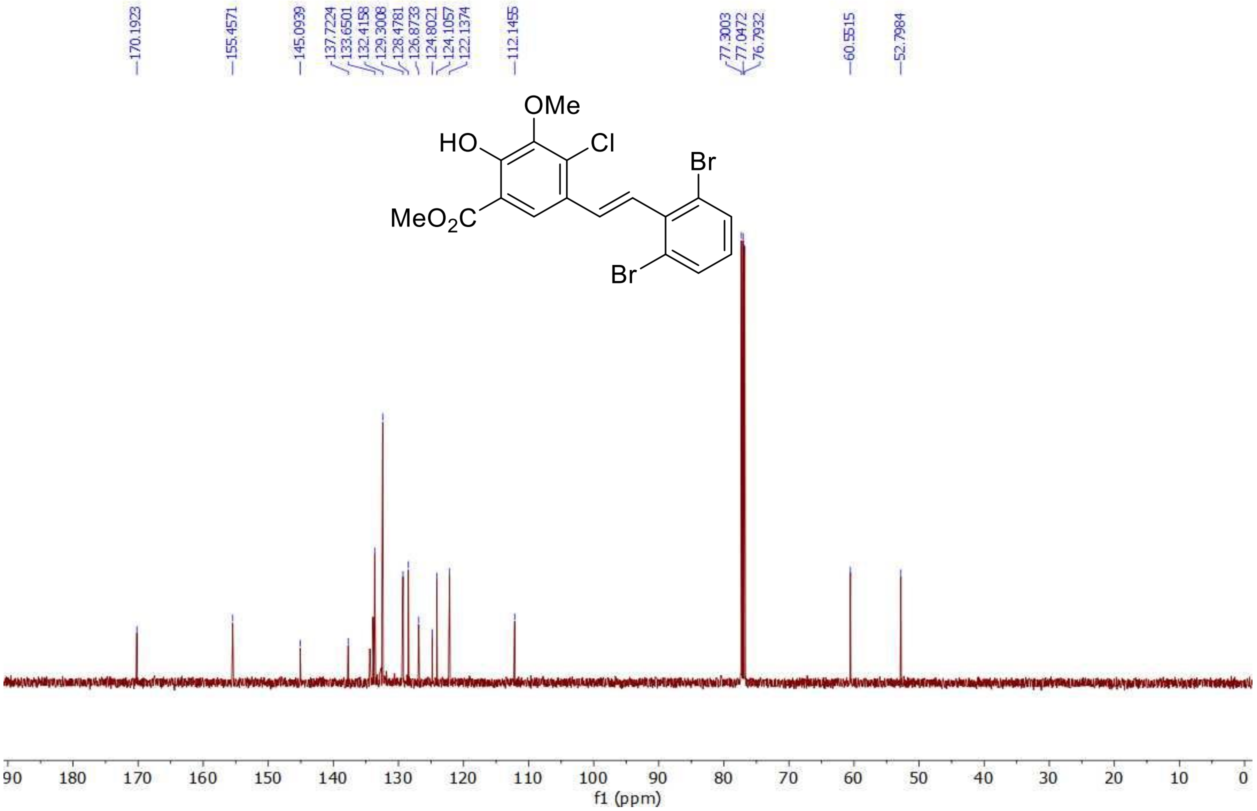

## $^{1}$H NMR (400 MHz, CDCl$_3$) and $^{13}$C NMR (100 MHz, CDCl$_3$) of 10x

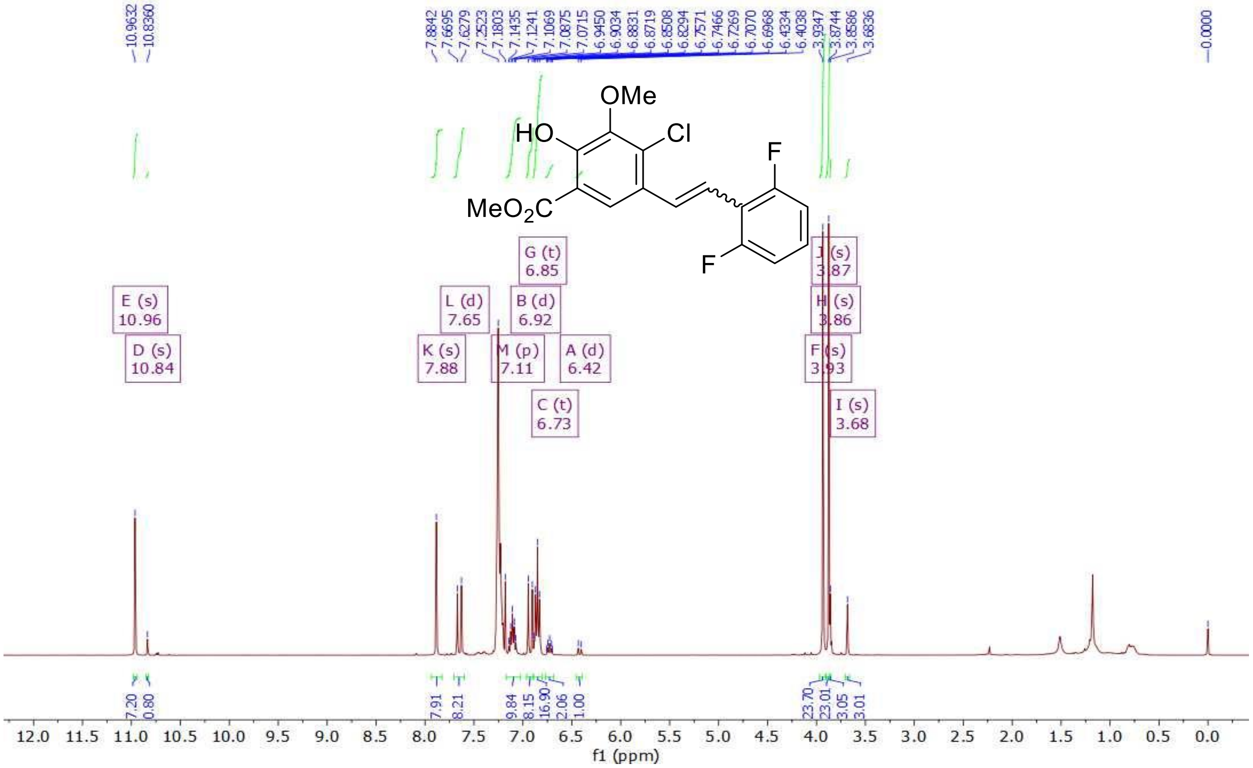


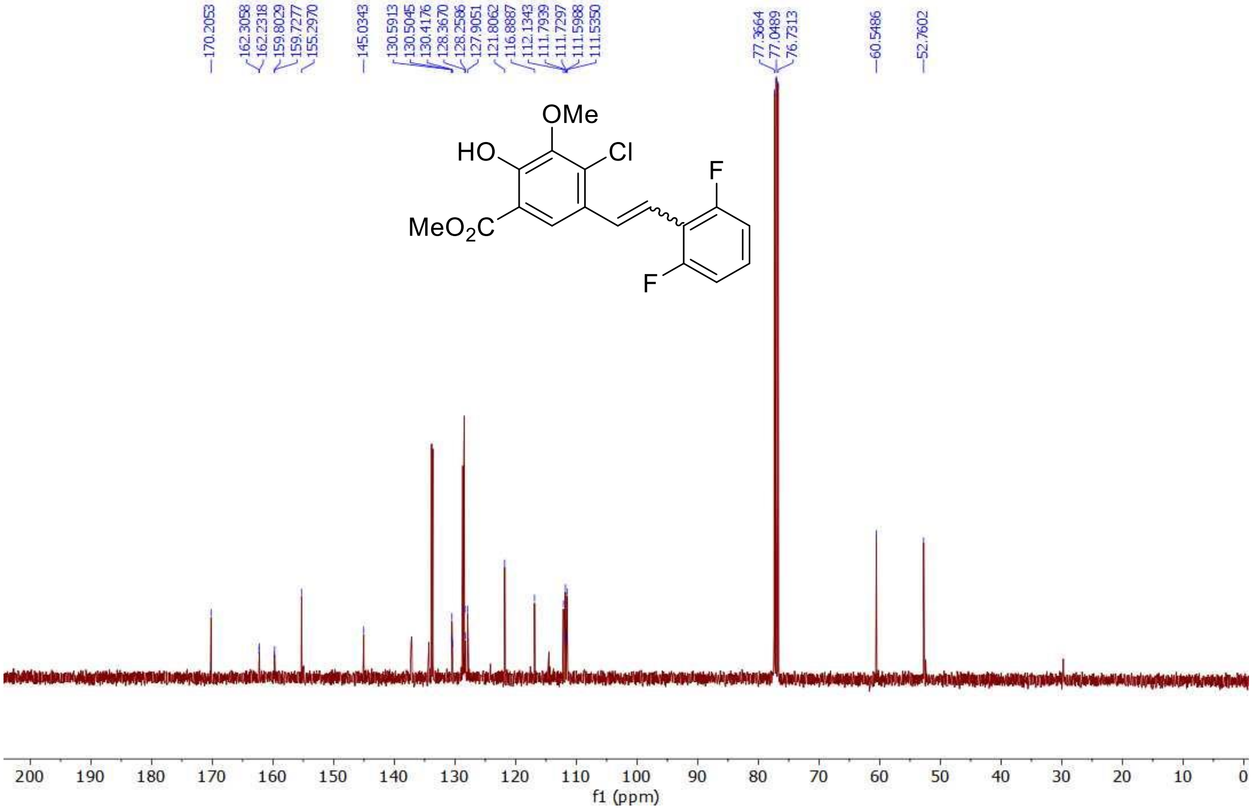

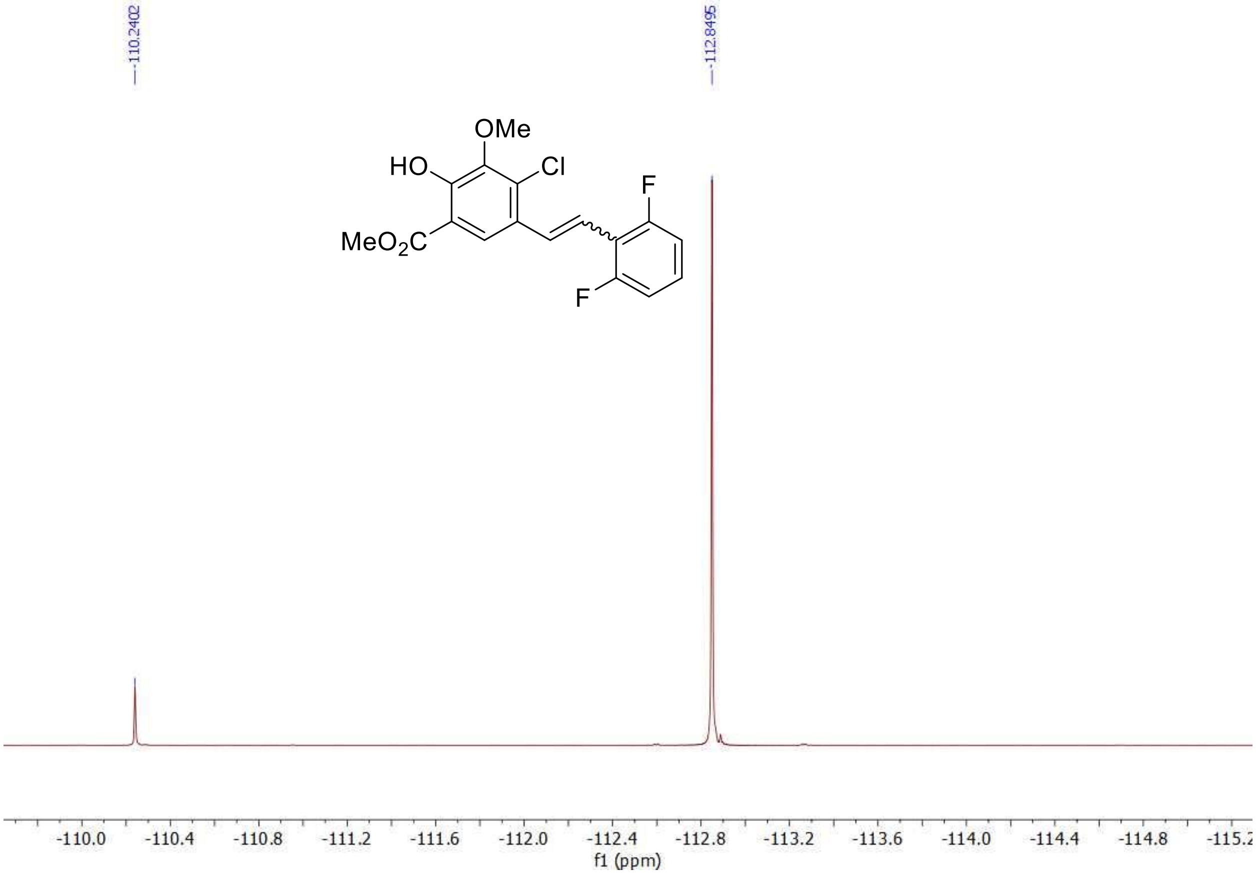

-110.2402
-112.8495
OMe
HO
Cl
F
MeO2C
F
-110.0
-110.4
-110.8
-111.2
-111.6
-112.0
-112.4
-112.8
-113.2
-113.6
-114.0
-114.4
-114.8
f1 (ppm)

**$^{1}H$ NMR (400 MHz, $CDCl_3$) and $^{13}C$ NMR (100 MHz, $CDCl_3$) of *cis*-isomer of 10y**

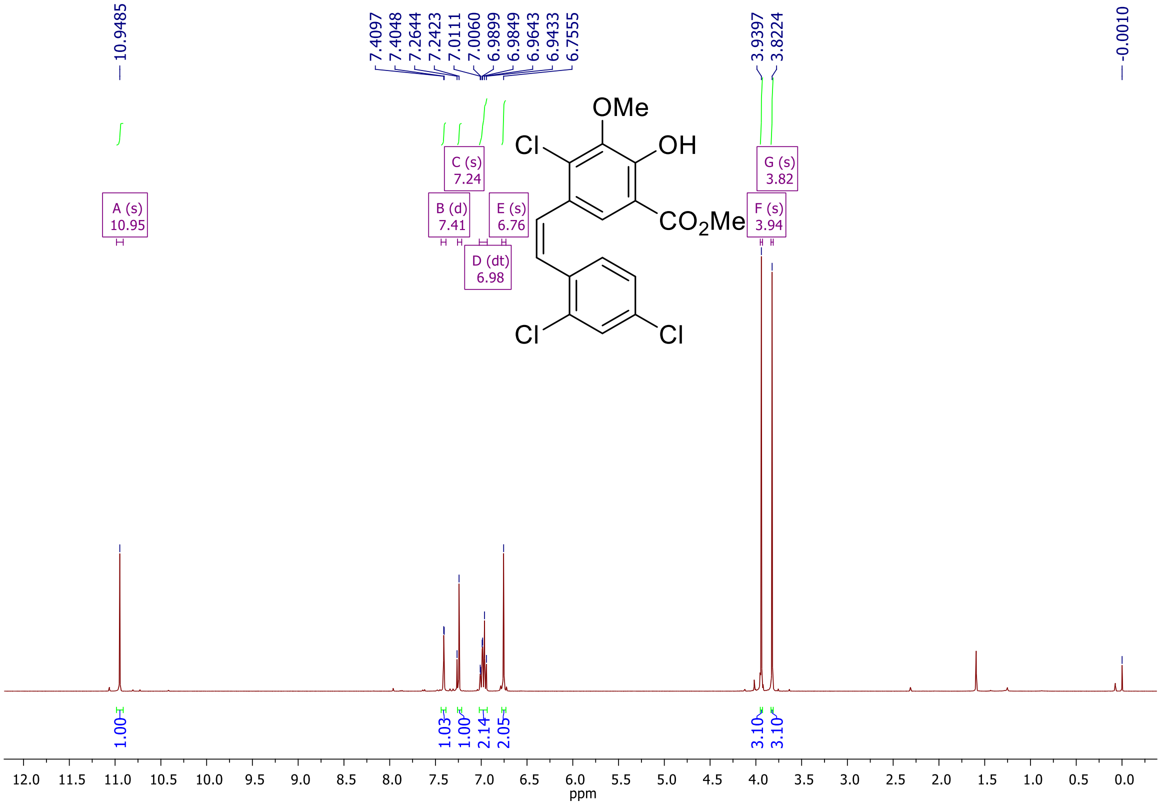


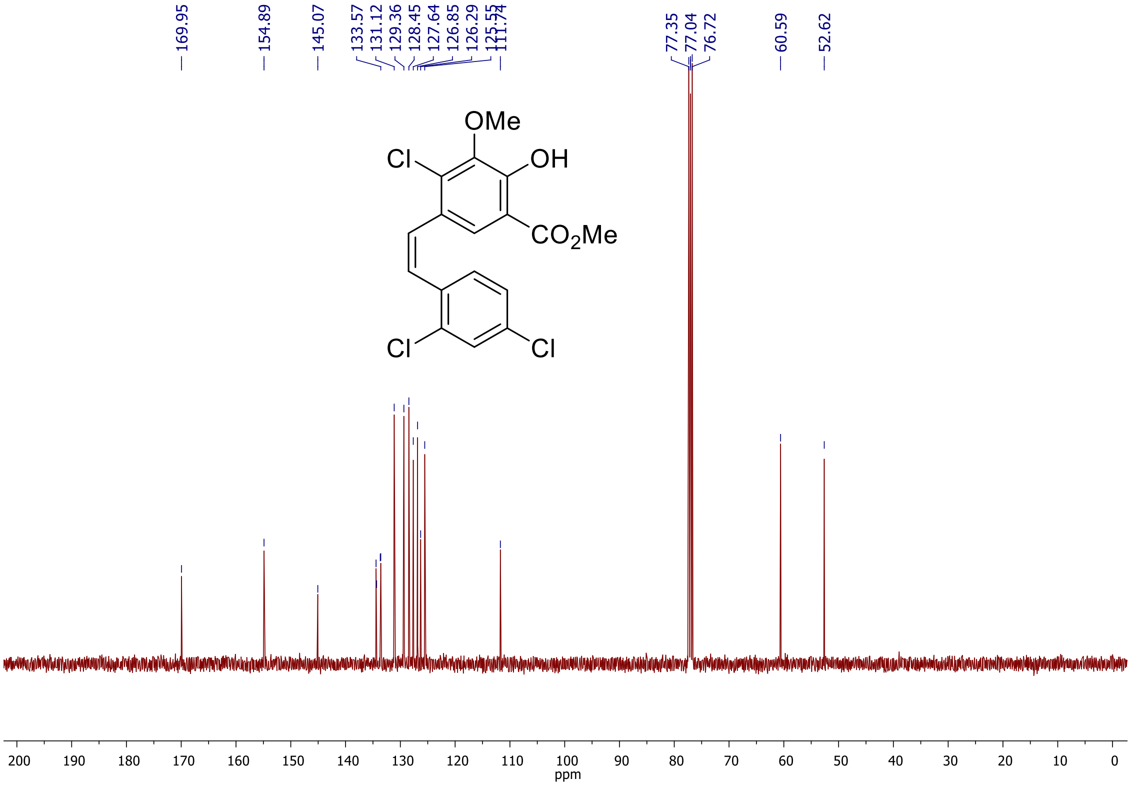

**$^1$H NMR (400 MHz, CDCl$_3$) and $^{13}$C NMR (100 MHz, CDCl$_3$) of *trans*-isomer of 10y**

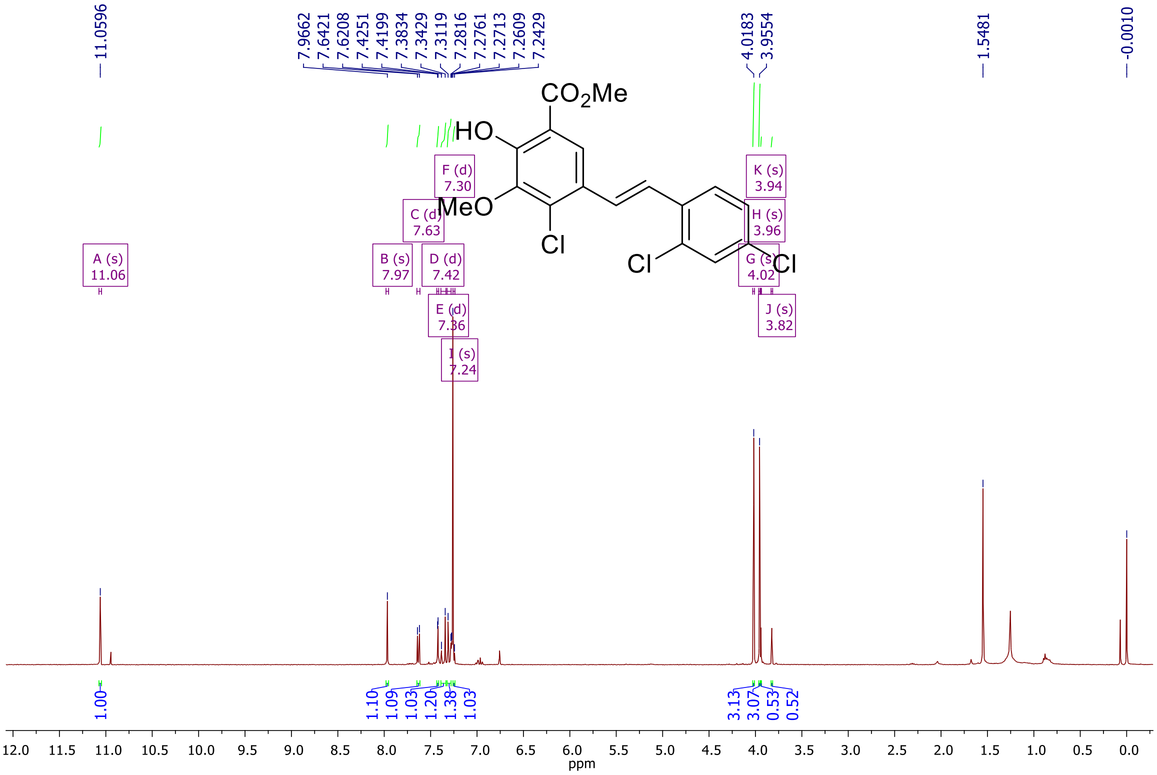

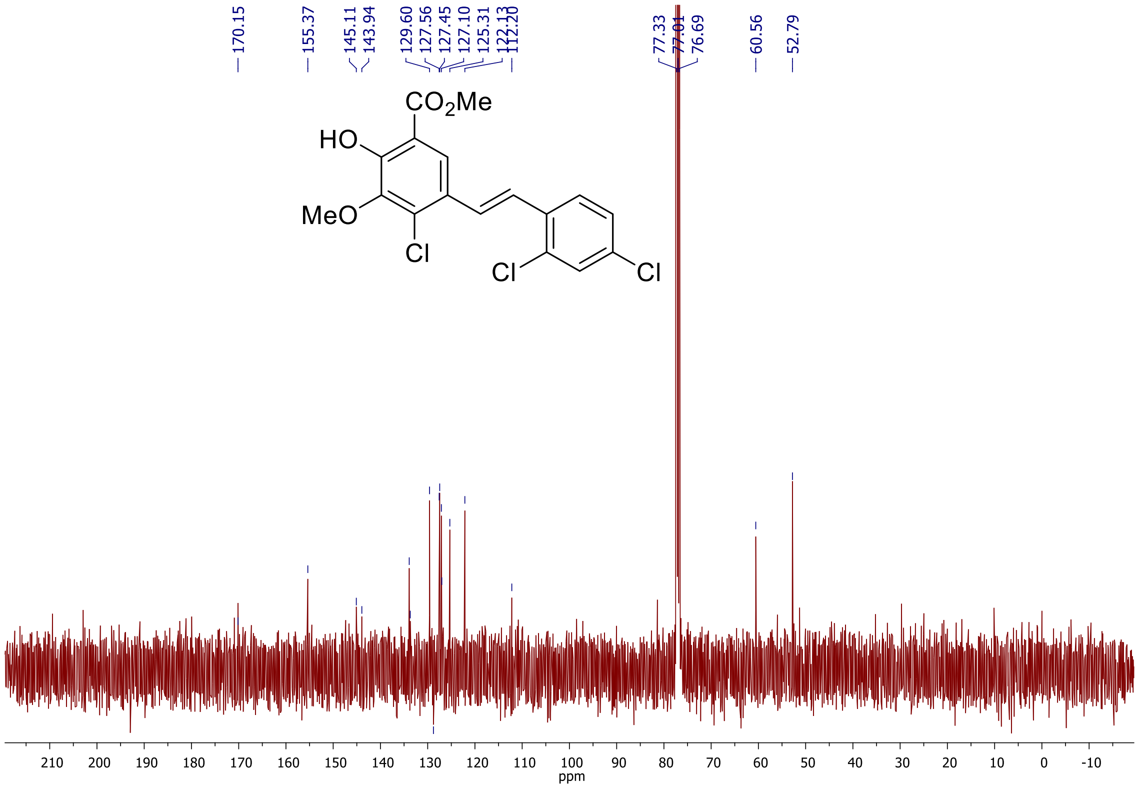

$^{1}H$ NMR (400 MHz, $CDCl_3$) and $^{13}C$ NMR (100 MHz, $CDCl_3$) of 10z

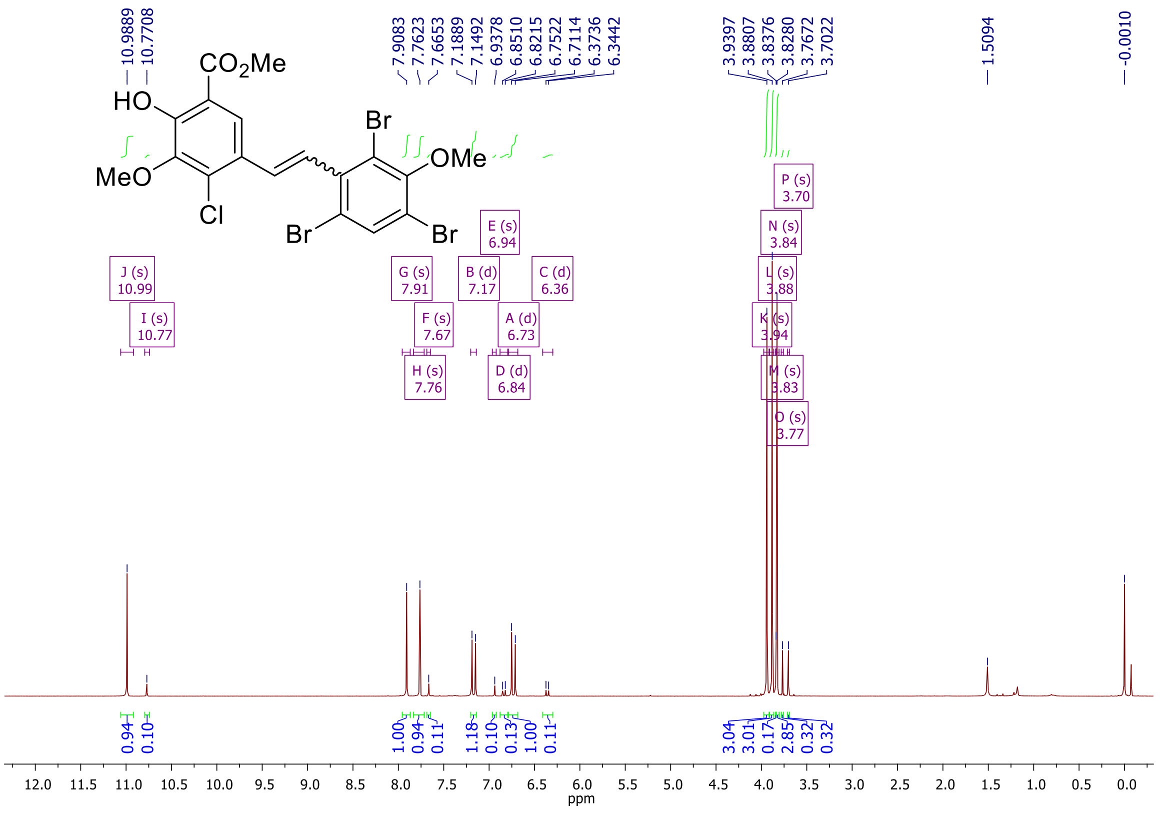

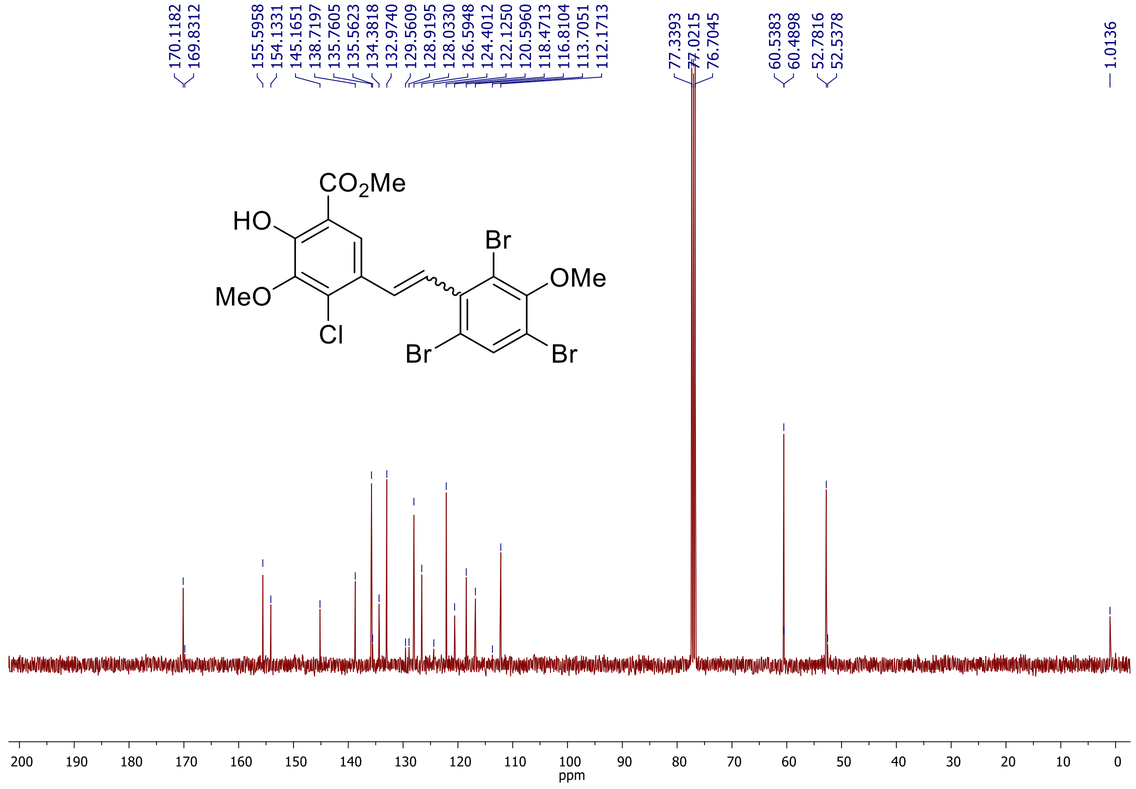

**$^1$H NMR (400 MHz, CDCl$_3$) and $^{13}$C NMR (100 MHz, CDCl$_3$) of 10aa**

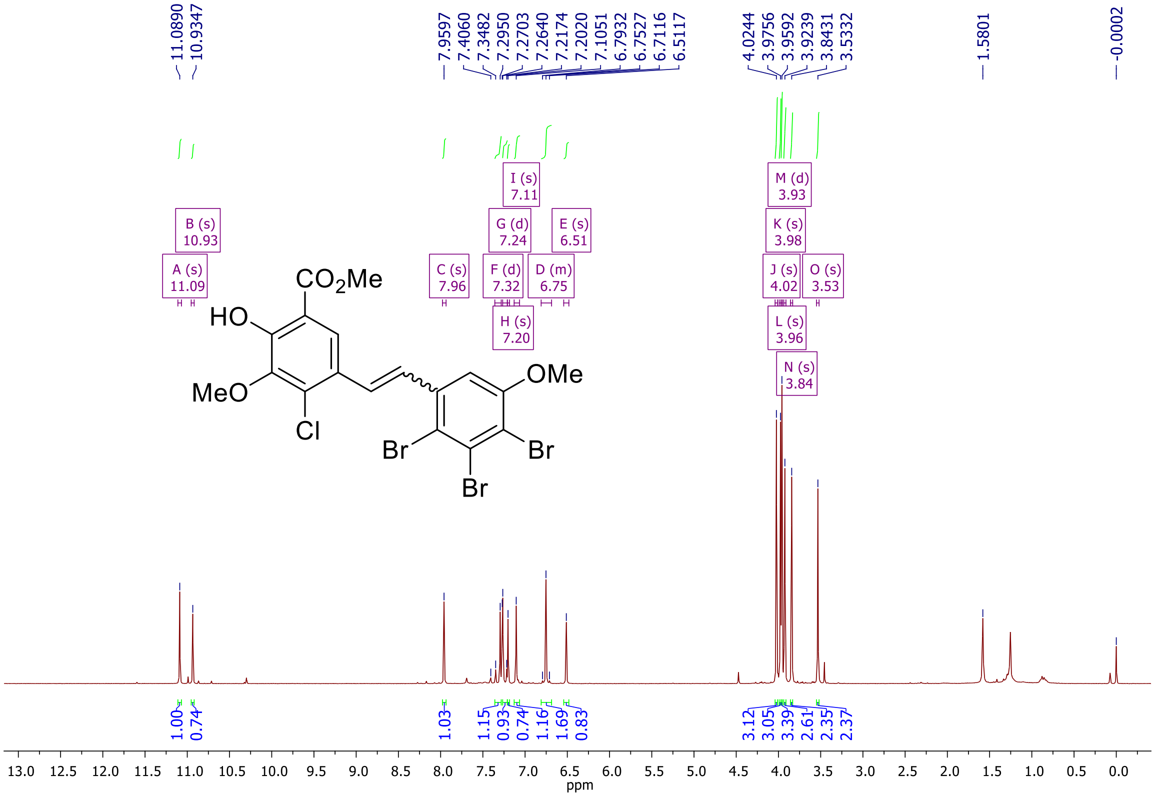


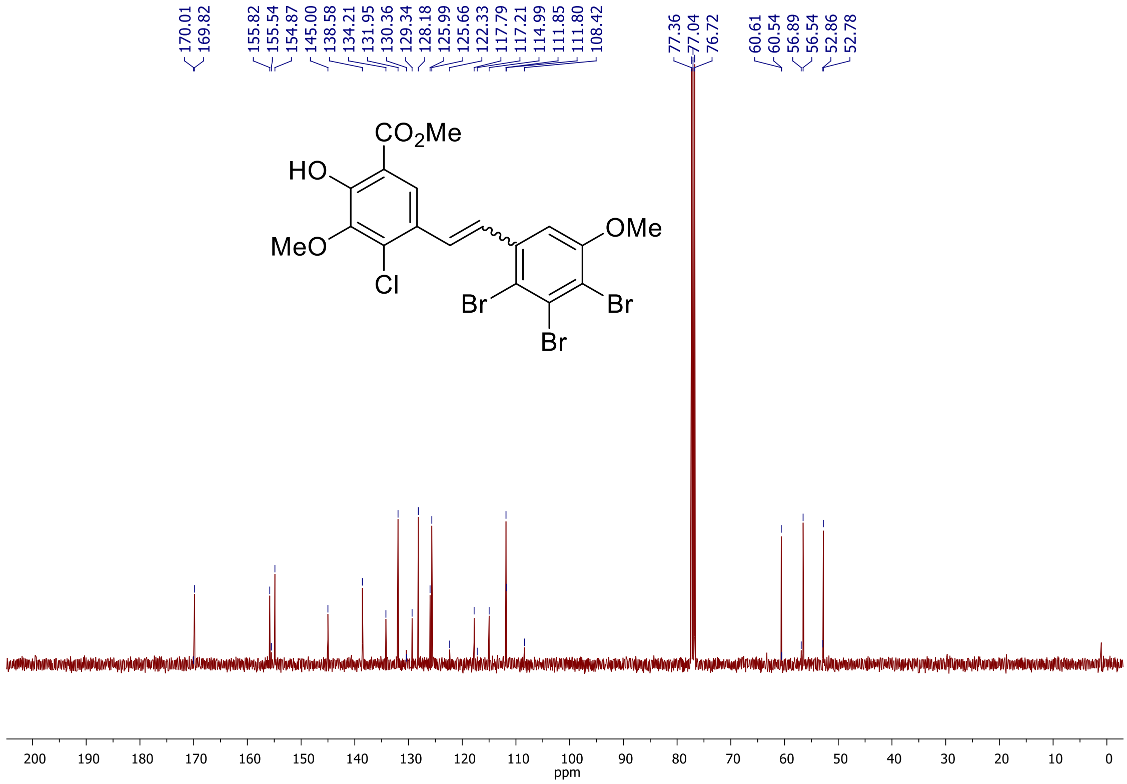

**$^{1}$H NMR (400 MHz, CDCl$_3$) and $^{13}$C NMR (100 MHz, CDCl$_3$) of 11a**

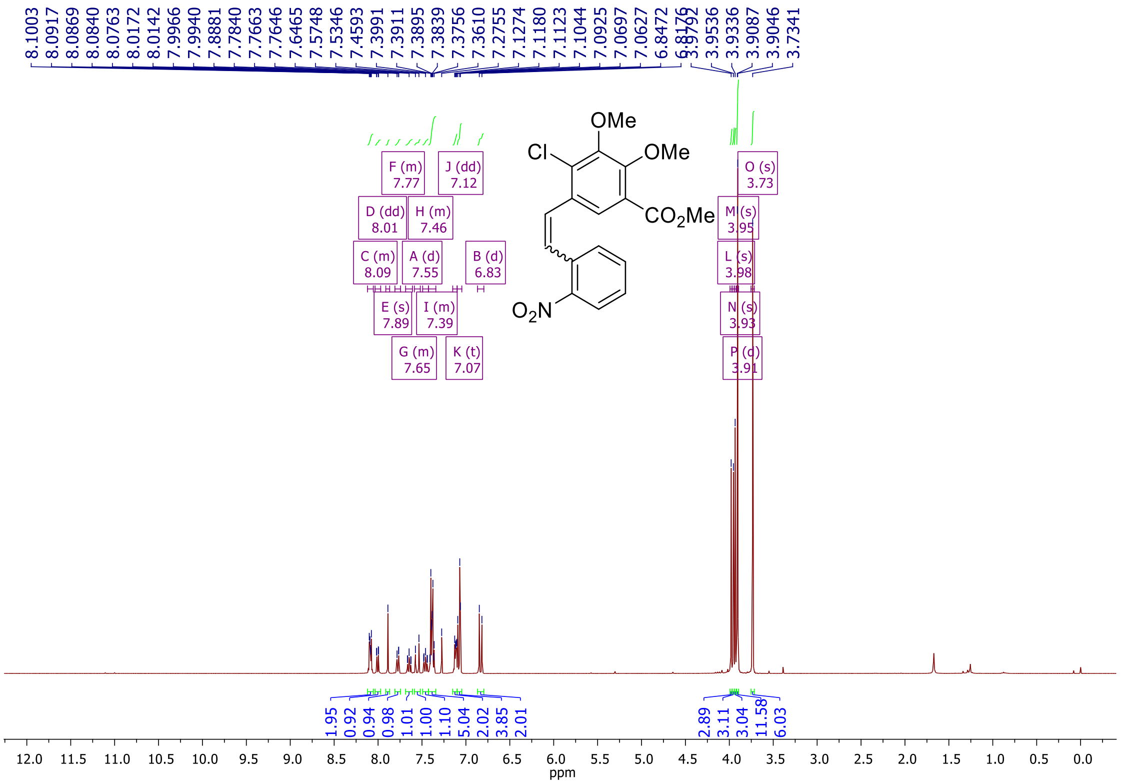

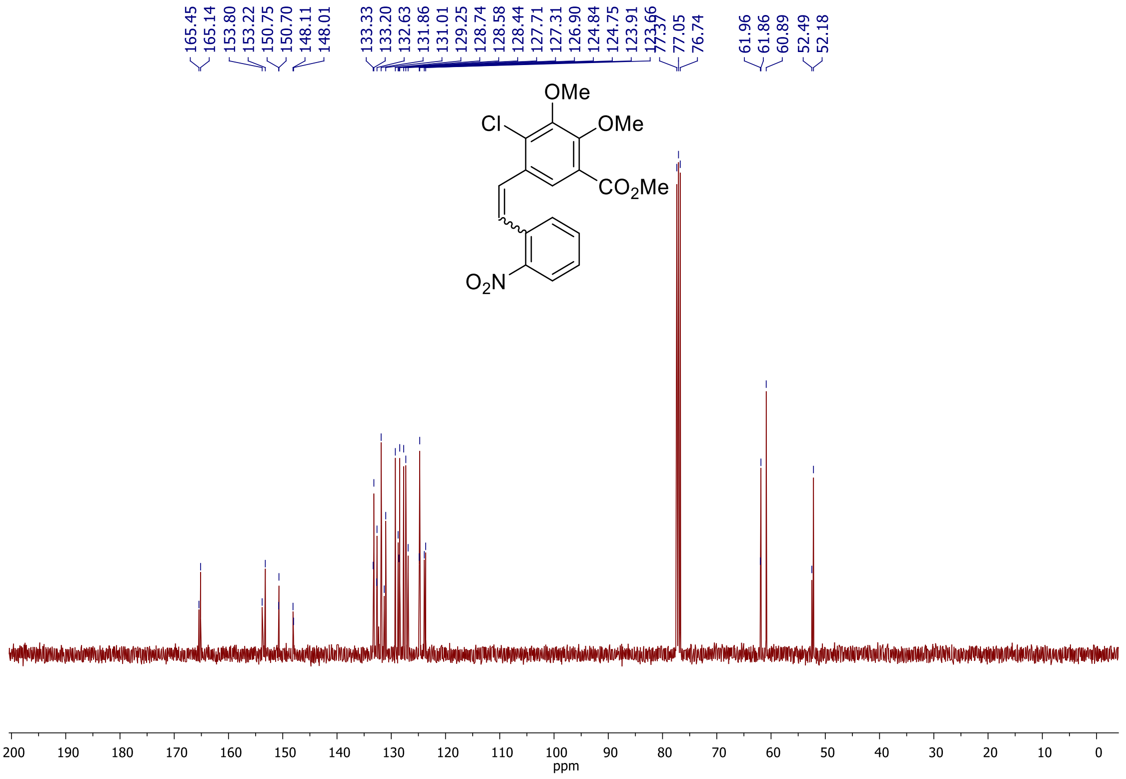

**$^{1}$H NMR (400 MHz, CDCl$_3$) and $^{13}$C NMR (100 MHz, CDCl$_3$) of 11b**

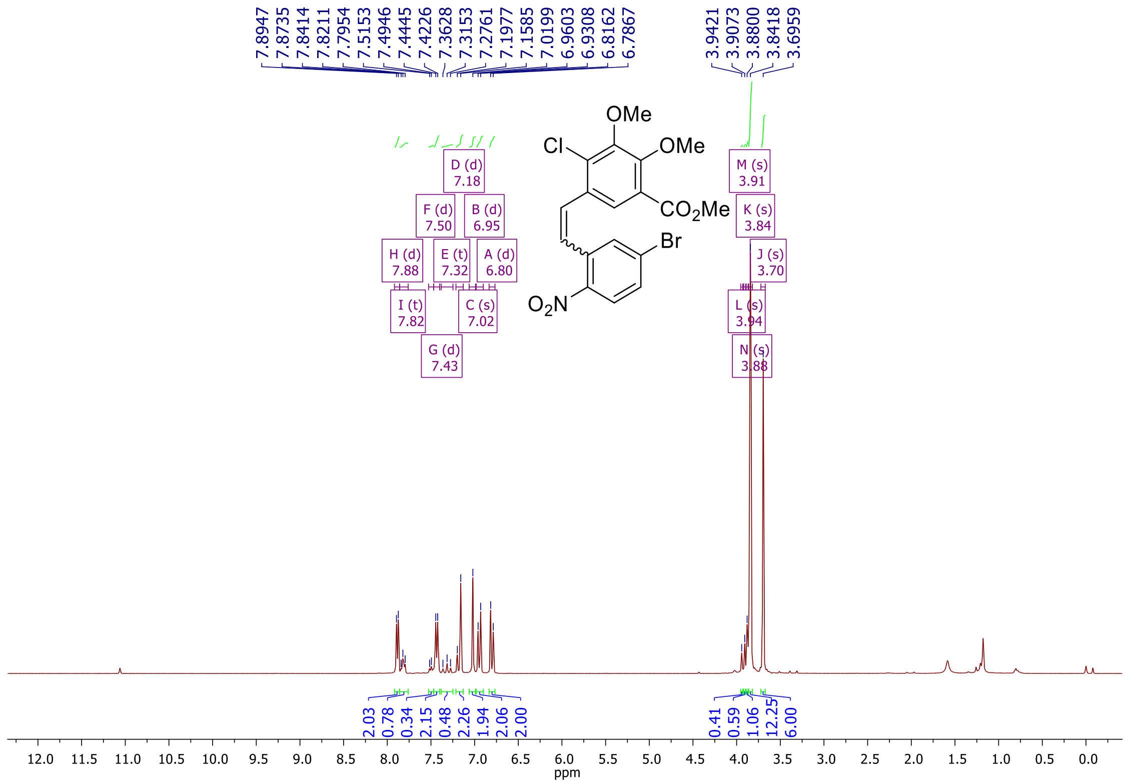


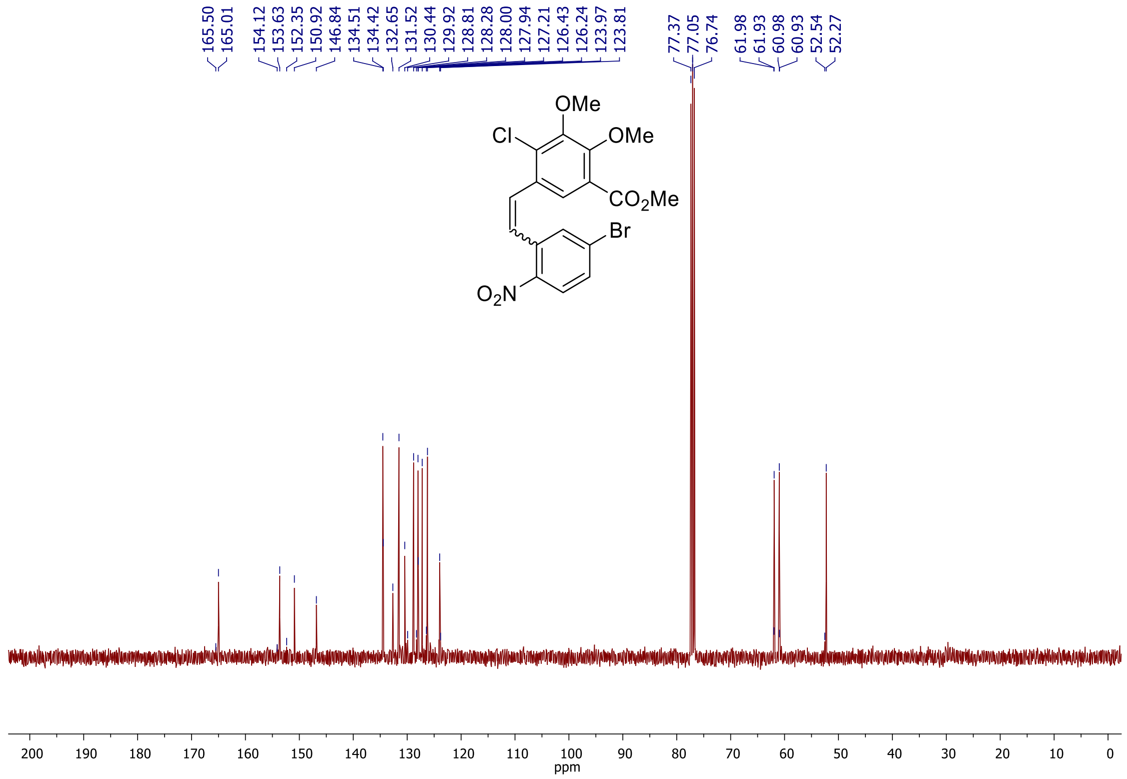

## $^{1}H$ NMR (400 MHz, $CDCl_3$) and $^{13}C$ NMR (100 MHz, $CDCl_3$) of 12a

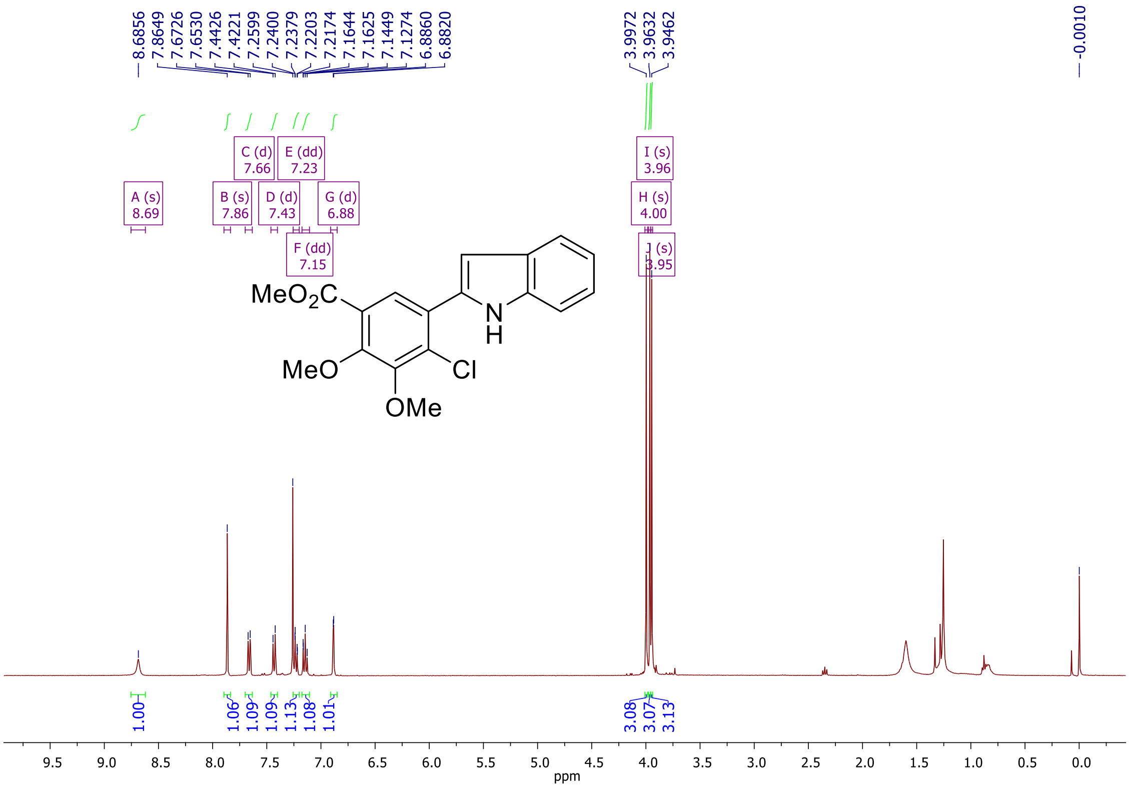


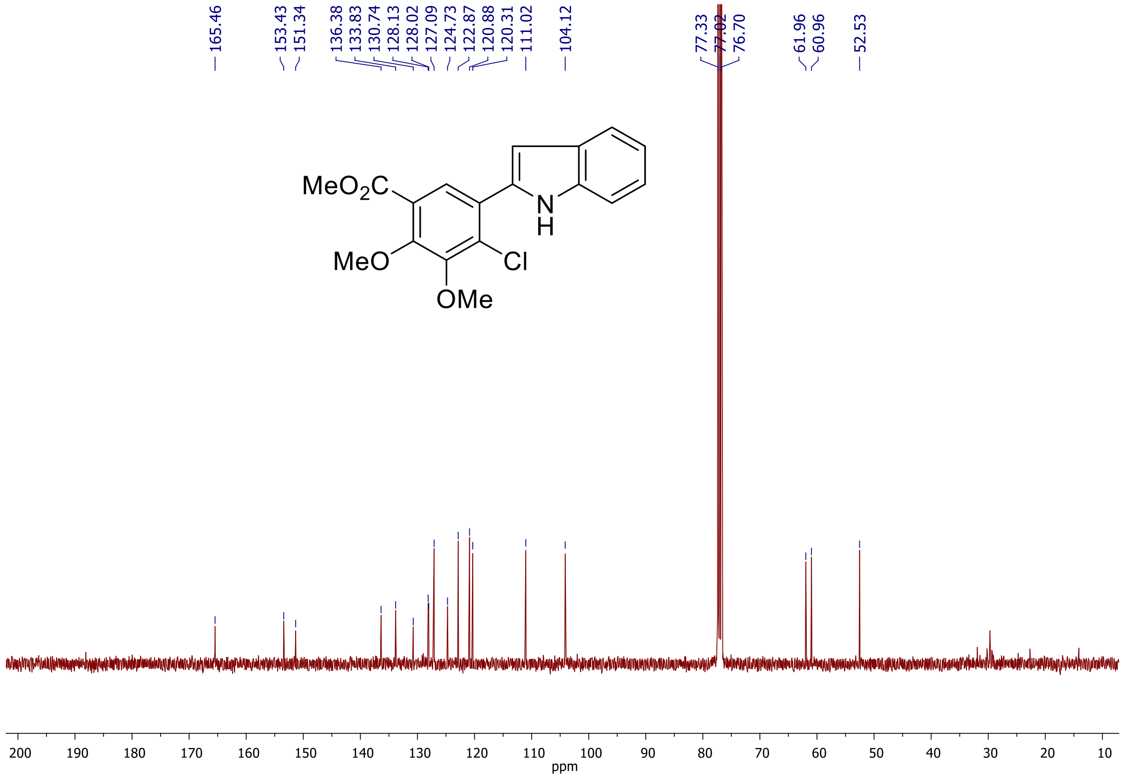

## $^{1}H$ NMR (400 MHz, $CDCl_3$) and $^{13}C$ NMR (100 MHz, $CDCl_3$) of 12b

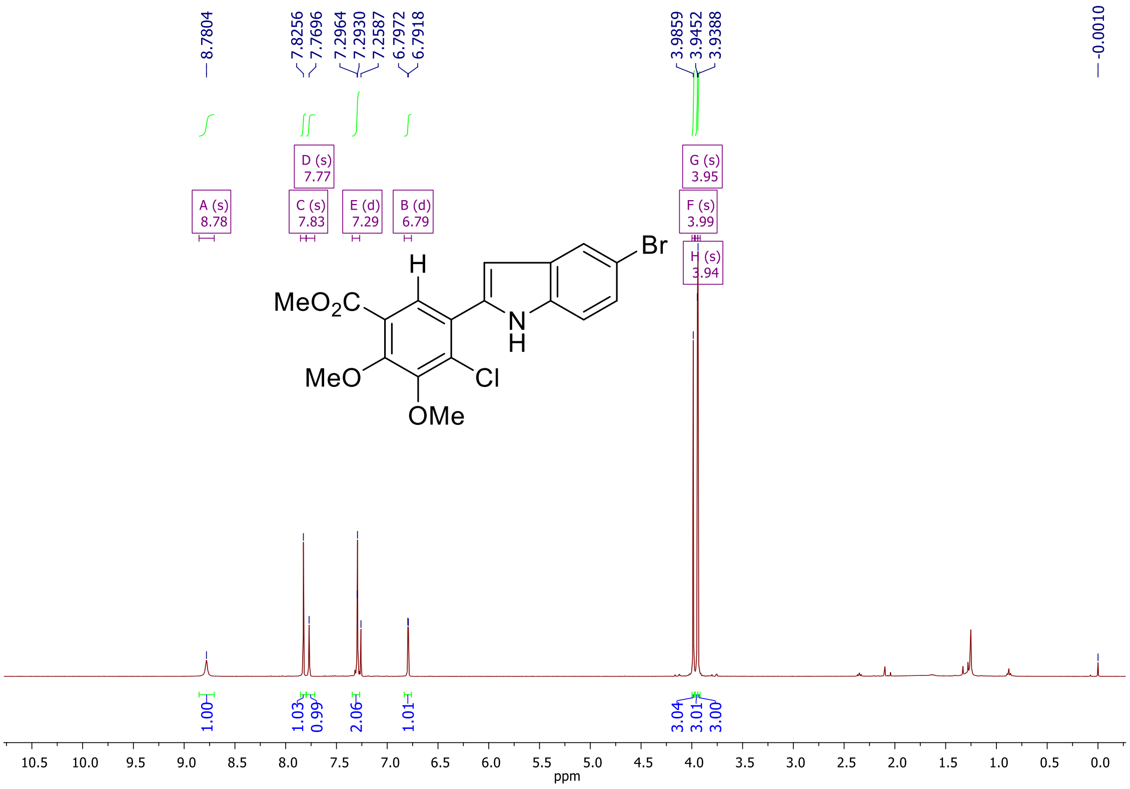

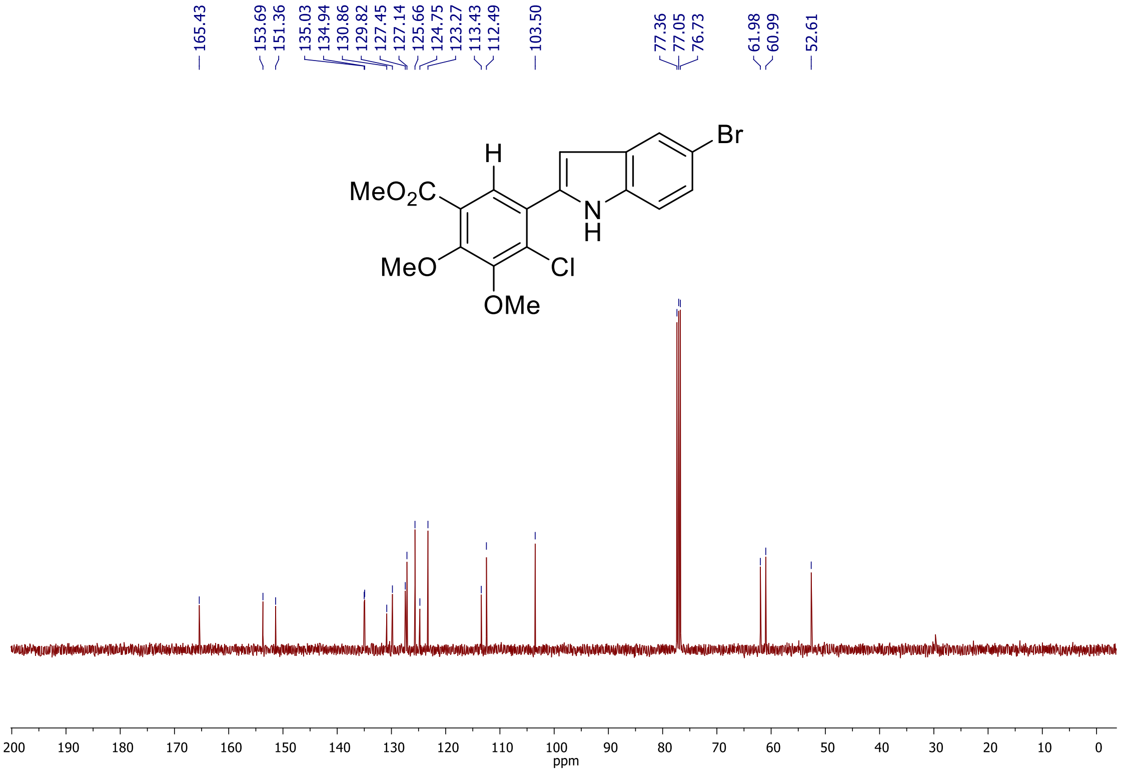

**References**


1 Purification of laboratory chemicals: Perrin, D. D.; Armarego, W. L. F. Pergamon press, 3rd Edition, **1988**.

2 An unusual formation of diarylmethane scaffolds from 4-(halomethyl)cyclohex-2-enone derivatives: Babu, K. R.; Khan, F. A. *Tetrahedron Lett.*, **2015**, *56*, 4067-4070.

3 (a) Li, W. -Y.; Xiong, X.-Q.; Zhao, D.-M.; Shi, Y.-F.; Yang, Z.-H.; Yu, C.; Fan, P.-W.; Cheng, M.-S.; Shen, J.-K.; Quinoline-3-carboxamide Derivatives as Potential Cholesteryl Ester Transfer Protein Inhibitors, *Molecules,* **2012**, *17*, 5497-5507; (b) Zhang, W.; Wu, D.; Zhang, J.; Liu, Y. *Eur. J. Org. Chem*. **2014**, *26*, 5827; (c) Ma, C.; Du, K.; Zhao, Y.; Zhang, L.; Hu, B.; Cheng, M. *Bioorganic & Medicinal Chemistry* **2018**, *26(18)*, 5151.

4 Cadogan, J. I. G.; Mackie, R. K. *Tervalent phosphorus compounds in organic synthesis. Chemical Society Reviews,* **1974,** *3(1),* 87.

5 Dolomanov, O.V., Bourhis, L.J., Gildea, R.J, Howard, J.A.K. & Puschmann, H. *J. Appl. Cryst.* **2009**, *42*, 339-341.

6 Sheldrick, G.M. *Acta Cryst*. **2008**, *A64*, 112-122.

7 Bourhis, L.J., Dolomanov, O.V., Gildea, R.J., Howard, J.A.K., Puschmann, H. *Acta Cryst*. **2015**, *A71*, 59-75.

8 (a) APEX3 Version 2016.5-0 and SAINT+ Version 8.37A. Bruker AXS, Inc., Madison, Wisconsin, USA, **2016**. (b) SADABS-2016/2: L. Krause, R. Herbst-Irmer, G. M. Sheldrick and D. Stalke, *J. Appl. Cryst.*, **2015**, *48*, 3.

9 (a) SHELXT: G. M. Sheldrick, *Acta Cryst*., **2015**, *A71*, 3. (b) SHELXL: G. M. Sheldrick, *Acta Cryst*., **2015**, *C71*, 3.

10 OLEX2: a complete structure solution, refinement, and analysis program: O. V. Dolomanov, L. J. Bourhis, R. J. Gildea, J. A. K. Howard and H. Puschmann, *J. Appl. Cryst.* **2009**, *42*, 339.
